# Biodiversity of Mineral Nutrient and Trace Element Accumulation in *Arabidopsis thaliana*

**DOI:** 10.1371/journal.pone.0035121

**Published:** 2012-04-27

**Authors:** Ivan Baxter, Christian Hermans, Brett Lahner, Elena Yakubova, Marina Tikhonova, Nathalie Verbruggen, Dai-yin Chao, David E. Salt

**Affiliations:** 1 Agricultural Research Service Plant Genetics Research Unit, Donald Danforth Plant Science Center, United States Department of Agriculture, St. Louis, Missouri, United States of America; 2 Center for Plant Stress Physiology, Purdue University, West Lafayette, Indiana, United States of America; 3 Laboratory of Plant Physiology and Molecular Genetics, Université Libre de Bruxelles, Brussels, Belgium; 4 Institute of Biological and Environmental Sciences, University of Aberdeen, Aberdeen, Scotland, United Kingdom; KU Leuven, Belgium

## Abstract

In order to grow on soils that vary widely in chemical composition, plants have evolved mechanisms for regulating the elemental composition of their tissues to balance the mineral nutrient and trace element bioavailability in the soil with the requirements of the plant for growth and development. The biodiversity that exists within a species can be utilized to investigate how regulatory mechanisms of individual elements interact and to identify genes important for these processes. We analyzed the elemental composition (ionome) of a set of 96 wild accessions of the genetic model plant *Arabidopsis thaliana* grown in hydroponic culture and soil using inductively coupled plasma mass spectrometry (ICP-MS). The concentrations of 17–19 elements were analyzed in roots and leaves from plants grown hydroponically, and leaves and seeds from plants grown in artificial soil. Significant genetic effects were detected for almost every element analyzed. We observed very few correlations between the elemental composition of the leaves and either the roots or seeds. There were many pairs of elements that were significantly correlated with each other within a tissue, but almost none of these pairs were consistently correlated across tissues and growth conditions, a phenomenon observed in several previous studies. These results suggest that the ionome of a plant tissue is variable, yet tightly controlled by genes and gene×environment interactions. The dataset provides a valuable resource for mapping studies to identify genes regulating elemental accumulation. All of the ionomic data is available at www.ionomicshub.org.

## Introduction

Broad variation in the physical and chemical properties of soil provide a large challenge to plant breeders attempts to develop crops to feed the worlds growing population [1]. In order to grow on marginal or degraded land, or with fewer inputs, breeders will need to identify loci or genes that can promote growth in these environments. Some wild plants show specific adaptations to certain soils, and many efforts have been directed towards identification of the mechanisms permitting growth in these environments [2]–[4]. Many of these studies have been limited by the lack of systems biology resources and appropriate mapping populations, though progress has been made in some species [5]–[9]. Accessions of the genetic model plant *A. thaliana* have been identified in a wide variety of environments [10] and genotypes that can withstand diverse laboratory conditions have also been identified [11]–[13]. When combined with the wealth of genetic and systems biology resources available for *A. thaliana* these lines can be potentially utilized as resources for understanding the physiology of adaptation and underlying genetics. One mechanism that plants have evolved to grow in widely varying soil chemistries is to alter the elemental composition of their tissues. Recently, there have been substantial efforts to measure the nutrient and trace element composition of plants, also known as the ionome, to understand its genetic and environmental regulation [14]–[16], and its effect on growth [17].

Elemental uptake, distribution and storage processes involve multiple molecular components including transporters, channels, chelators and the genes that encode and regulate them. Processes that alter the physiological properties such as root architecture and transpiration can also affect elemental accumulation [18]. Interestingly, many of these changes can affect multiple elements. Altering the Fe and P availability in the soil leads to reproducible and predictable alterations in five and six elements respectively in *A. thaliana* [19]. The alterations in elements in response to Fe deficiency are likely due to well-characterized molecular mechanisms. However, the physiological and molecular drivers of the elemental response to phosphate and the rules governing relationships between many other elements are far from clear. One method of elucidating these rules is to identify the genes controlling the accumulation of individual elements or groups of highly correlated elements. By screening mutant populations for alterations in the ionome, two genes that alter the sphingolipid and suberin pathways were identified which control root processes involved in mineral ion homeostasis and water relations [18], [20].

**Table 1:** Summary of Root Hydroponic and Leaf Hydroponic Experiments.

	Root Hydro						Leaf Hydro					
	Min	Max	Ratio	Mean	SD	Heritability	Min	Max	Ratio	Mean	SD	Heritability
Li	NA	NA	NA	NA	NA	NA	NA	NA	NA	NA	NA	NA
B	12.25	27.67	2.3	20.74	2.75	0.58	41.75	74.50	1.8	56.50	7.47	0.74
Na	464	4720	10.2	1678	721	0.64	138	561	4.1	265	68	0.76
Mg	1662	3032	1.8	2318	279	0.62	7558	11562	1.5	9102	962	0.76
P	9595	16483	1.7	12046	1377	0.79	6774	11409	1.7	9034	1019	0.72
S	13115	20479	1.6	16596	1758	0.70	6874	13318	1.9	9818	1221	0.70
K	43803	79510	1.8	60618	6774	0.68	36924	72417	2.0	57051	7026	0.74
Ca	7978	18389	2.3	10134	1706	0.45	43486	71776	1.7	54727	6379	0.82
Mn	54	494	9.2	162	89	0.90	61	142	2.3	101	15	0.92
Fe	1328	2550	1.9	1877	260	0.78	93	141	1.5	118	10	0.83
Co	0.19	1.62	8.7	0.53	0.27	0.69	0.12	0.35	3.0	0.17	0.05	0.55
Ni	3.21	12.36	3.9	5.50	1.71	0.51	4.58	7.90	1.7	6.09	0.76	0.59
Cu	13.50	40.31	3.0	23.12	5.89	0.79	5.33	9.60	1.8	6.80	0.94	0.75
Zn	222	790	3.6	427	101	0.82	48	134	2.8	76	16	0.84
As	NA	NA	NA	NA	NA	NA	NA	NA	NA	NA	NA	NA
Se	NA	NA	NA	NA	NA	NA	NA	NA	NA	NA	NA	NA
Rb	1.51	5.01	3.3	2.42	0.62	0.64	0.80	1.59	2.0	1.07	0.14	0.80
Mo	7.76	372	48.0	85.5	68.7	0.95	1.13	52.0	46.0	11.2	10.1	0.98
Cd	0.24	1.93	8.1	0.66	0.32	0.81	0.06	0.29	5.3	0.11	0.04	0.86
S/Se ratio	NA	NA	NA	NA	NA	NA	NA	NA	NA	NA	NA	NA
K/RB ratio	14350	40630	2.8	26910	6273	0.67	34710	75050	2.2	53970	7442	0.70

Minimum (Min), maximum (Max), Max/Min (Ratio), mean and standard deviation (SD) values are calculated off the outlier removed, normalized line averages. Italics: The line factor was not significant in an ANOVA of a linear model for that element. Elements not measured in a given experiment are noted with an ‘NA’.

**Table 2:** Summary of Soil Leaf 1 and Soil Leaf 2 Experiments.

	Soil Leaf 1						Soil Leaf 2					
	Min	Max	Ratio	Mean	SD	Heritability	Min	Max	Ratio	Mean	SD	Heritability
Li	4.77	9.00	1.9	6.94	0.99	0.36	4.90	9.44	1.9	7.22	1.08	0.30
B	143.58	338.38	2.4	241.46	33.81	0.39	60.81	112.55	1.9	83.76	8.89	0.54
Na	439	2908	6.6	994	462	0.83	325	2395	7.4	798	451	0.81
Mg	13612	23955	1.8	19541	1909	0.63	10752	16304	1.5	13454	1182	0.66
P	7768	16597	2.1	11425	1548	0.39	6275	10905	1.7	8360	945	0.59
S	NA	NA	NA	NA	NA	NA	7435	14455	1.9	10719	1535	0.48
K	42876	81804	1.9	56689	7774	0.47	20773	48153	2.3	31377	4730	0.52
Ca	25137	41702	1.7	35288	3187	0.54	21322	32937	1.5	27361	2213	0.48
Mn	70	189	2.7	116	22	0.31	66	162	2.4	99	18	0.41
Fe	77	110	1.4	91	7	0.33	52	71	1.4	61	4	0.26
Co	0.45	1.64	3.6	0.86	0.22	0.38	0.79	2.18	2.7	1.33	0.27	0.40
Ni	1.45	2.57	1.8	2.03	0.22	0.18	1.32	4.37	3.3	3.01	0.66	0.19
Cu	2.04	5.27	2.6	3.33	0.63	0.24	0.56	1.50	2.7	1.00	0.21	0.21
Zn	51	157	3.1	90	17	0.52	35	96	2.7	70	11	0.60
As	0.16	1.04	6.5	0.35	0.10	0.29	0.12	0.35	3.0	0.21	0.05	0.19
Se	12.73	28.86	2.3	17.99	2.77	0.46	4.74	9.37	2.0	6.74	0.90	0.34
Rb	NA	NA	NA	NA	NA	NA	NA	NA	NA	NA	NA	NA
Mo	0.29	4.55	15.8	1.77	0.92	0.62	0.26	10.3	40.2	2.60	1.53	0.56
Cd	1.69	5.04	3.0	3.29	0.74	0.34	2.16	5.68	2.6	3.43	0.78	0.45
S/Se ratio	NA	NA	NA	NA	NA	NA	1306	1939	1.5	1557	128	0.27
K/RB ratio	NA	NA	NA	NA	NA	NA	NA	NA	NA	NA	NA	NA

Minimum (Min), maximum (Max), Max/Min (Ratio), mean and standard deviation (SD) values are calculated off the outlier removed, normalized line averages. Italics: The line factor was not significant in an ANOVA of a linear model for that element. Elements not measured in a given experiment are noted with an ‘NA’.

**Table 3:** Summary of Soil Seed Experiment.

	Min	Max	Ratio	Mean	SD	Heritability
Li	0.23	3.37	14.4	1.16	0.65	0.46
B	8.98	40.59	4.5	14.84	4.78	0.39
Na	13	416	31.2	99	86	0.49
Mg	2911	5295	1.8	3990	451	0.42
P	7364	10822	1.5	8873	619	0.49
S	8195	20024	2.4	12894	1800	0.65
K	5668	12698	2.2	8466	1652	0.59
Ca	3009	7337	2.4	4846	694	0.59
Mn	33	111	3.3	63	15	0.59
Fe	14	48	3.4	28	5	0.28
Co	0.10	0.70	7.2	0.30	0.12	0.63
Ni	1.31	6.37	4.9	2.50	0.90	0.55
Cu	4.68	21.05	4.5	8.63	2.93	0.66
Zn	62	172	2.8	90	17	0.54
As	0.36	4.44	12.5	1.59	0.74	0.59
Se	2.02	9.28	4.6	6.02	1.45	0.45
Rb	2.35	7.63	3.3	3.55	0.93	0.46
Mo	0.22	3.60	16.3	1.32	0.60	0.84
Cd	0.27	1.38	5.1	0.67	0.24	0.49
S/Se ratio	1703	4701	2.760422783	2458	508	0.42
K/RB ratio	1135	3917	3.451101322	2516	426	0.54

Minimum (Min), maximum (Max), Max/Min (Ratio), mean and standard deviation (SD) values are calculated off the outlier removed, normalized line averages. Italics: The line factor was not significant in an ANOVA of a linear model for that element. Elements not measured in a given experiment are noted with an ‘NA’.

Finding genes through mutant screens is a laborious process, and is limited to the single genetic background that was mutagenized. Natural populations contain a large amount of standing variation that can be exploited for gene identification. Coupled with recent developments in genotyping that enable association mapping methodologies for gene identification this variation is an attractive target for phenotyping approaches such as ionomics [21]. Accessions of the genetic model plant *A. thaliana* have been collected from a wide geographical area encompassing many different soil types. The compact growth habitat of *A. thaliana* also makes it amenable to the large common garden experiments required for association mapping studies of this type.

In this study, we used ionomics to analyze several tissues from a diverse panel of 96 *A. thaliana* accessions grown in hydroponic culture or on artificial soil. This effort allowed us to compare the accumulation of elements between different tissues, and the correlations of elements within tissues. Furthermore, it allowed us to identify accessions with extreme accumulations of all the elements measured.

**Figure 1:**
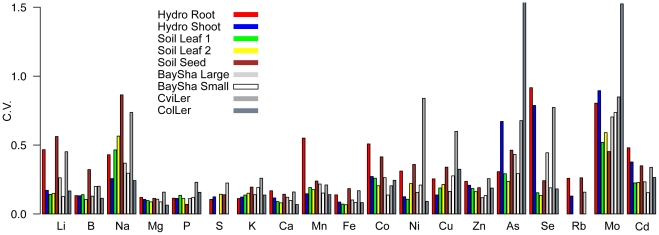
Coefficiant of variation (C.V., standard deviation(line averages)/mean( line averages)). BaySha Large, BaySha Small, CviLer and ColLer are RIL populations analyzed in [24].

## Results

Nordborg et al. selected 96 wild accessions of *A. thaliana*, including 25 pairs of accessions collected extremely close to each other (i.e. a few hundred meters or less) to survey the available genetic diversity [22]. We used this population of accessions as the basis for a screen of the biodiversity of elemental accumulation in roots, leaves and seeds from plants grown hydroponically or in soil. In total we analyzed the concentrations of 17–19 elements (S and Rb were only measured in some of the sets and Li, Co, Se and As were not added to the hydroponic growth medium) in roots and leaves from hydroponically grown plants, two sets of leaves from soil grown plants, and seeds from soil grown plants (Tables 1, 2, 3). With the exception of Ni and As in the second soil leaf experiment, there was a significant effect of genotype on all elements in all experiments (*p*<0.01 with a Bonferroni correction), indicating that the ionome as a whole is under genetic control. For the soil experiments where variability between independent experimental blocks is harder to control than hydroponics, we included two, three or four control accessions in common in each block of plants. Data derived from plants grown in each block were normalized using those common controls, eliminating some of the systematic variation between blocks (Tables 1, 2, 3) [23]. The heritability was quite high in the hydroponics experiments (0.54–0.98), while soil grown leaves (0.18–0.81) and seeds (0.28–0.84) had a few elements with lower heritability. Where both elements were measured, we included the ratio of the chemical analogs S/Se and K/Rb, both of which displayed significant, heritable variation.

**Table 4:** Significant (p<0.001) Correlations between Tissues for each Element.

	HR toHL	HL toSL1	HL toSL2	SL1 toSL2	SL1 toSS	SL2 toSS
Li	nm	nm	Nm	0.39	ns	ns
B	ns	ns	Ns	0.5	ns	ns
Na	ns	0.53	0.55	0.84	ns	ns
Mg	ns	0.41	0.29	0.5	ns	ns
P	0.28	0.33	0.35	0.52	ns	ns
S	ns	nm	0.54	nm	nm	ns
K	ns	0.44	0.44	0.6	ns	ns
Ca	ns	0.47	Ns	0.43	ns	ns
Mn	ns	ns	Ns	ns	ns	ns
Fe	ns	ns	Ns	0.43	ns	ns
Co	0.63	ns	Ns	0.27	ns	0.28
Ni	ns	ns	Ns	ns	0.28	ns
Cu	ns	ns	Ns	ns	ns	ns
Zn	0.51	0.6	0.51	0.64	ns	ns
As	nm	nm	Nm	ns	ns	ns
Se	nm	nm	Nm	0.46	ns	ns
Rb	ns	nm	Nm	nm	nm	nm
Mo	0.93	0.66	0.74	0.83	0.59	0.56
Cd	0.73	ns	Ns	0.54	ns	ns

nm- not measured ns- non-significant.

HR to HL- Hydroponic Root to Hydroponic Leaf, HL to SL1- Hydroponic Leaf to Soil Leaf, HL to SL2- Hydroponic Leaf to Soil Leaf 2, SL1 to SL2- Soil Leaf 1 to Soil Leaf 2, SL1 to SS- Soil Leaf 1 to Soil Seed, SL2 to SS- Soil Leaf 2 to Soil Seed.

While there is significant variation associated with genotypes for all elements (See histograms in Figure S1), the range of that variation as measured by the ratio of the mean of the highest to mean of the lowest accession (Tables 1, 2, 3) or by the coefficient of variation (standard deviation accession means/mean of accession means, C.V., Figure 1) is highly element dependent. The macronutrients (Mg, P, S, K, Ca) and Fe all vary within a ∼2 fold range with a C.V. of <20%, while Na and the micronutrient Mo have C.V.s. higher than 50% and can vary by an order of magnitude in some of the experiments. The C.V.s measured here are similar to the C.V.s measured for the corresponding elements in three RIL populations grown and analyzed using the same ionomic methods [24]. For many of the elements the range of accumulation was similar between the tissues and experiments. In the soil experiments, the seeds tended to have higher ionomic variability than the leaves, especially for some of the low abundance elements. In the hydroponics experiments, with the exception of S, K and Mo, the leaves were more variable than the roots.

**Figure 2:**
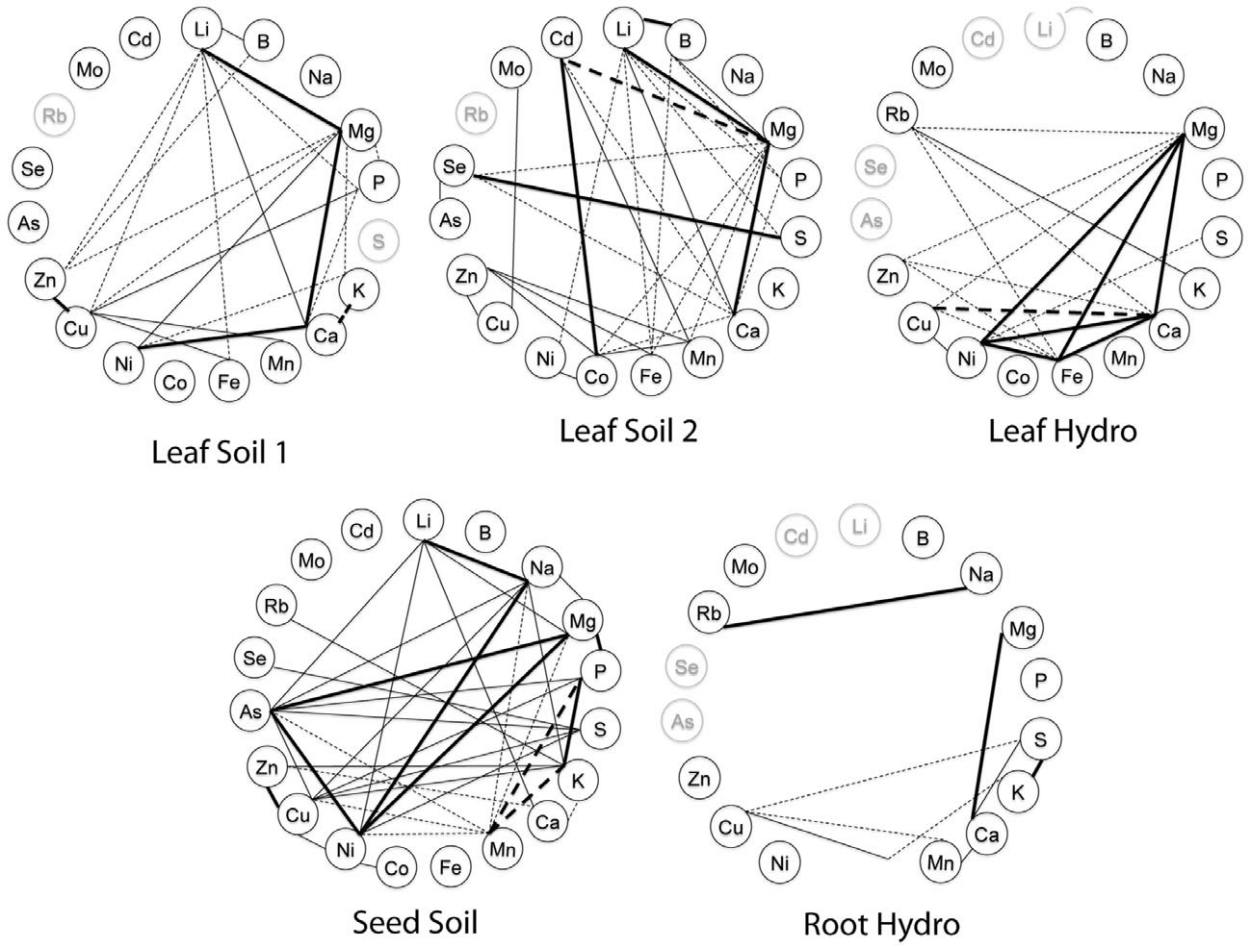
Correlations wheels showing significant correlations between elements within an experiment. Correlations were calculated from line averages and only significant correlations (p<0.001 and R^2^>0.32) are displayed on each wheel. Positive correlations are denoted by solid lines, negative correlations are denoted by dashed lines. Thick lines indicate R^2^>0.5, thin lines indicate 0.32<R^2^<0.5. Ions not measured in a given experiment are colored in grey.

**Figure 3:**
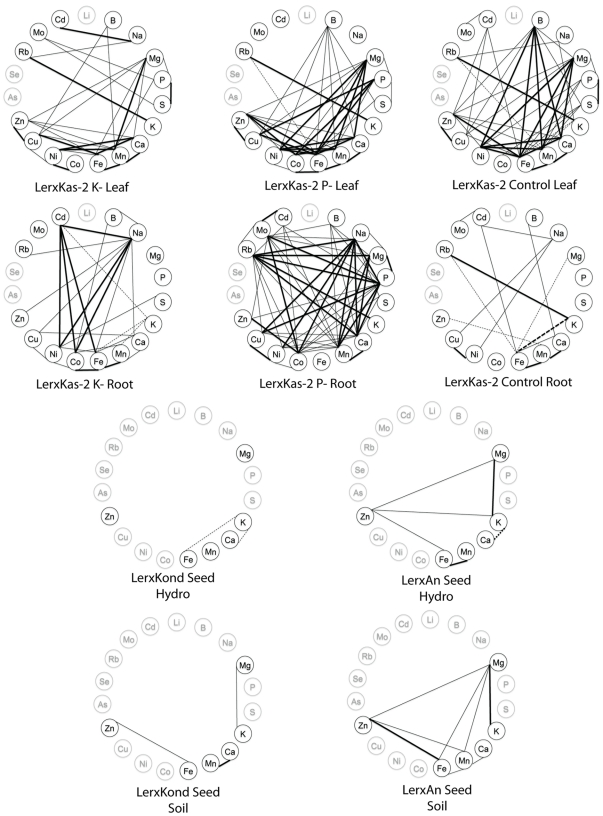
Correlations wheels showing significant correlations between elements within an experiment. A–F are from Prinzenberg et al. [17], with correlations calculated from the original data. G–J are taken from Ghandilyan 2009, [27], Table 3. Only significant correlations (p<0.001 and R^2^>0.32) are displayed on each wheel. Positive correlations are denoted by solid lines, negative correlations are denoted by dashed lines. Thick lines indicate R^2^>0.5, thin lines indicate 0.32<R^2^<0.5. Ions not measured in a given experiment are colored in grey.

**Figure 4:**
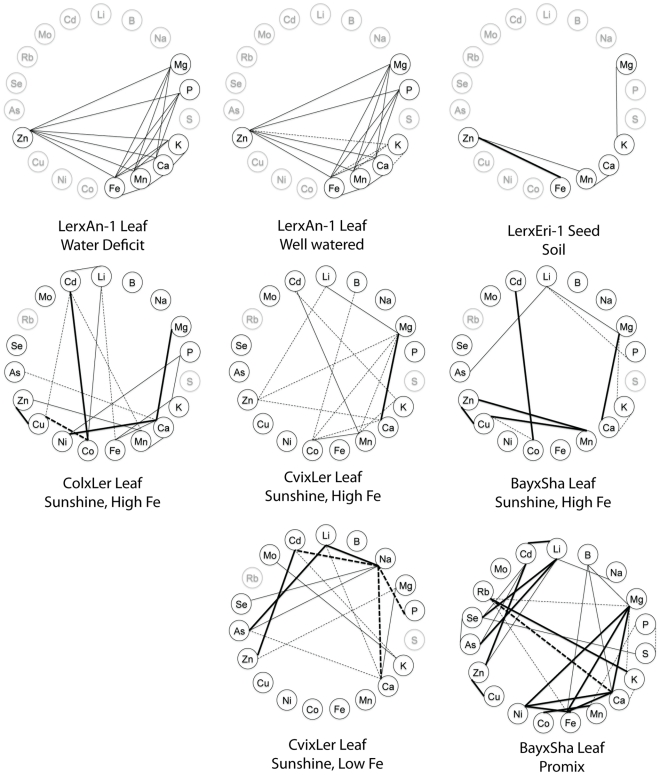
Correlations wheels showing significant correlations between elements within an experiment. A–B are taken from Ghandilyan 2009, [26], Figure 5. C is taken from Ghandilyan 2009, [27], Table 2. D–H taken from Buescher et al. [24] Figure 1. Only significant correlations (p<0.001 and R^2^>0.32) are displayed on each wheel. Positive correlations are denoted by solid lines, negative correlations are denoted by dashed lines. Thick lines indicate R^2^>0.5, thin lines indicate 0.32<R^2^<0.5. Ions not measured in a given experiment are colored in grey.

**Figure 5:**
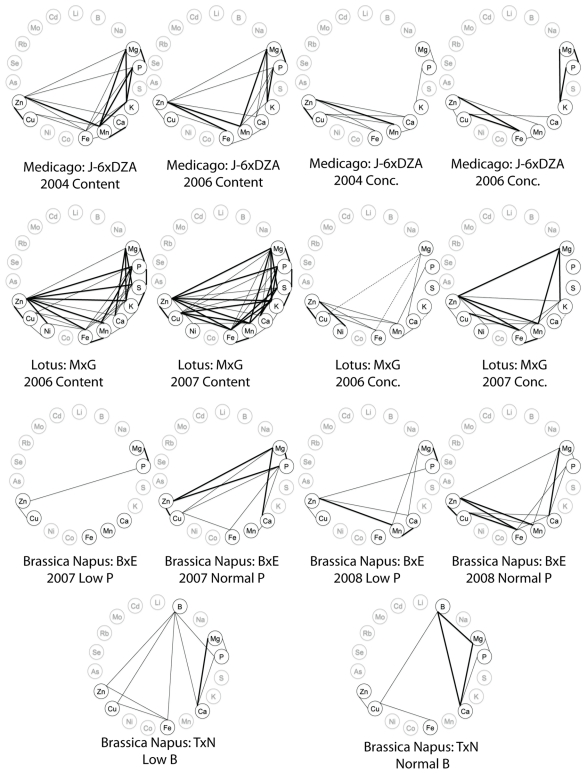
Correlations wheels showing significant correlations between elements within an experiment. A–D from Sankaran et al. [30] Tables 1 & 2 for the *Medicago Truncatula* Jemalong-6×DZA315.16 population. E–H from Klein et al. [28] Table 2. For the *Lotus Japonicus* Miyakojima MG-20×Gifu B-129 population. I–L from Ding et al. [25] Table 2 for the *Brassica Napus* B104-2Eyou Changjia population. Mn from Liu et al. 29 table 2 for the Brassica Napus TapidorNingyou 7 population. Only significant correlations (p<0.001 and R^2^>0.32) are displayed on each wheel. Positive correlations are denoted by solid lines, negative correlations are denoted by dashed lines. Thick lines indicate R^2^>0.5, thin lines indicate 0.32<R^2^<0.5. Ions not measured in a given experiment are colored in grey.

### Between Tissue Elemental Correlations

In order to compare the genetic control of each element between tissues and experiments, we calculated the correlations between the tissues on the same growth medium and all three leaf experiments (Table 4). Of the 97 comparisons, 36 were significantly positively correlated (*p*<0.01) and none were negatively correlated. In the comparison of the two soil leaf experiments, all but three of the elements were significantly correlated, demonstrating that the phenotypes are quite reproducible within a given tissue and fairly similar environments. Mo was significantly correlated in every comparison while Zn was significantly correlated in all comparisons except those including the seeds. Almost all the macronutrients (Na, Mg, P and K) were correlated between the three leaf experiments, but with the exception of P between the roots and leaves in hydroponics, no macronutrients were correlated between the leaves and either roots or seeds.

### Within Tissue Element Correlations

We also analyzed the correlations between elements within each tissue to identify genetically correlated elements (Figure 2). In the leaf datasets, we identified a large number of positively and negatively correlated elements, while in the seeds almost all of the correlations were positive. No elements were correlated with each other in all five datasets, although Mg/Ca a pair of elements that has been found to correlate in many other studies, were correlated in all but the seed dataset and the chemical analogs S/Se were correlated in all experiments where both analogs were measured. Correlations between elements within a tissue appears to be highly variable between species, accessions, tissues and environments as seen in the wheel plots we made based on data from a large number of other ionomics studies (Figures 3,4, and 5) [17], [24]–[30].

**Figure 6:**
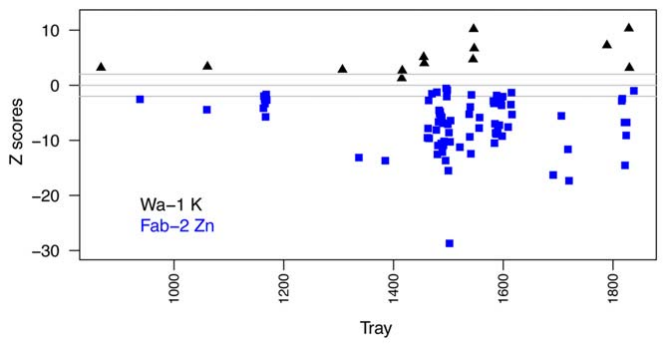
Z-score values of Wa-1 for K and Fab-2 for Zn across the ionomicshub.org database. Z-scores ([Line average – Control Average]/Control S.D.) compared to Col-0 in each tray for all instances where Wa-1 K (black triangle) or Fab-2 Zn (blue squares) were measured. Grey lines denote zscores of −2, 0, and 2.

### Identification of Confirmed Extreme Accessions

The ionomic profiles of the 96 accessions provide a resource for the identification of genes underlying the variation that we observed. To identify potential candidates for accessions accumulating reproducibly high and low levels of each element, we compared the lists of top and bottom five accessions in the three leaf experiments (Table S1). For most elements, we were able to identify accessions that showed up in the same extreme in at least two of the three experiments. To confirm the seed accessions in the seed screen, we compared the accessions from top and bottom five lists that were also in a repeat experiment of 12 accessions and 46% of the possible differences confirmed (Table S2). Several accessions were selected for further study based on extreme leaf ionomic phenotypes (for example: high K in Wa-1 and low Zn in Fab-2 and Van-0) and their phenotypes have repeated over many experiments (Figure 6).

## Discussion

The large dataset described here increases the resources that can be utilized to understand the natural variation of the ionome. Previous efforts to study the variability of the ionome have focused on smaller sets of elements and diverse lines along with inbred populations derived from a few parents [17], [24]–[32]. Our studies used ICP-MS instead of ICP-OES and we added various trace elements to the soil or watering solution in sub-toxic concentrations, allowing us to measure the concentrations of low abundance elements such Li, Co, Ni, Se, As, Rb, Mo, and Cd [33]. Quantifying these additional elements provides a fuller picture of how the ionome is regulated at the genetic, tissue and environmental level.

Our results suggest that the ionome is under tight genetic control, but the different tissues of a plant are independently regulated and there are strong interactions with the environment driving the observed variation. Within each of the experiments, the underlying genetic variation was a significant contributor to the observed phenotypic variation for almost all of the elements. The similar C.V. values between this diverse population and the RIL populations previously studied (Figure 1) suggests that there are strong constraints on the evolution of ionomic traits. The heritabilities for most of the traits were high enough that genetic mapping studies could be undertaken to identify the genes responsible for the phenotypic variation. Indeed, the ionomics approach has been successfully used to clone genes responsible for natural variation in Na, Co, Mo, S and Cu homeostasis in *A. thaliana* [23], [34]–[38].

In a result that was also observed by Ghandilyan et al. [26] when tissue pairs (root/leaf and leaf/seed) were compared, few correlations were found for the accumulation of a given element. This was the case even in the root/shoot comparison in hydroponics, where the samples came from the same plants. The absence of any significant negative correlations was somewhat surprising, as preferential sequestration in the roots or leaves has been posited as a possible mechanism for reducing accumulation of some elements in the leaves or seeds, respectively. The lack of tissue correlations suggests that analyzing leaf ionomic phenotypes is a poor proxy for seed phenotypes. Therefore researchers interested in improving the mineral nutrient and trace element content of seeds or leaf tissue should focus on profiling the tissue of interest.

Significant correlations of a pair of elements across a genetically segregating population is an indication that the two elements are controlled by linked genetic loci. In diversity panels such as the one in this study, linkage decays quickly, leaving only a small number of genes in linkage with each other, making it less likely that a pair of correlated traits are being controlled by two unique but linked loci. Therefore, the correlations we observe are likely due to loci that regulate an uptake, transport, sequestration, or remobilization pathway, a regulatory network, or a physiological process that affects both elements. Previous studies have shown that which individual pairs of elements are correlated in a given experiment is highly population and environment specific [17], [24]–[32]. Figures 3, 4, and 5 display the significant correlations identified in other studies, including root, leaf and seed datasets in *A. thaliana* and other dicotyledonous species, in the same format as the data presented in Figure 2. Comparisons between all the studies and the experiments within them are difficult due to the different growth substrates and analysis methods, however, the only element pair significantly correlated in all the experiments where they were both measured was the chemical analogs S and Se. Another pair of chemical analogs, K and Rb, are correlated in all leaf tissues where both were measured but not in the root hydroponics of our data or in the root hydroponics of Prinzenberg et al. [17] when grown in low K media. Interestingly, even though they are correlated, the ratios of both pairs of elements showed genetic variation. This suggests that there are alleles affecting processes that discriminate, albeit slightly, between the analogs segregating in the population.

There were several pairs of elements that were consistently correlated in a single tissue, but not in other tissues. Mg and P were significantly correlated in the seeds of the 96 accessions, and this correlation occurred in many of the other seed experiments. Ca and Mg were significantly correlated in every leaf experiment but only a subset of the root and seed tissues. The correlation between Ca and Mg appears to be quite robust, as it has been noted in several other species as well, even though there are clearly different cellular pathways for the two elements and there relationship is broken in the *esb1* mutant [18]. The reduced correlation in the roots and seeds may be due to the lower phloem mobility of Ca when compared to Mg [1], [39]–[42]. Even correlations that appear in a single experiment are likely to be biologically relevant. For example, the Cd-Mg anti correlation observed in the second soil leaf was confirmed by Hermans et al., who demonstrated that low Mg status has a protective effect during Cd exposure [43].

It is important not to over-interpret the lack of observed correlations as evidence that no common genetic mechanisms exist between tissues or elements as several factors complicate the analysis. 1) Unlike recombinant inbred populations where there are only two alleles, present at a frequency ∼0.5, at any loci, the populations in this study may have many different alleles at each locus. An uncommon variant could significantly affect multiple tissue or elements, but have a low enough frequency that the effect will not make a significant contribution to the correlation among 96 accessions. 2) There is ample evidence that the seed ionome is composed of elements that traffic directly from the root as well as those remobilized from the leaves, making perfect correlations between the leaf and seed ionome unlikely [44]. 3) Experimental design factors may limit our ability to detect correlations, for example, iron-phosphate plaques accumulating on the roots in hydroponics may obscure the signal of internal Fe and P.

The lack of correlation observed between tissues suggests that researchers interested in an ionomic trait in a given tissue should look for data on elemental accumulation in that tissue as the primary method for selecting lines for further genetic studies. There are two important caveats to this conclusion. The first is that the extremes of the seed ionome appear to be less reproducible than leaves, although the confirmation of seed phenotypes experiment we did was limited to 12 accessions (Table S1). The second caveat is that this conclusion only appears to be valid if the ionome itself is of interest in a given tissue. There is ample evidence that profiling the leaves is a good way to interrogate root processes, if not the root ionome. There are now several examples of mutations that affect root processes that alter the leaf ionome [18]–[20], [34], [38], [45]. Given the difficulty of precisely quantifying the root ionome of plants grown in soil due to contamination of the surface of the root with soil derived material, the leaf ionome is probably the tissue of choice for investigating root processes involved in regulating the ionome in soil grown *A. thaliana* plants.

The population studied here was originally designed for association mapping [22], however, it was later found to be inadequate, mainly due to the low number of accessions. Accordingly, when we performed association analysis on the Soil Leaf 1 dataset, only a few SNPs were found to exceed the genome wide permutation thresholds [21]. This does not mean that there are not true positive associations to be found by applying these methods to the datasets in this manuscript, just that additional bioinformatic and experimental approaches will be necessary to identify promising candidates. These datasets are useful for identifying extreme accumulators to be used for the development of experimental F2 populations for conventional linkage-based mapping approaches such as bulk segregant analysis [46] and these efforts are ongoing in the authors laboratories [47]. The genomic regions identified through these approaches can be used to prioritize candidates identified in the association mapping analysis. HKT1;1, FPN2 and MOT1 have previously been identified as the genes underlying Na, Co and Mo QTLs in *A. thaliana* [23], [34], [37], [38]. These three loci are clearly affecting the phenotypic distributions of Na, Mo, and Co observed in this study.

The populations contained several pairs of accessions that were collected within a few kilometers of each other. Several of these pairs exhibit strong differences in ionomic phenotypes. For example, the low Zn in Fab-2 and the high Na in Ts-1 are not found in the nearby accessions Fab-4 and Ts-5 respectively. Allelic variation at loci controlling the ionome is therefore likely to be segregating in these populations, suggests that ionomic phenotypes may be reflecting very local adaptations to the environment.

### Conclusion

We have analyzed the elemental content of roots, leaves and seeds from a diverse collection of *A. thaliana* accessions. While genetically-based variation exists for all elements we measured in the root, leaf and seed ionomes, the patterns of accumulation are not consistently correlated between elements within a tissue nor between tissues for a given element. These results suggest that the ionome of a plant tissue is highly plastic, yet tightly controlled by genes and gene×environment interactions. The dataset provides a valuable resource for mapping studies to identify genes regulating elemental accumulation. All of the ionomic data presented in the study is available at www.ionomicshub.org.

## Materials and Methods

### Plant Growth

#### Soil Leaves

A. thaliana plants for ICP-MS analysis were grown in a highly controlled environment that have been described before [33]. Briefly, seeds were germinated on a 20-row tray with moist soil Sunshine mix LB2 (Sun Gro Horticulture, screened through a 1/4 inch mesh) after stratified at 4°C for 3 days. The plants were then grown in the growth room of Purdue Ionomics center with 8 h light (90 µmol·m^−2^s^−1^)/16 h dark and 19 to 22°C temperature. During following days, plants were bottom-watered twice a week with modified 0.25×Hoagland solution [18]. The biggest one or two leaves were harvested from 5-weeks plants for elemental analysis.

#### Soil Seeds

Plants were grown in 72 pot trays with a single plant per pot. Four control lines: Col-0(n = 6), Kas-1 (n = 6), Ler-2(n = 5), and Cvi-0 (n = 5), were grown in each tray with eight test lines (n = 6) and two pots removed to provide watering access. All trays were planted (2–3 seeds/pot) then stratified for 3 days at 4C before being transferred to a growth room under 8 h light for 7 days. Trays were then transferred to a lighted (8 h days) 4C cooler for 8 weeks during which the pots were weeded to leave only one plant. After 8 weeks, the plants were transferred to a long day growth room and grown until the plants dried up. At that point, all of the available seed was harvested and cleaned for ICP-MS analysis.

### Hydroponics

Seeds were germinated in soil and two-weeks-old plantlets were transferred to hydroponic systems. Roots of plantlets were rinsed in distilled water and immediately placed on tiles covering the containers (capacity of 4.5 l) filled with mineral solution (Hermans et al., 2010b). The macronutrients concentrations in mM were 1.00 Ca(NO_3_)_2_, 1.00 MgSO_4_, 0.88 K_2_SO_4_, 0.25 KH_2_PO_4_, and micronutrients concentrations in µM were 20 FeEDTA, 10 NaCl, 10 H_3_BO_3_, 1.00 ZnSO_4_, 1.00 MnSO_4_, 0.10 CuSO_4_, 0.01 (NH_4_)_6_Mo_7_O_24_. The pH of the solution was adjusted to 5.8±0.1 with KOH 1 M. Nutrient solutions were replaced every 4 days. Plants were grown with 8 h light (80 µmole photon m^−2^ s^−1^)/16 h dark, 20°C temperature and 70% relative humidity in the SGC-110 Vötsch growth chamber (Weiss Technik, Belgium). The 5 most recently expanded leaves were harvested from five-weeks-old plants for elemental analysis.

### Tissue Elemental Analysis

Tissue samples were dried at 92°C for 20 h in Pyrex tubes (16×100 mm) to yield approximately 2–4 mg of tissue for elemental analysis. After cooling, seven of approximately 100 samples from each sample set were weighed. All samples were digested with 0.7 ml of concentrated nitric acid (OmniTrace; VWR Scientific Products; http://www.vwr.com), and diluted to 6.0 ml with 18 MΩ water. Elemental analysis was performed with an ICP-MS (Elan DRCe; PerkinElmer, http://www.perkinelmer.com) for Li, B, Na, Mg, P, S,K, Ca, Mn, Fe, Co, Ni, Cu, Zn, As, Se, Rb, Mo, and Cd. A liquid reference material composed of pooled samples from A. thaliana leaves was run every 9^th^ sample to correct for run to run variation and within-run drift for all datasets except Soil Leaf 1. All samples were normalized to calculated weights, as determined with an iterative algorithm using the best-measured elements, the weights of the seven weighed samples, and the solution concentrations, implemented in the ionomicshub.org database (for a full description, see http://www.ionomicshub.org/piims/files/WeightCalculation_description_examples.zip, [48]). Data for all elements is available at the ionomicshub.org database either as a single zip file (at www.ionomicshub.org/dataexchange?category=A. thaliana) or as individual trays (Soil Leaf 1 = Trays:861-872,908,938,1010,1011, Soil Leaf 2 = 1163–1169, Soil Seed = 1170–1181, seed confirmation experiment = 1342–1345).

### Data Normalization

Measurements below zero were removed before removing extreme outliers (those values that were greater than the 90^th^ percentile +2×(90^th^–10^th^ percentile) within each tray. To account for variation in the growth environment in the soil experiments, two (Col-0 and Cvi-0 in the soil leaf 1) three (Col-0, Kas-1 and Cvi-0 in the soil seed screen) or four (Col-0, Cvi-0, Fab-2 and Ts-1 in the soil leaf 2 screen) control lines were used to create a tray specific normalization factor. Briefly, for each element, each line in a given tray was compared to the overall average for that line across all trays to obtain an elementxlinextray specific normalization factor. The elementxlinextray factors in a given tray were then averaged to create a trayxelement normalization factor for the tray. Every value for the element in the tray was then multiplied by the normalization factor. Plots of the control lines before and after the normalization are shown in Figure S2, S3, S4, S5, S6, S7.

We then tested for significant genotypic contributions to the variance using the linear model Element∼Tray+Genotype and the *lm* and *anova* functions from R v2.9.1.

### Correlation analysis

All comparisons were based on line averages. For each pairwise combination of elements in the experiment, Pearson correlation coefficients were found using the line average data for pairwise complete observations utilizing the corr function in R. Statistically significant correlations were identified using the t-distribution with n−2 degrees of freedom (where n = 96 for experiments where all lines grew) where t = (corr*sqrt(n−2))/(sqrt(1−corr^2^)), or equivalently using the F-distribution with 1 and n−2 degrees of freedom where F = (corr^2^*(n−2))/(1−corr^2^). A conservative p value cutoff of 0.001 was used.

## Supporting Information

Table S1 **Lists of the five highest and lowest accumulating accessions in each experiment for each element, with the average concentration (PPM) of the accession in that experiment.** Confirmed accessions are indicated in bold. For leaves, confirmed accessions are those that are in the highest/lowest five accessions in at least two of the three experiments. Accessions appearing on the lists in all three experiments are highlighted in grey. For seeds, confirmed accessions are those that were either high or low in the seed confirmation experiment (Table S2). Lines that were in the seed confirmation experiment that didn’t confirm are noted with italics.
(XLSX)

Table S2 **Weight normalized data from the seed confirmation experiment (Trays 1342-1345 at ionomicshub.org).**
(CSV)

Figure S1 **Histograms of line averages of weight normalized PPM data from each experiment for each element.**
(PDF)

Figure S2 **Plot of control line averages for each tray before normalization for Soil Leaf 1 experiment.** All non-control lines are averaged into the “Other” line.
(PDF)

Figure S3 **Plot of control line averages for each tray after normalization for Soil Leaf 1 experiment.** All non-control lines are averaged into the “Other” line.
(PDF)

Figure S4 **Plot of control line averages for each tray before normalization for Soil Leaf 2 experiment.** All non-control lines are averaged into the “Other” line.
(PDF)

Figure S5 **Plot of control line averages for each tray after normalization for Soil Leaf 2 experiment.** All non-control lines are averaged into the “Other” line.
(PDF)

Figure S6 **Plot of control line averages for each tray before normalization for Seed experiment.** All non-control lines are averaged into the “Other” line.
(PDF)

Figure S7 **Plot of control line averages for each tray after normalization for Seed experiment.** All non-control lines are averaged into the “Other” line.
(PDF)
