# Supplementary figures and images for: Biodiversity of Mineral Nutrient and Trace Element Accumulation in Arabidopsis thaliana
Source: PLoS One. 2012 Apr 27;7(4):e35121. doi: 10.1371/journal.pone.0035121 (PMC3338729; doi:10.1371/journal.pone.0035121)

# Li7

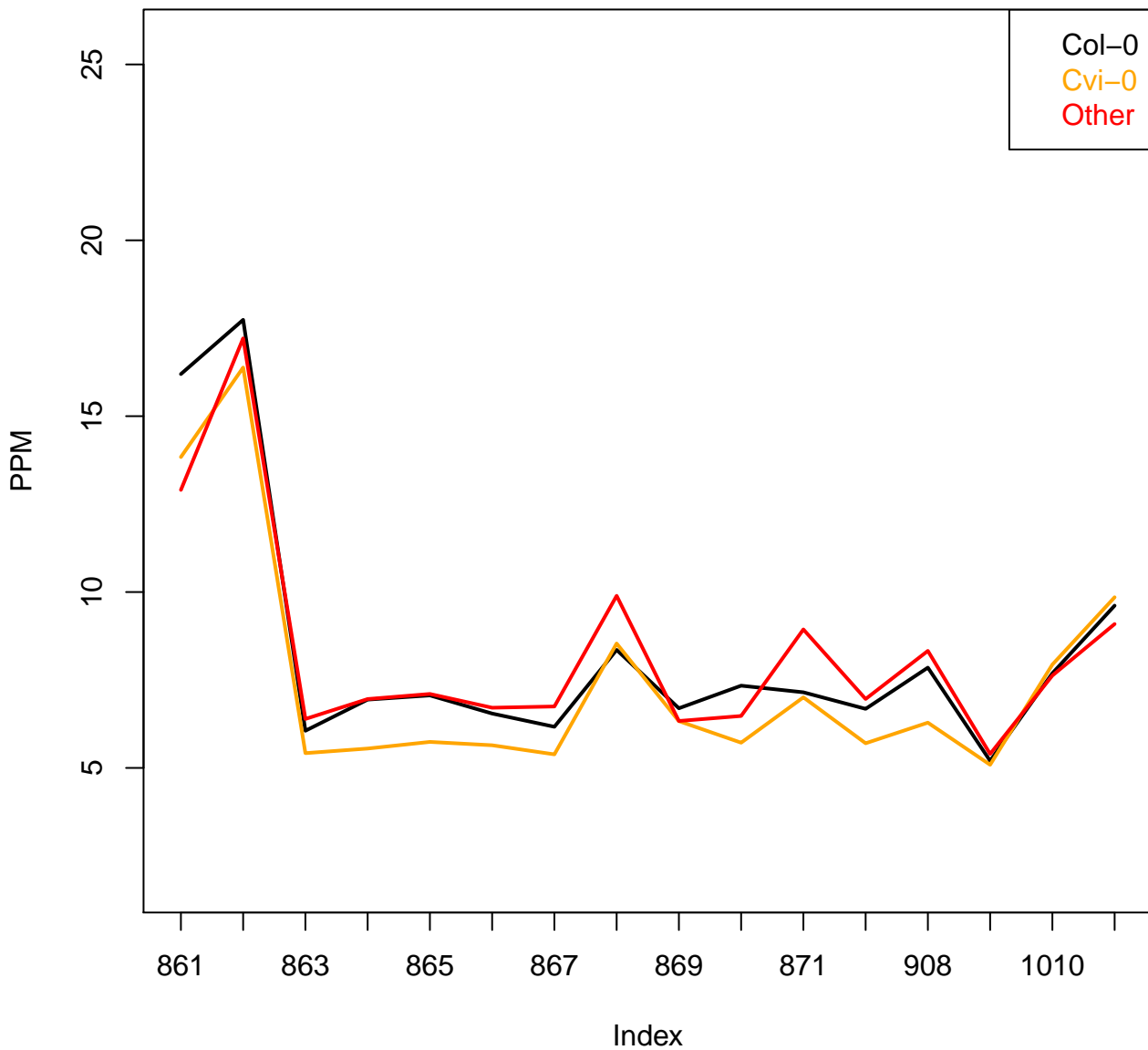

# B11

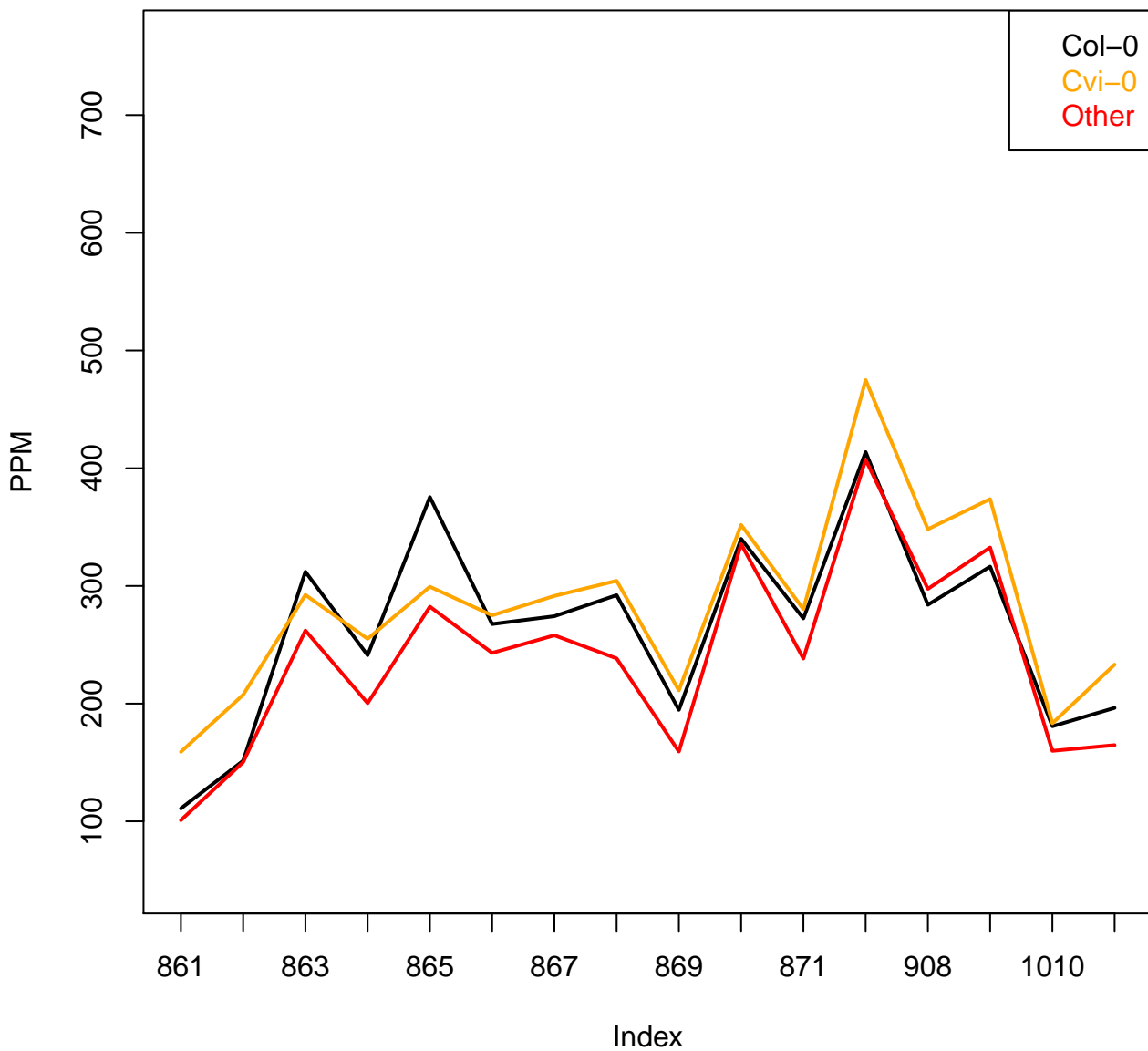

# Na23

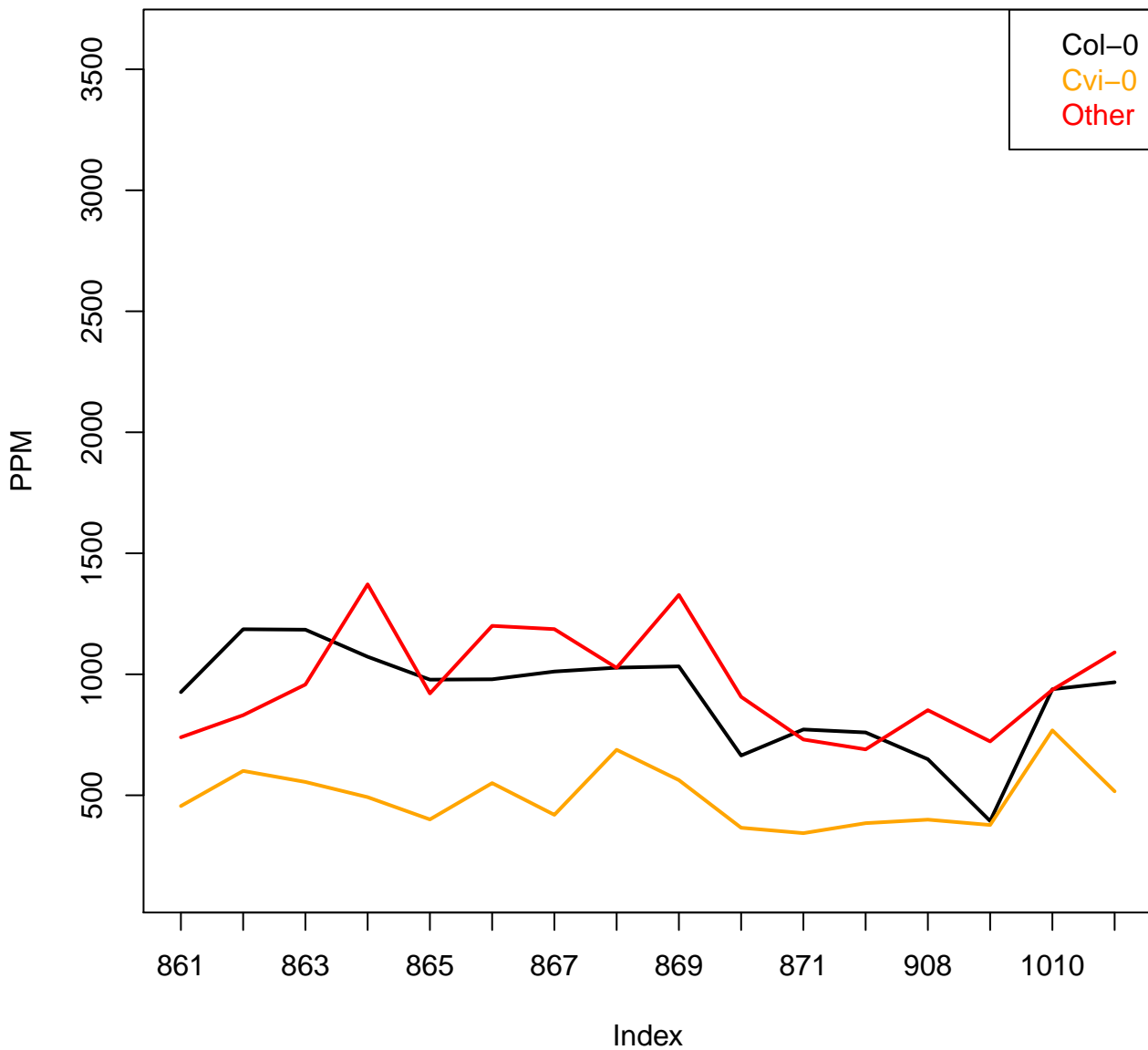

# Mg25

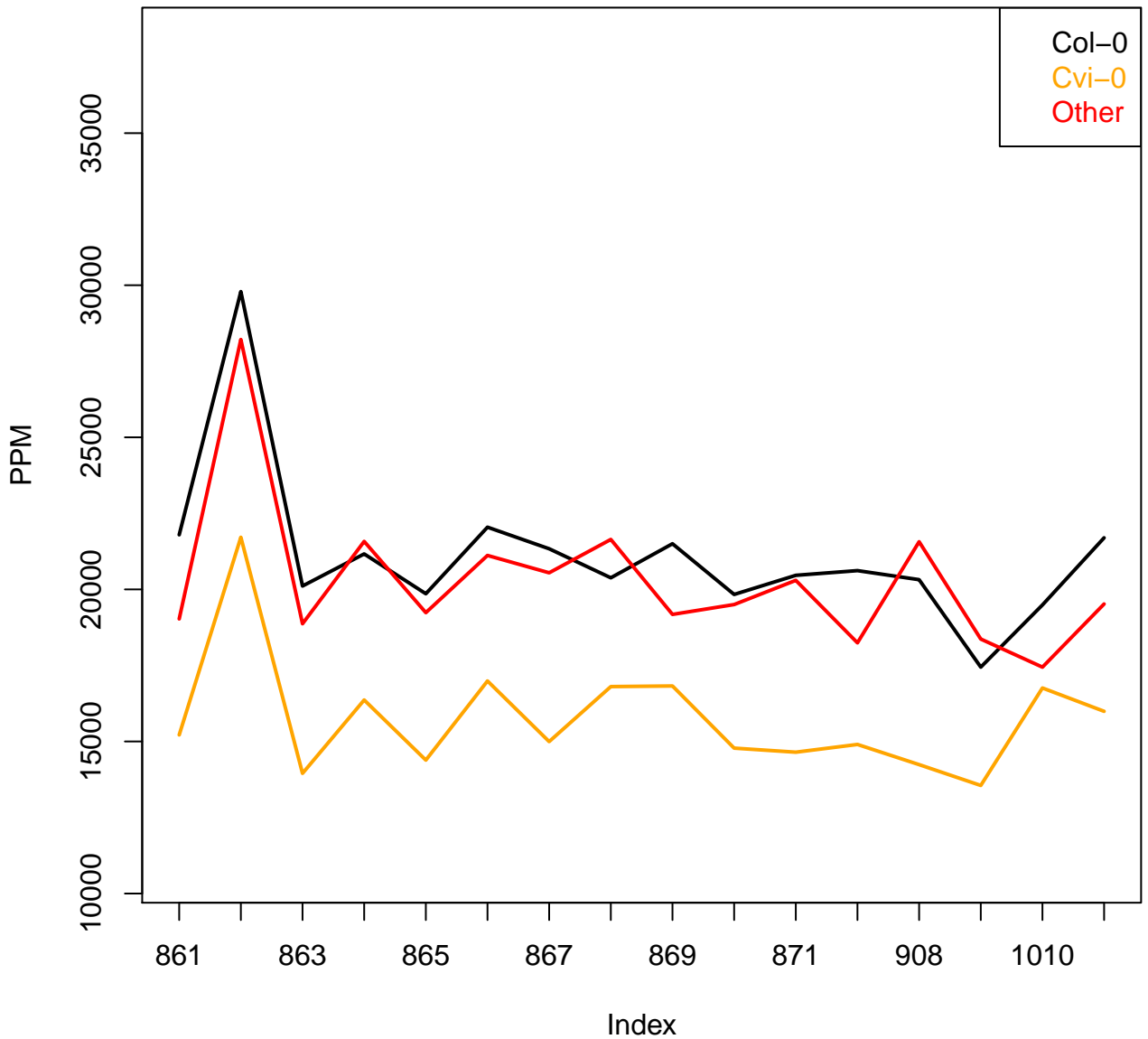

# P31

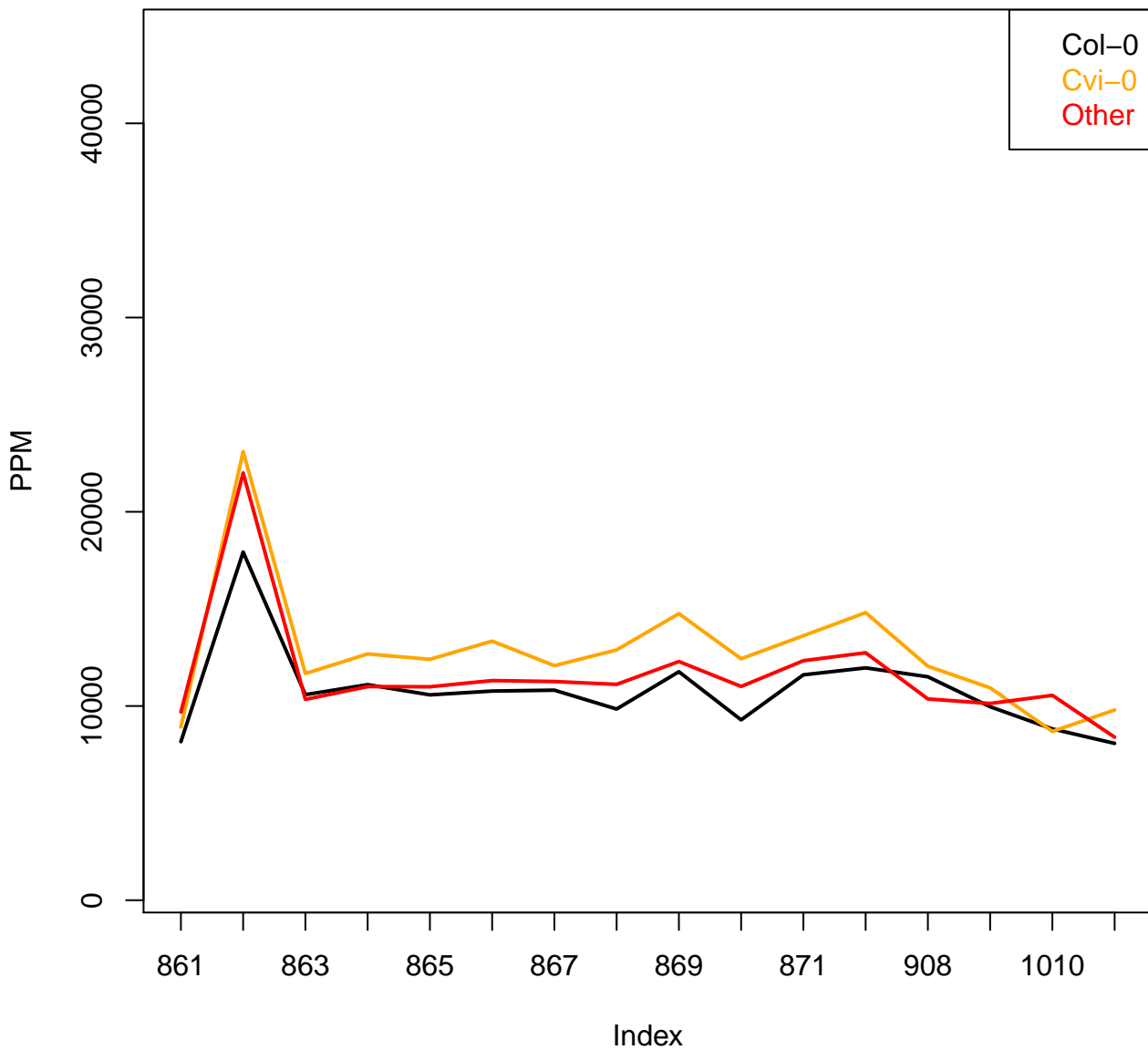

# K39

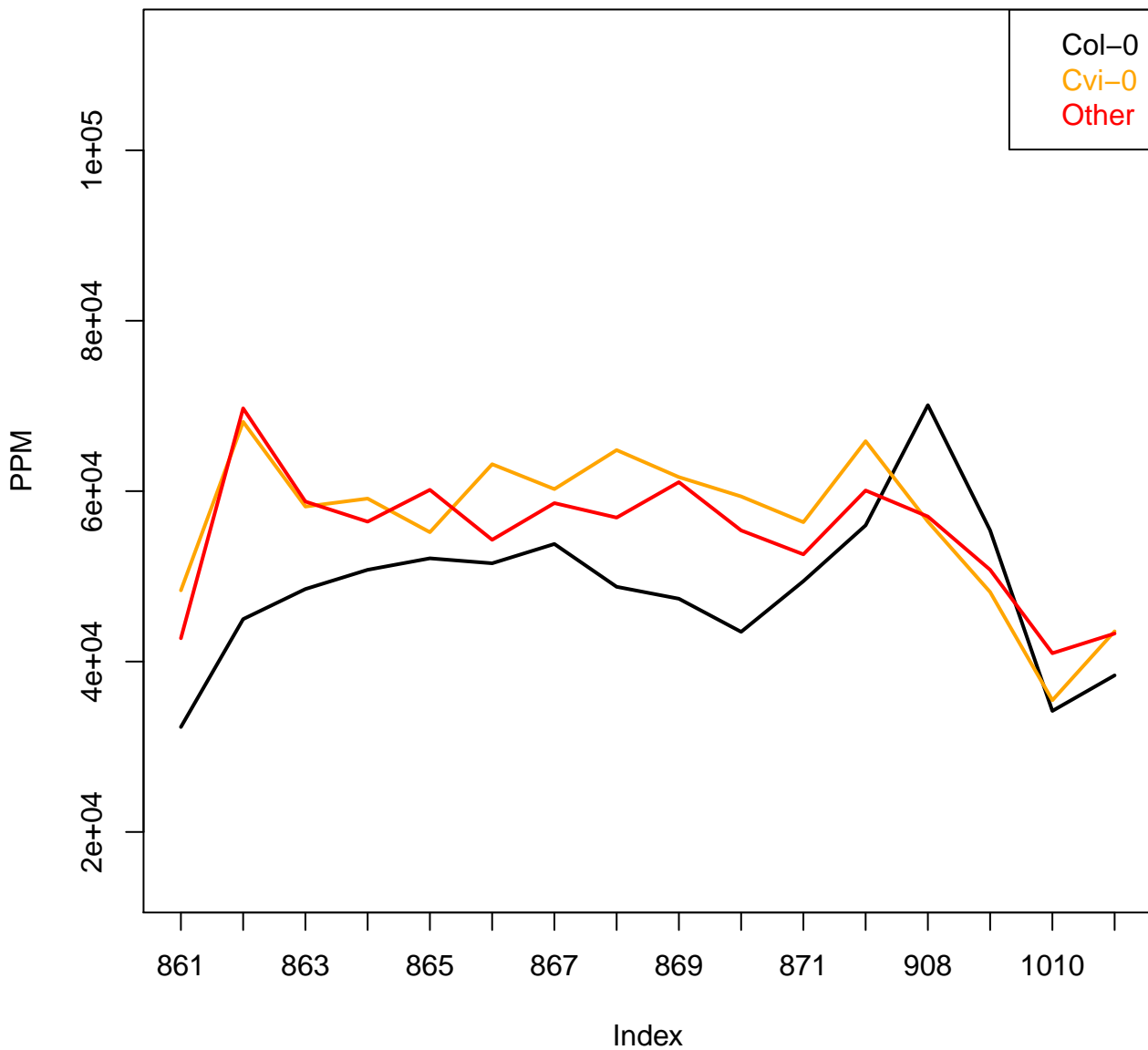

# Ca43

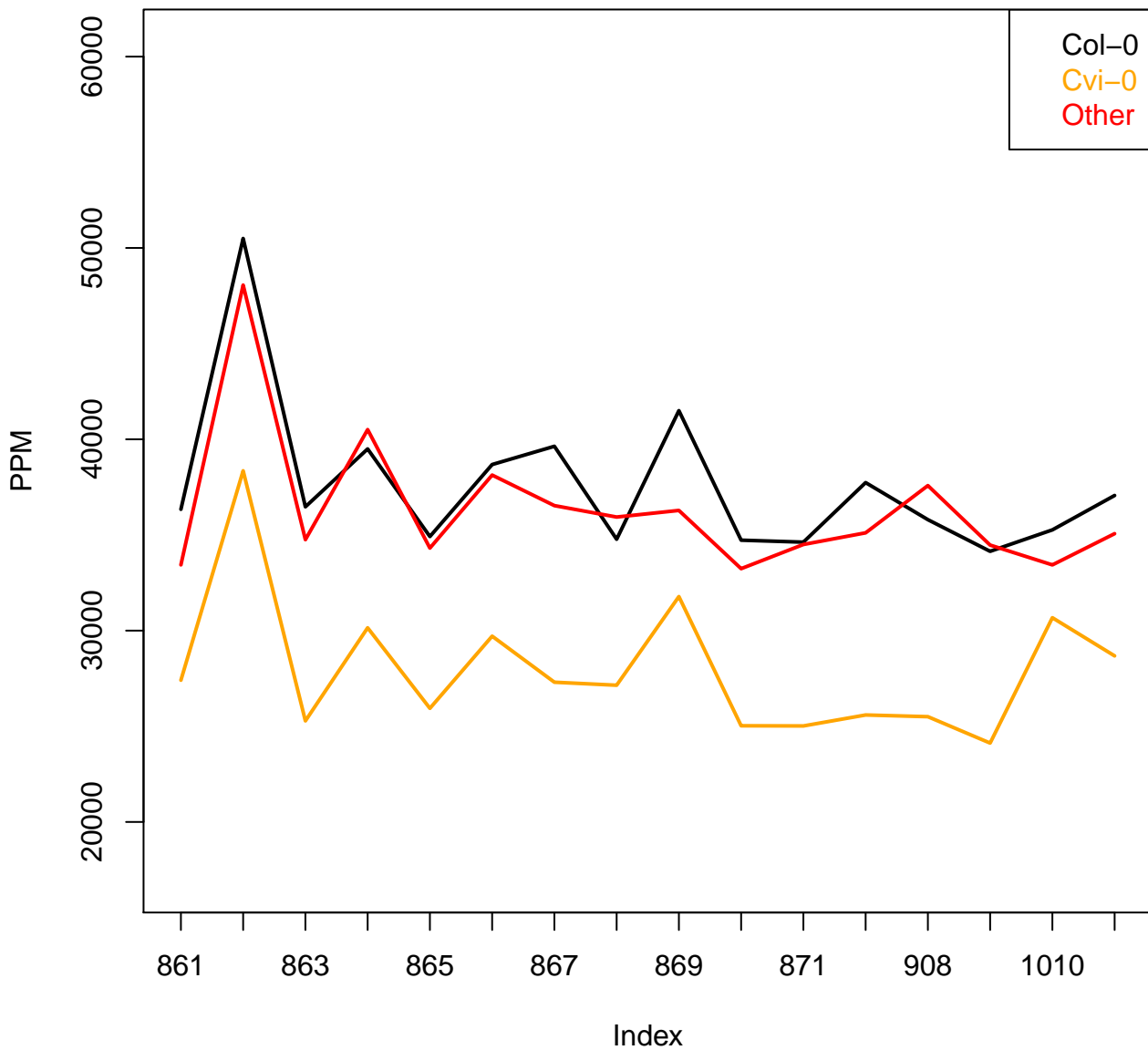

# Mn55

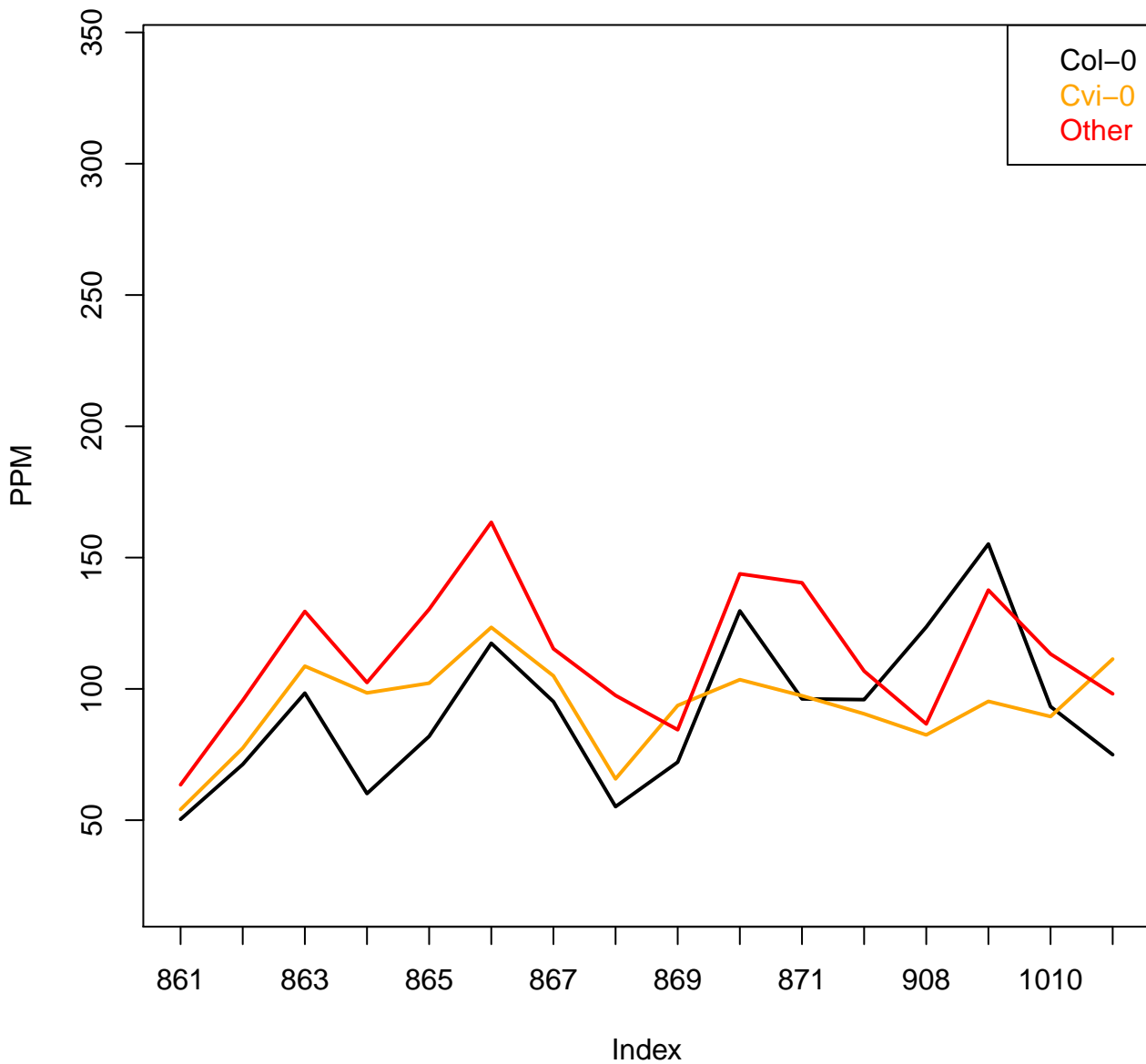

# Fe56

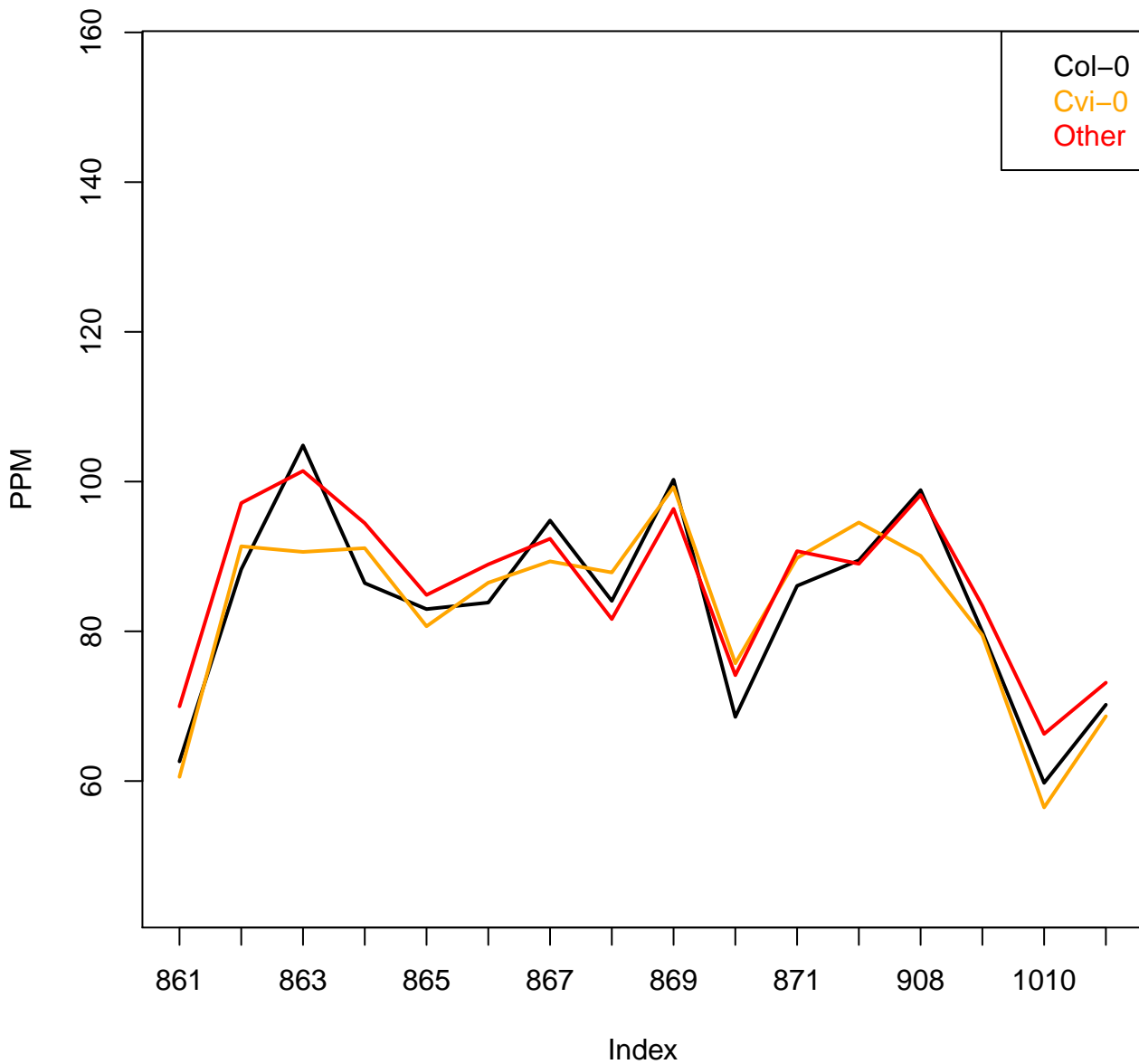

# Co59

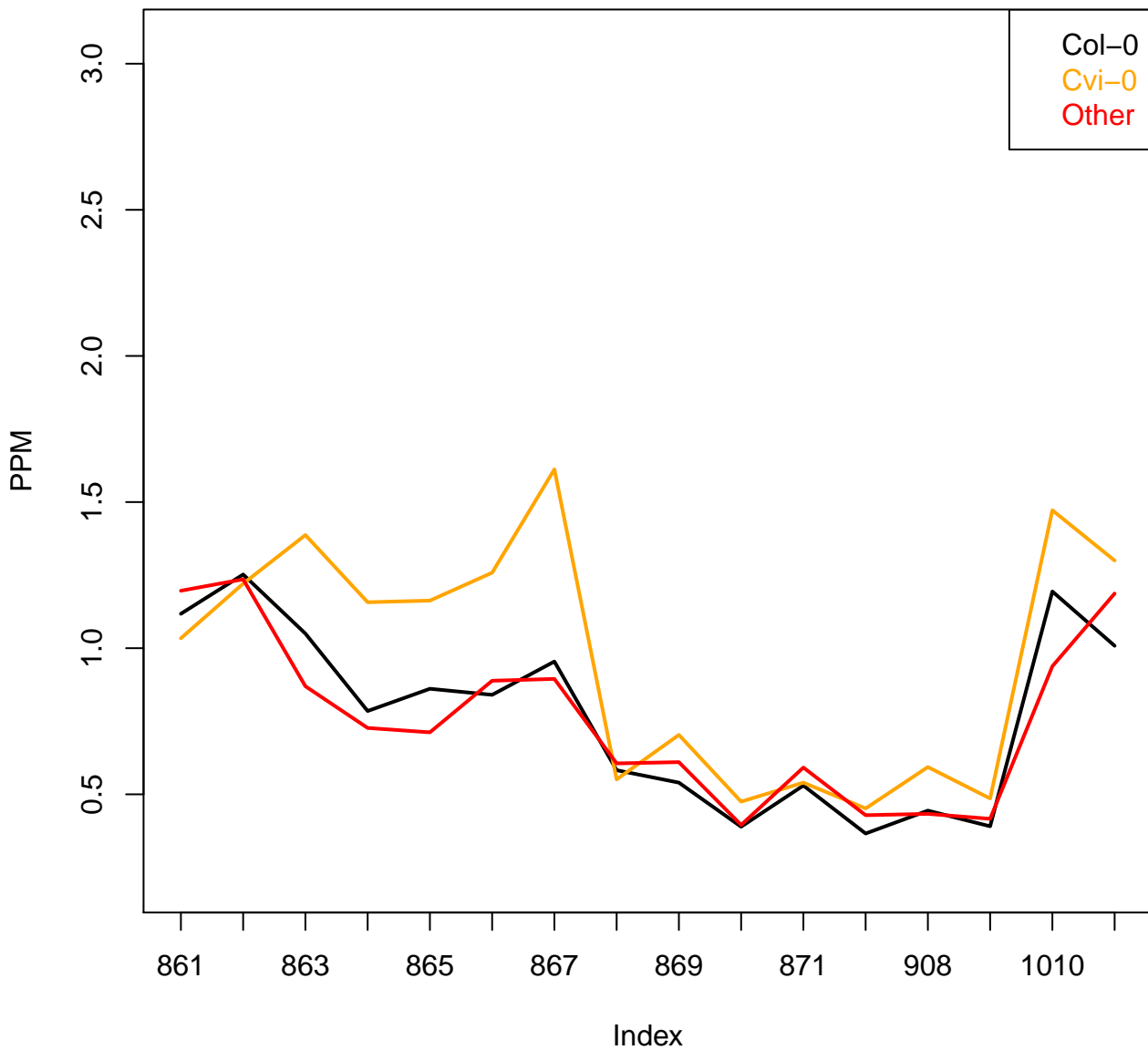

# Ni60

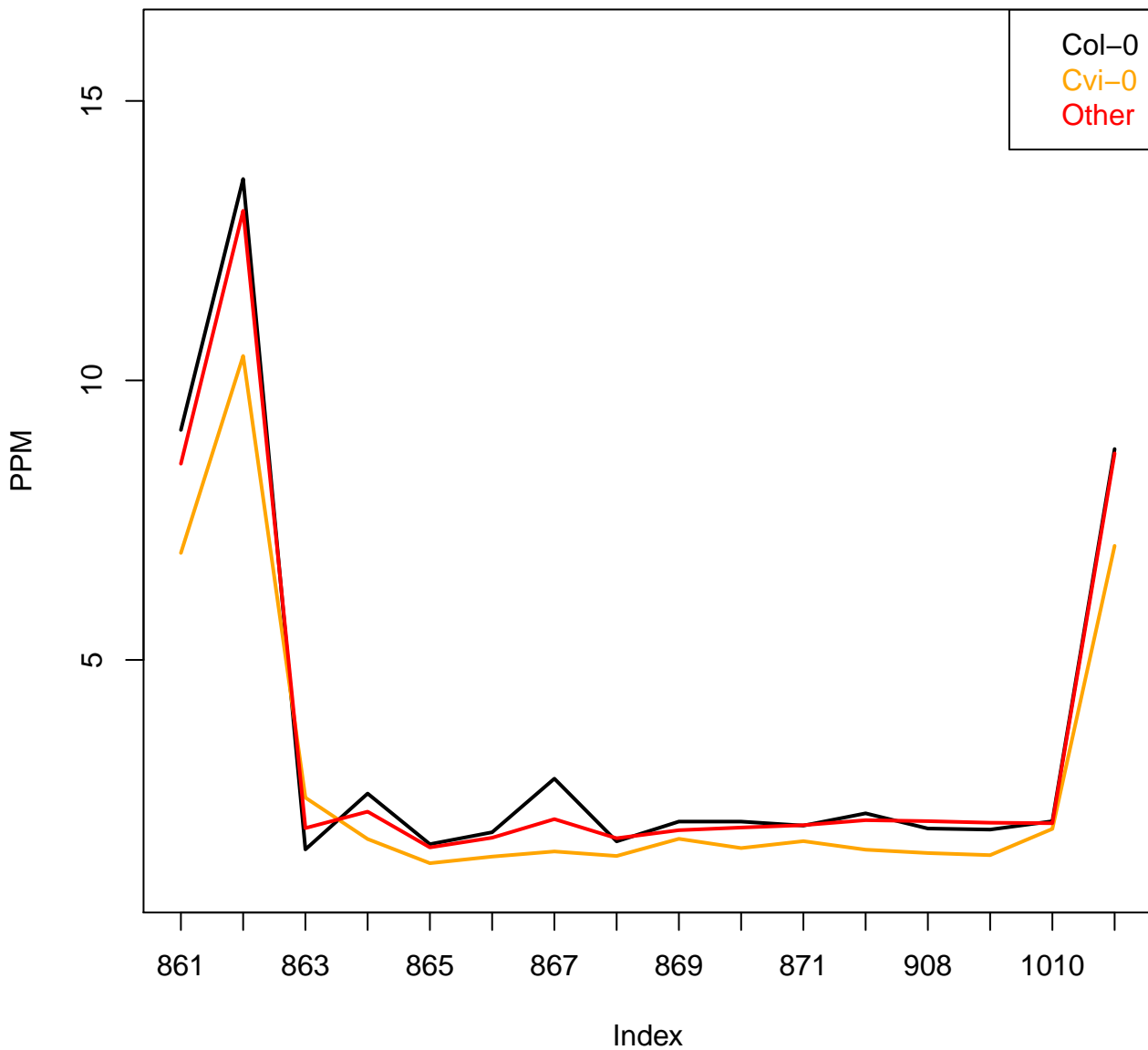

# Cu65

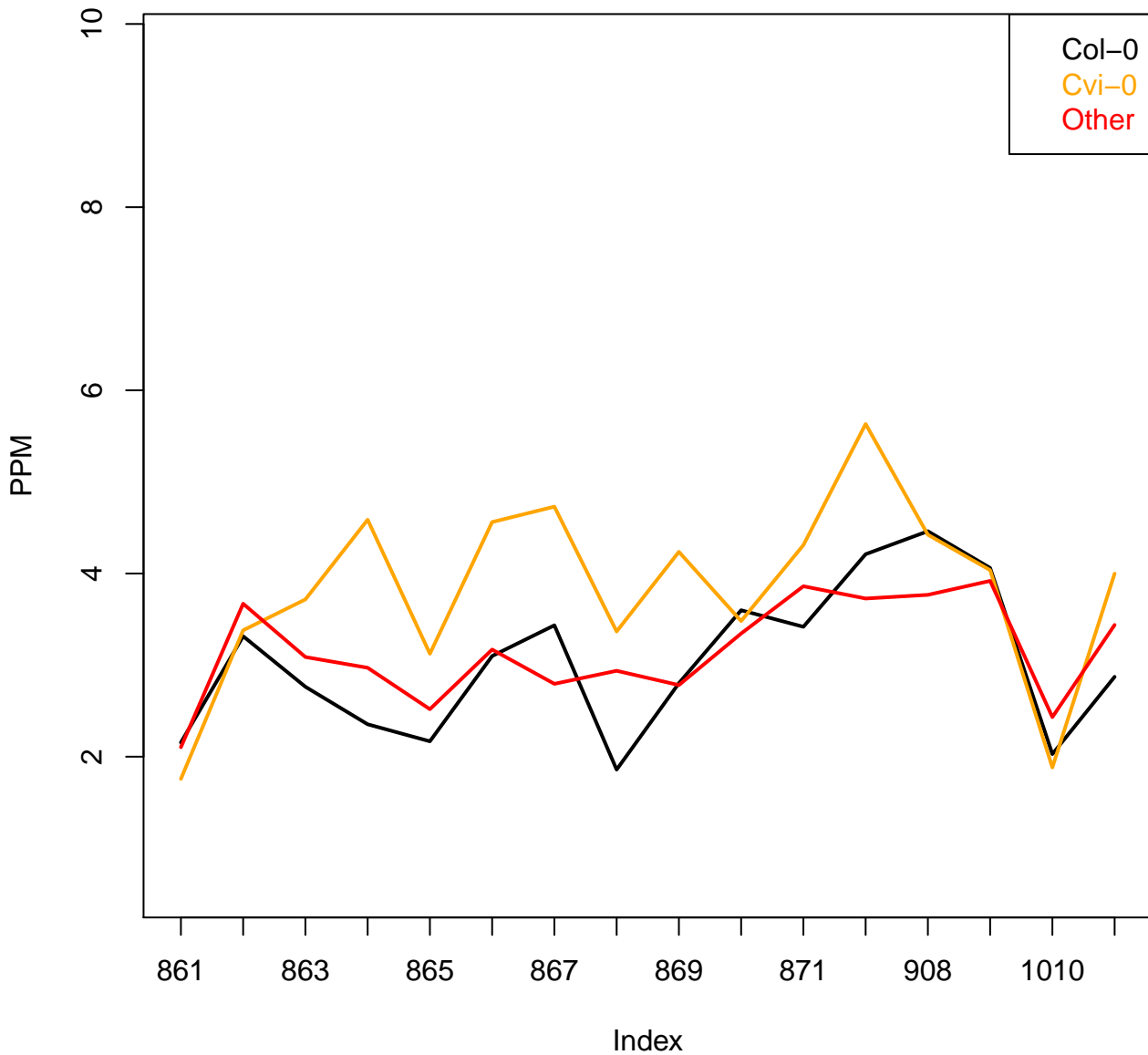

# Zn66

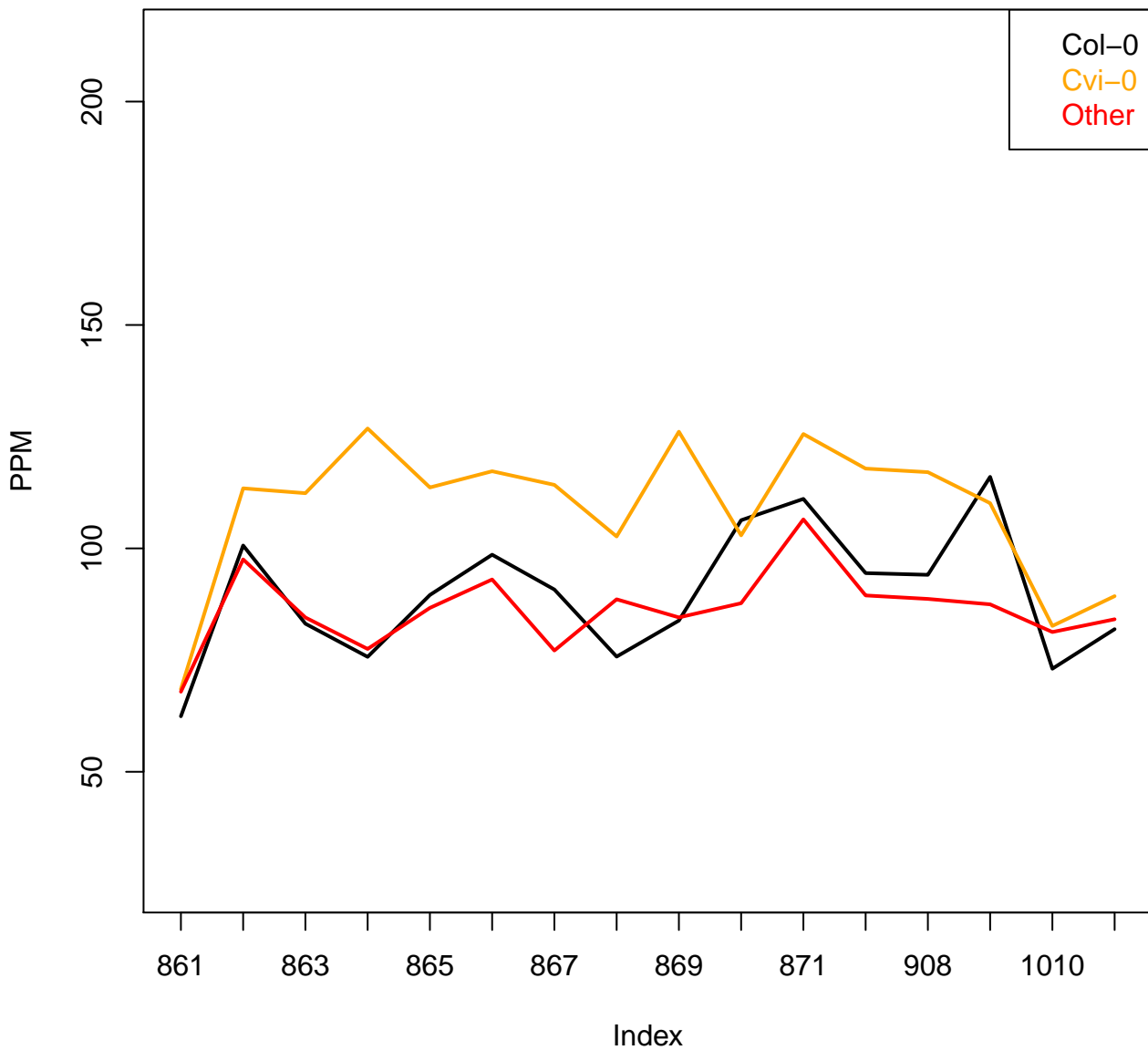

# As75

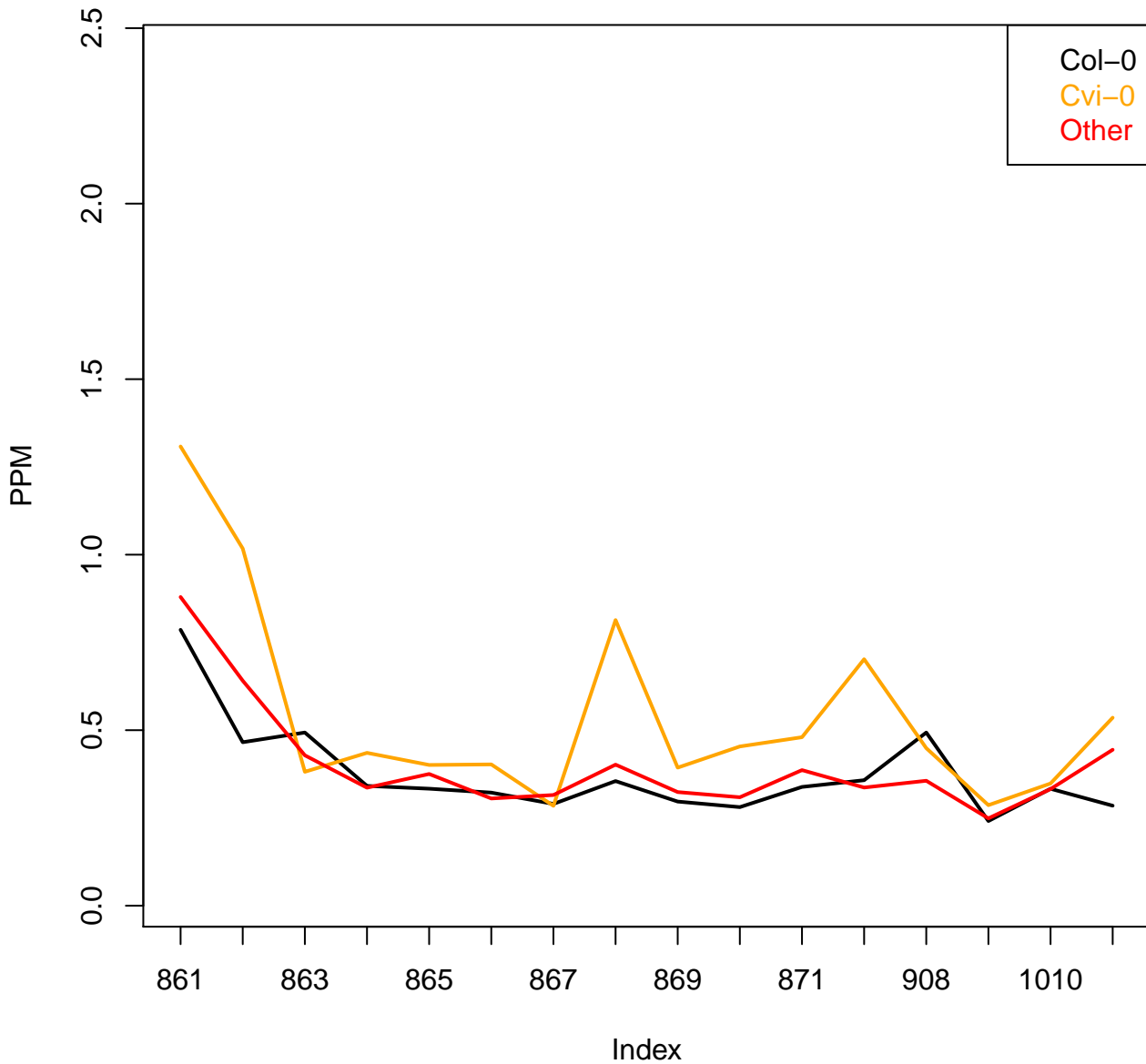

# Se77

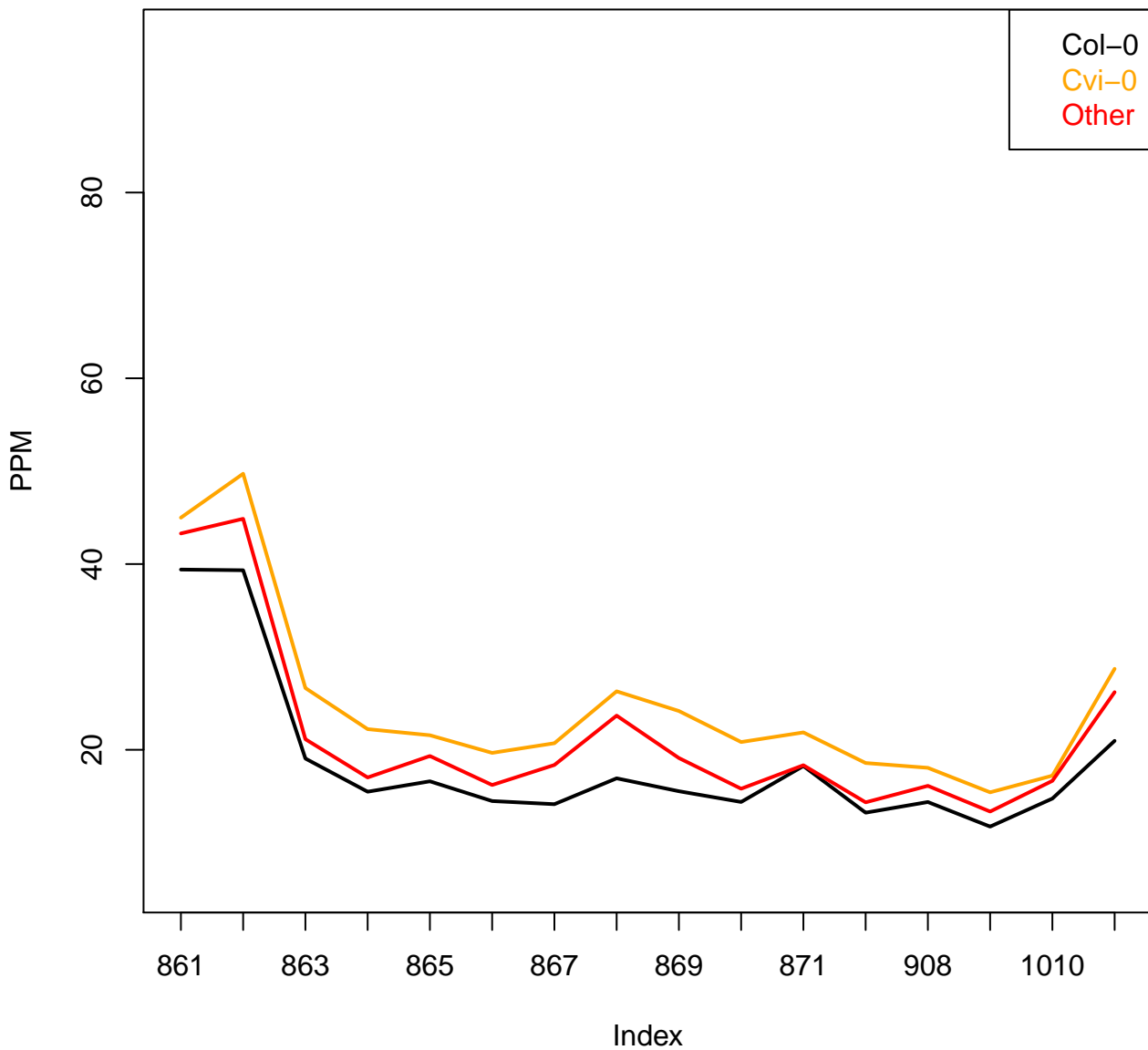

# Mo95

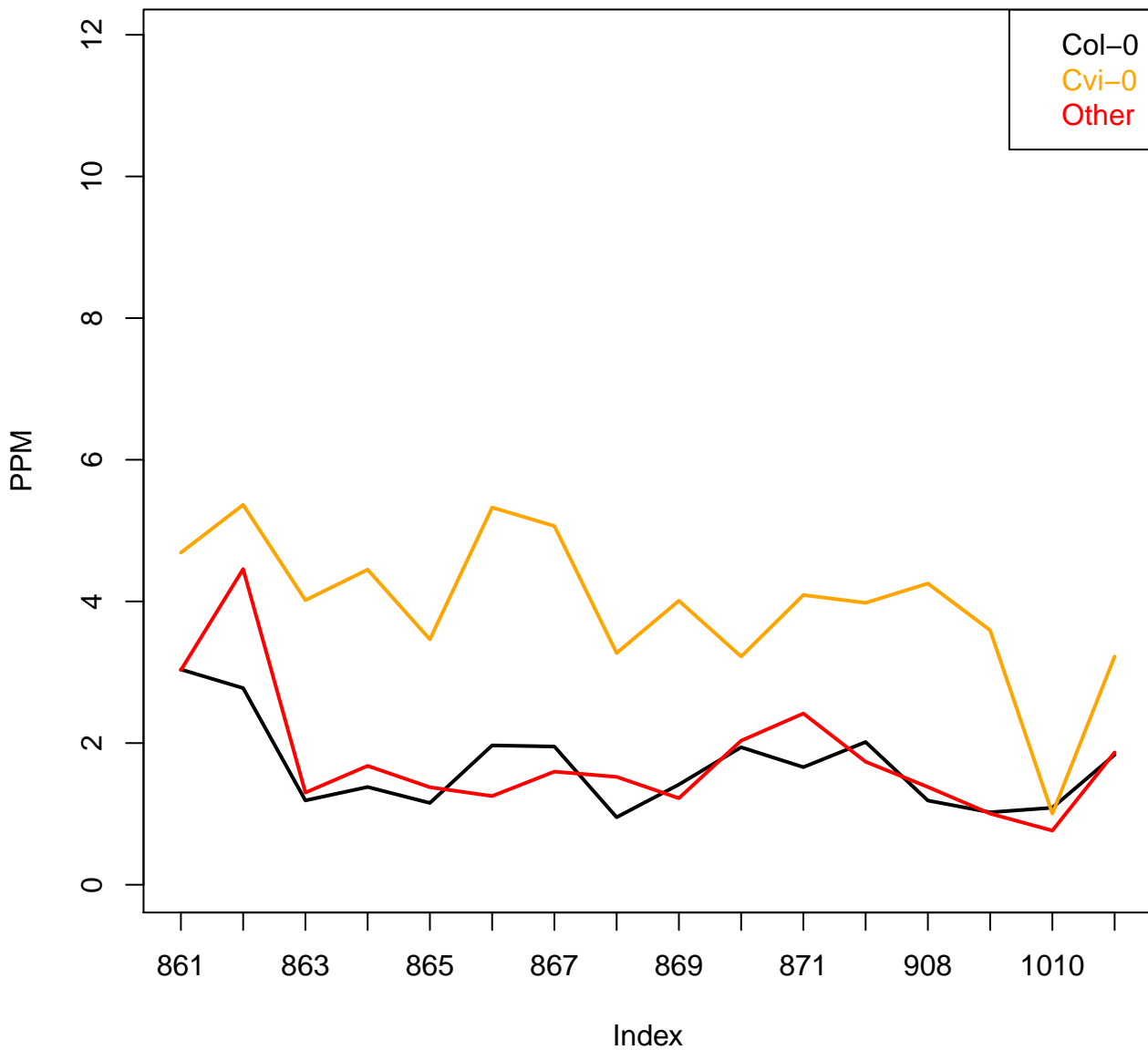

# Cd111

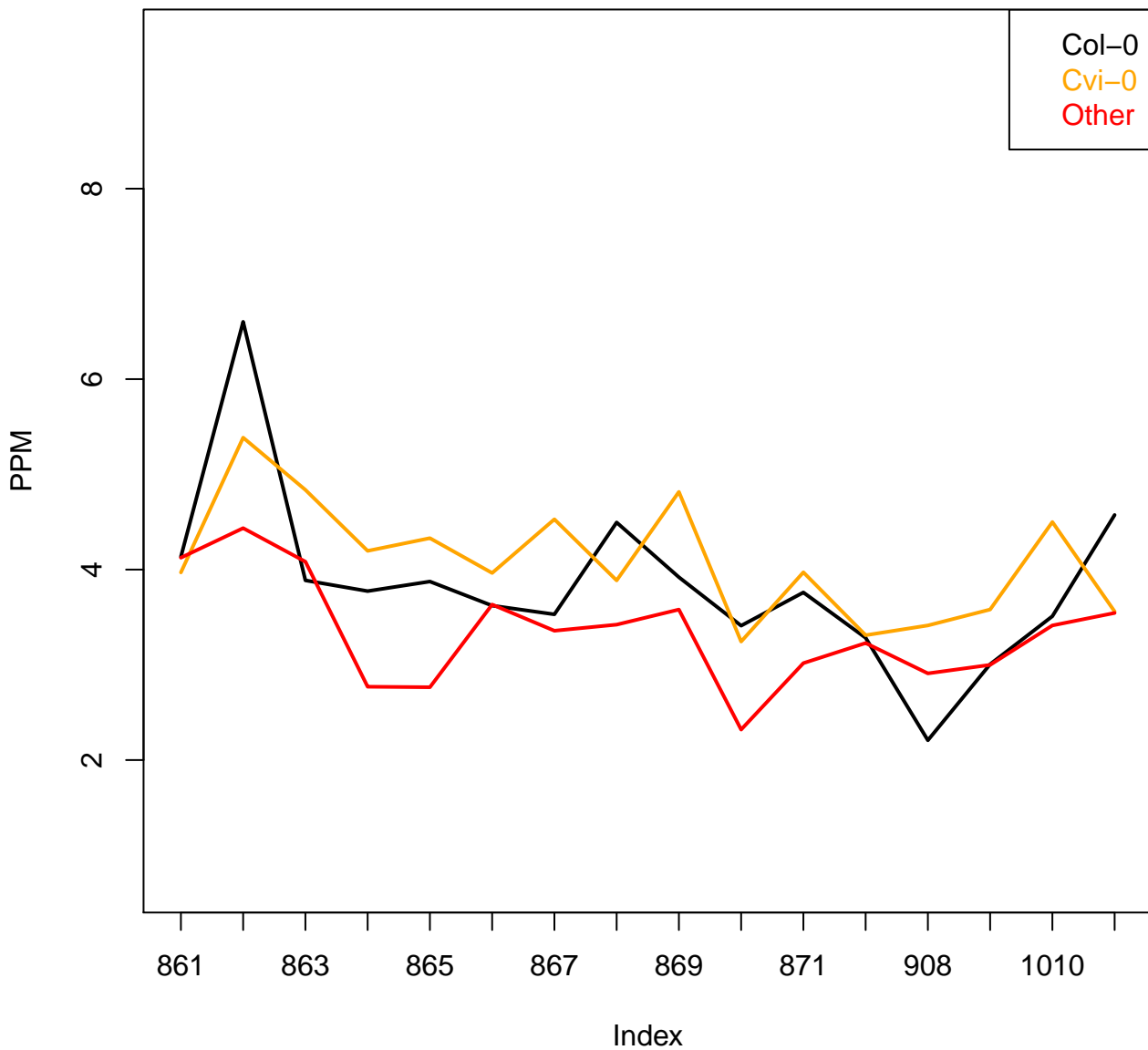

Supplement: Figure S2 — Plot of control line averages for each tray before normalization for Soil Leaf 1 experiment. All non-control lines are averaged into the “Other” line. (PDF) [file pone.0035121.s004.pdf]

# Li7

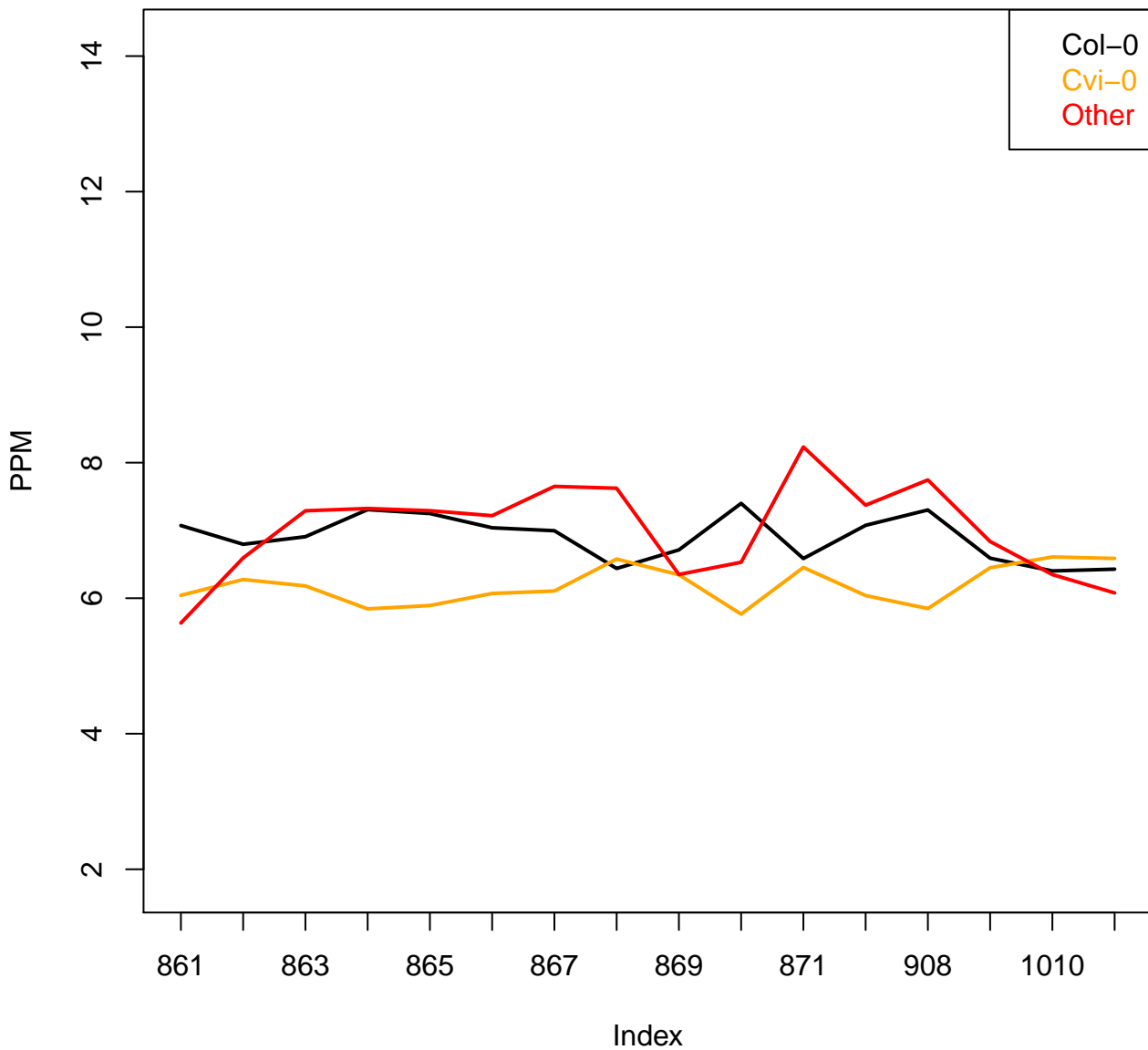

# B11

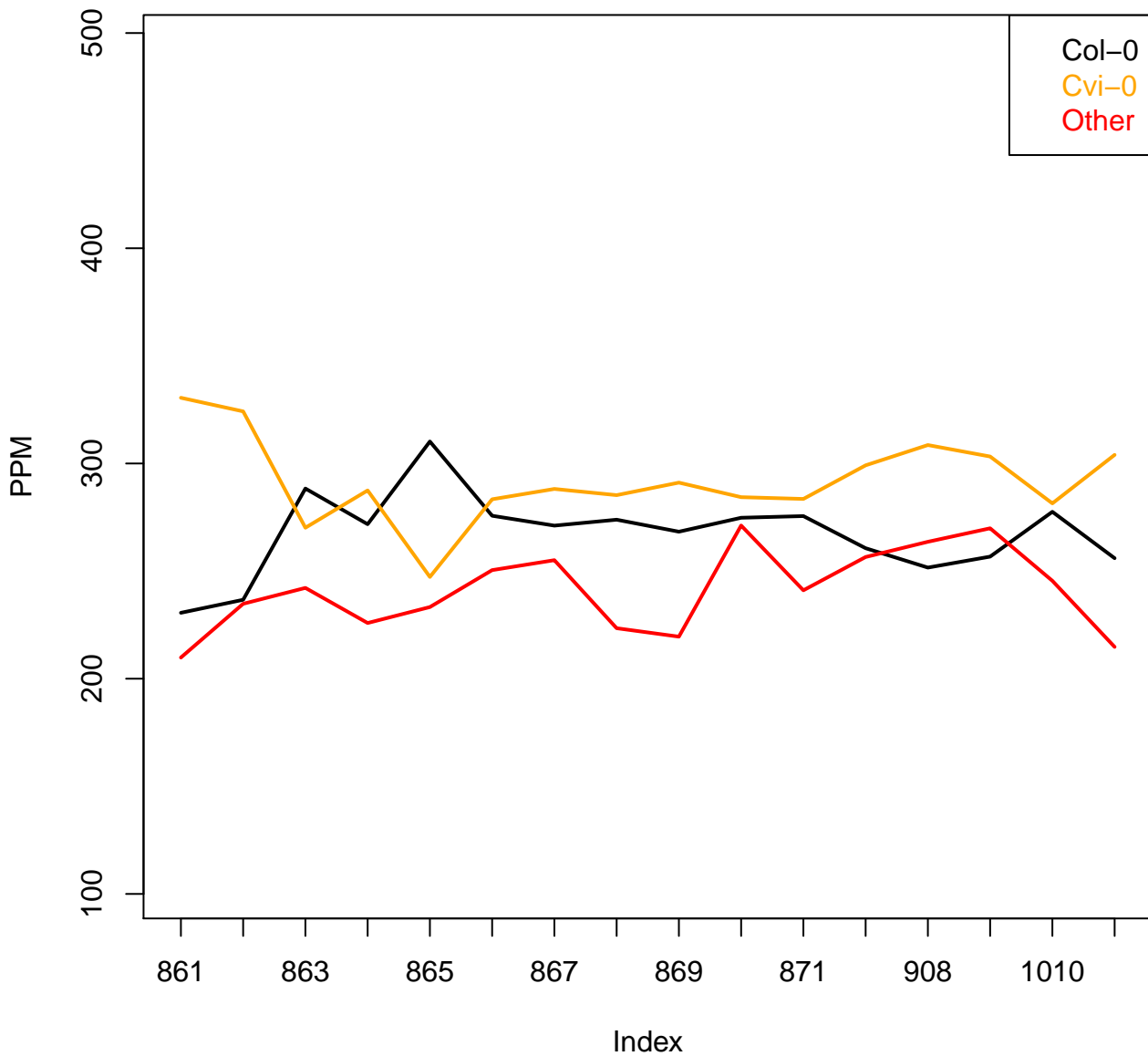

# Na23

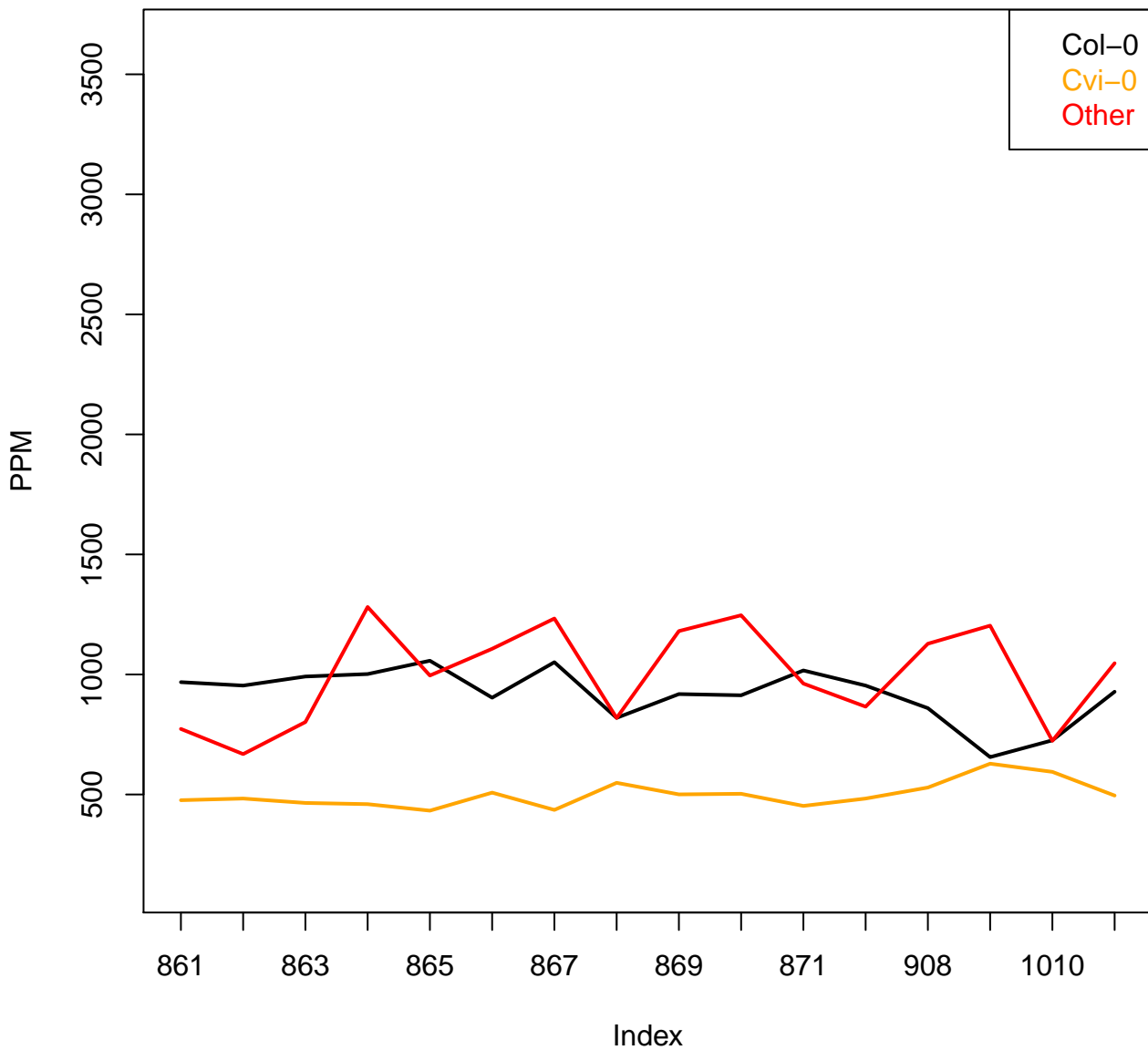

# Mg25

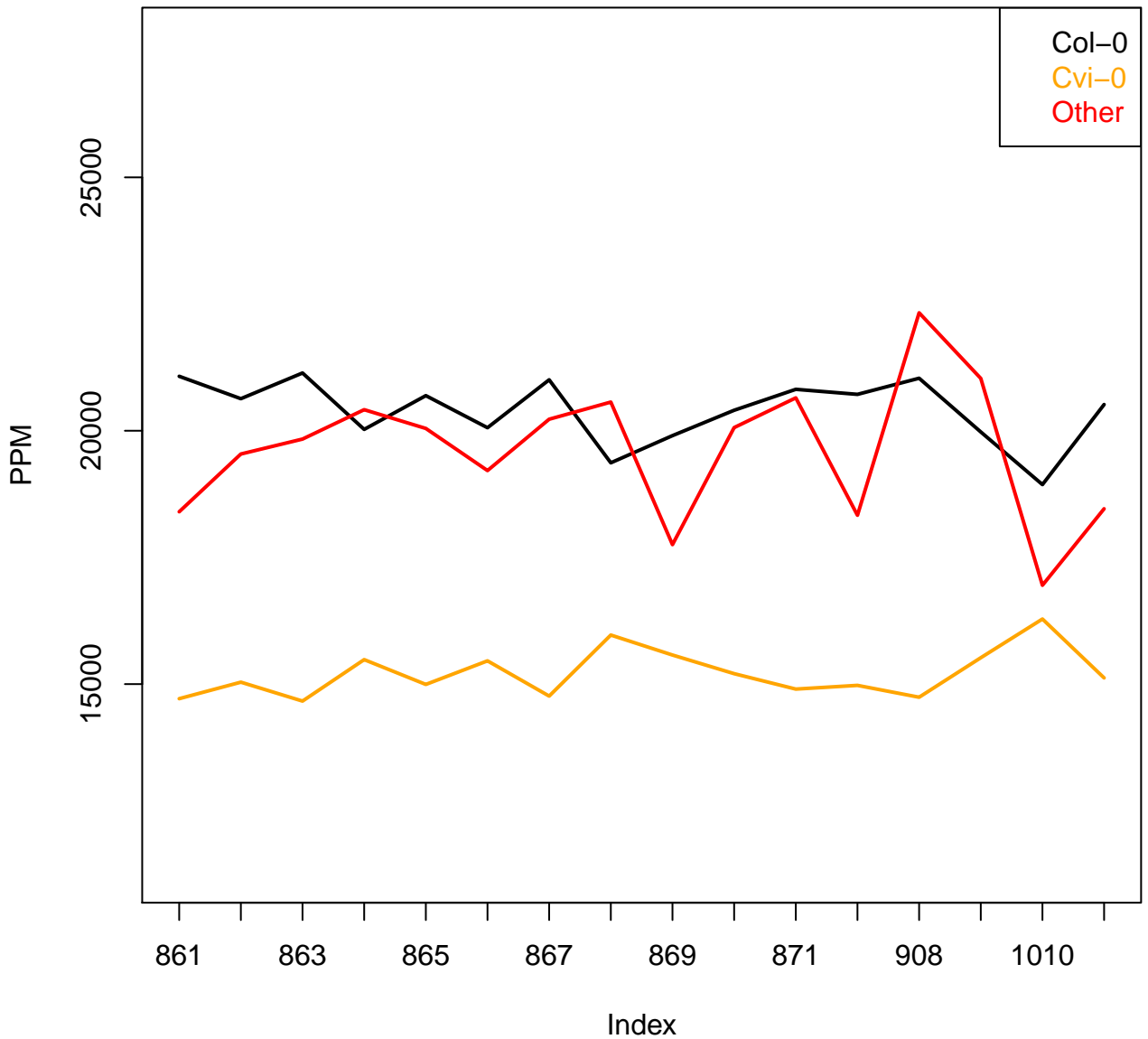

# P31

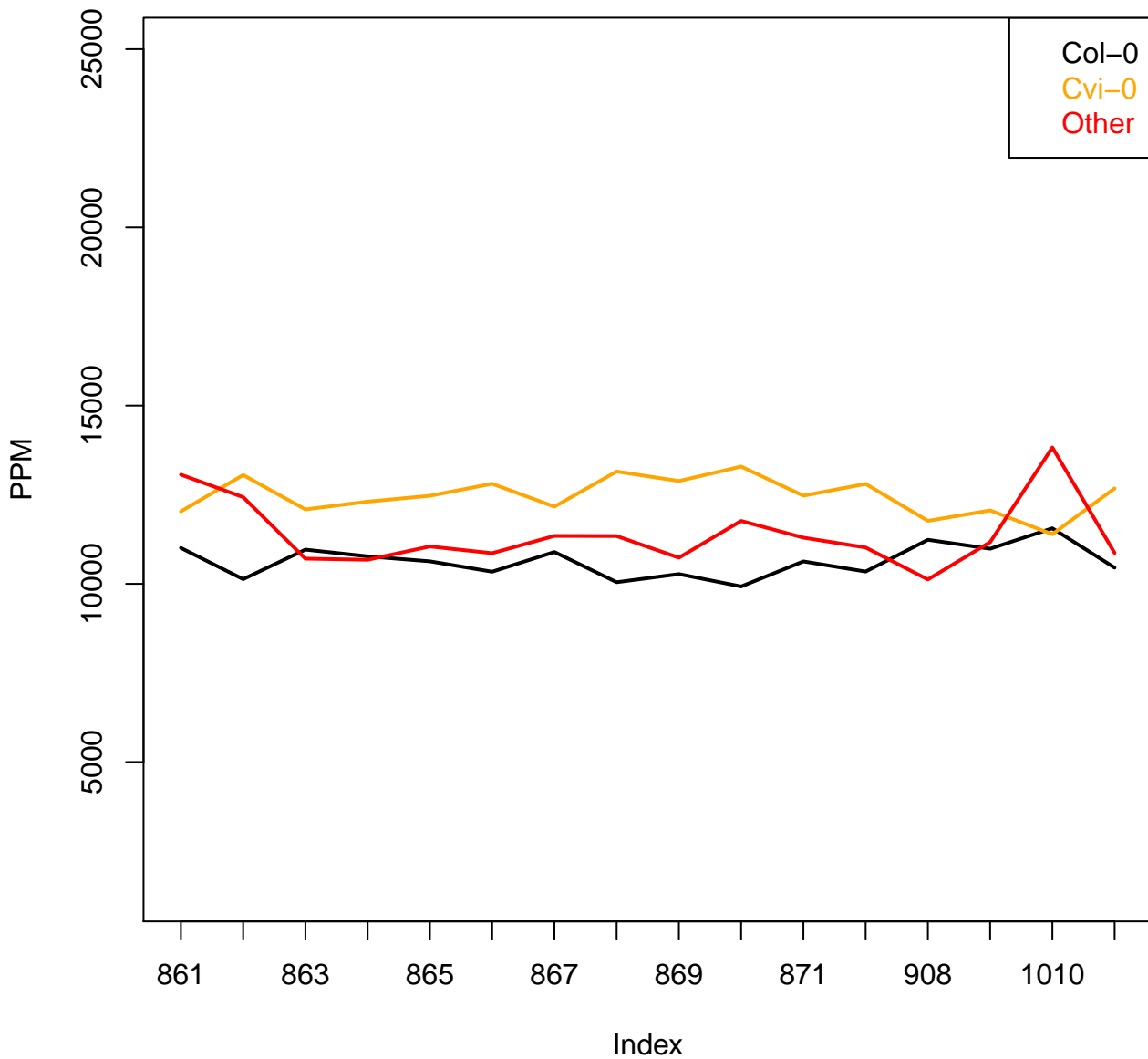

# K39

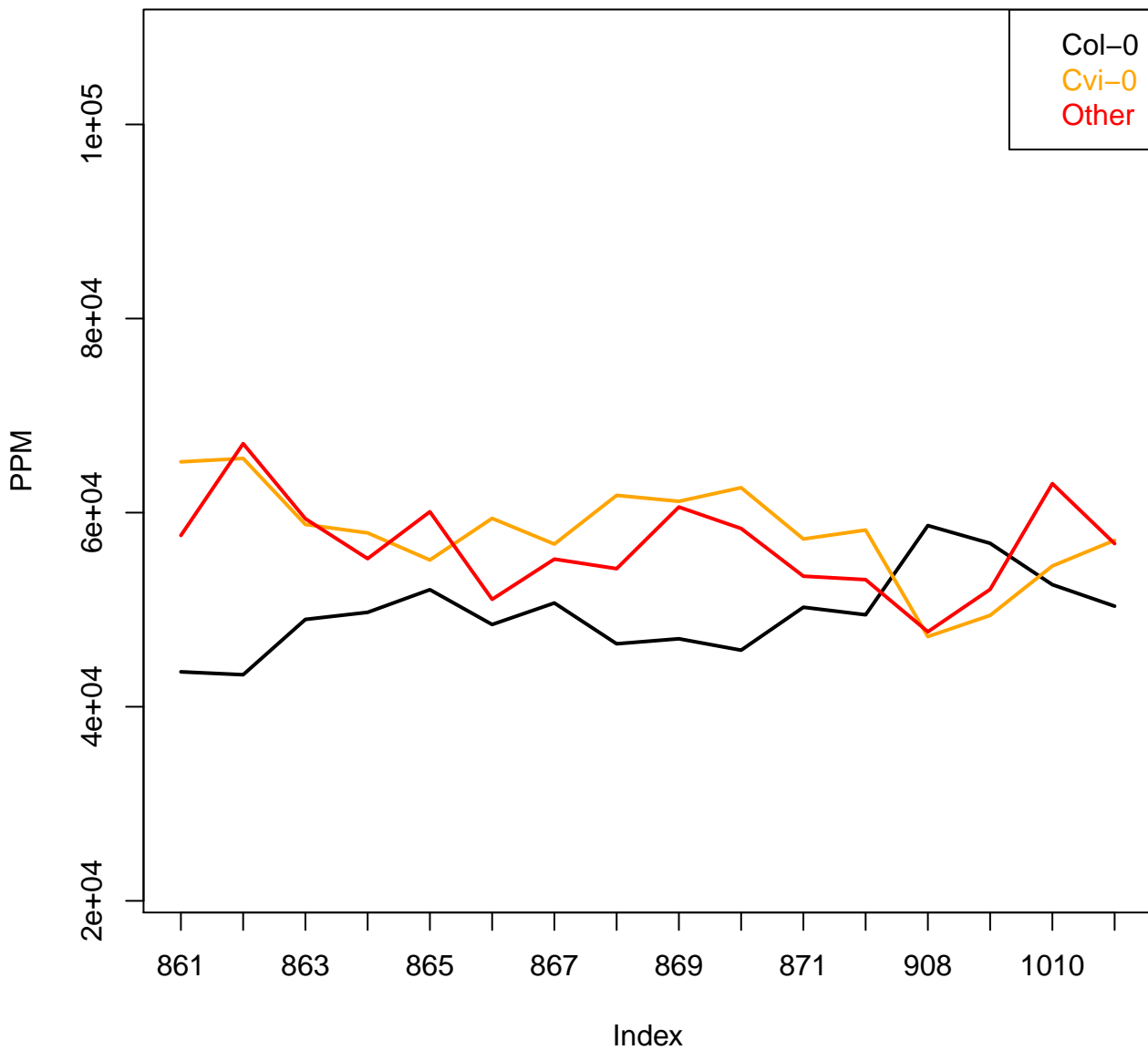

# Ca43

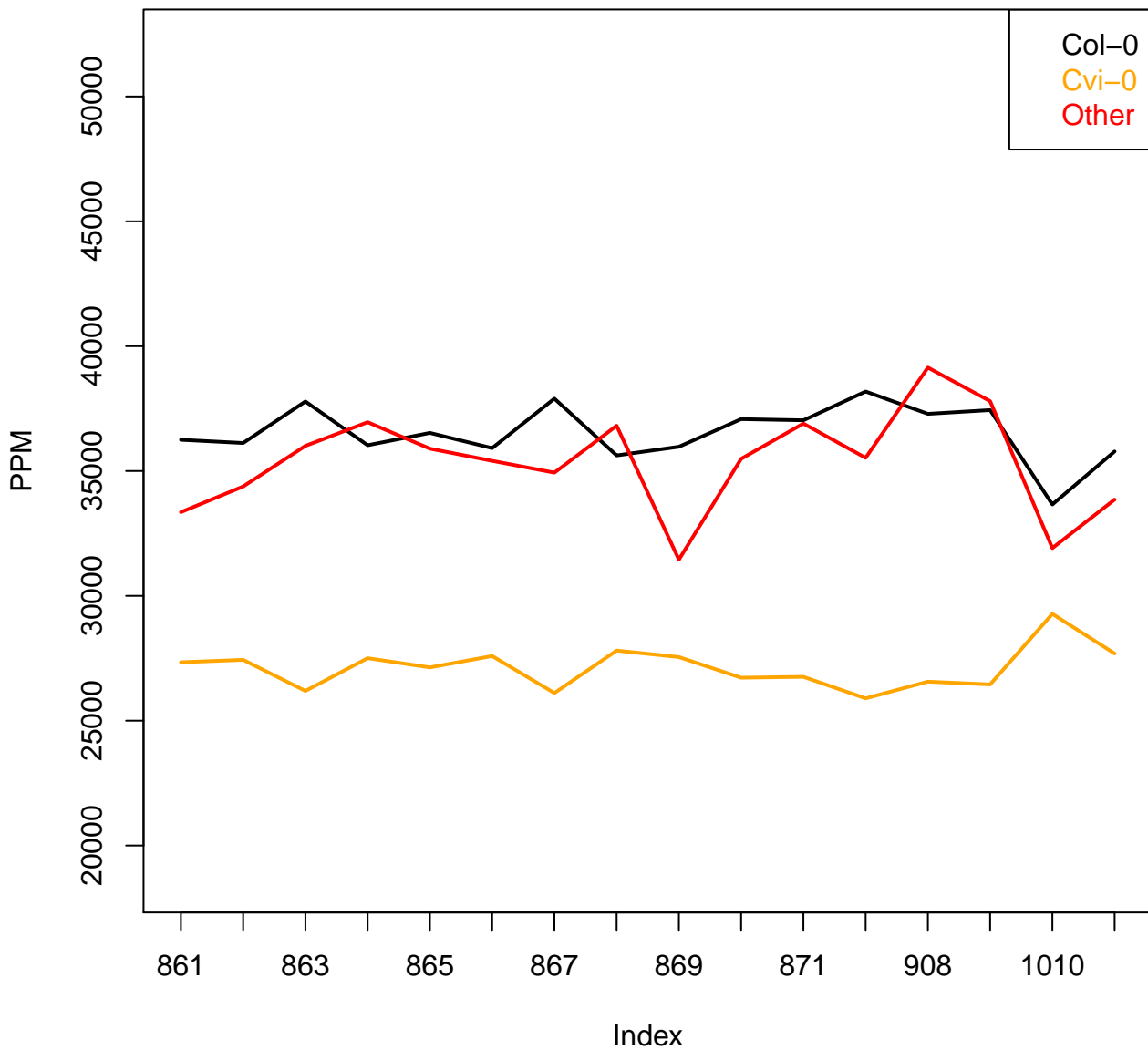

# Mn55

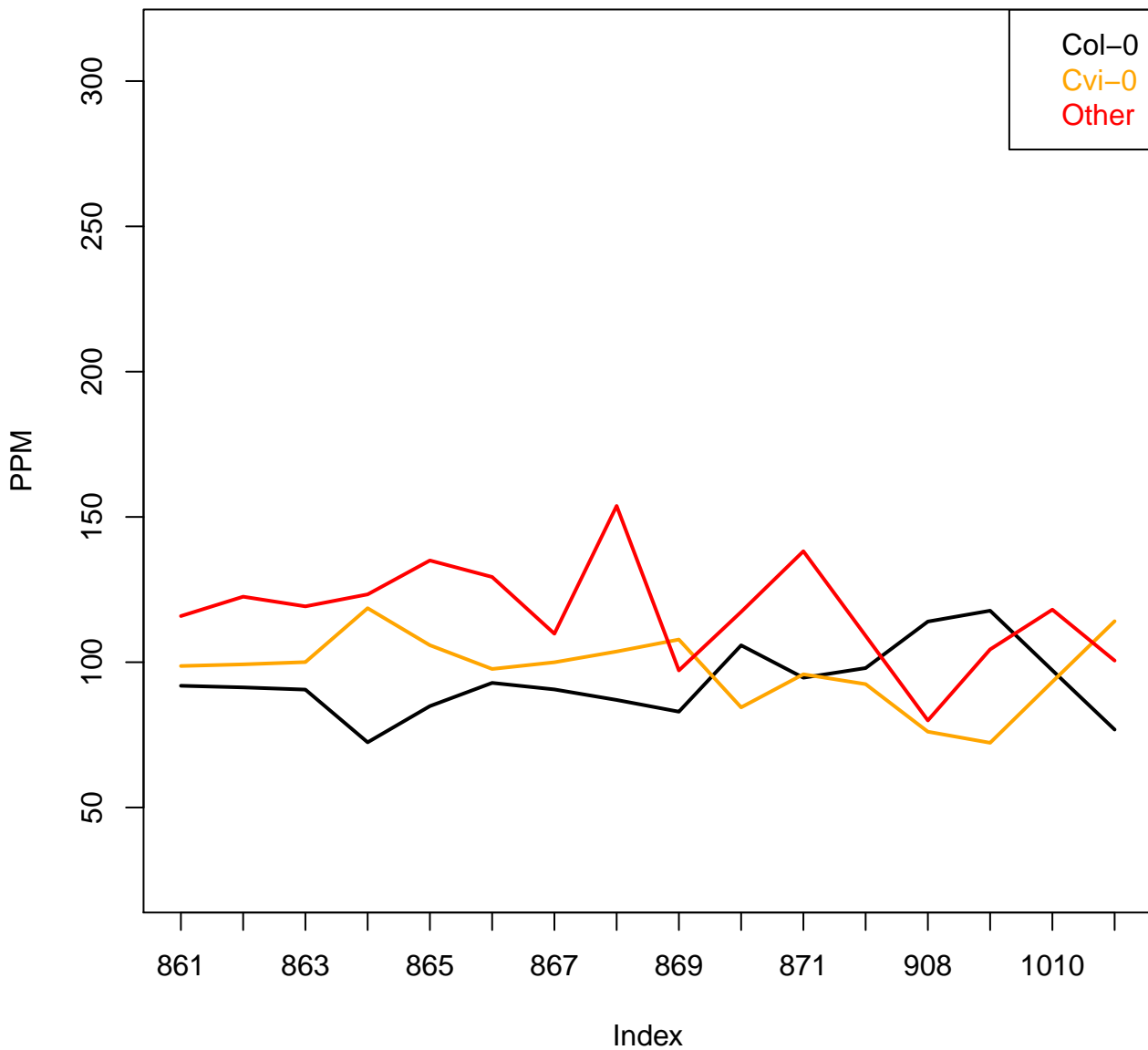

# Fe56

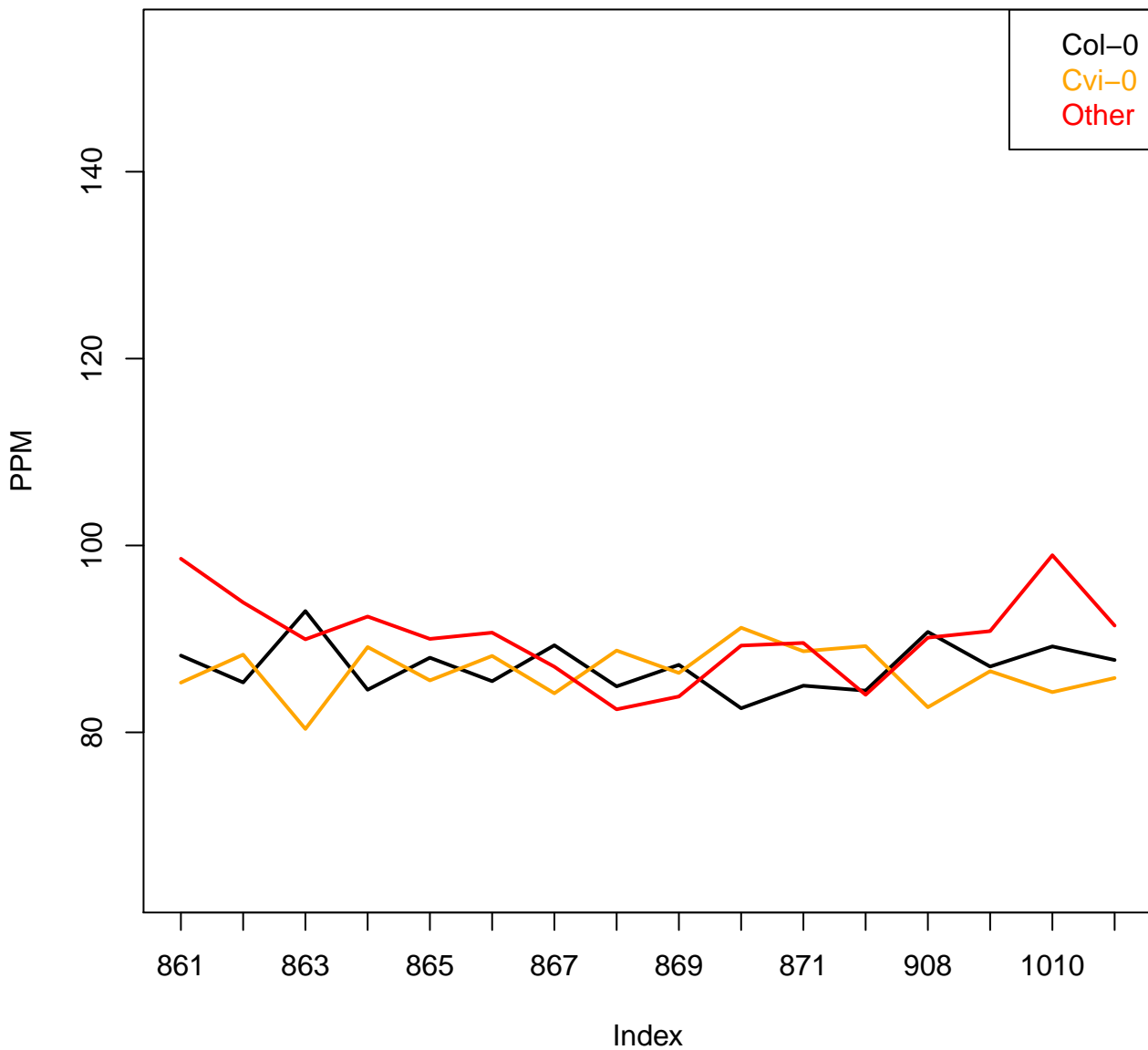

# Co59

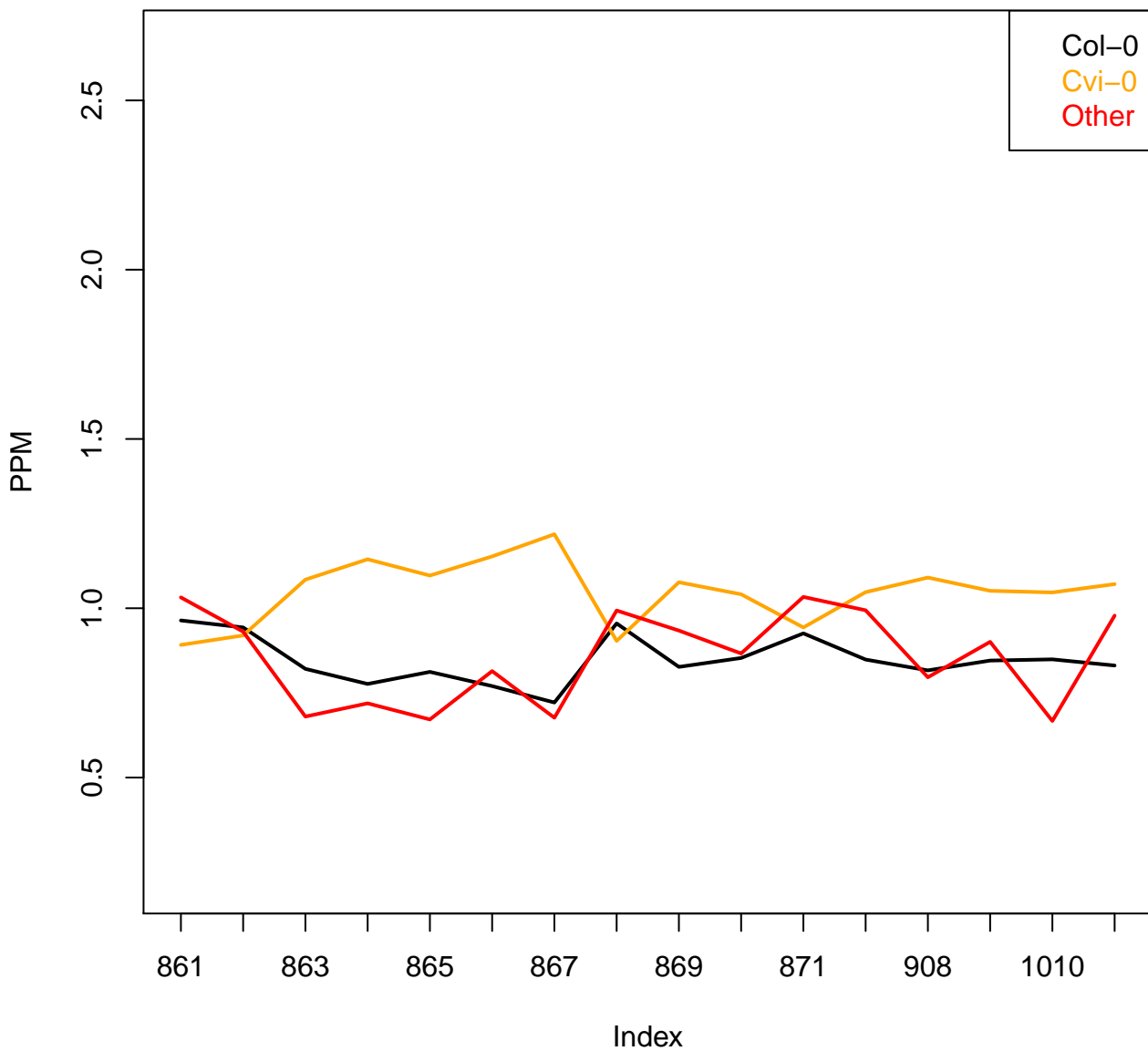

# Ni60

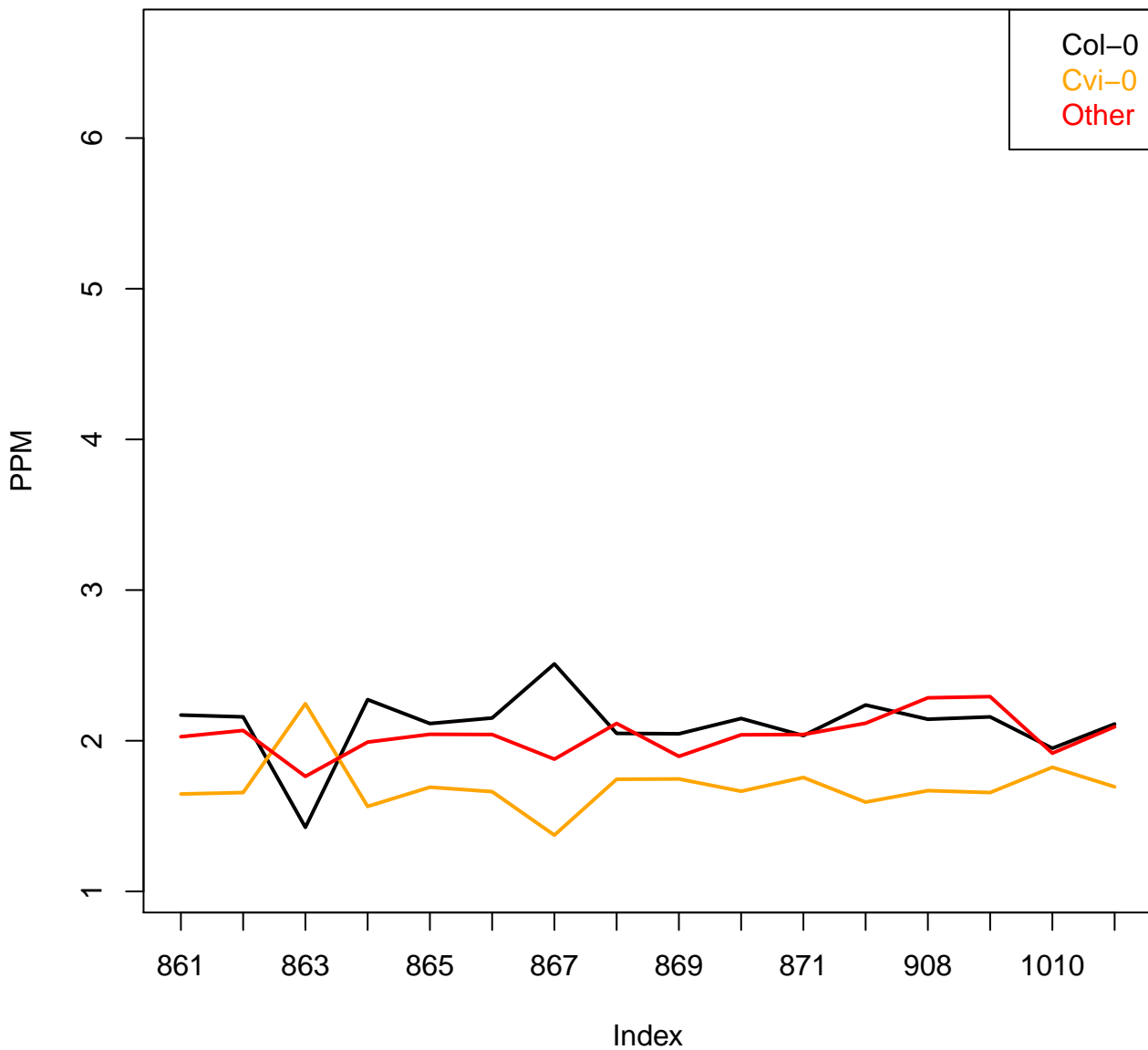

# Cu65

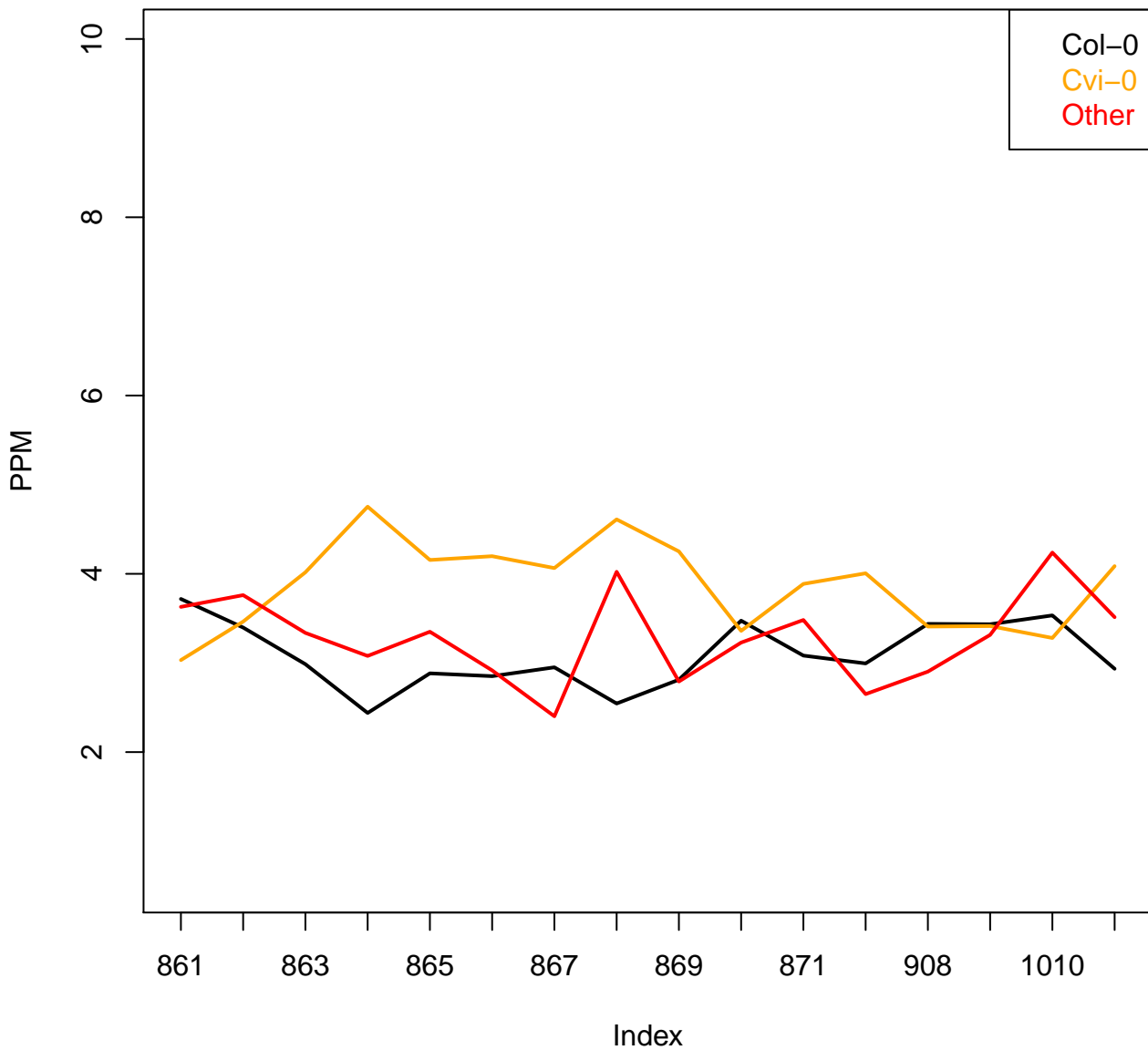

# Zn66

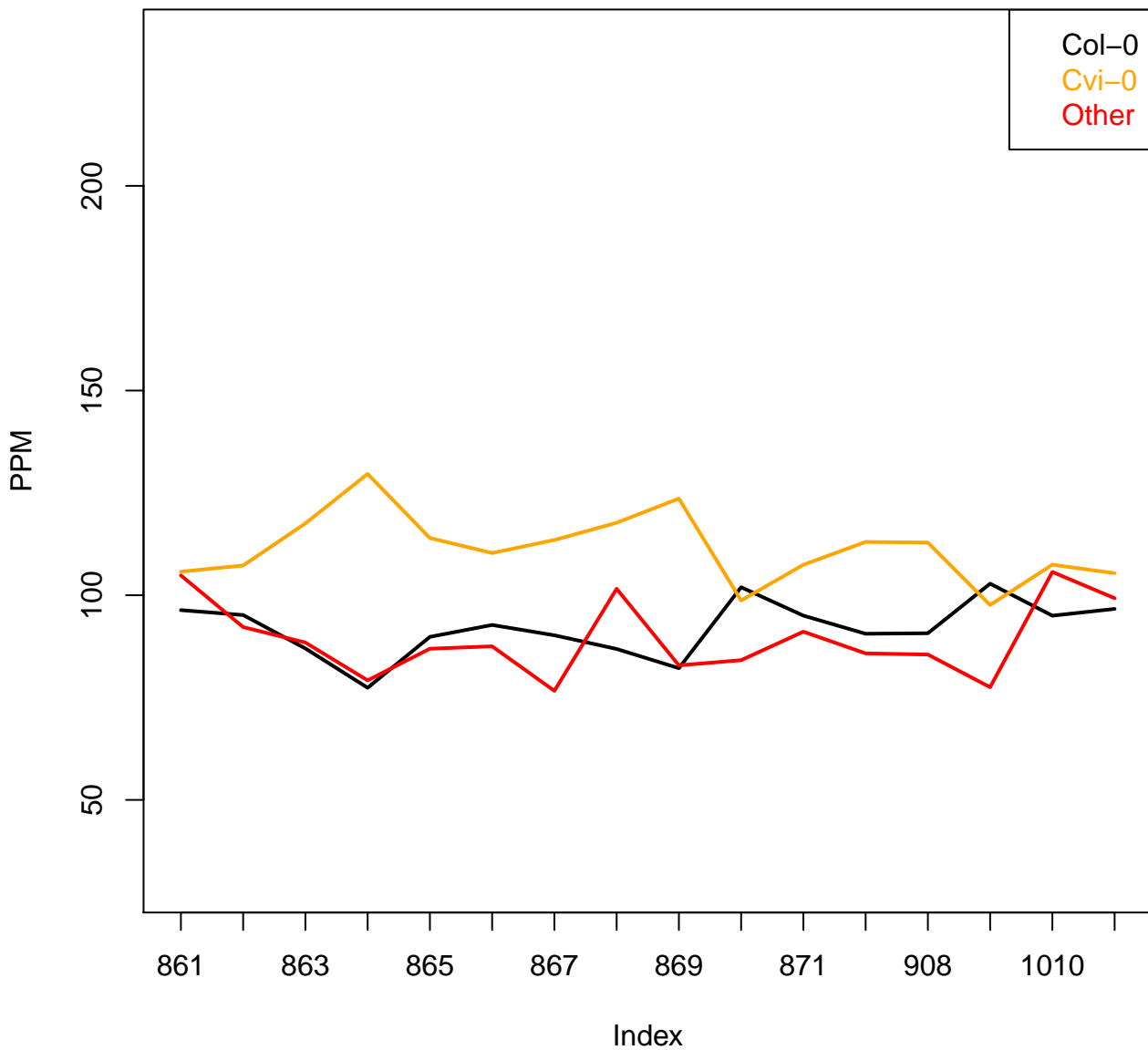

# As75

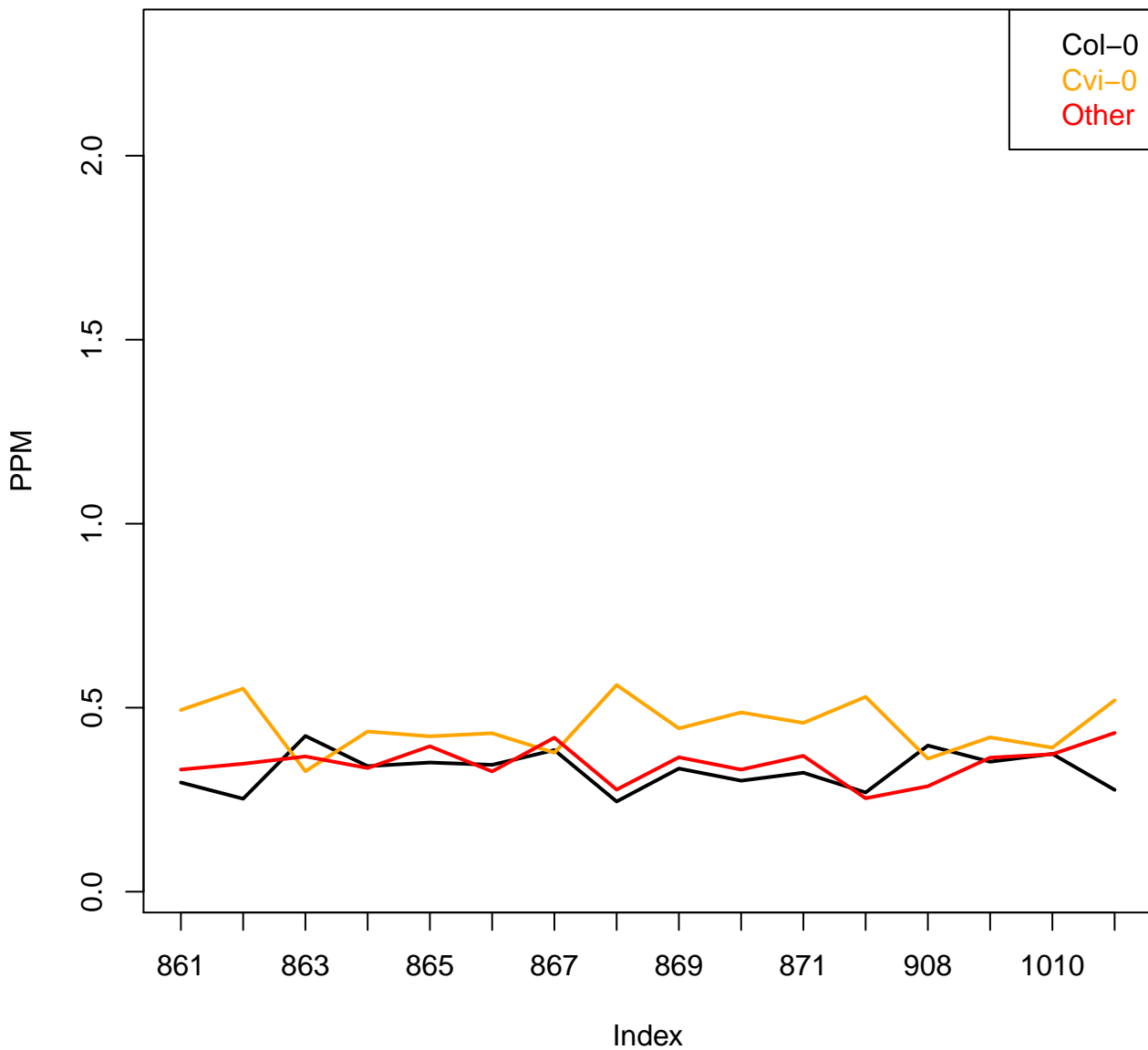

# Se77

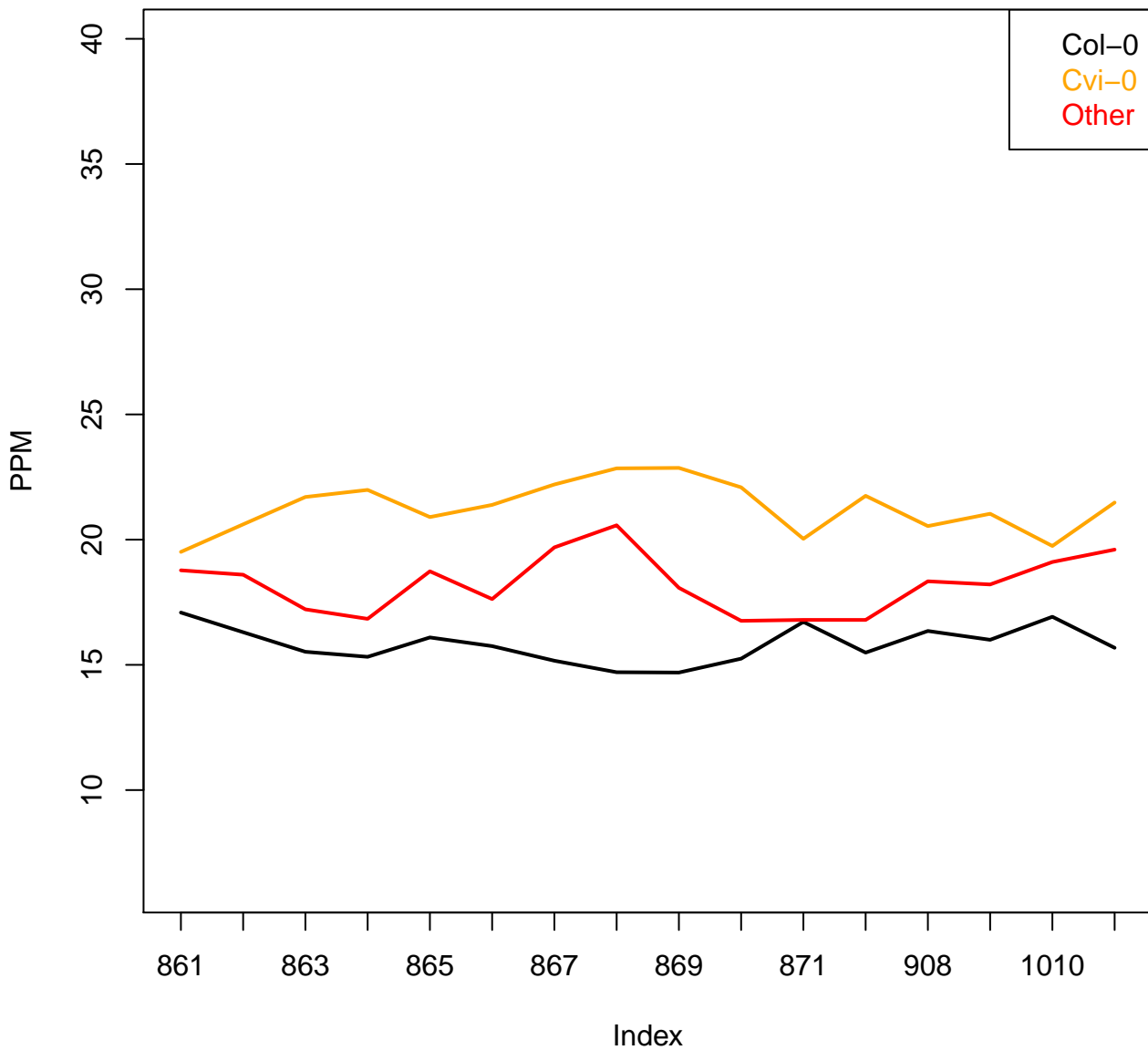

# Mo95

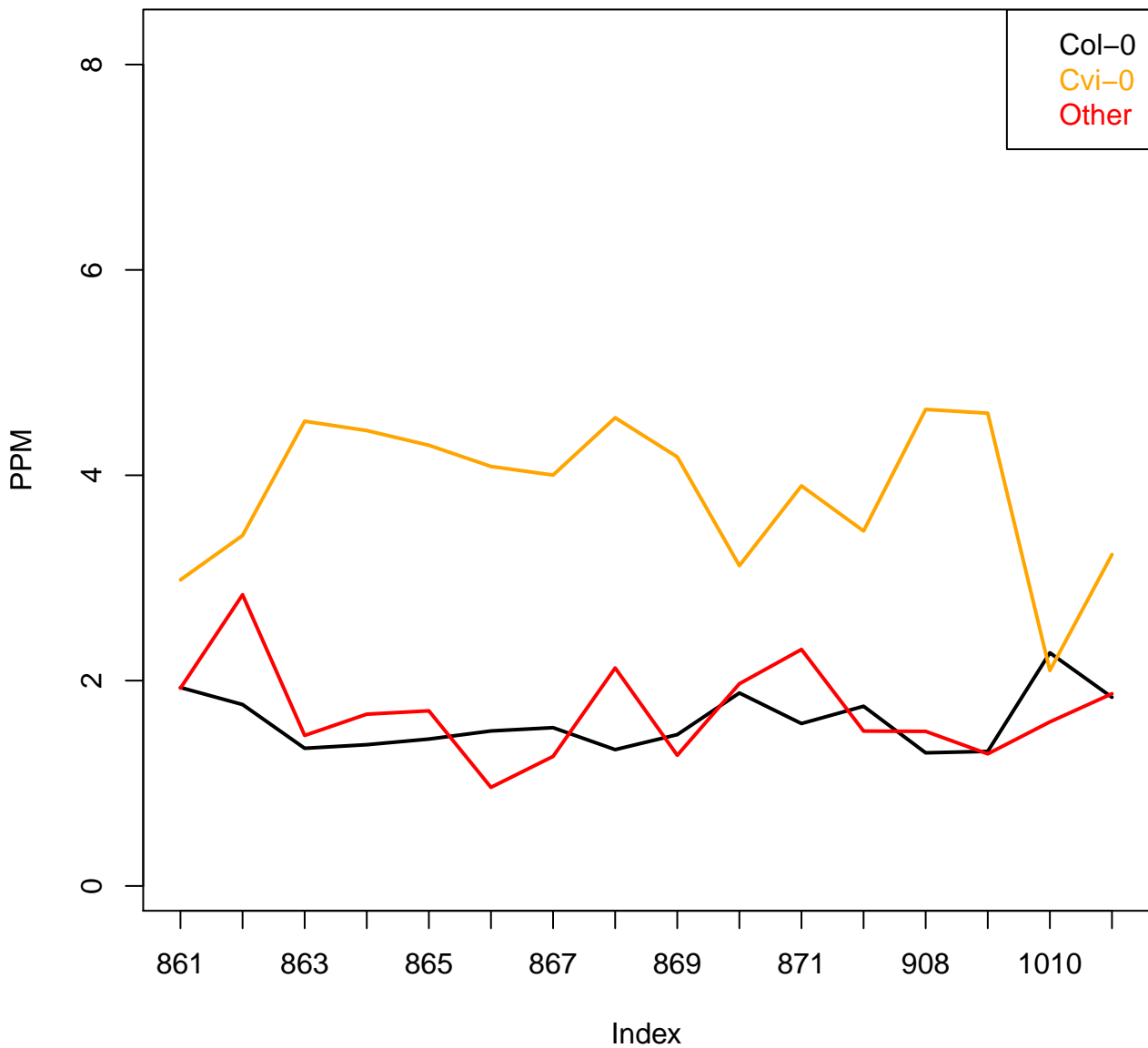

# Cd111

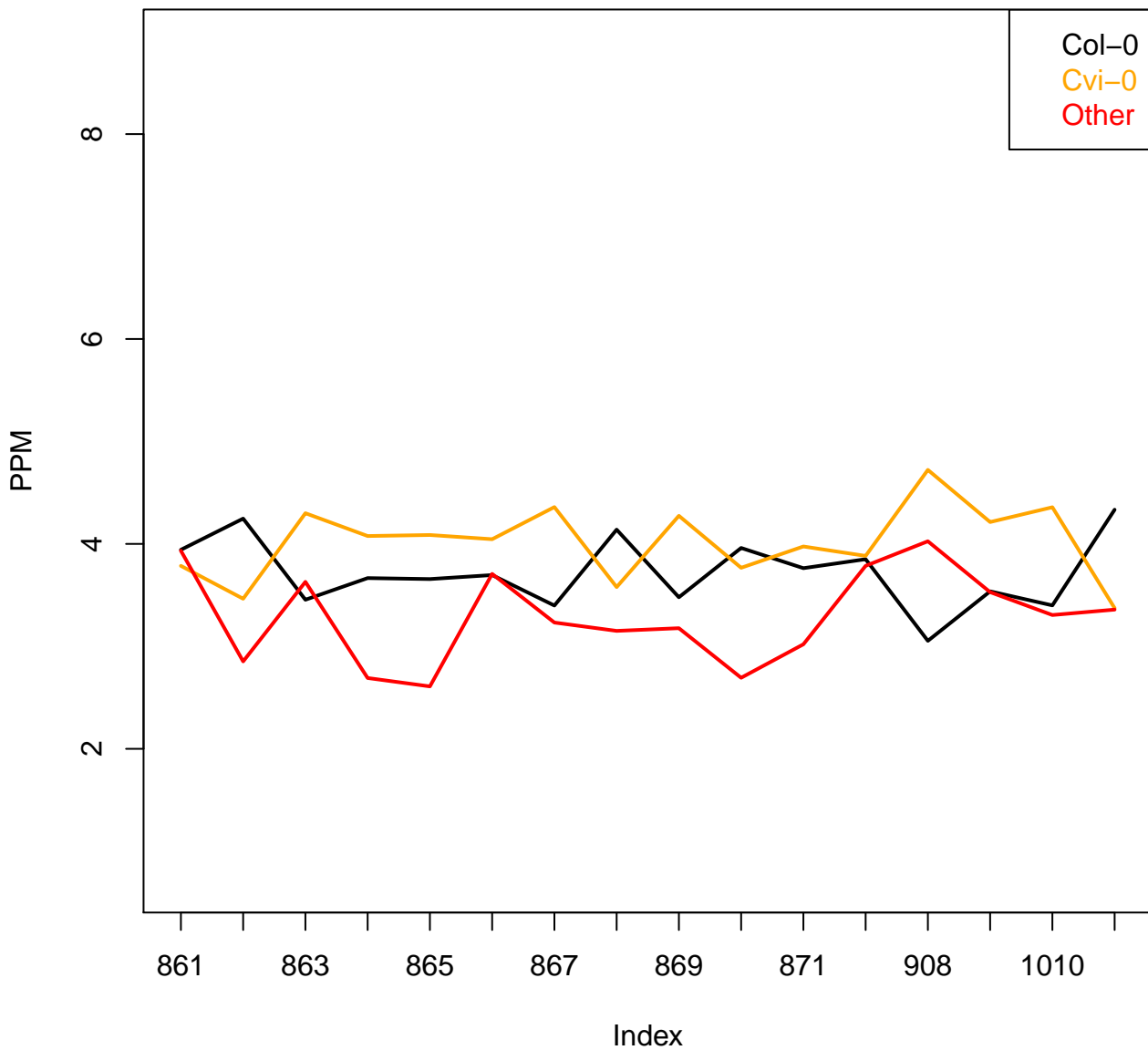

Supplement: Figure S3 — Plot of control line averages for each tray after normalization for Soil Leaf 1 experiment. All non-control lines are averaged into the “Other” line. (PDF) [file pone.0035121.s005.pdf]

# Li7

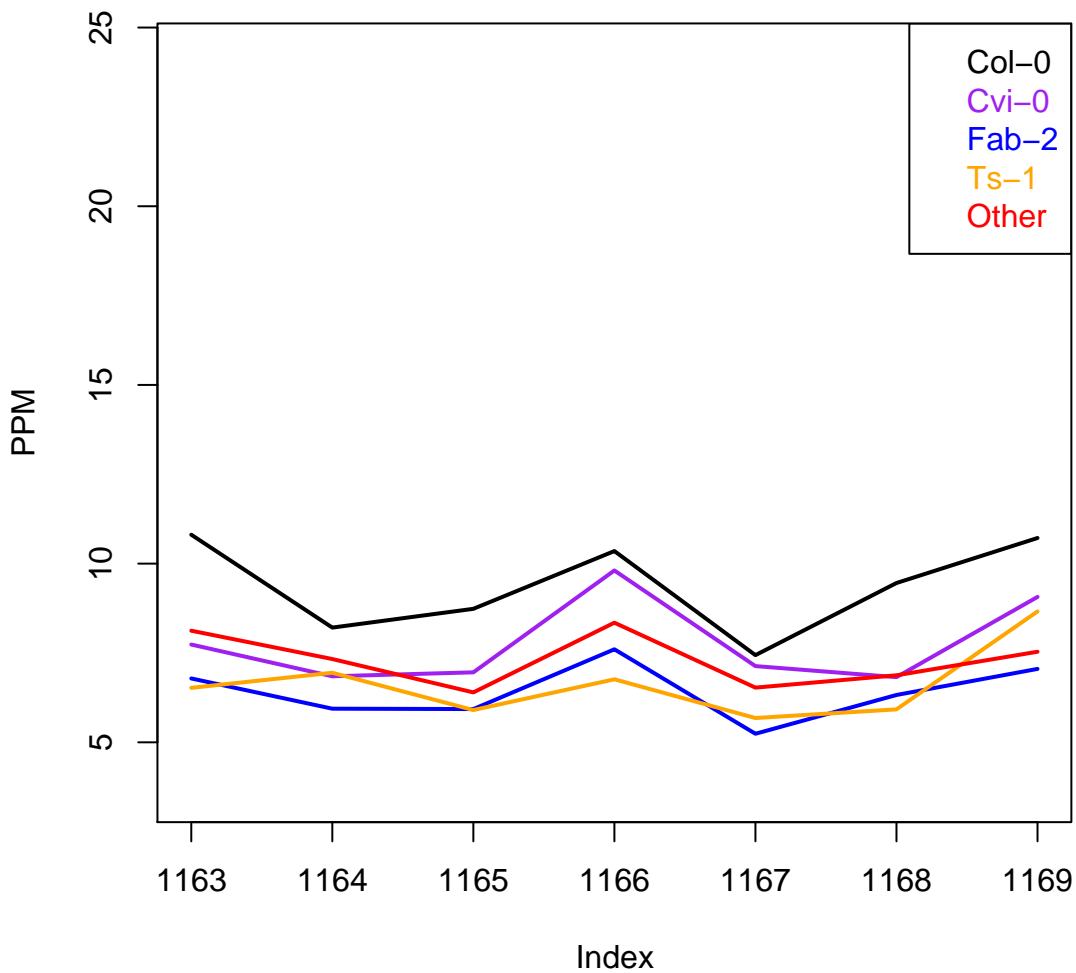

# B11

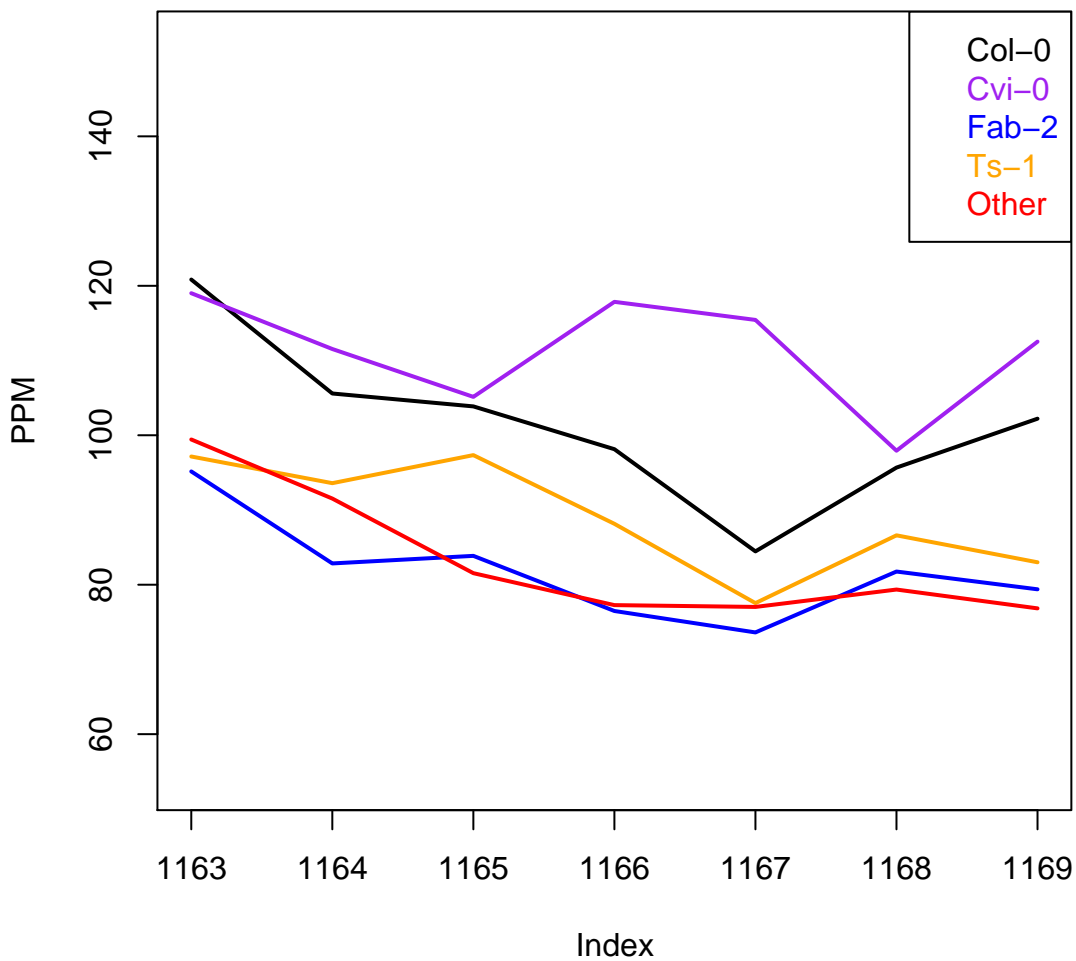

# Na23

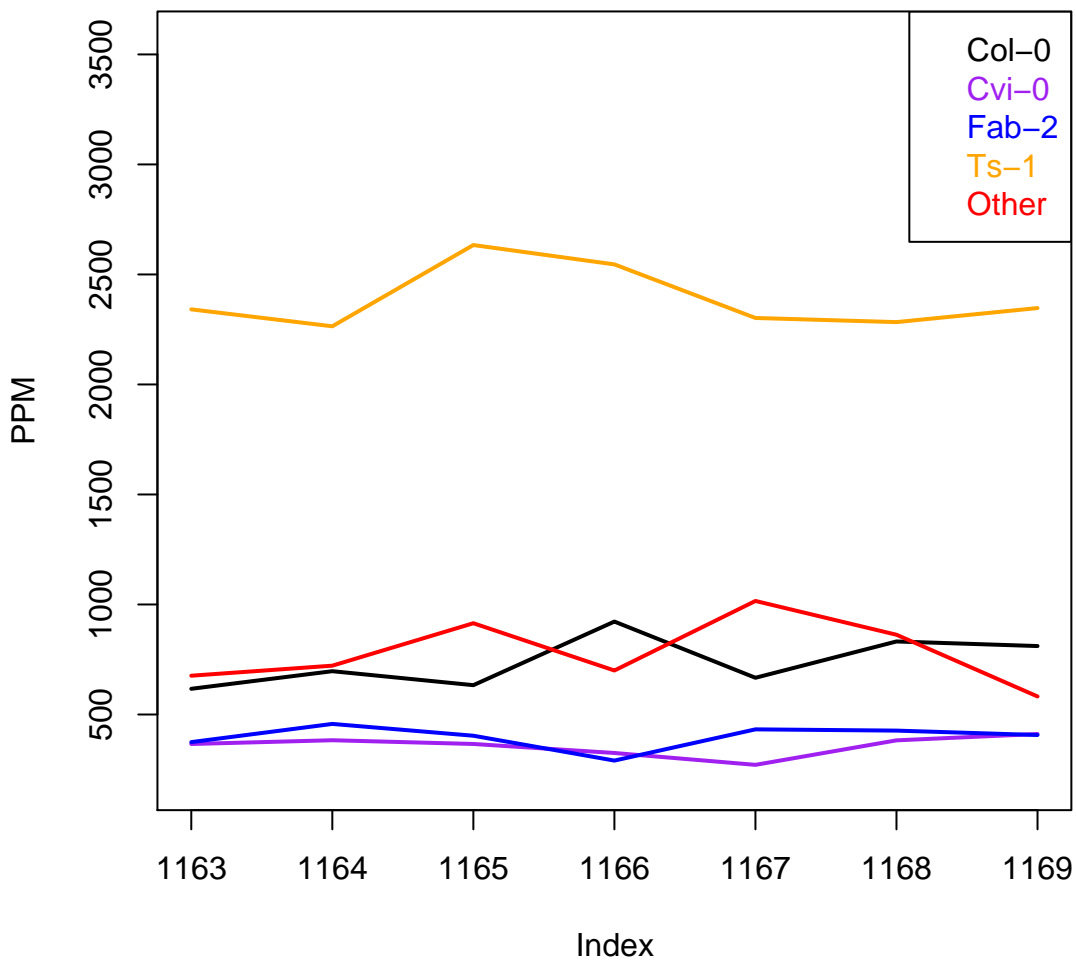

# Mg25

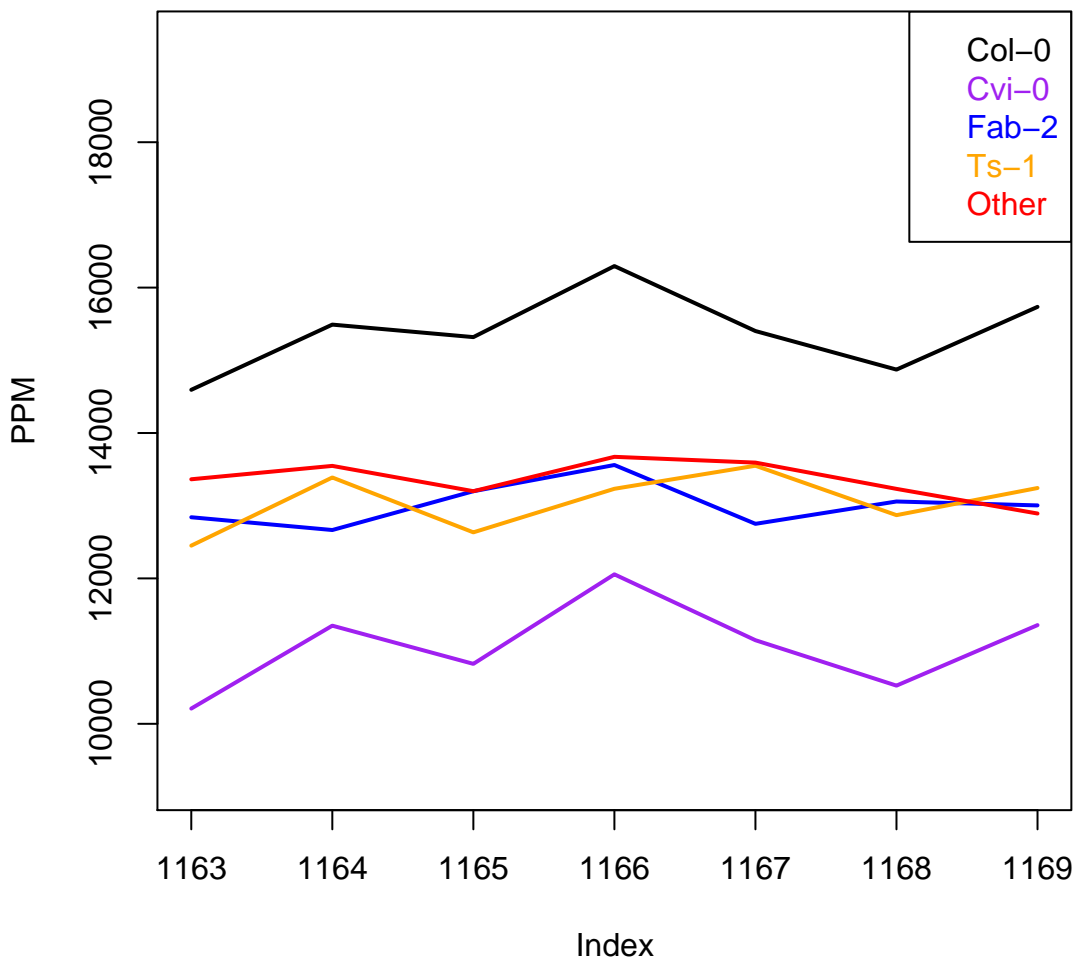

# P31

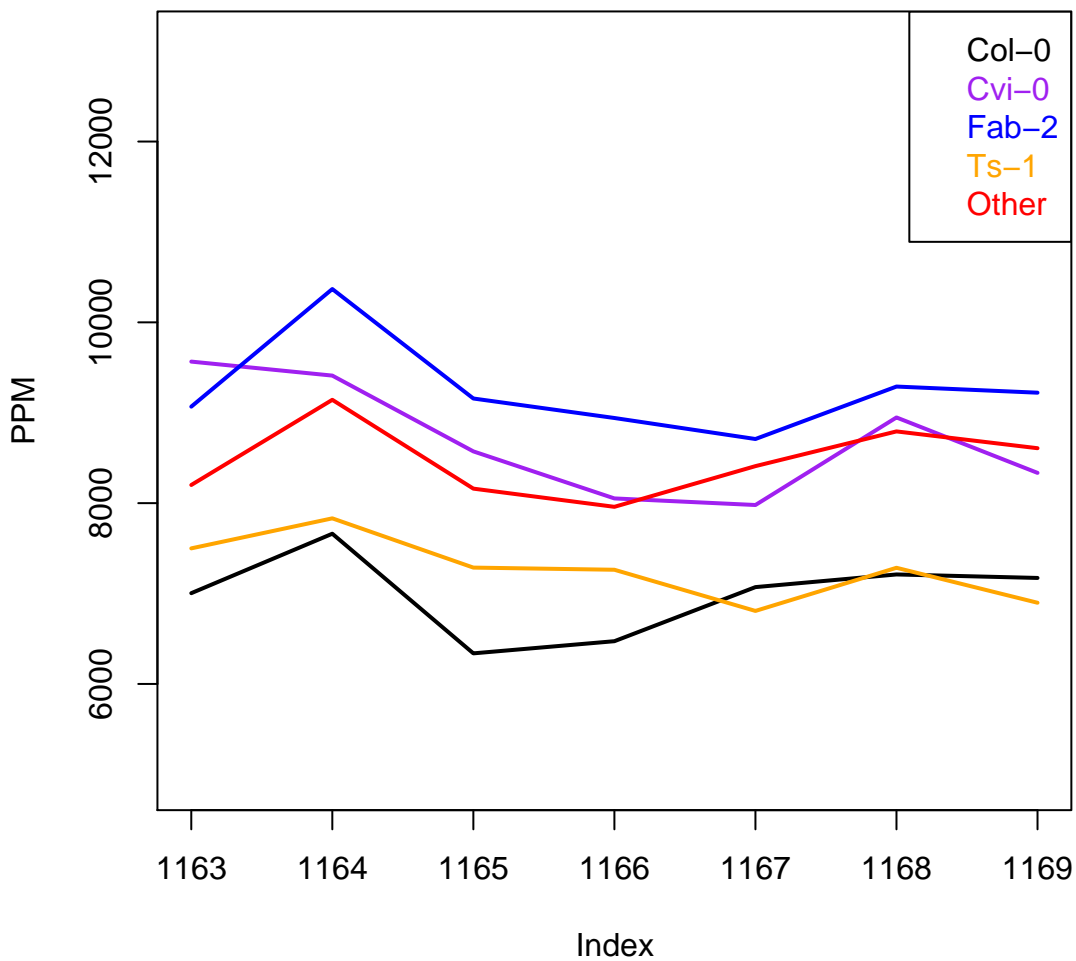

# S34

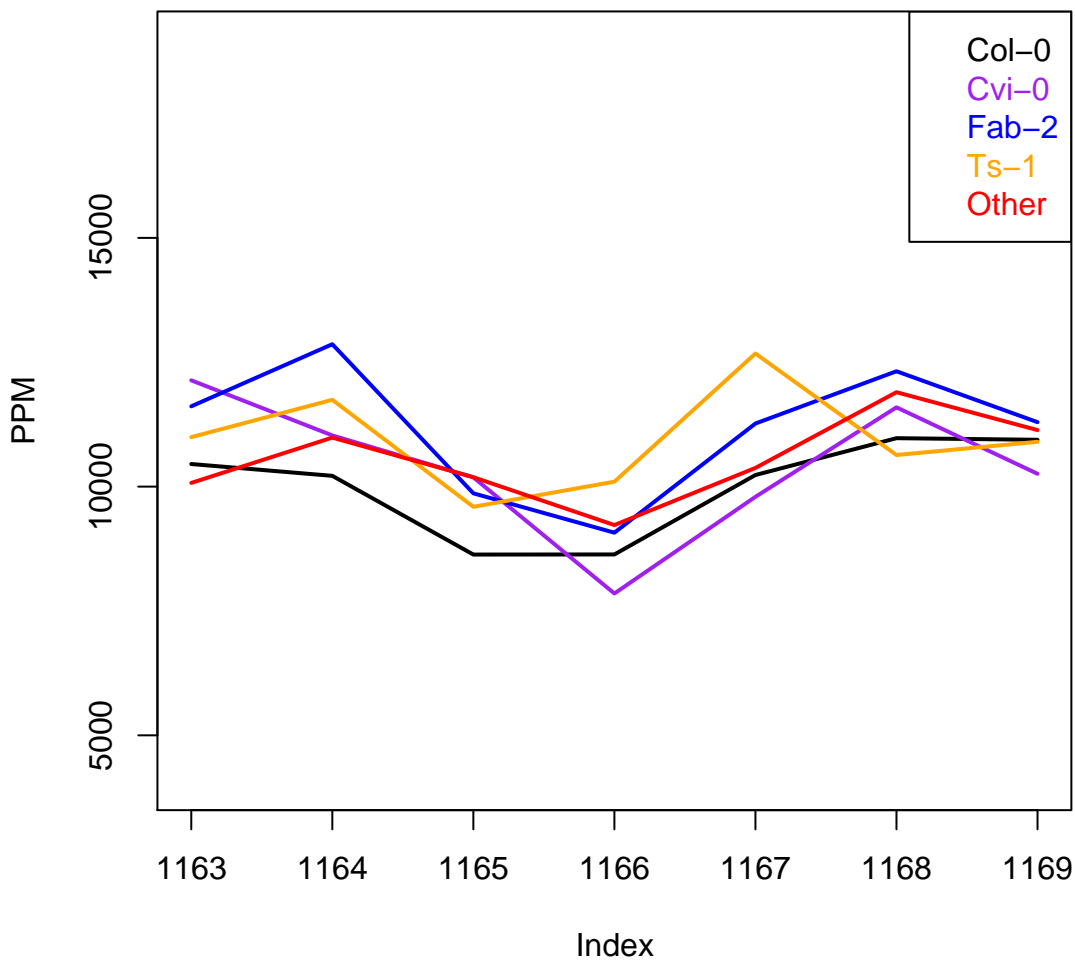

# K39

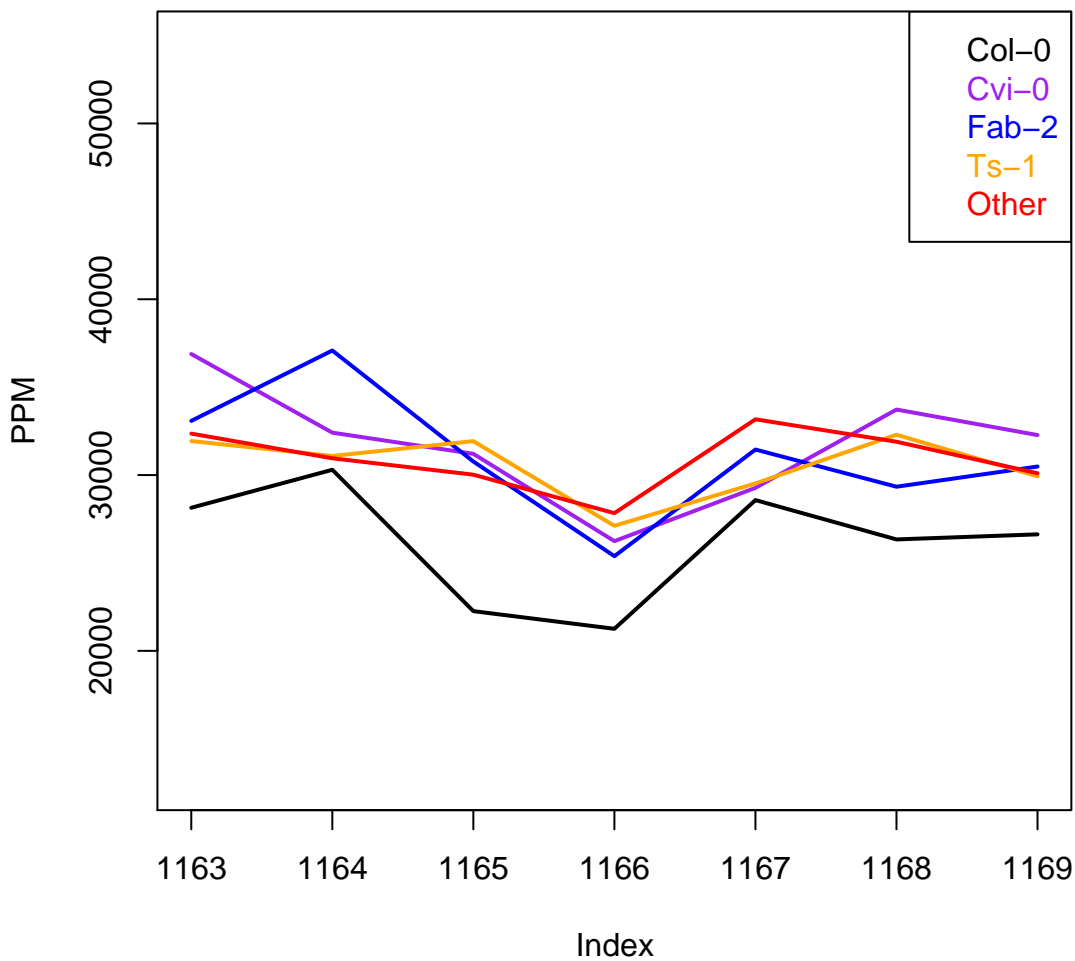

# Ca43

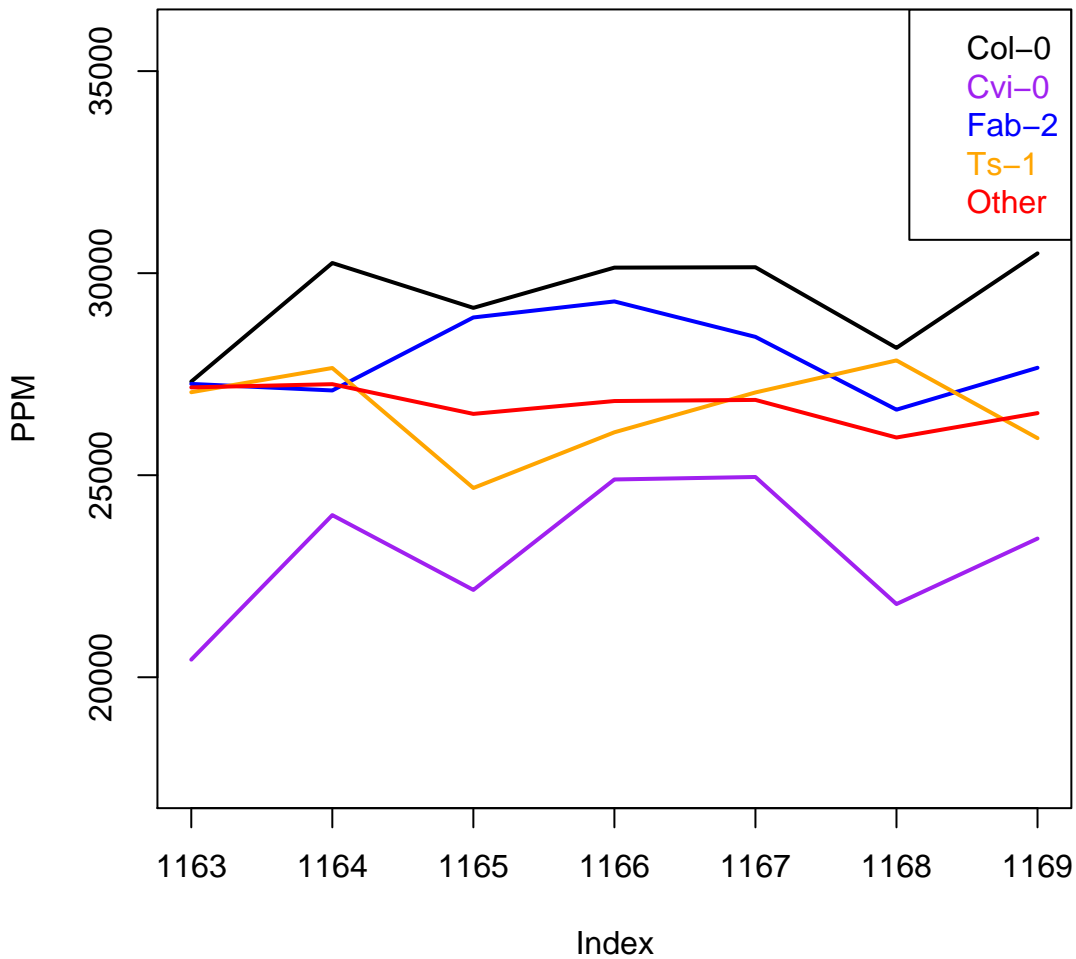

# Mn55

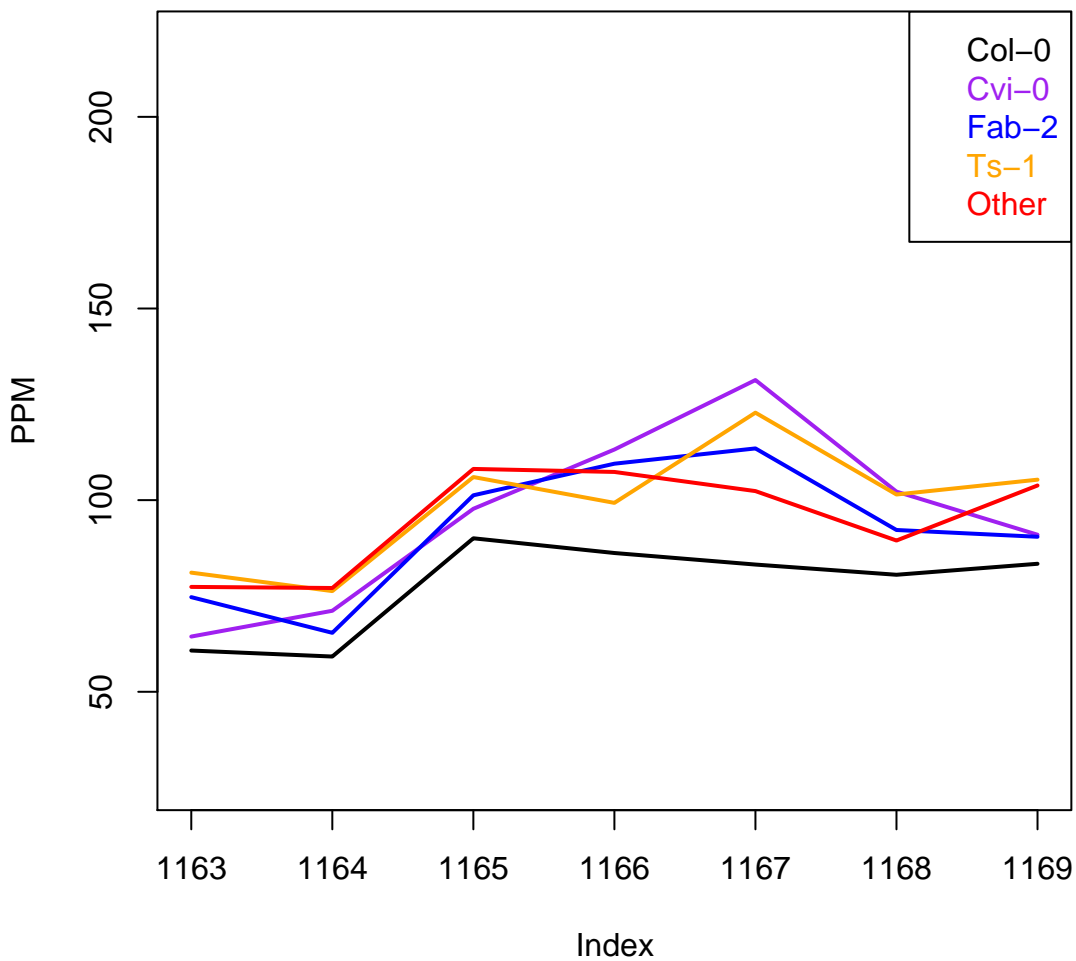

# Fe56

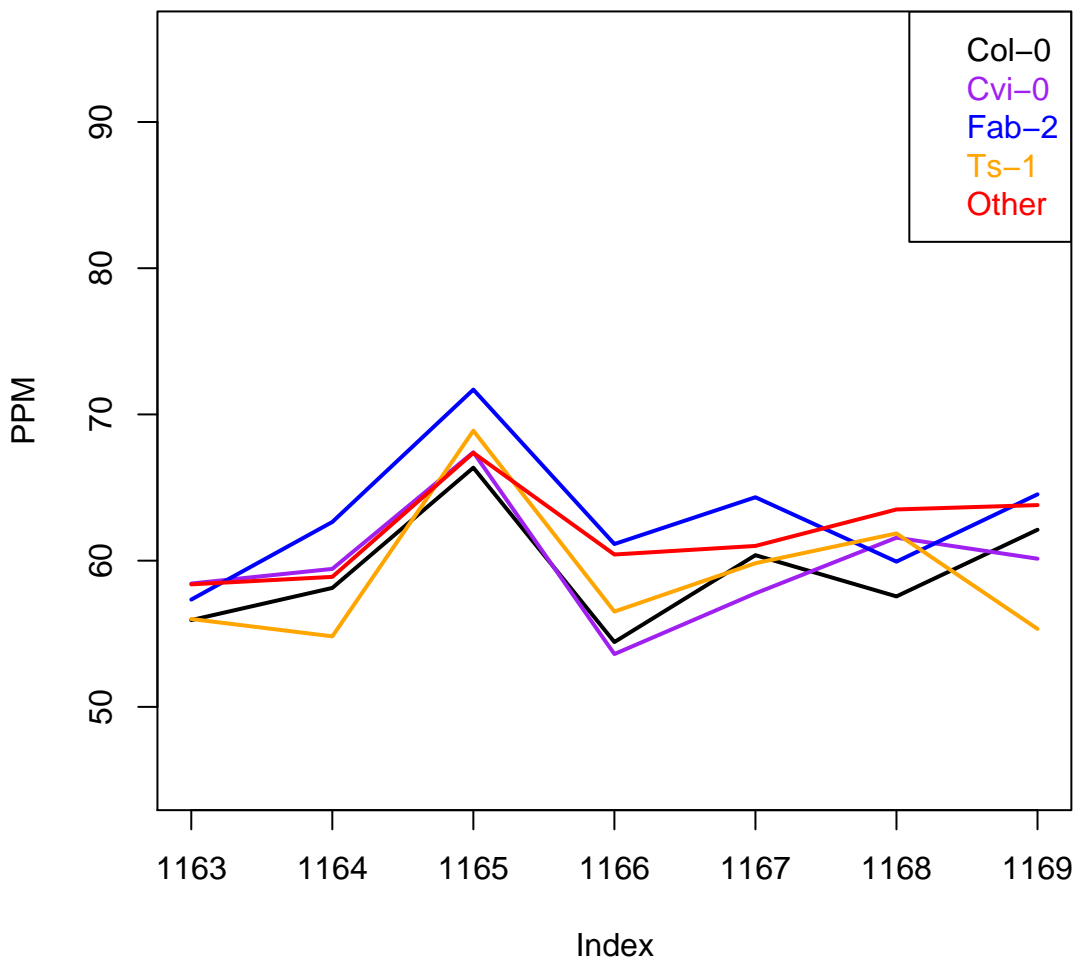

# Co59

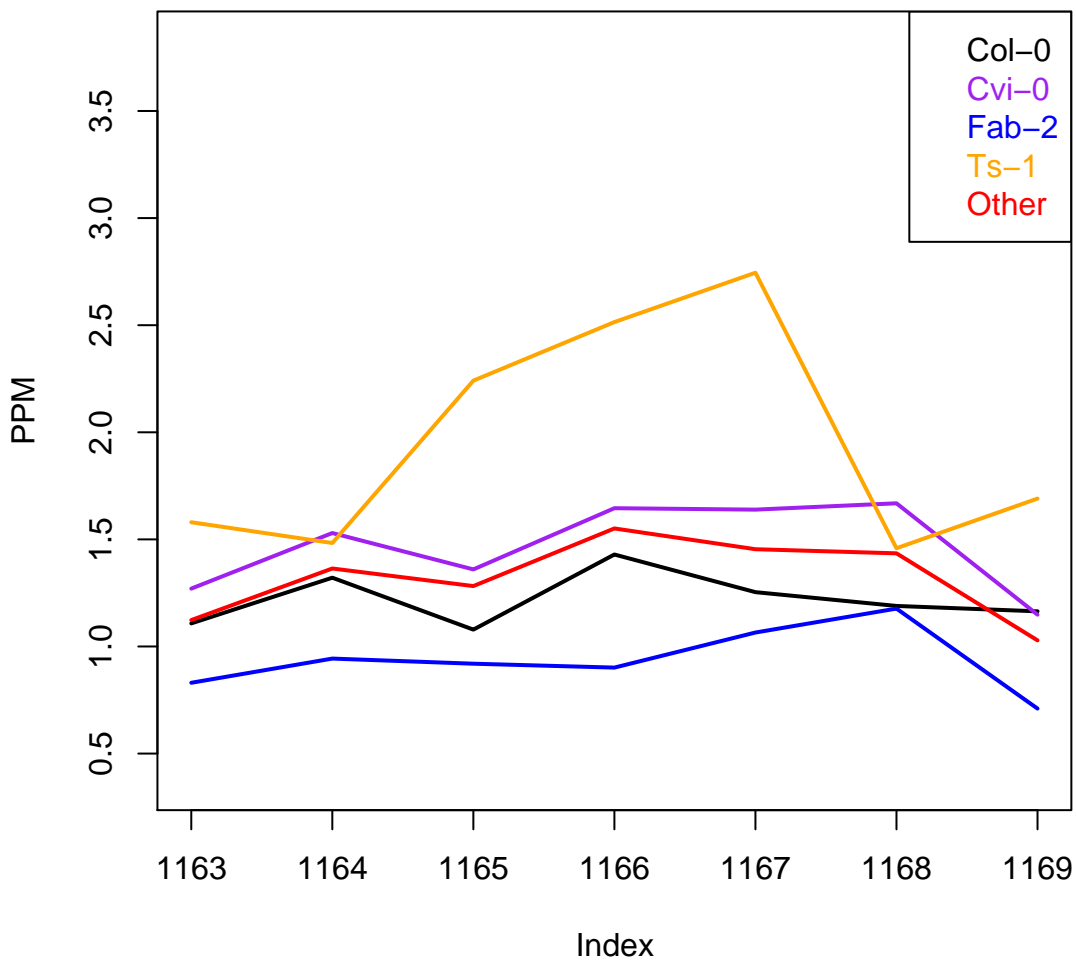

# Ni60

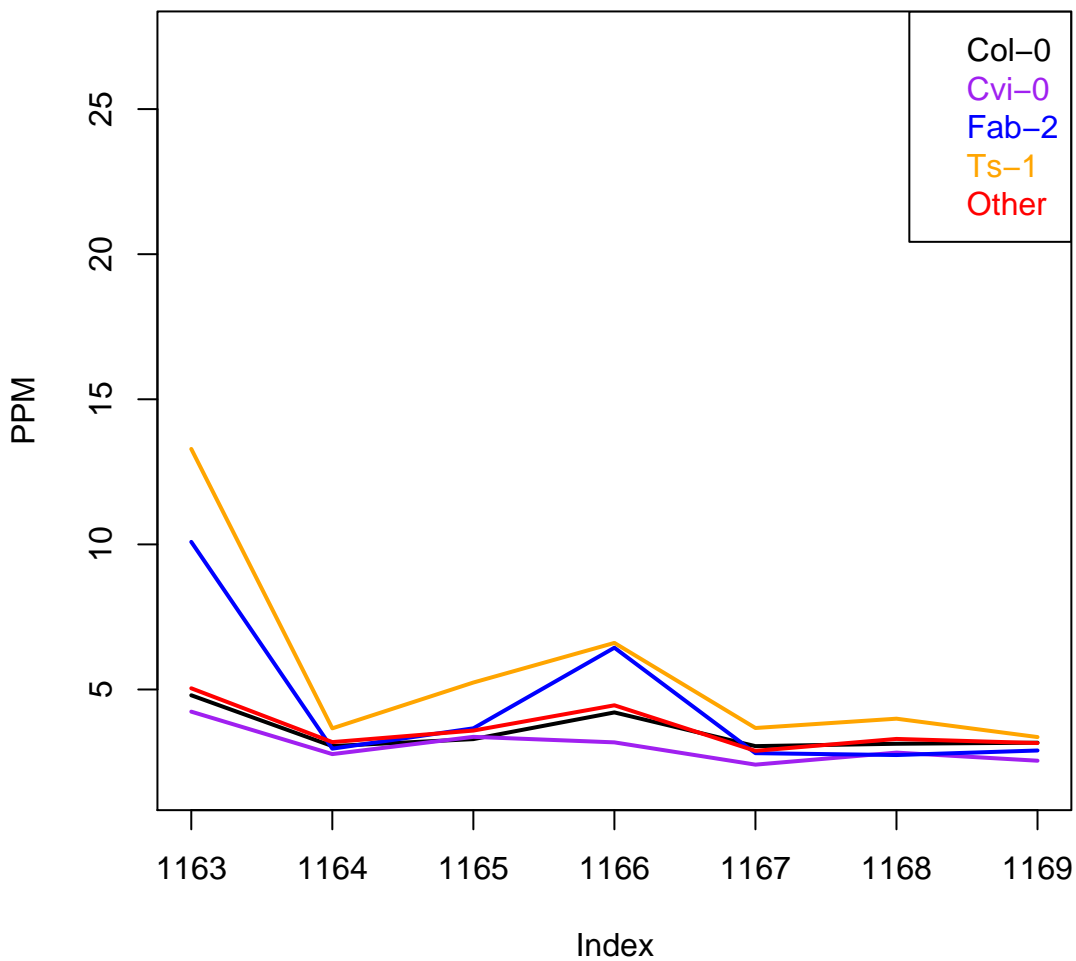

# Cu65

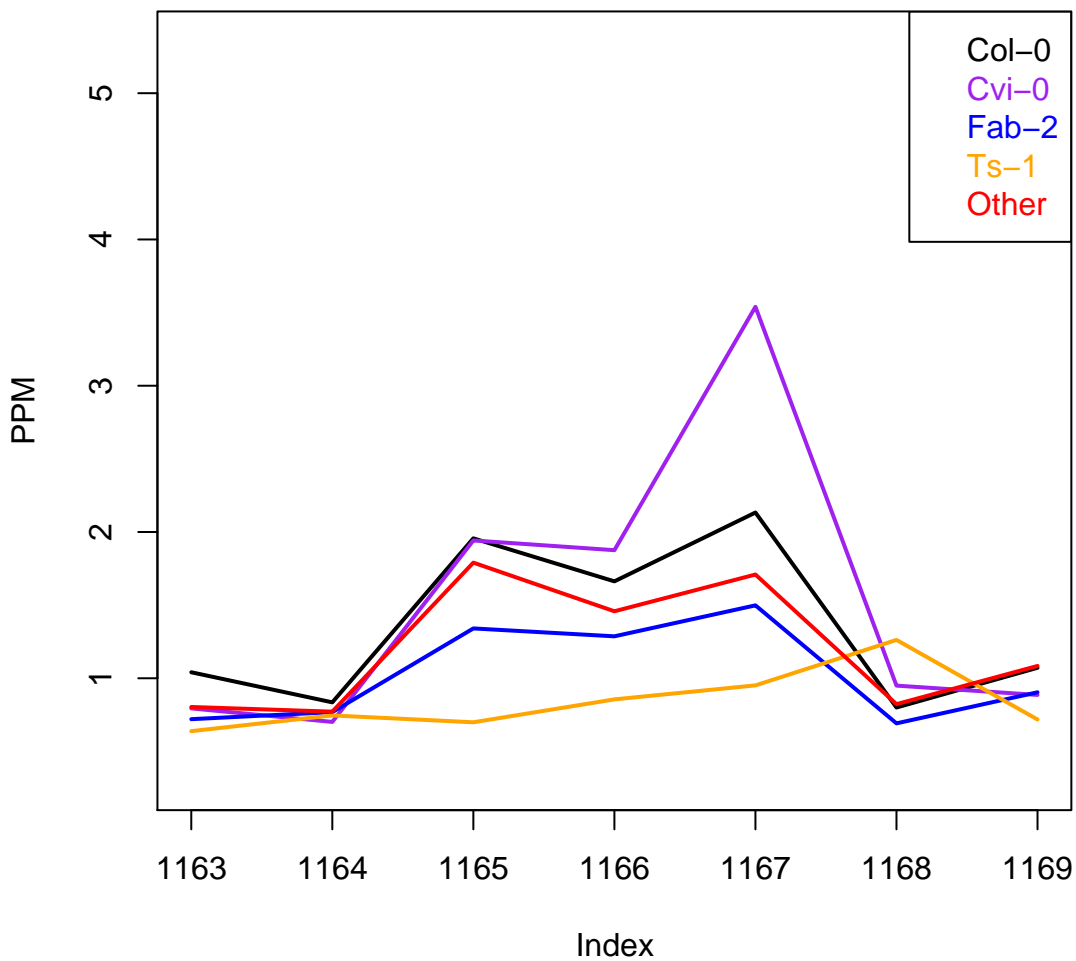

# Zn66

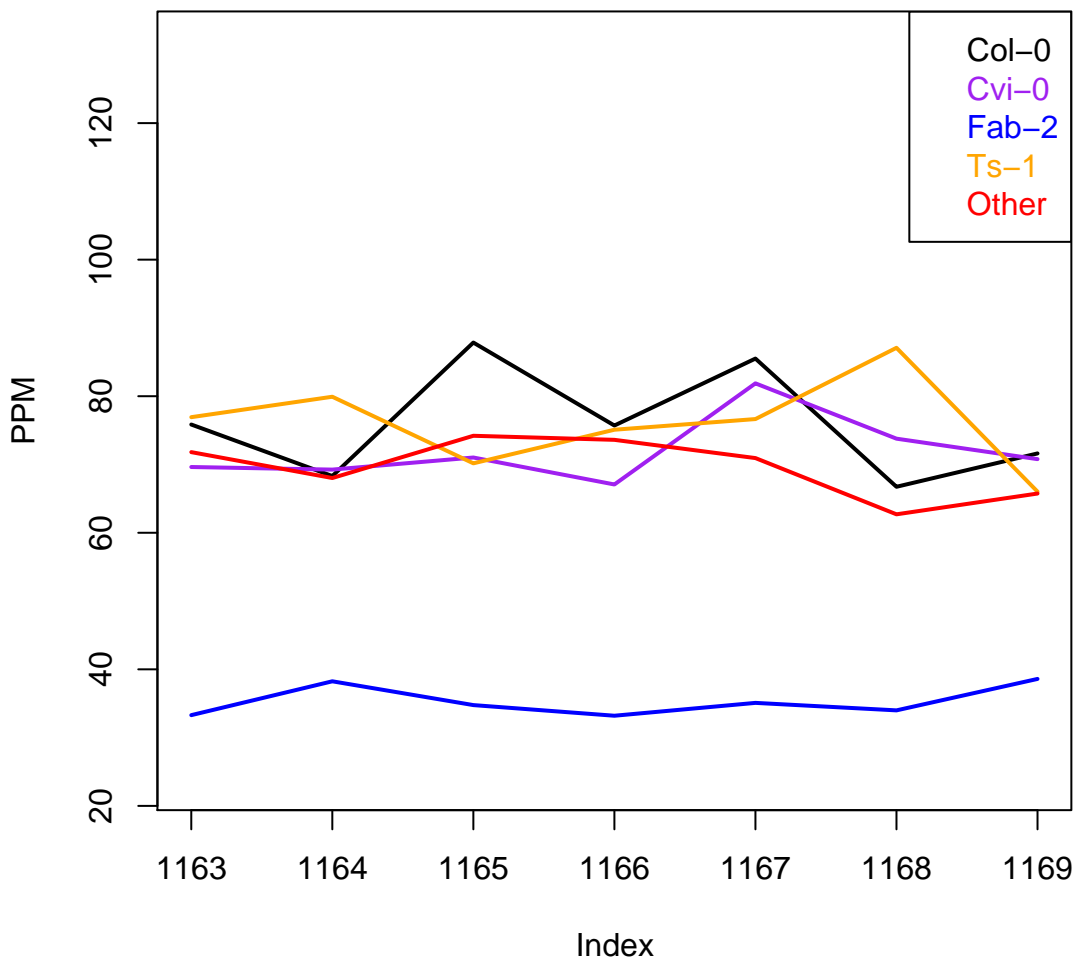

# As75

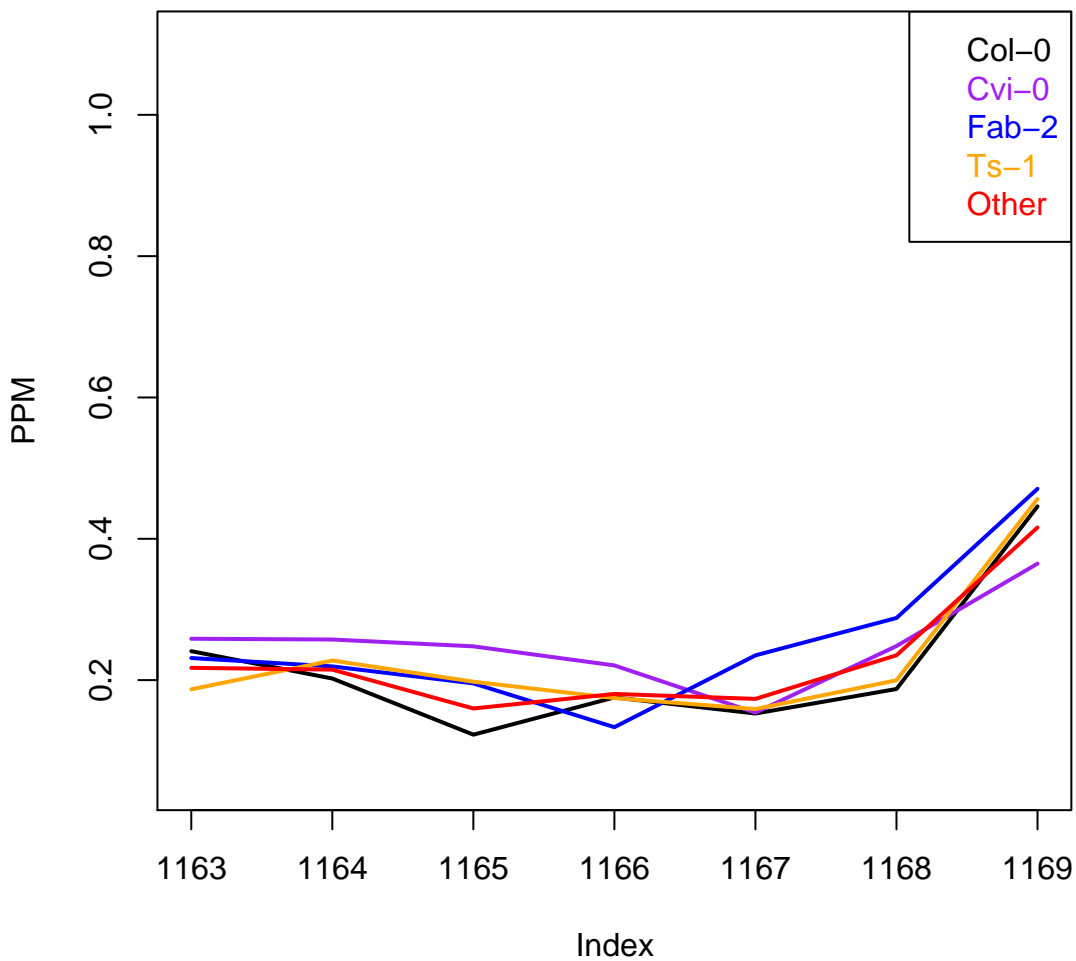

# Se82

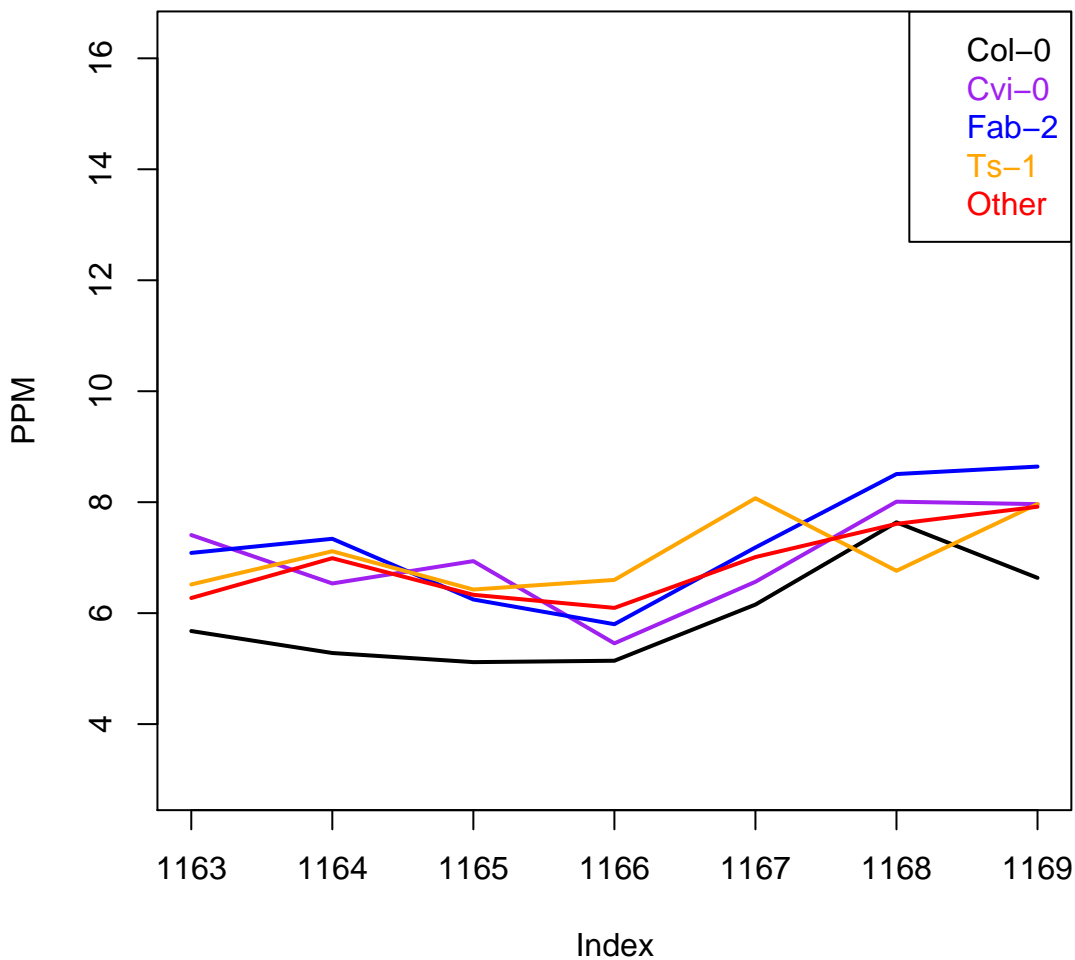

# Mo98

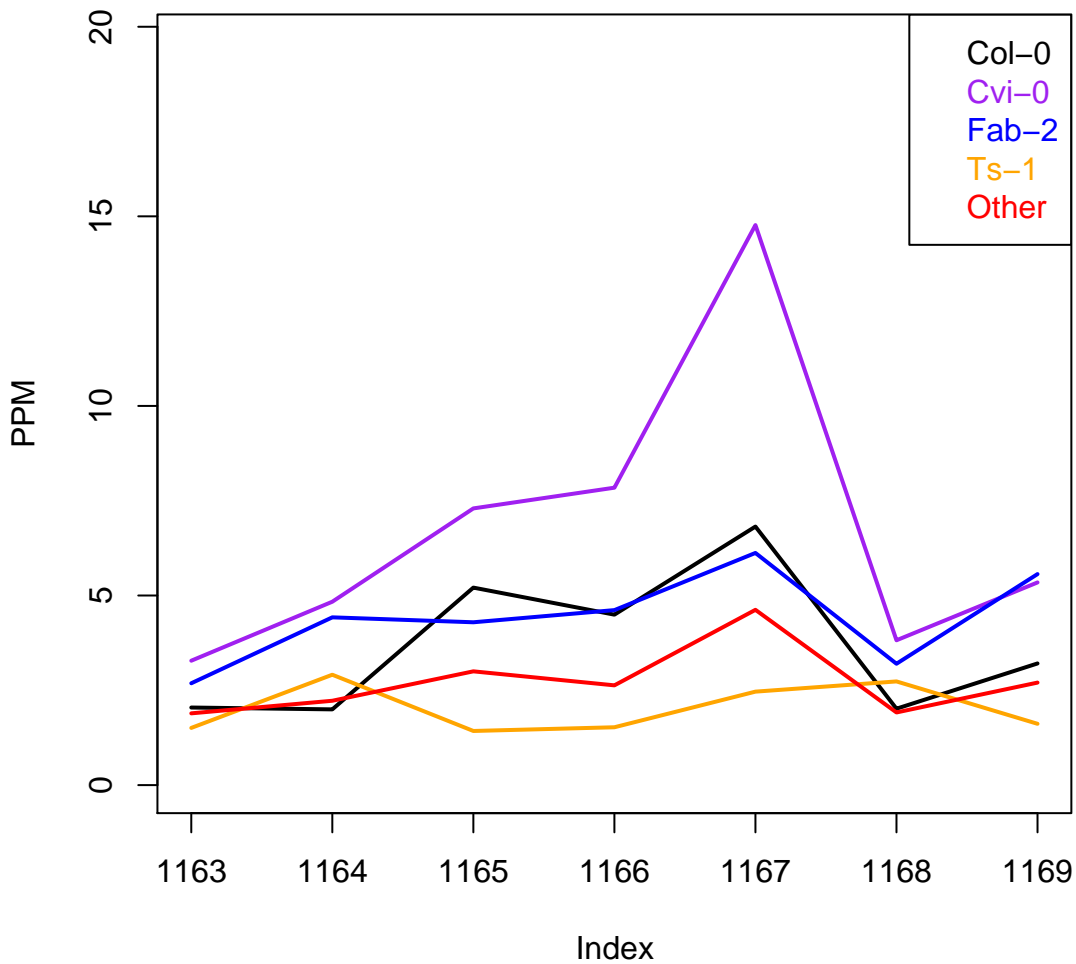

# Cd114

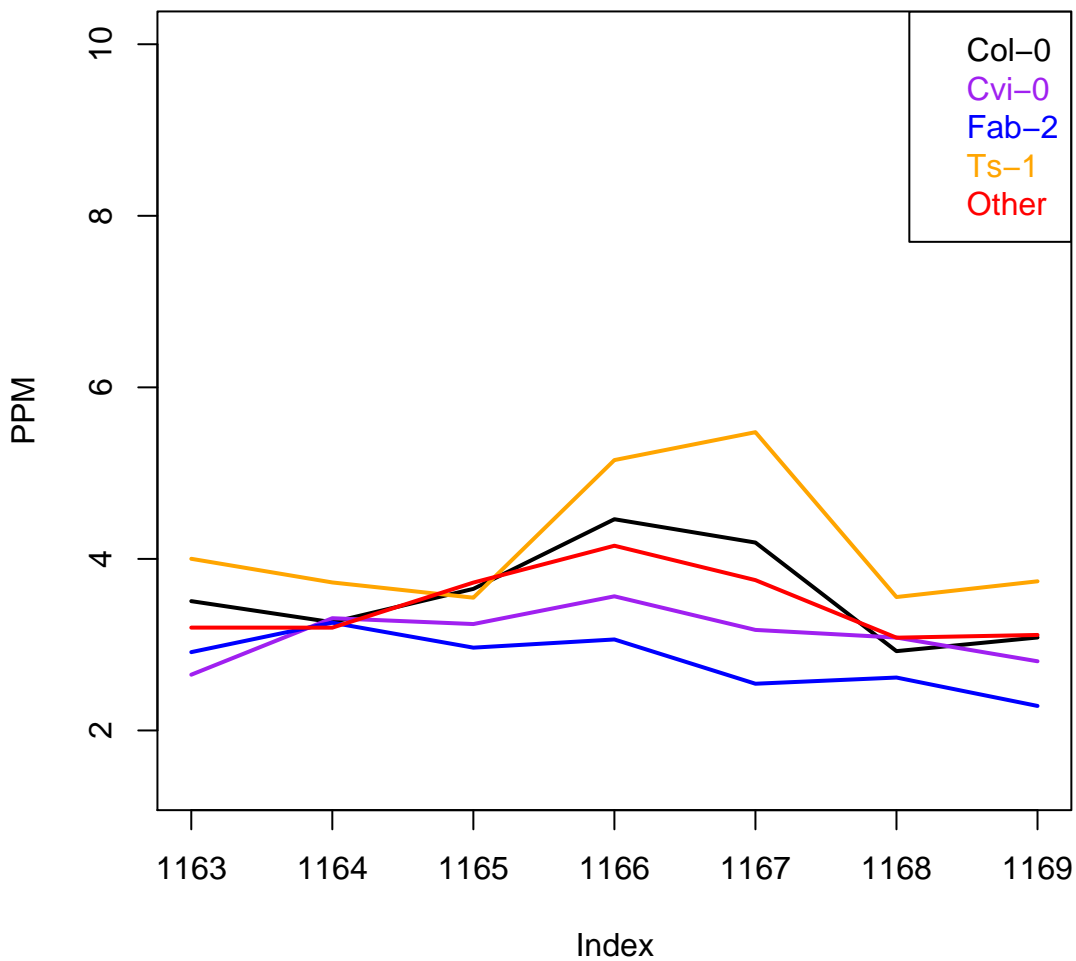

Supplement: Figure S4 — Plot of control line averages for each tray before normalization for Soil Leaf 2 experiment. All non-control lines are averaged into the “Other” line. (PDF) [file pone.0035121.s006.pdf]

# Li7

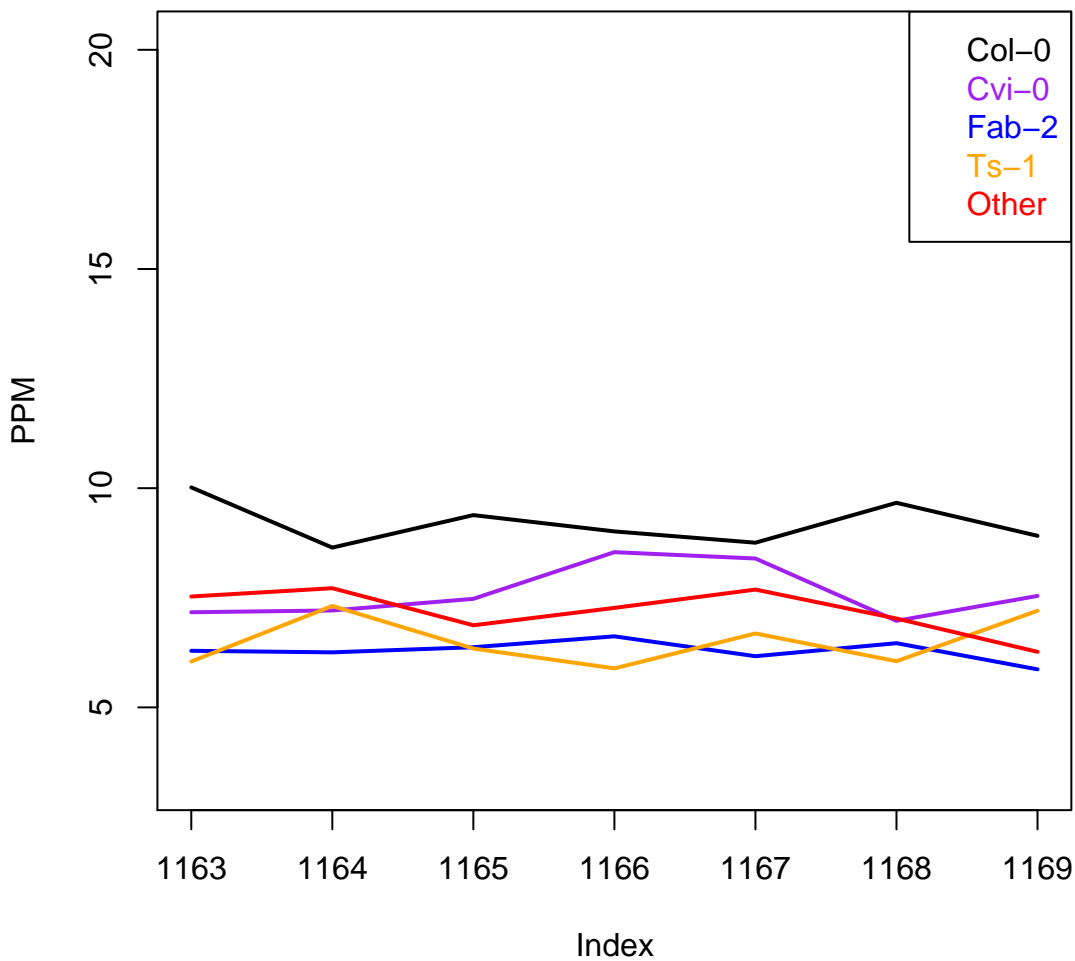

# B11

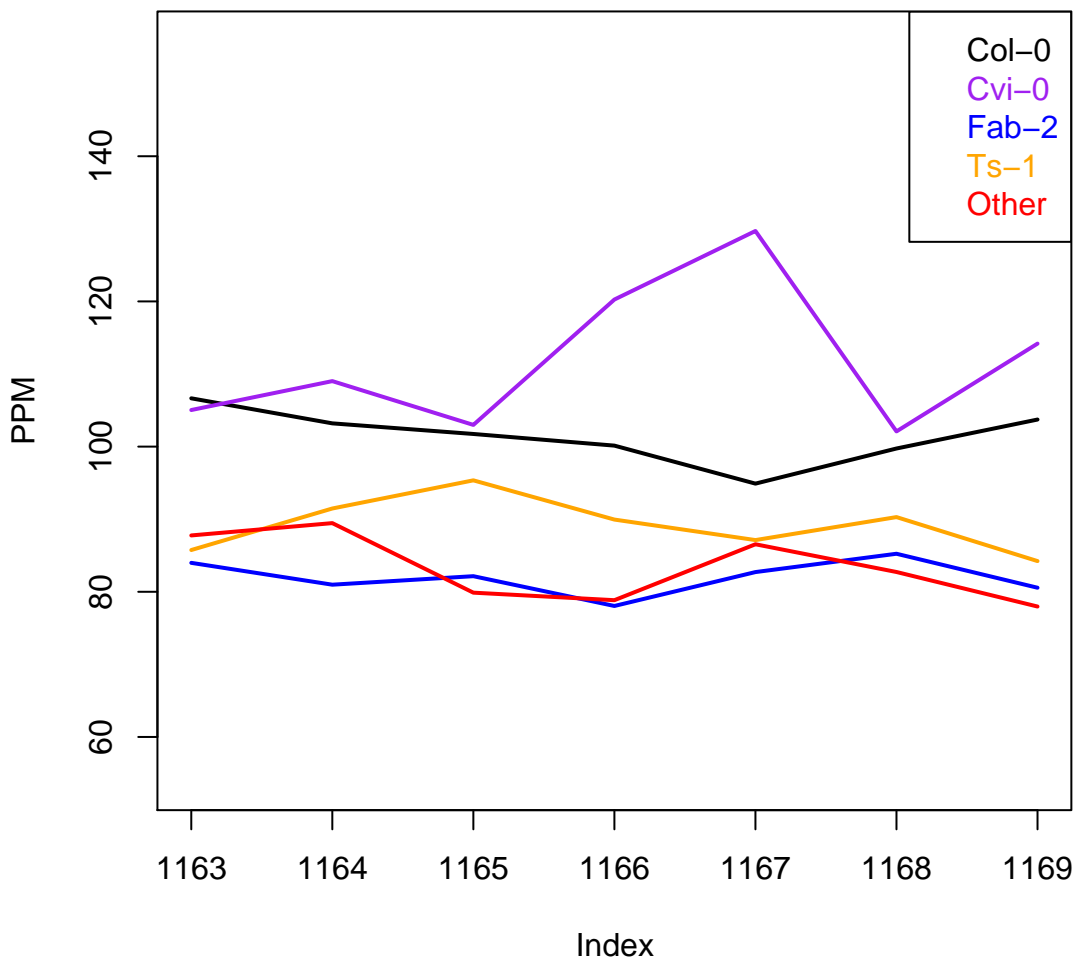

# Na23

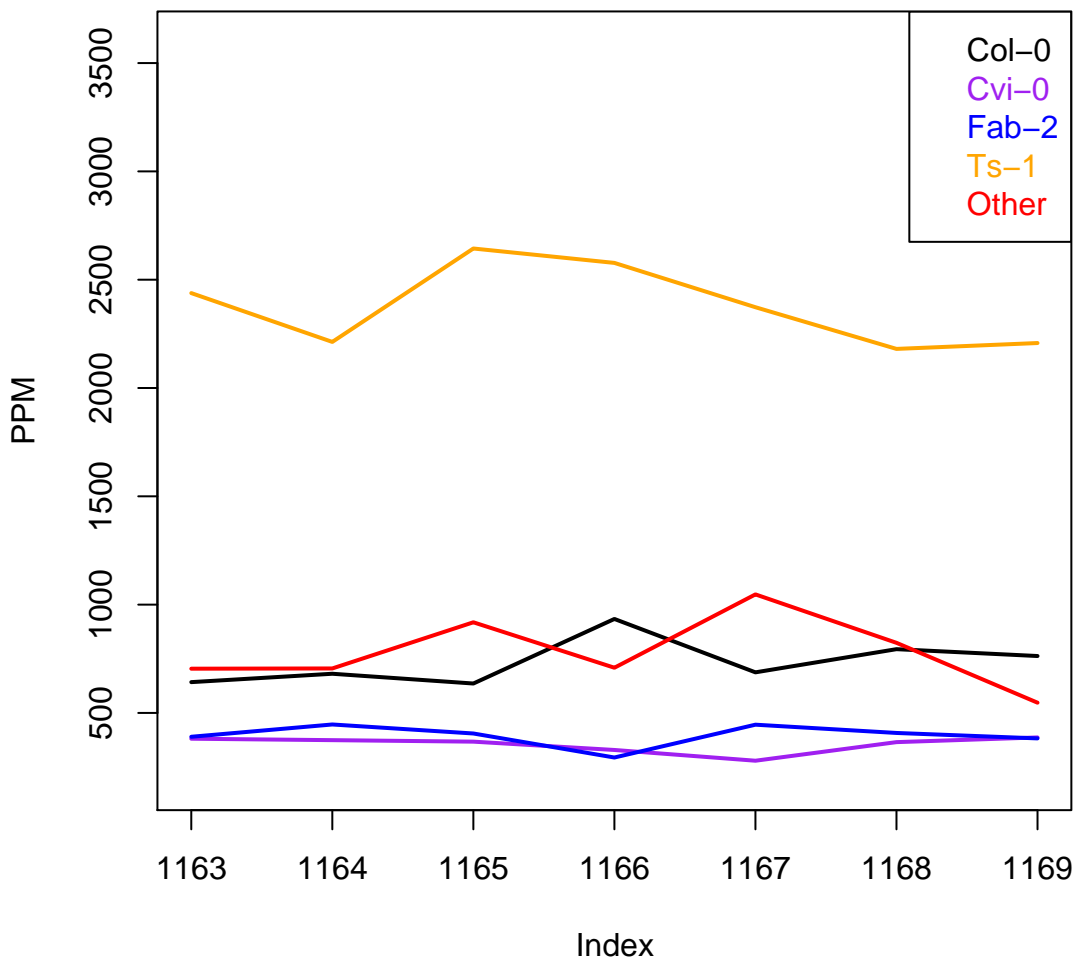

# Mg25

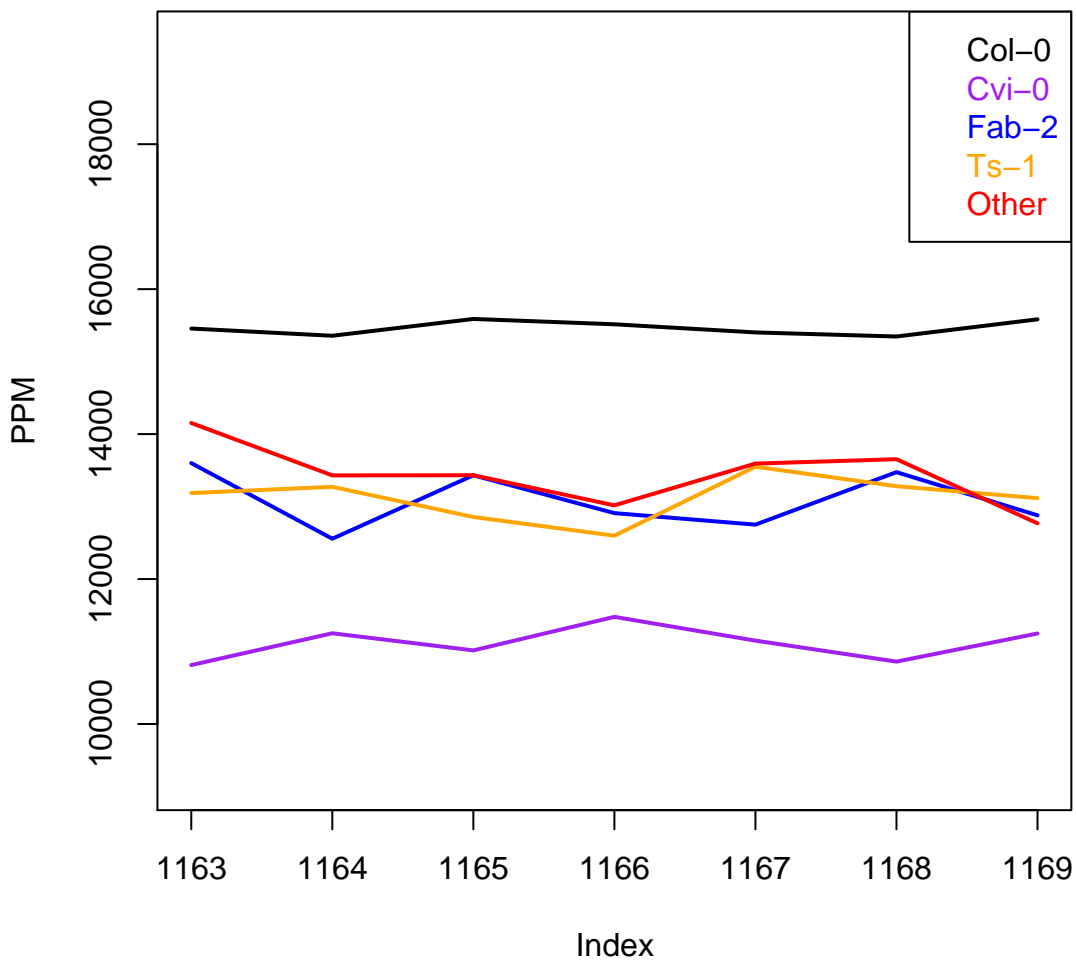

# P31

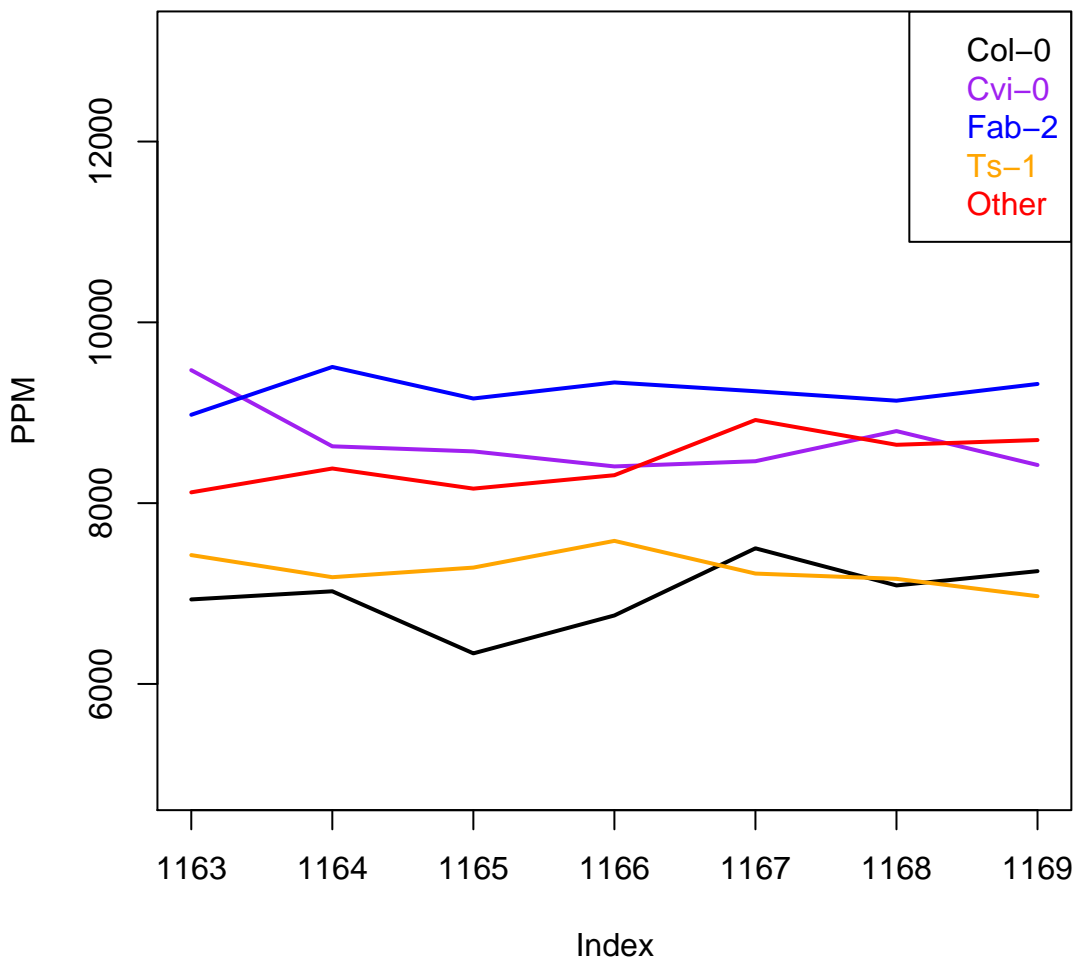

# S34

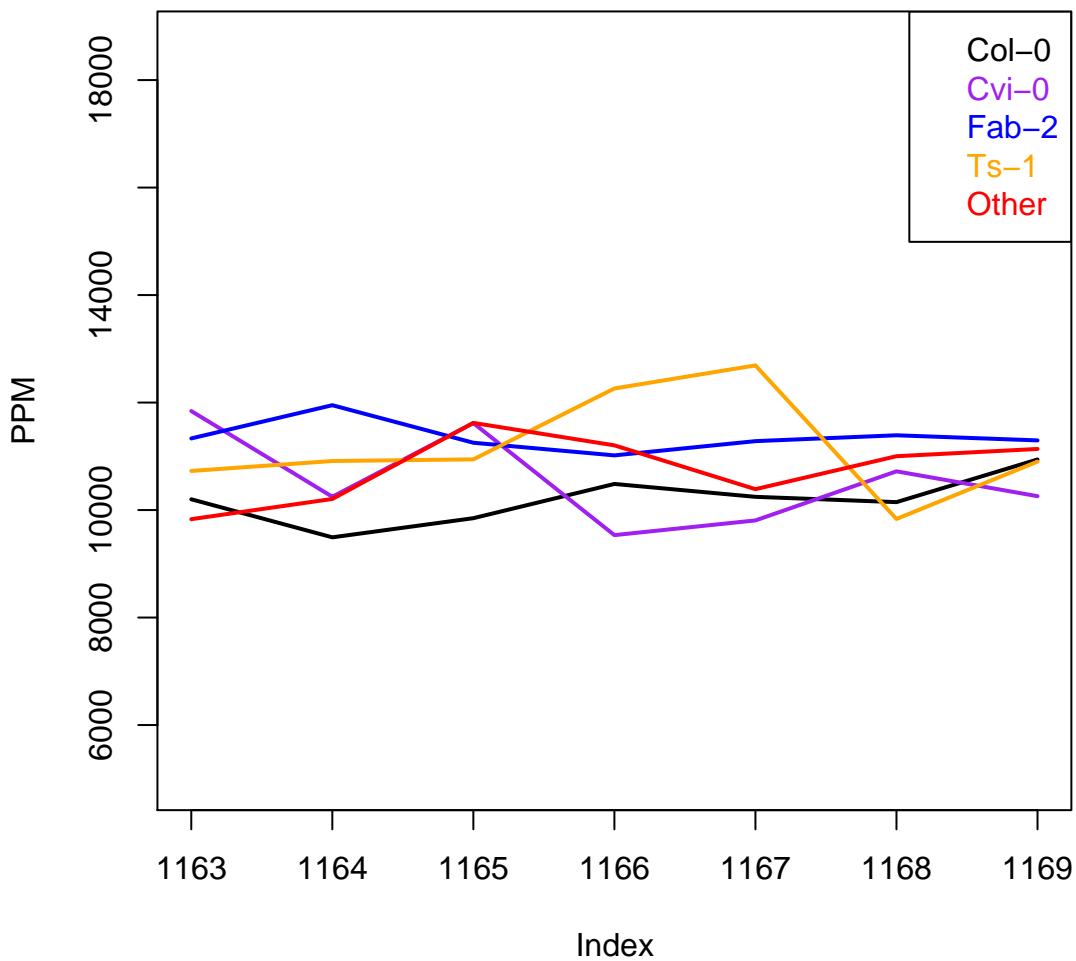

# K39

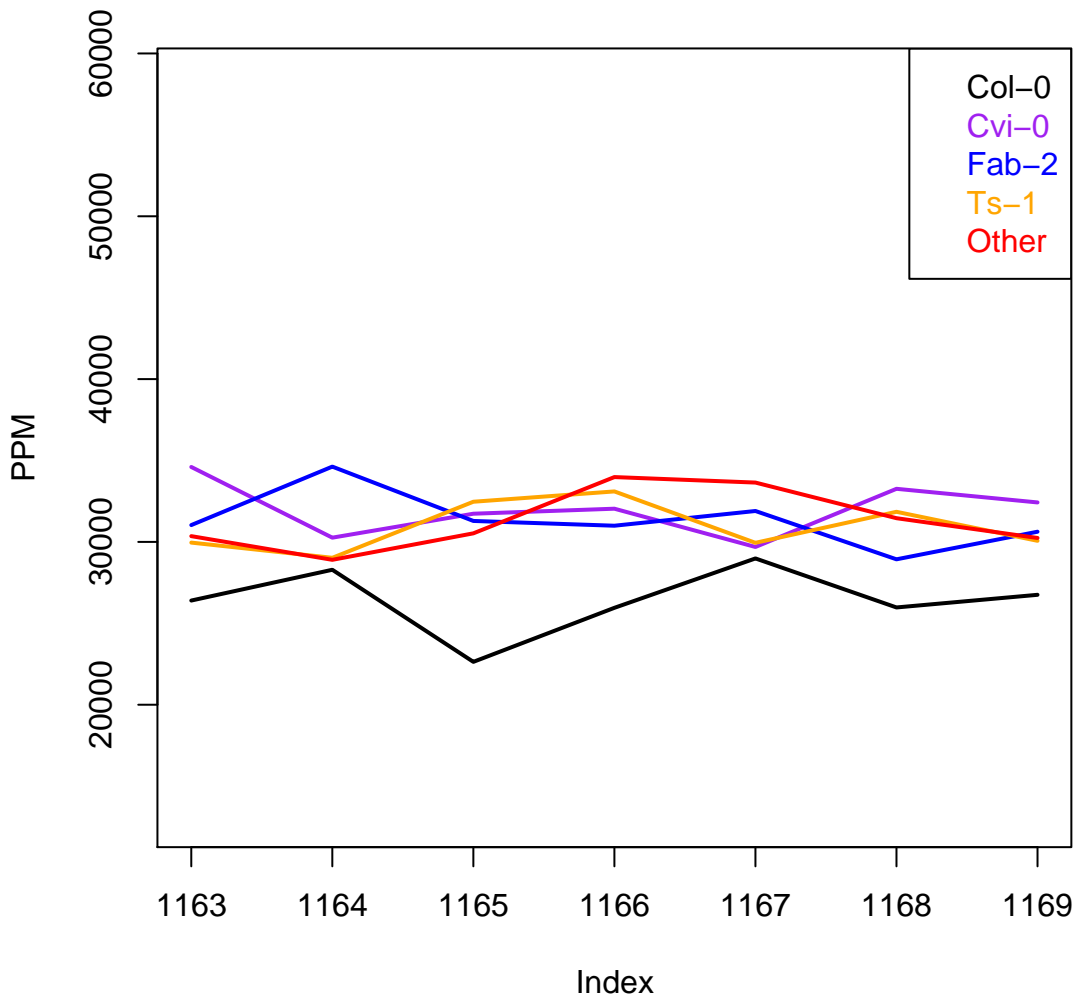

# Ca43

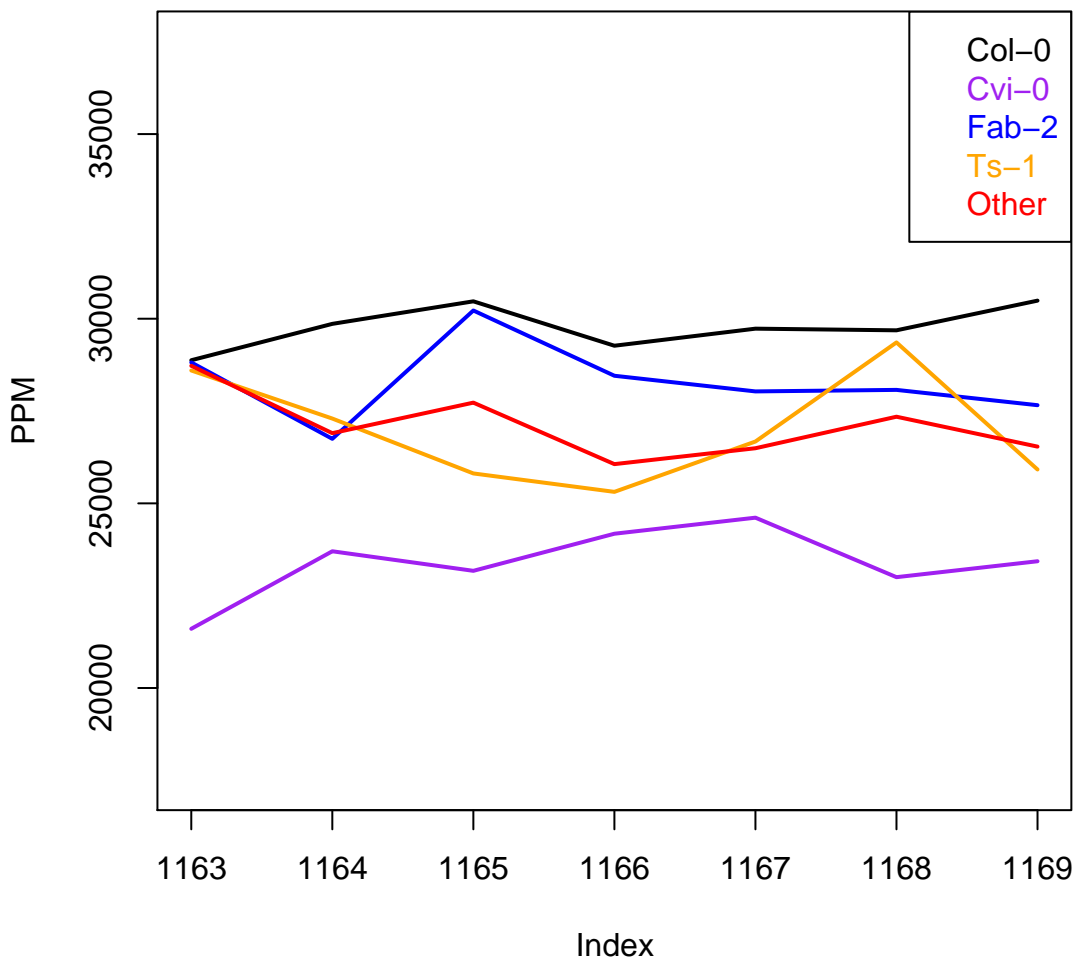

# Mn55

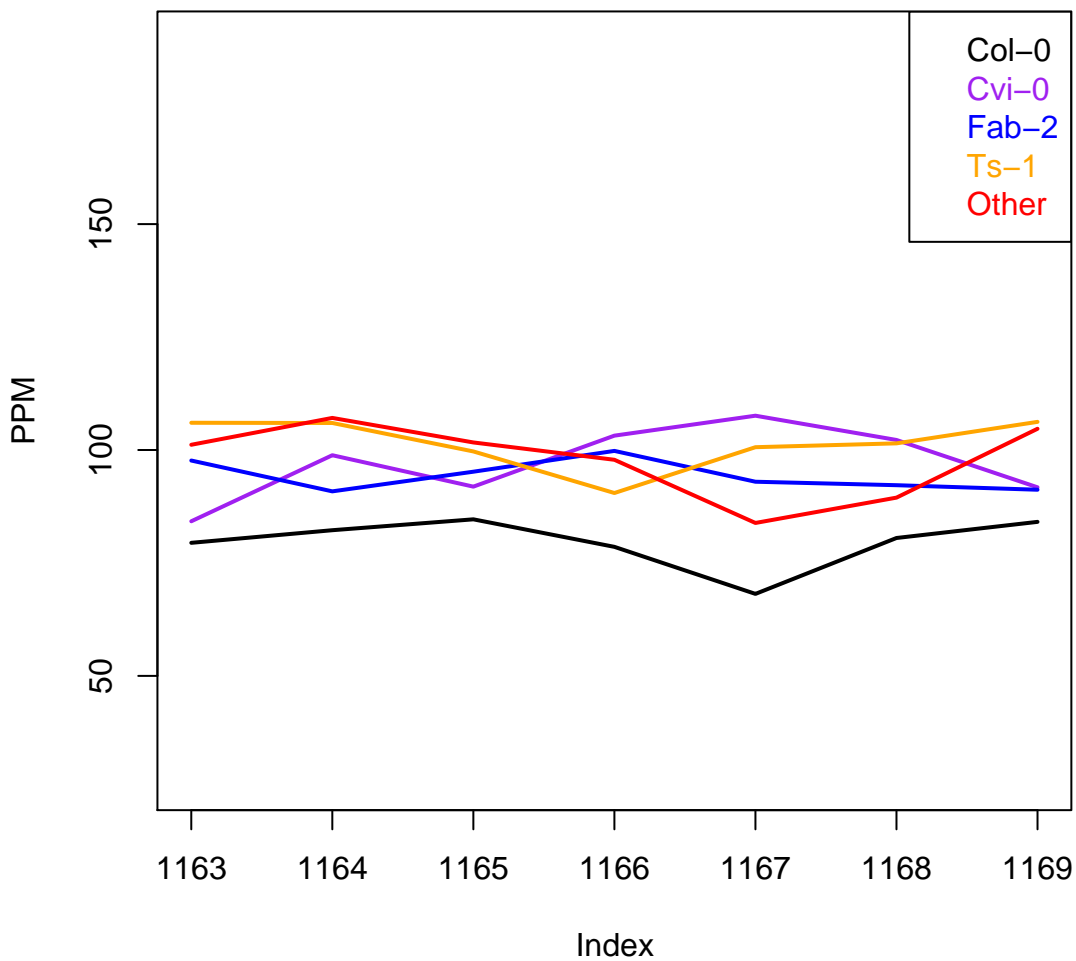

# Fe56

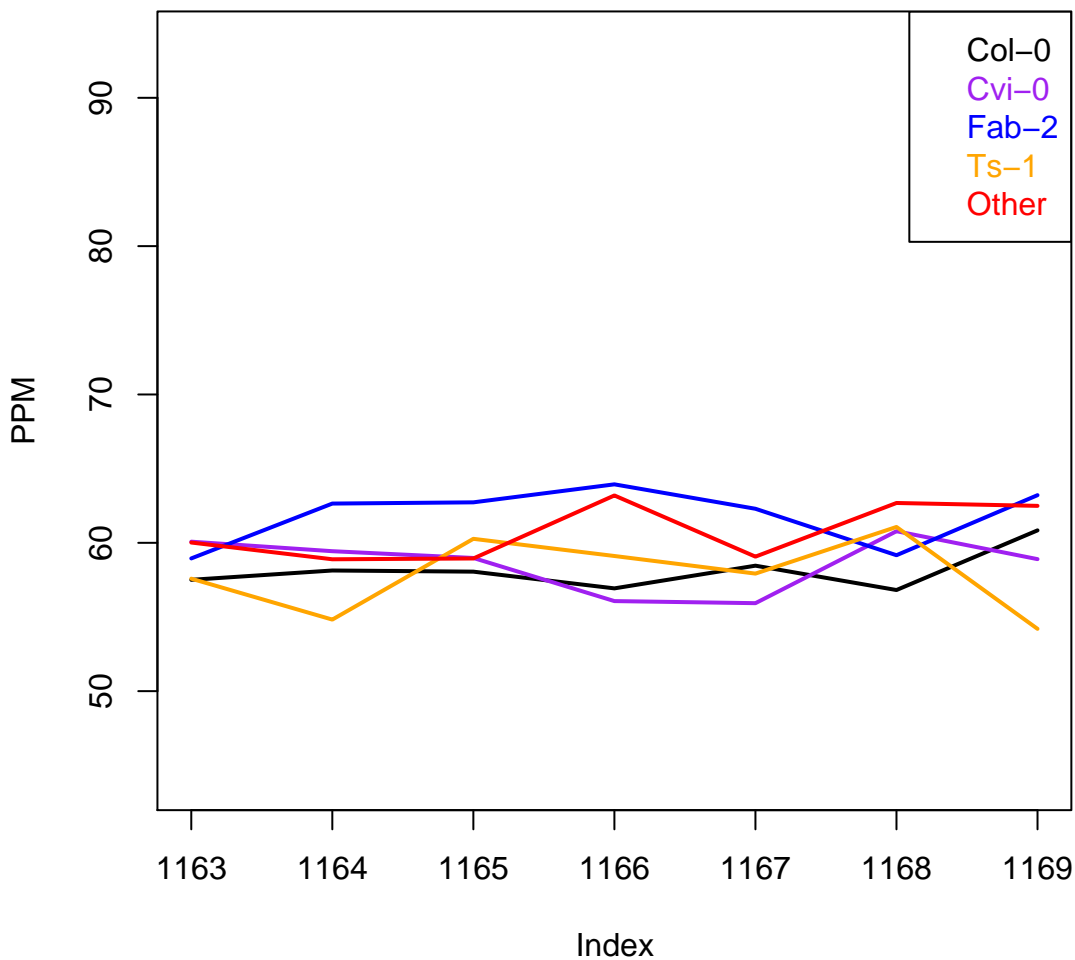

# Co59

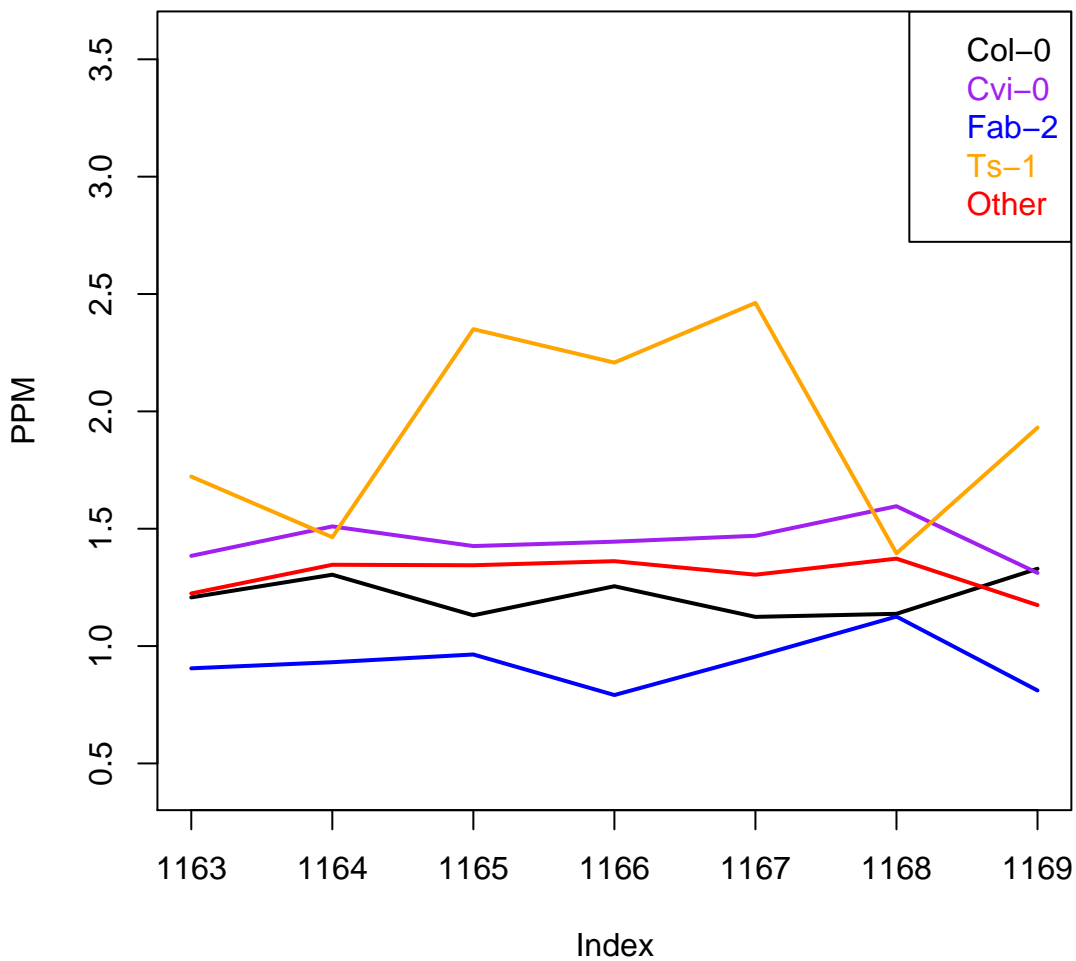

# Ni60

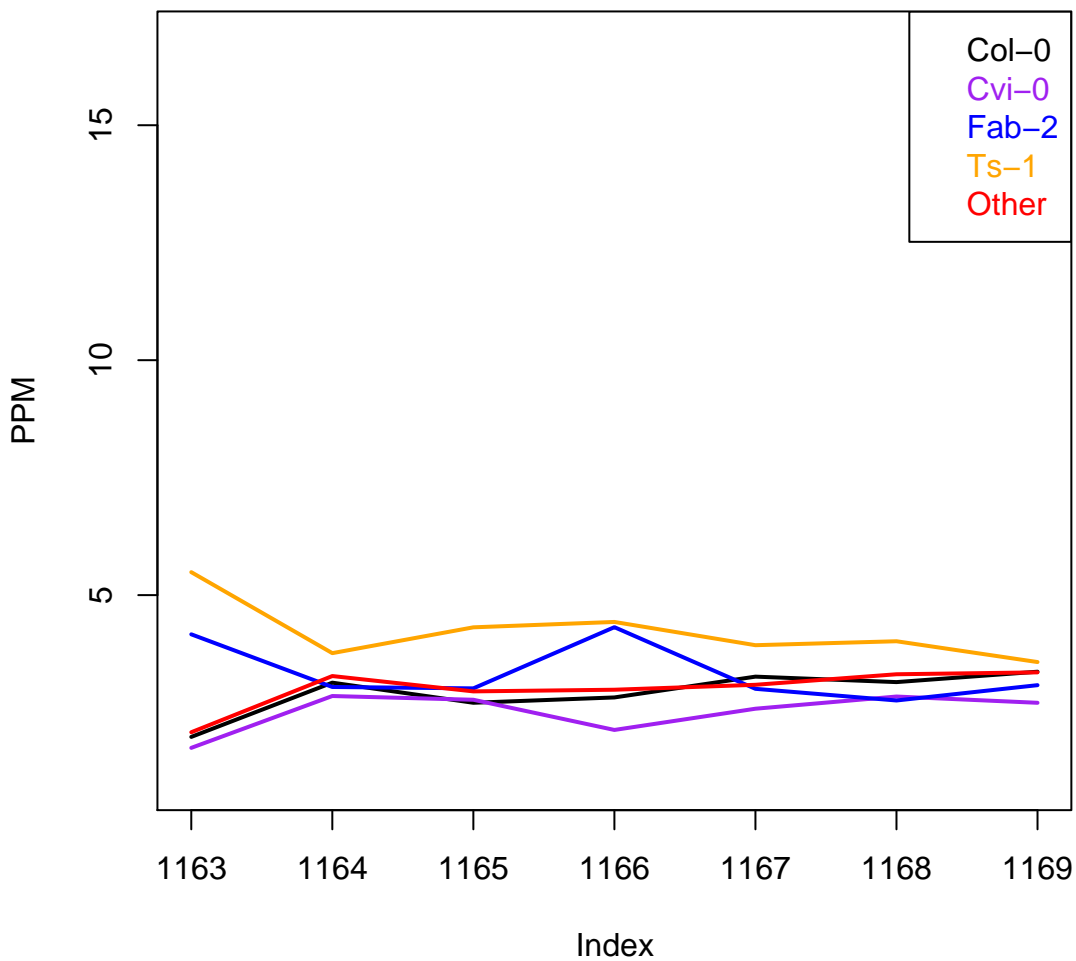

# Cu65

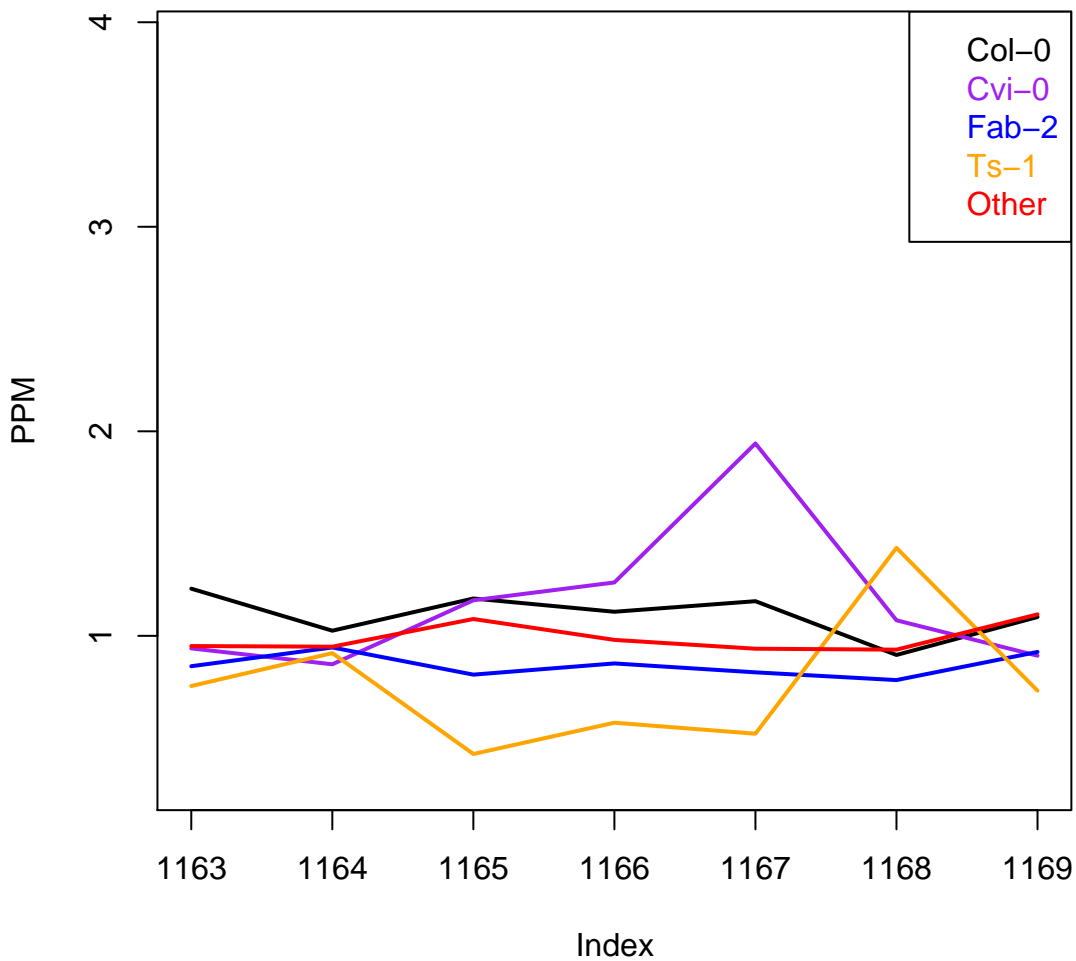

# Zn66

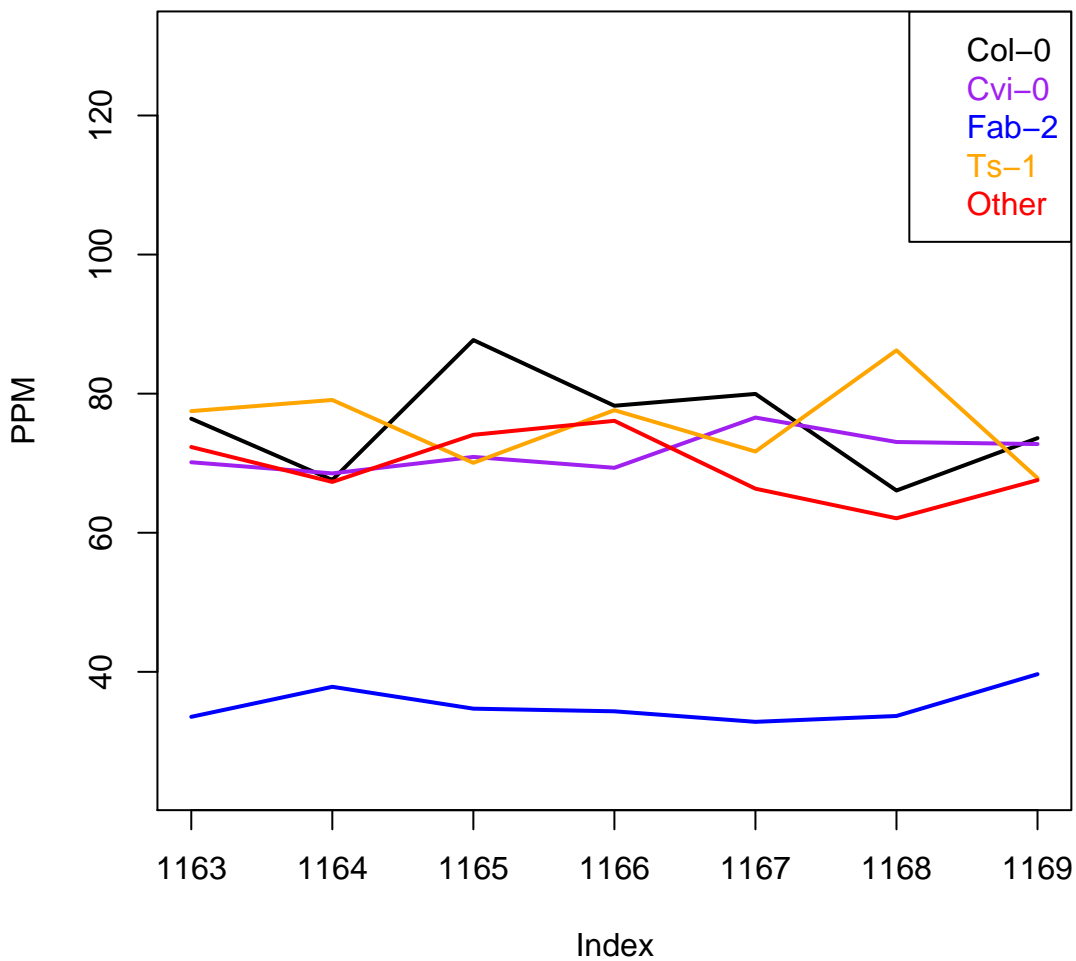

# As75

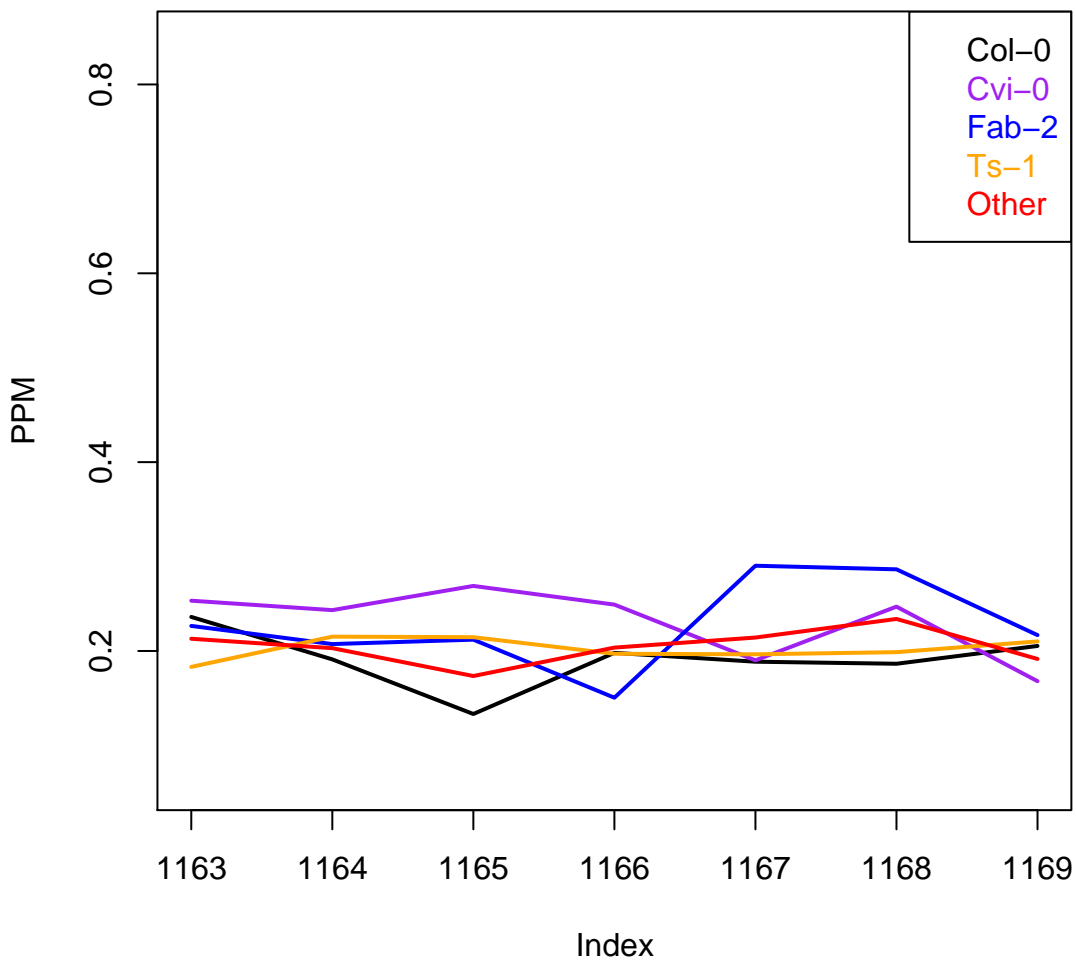

# Se82

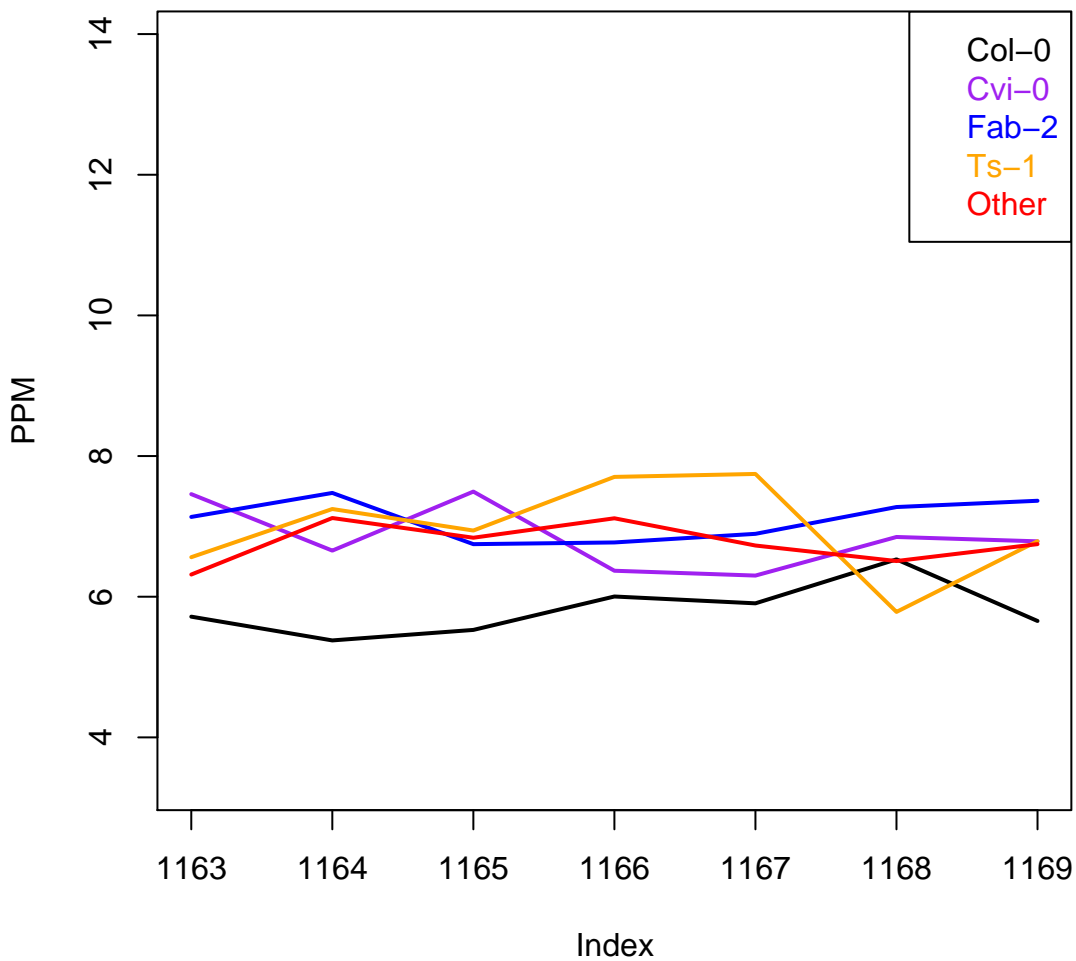

# Mo98

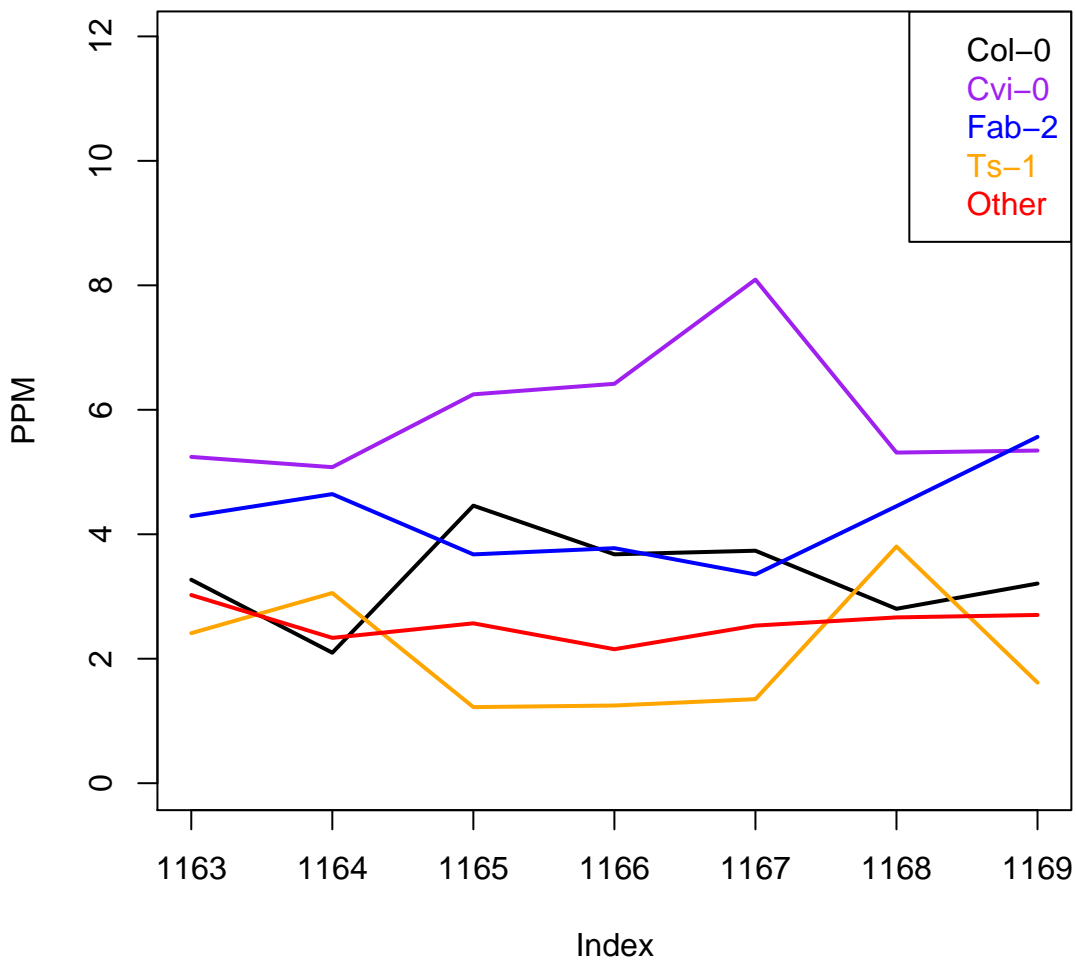

# Cd114

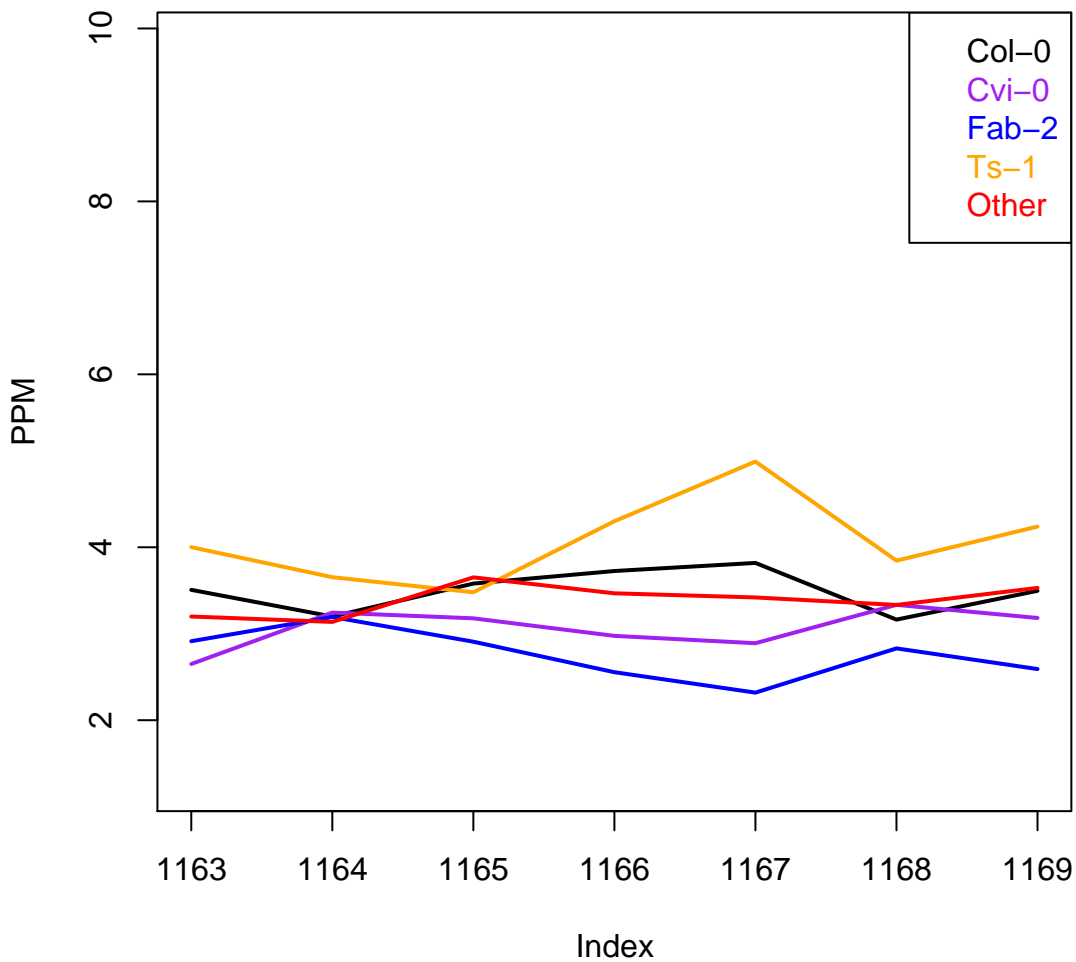

Supplement: Figure S5 — Plot of control line averages for each tray after normalization for Soil Leaf 2 experiment. All non-control lines are averaged into the “Other” line. (PDF) [file pone.0035121.s007.pdf]

# Li7

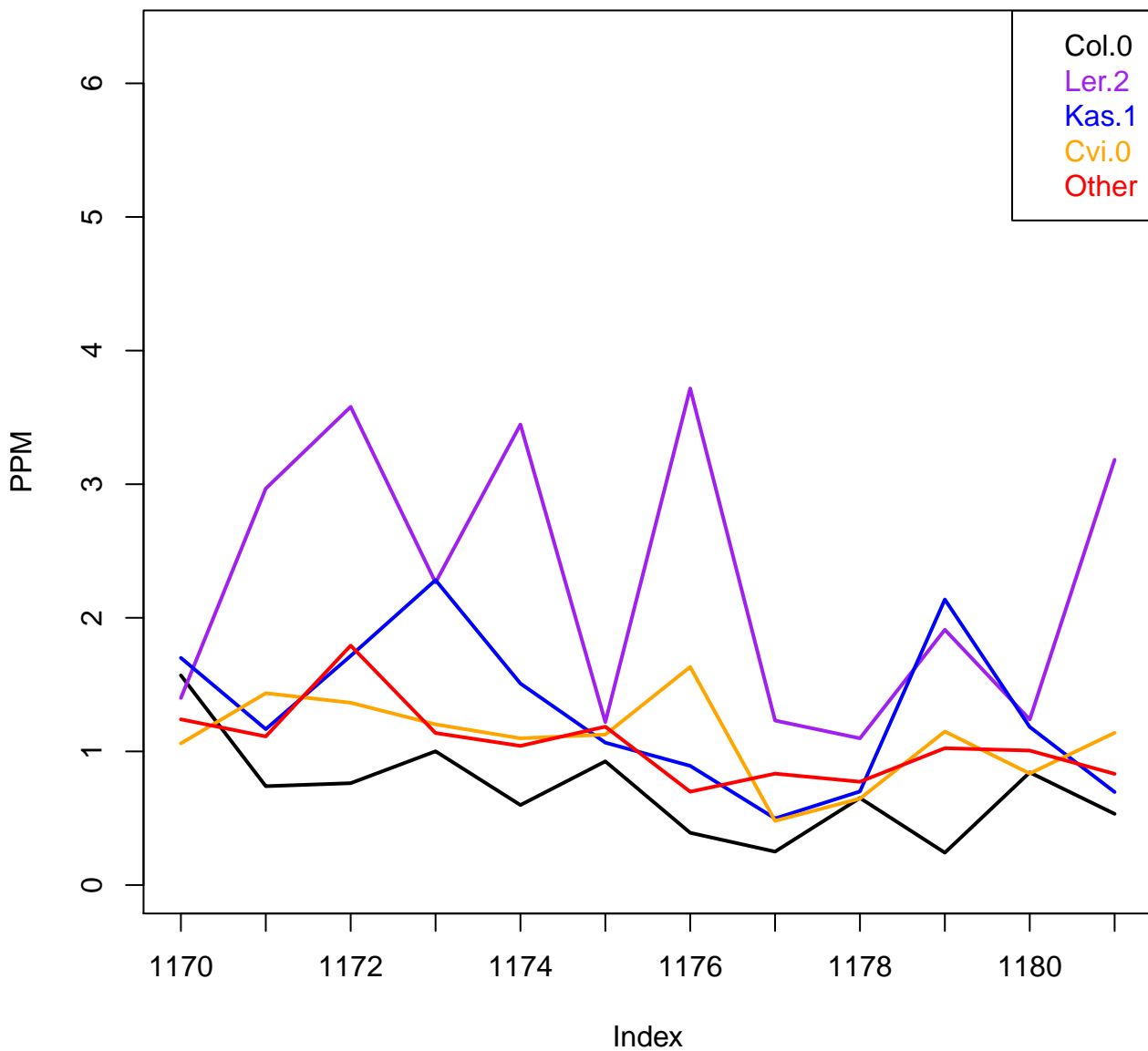

# B11

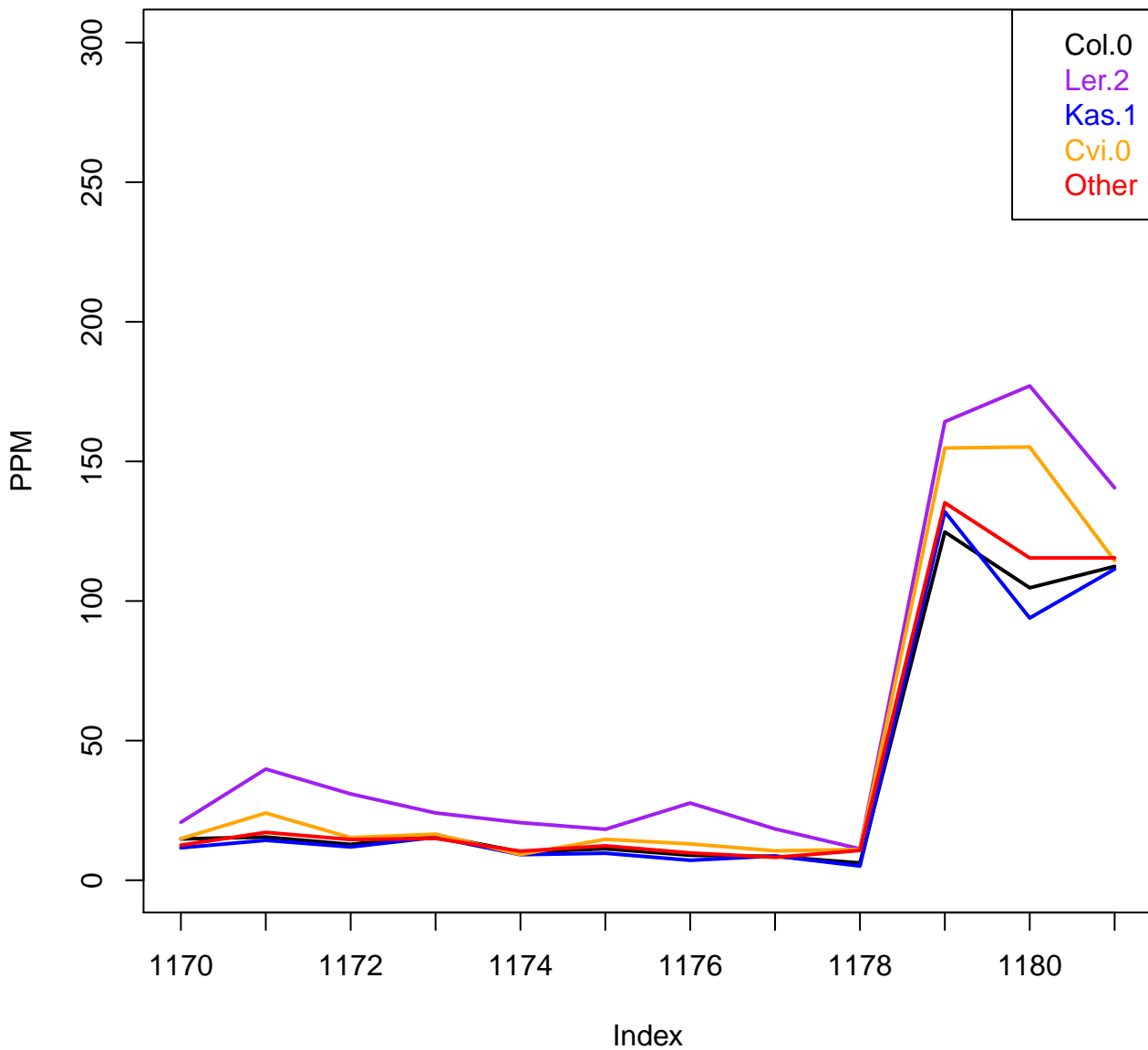

# Na23

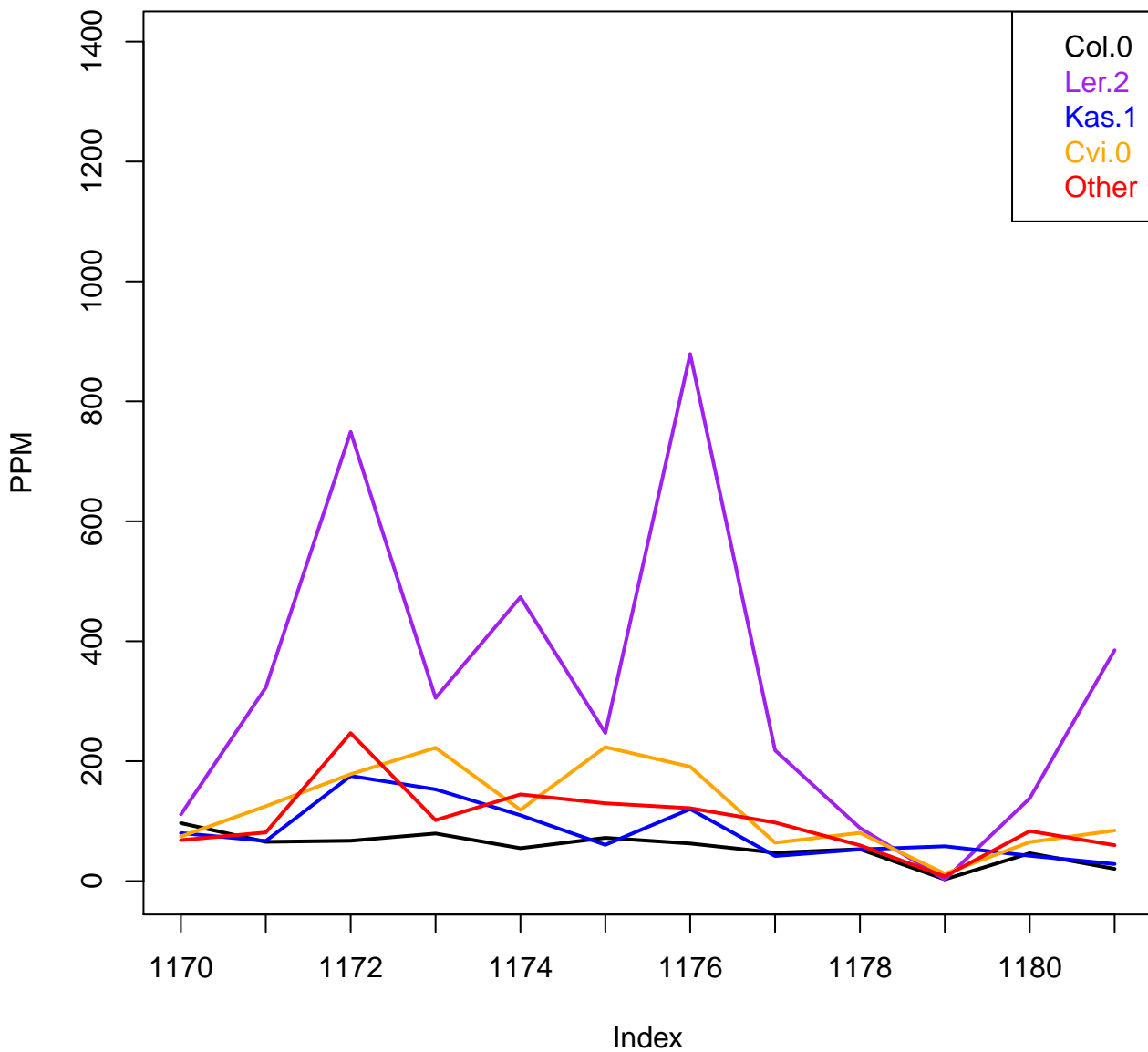

# Mg25

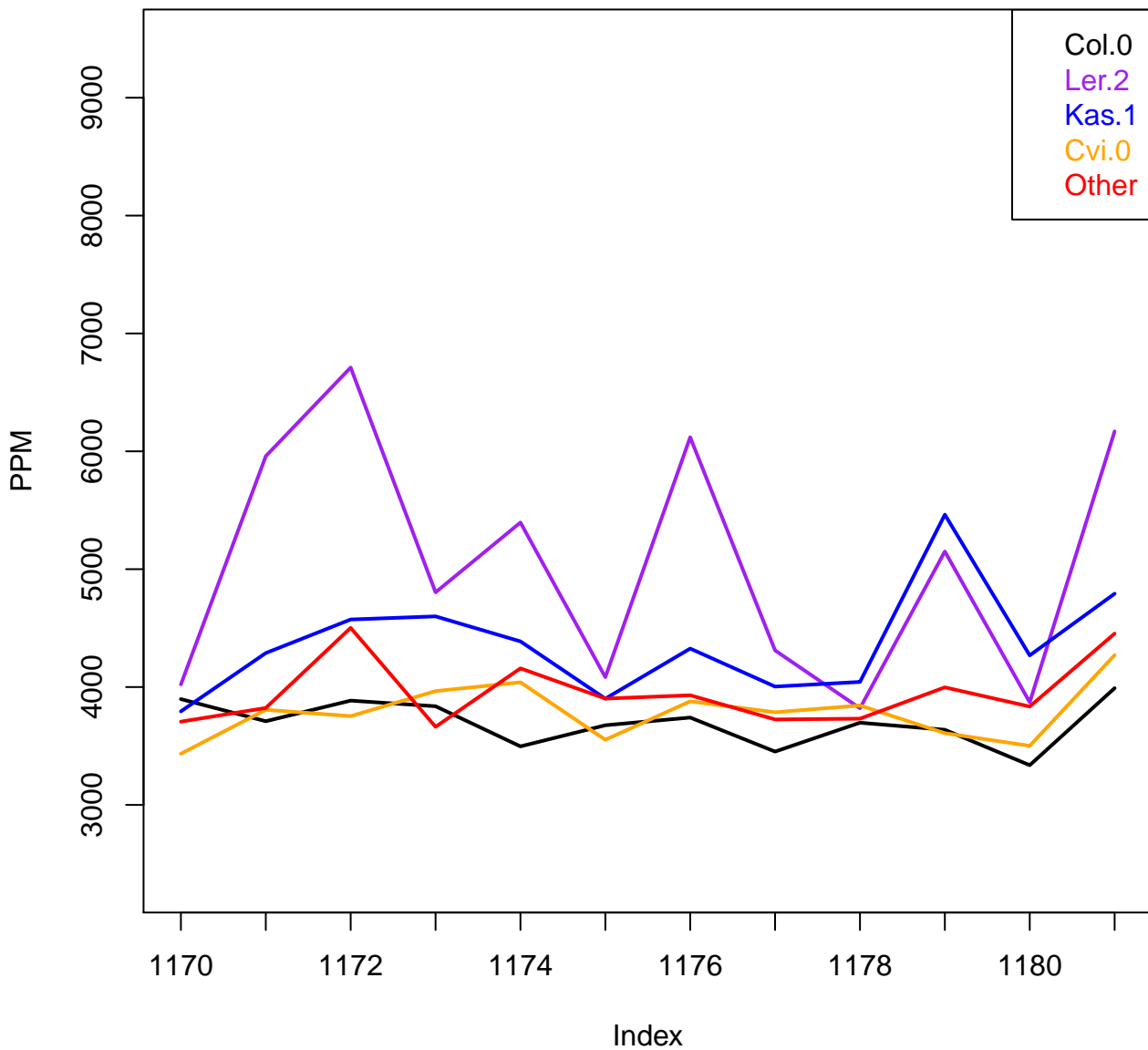

# P31

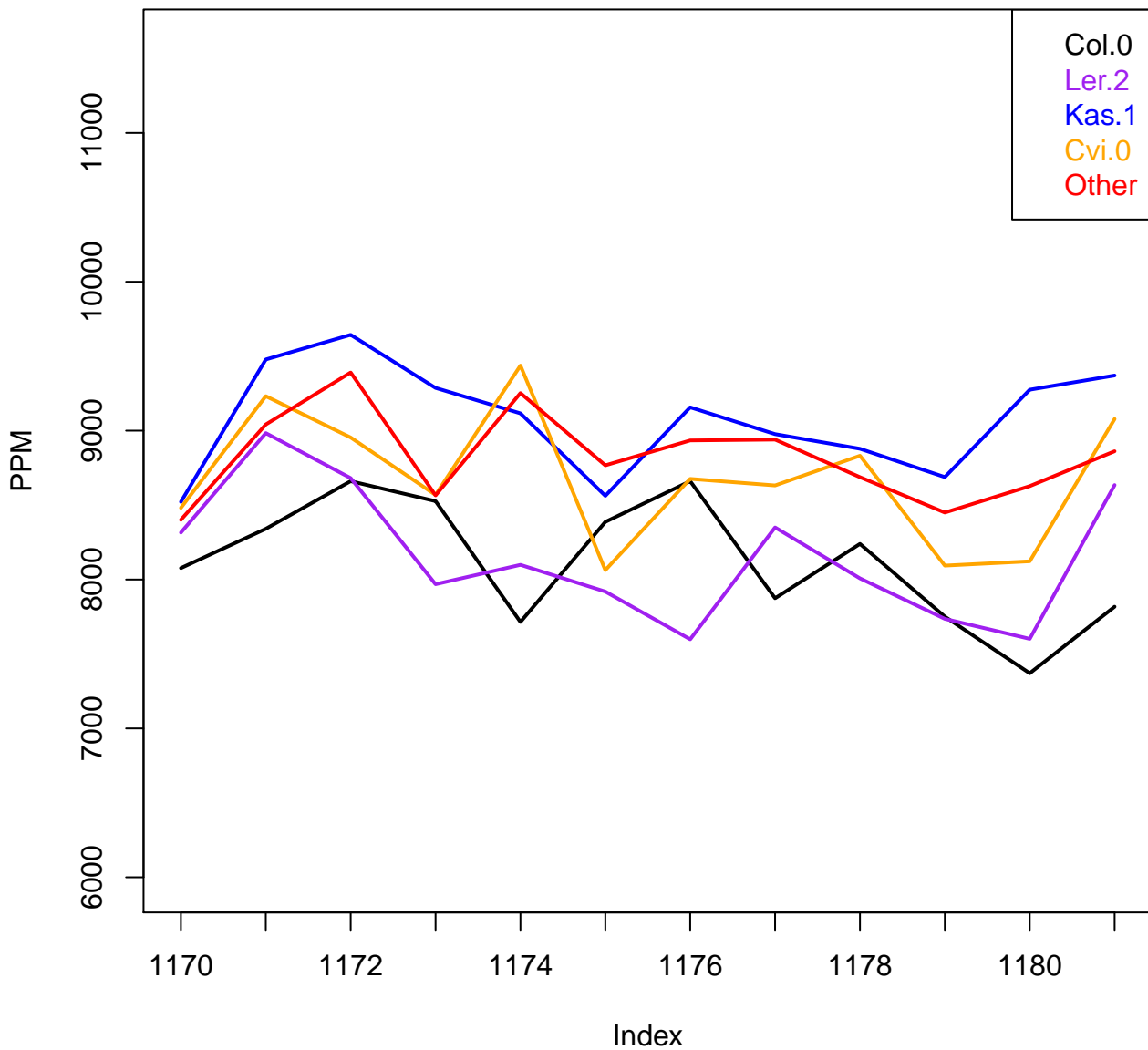

# S34

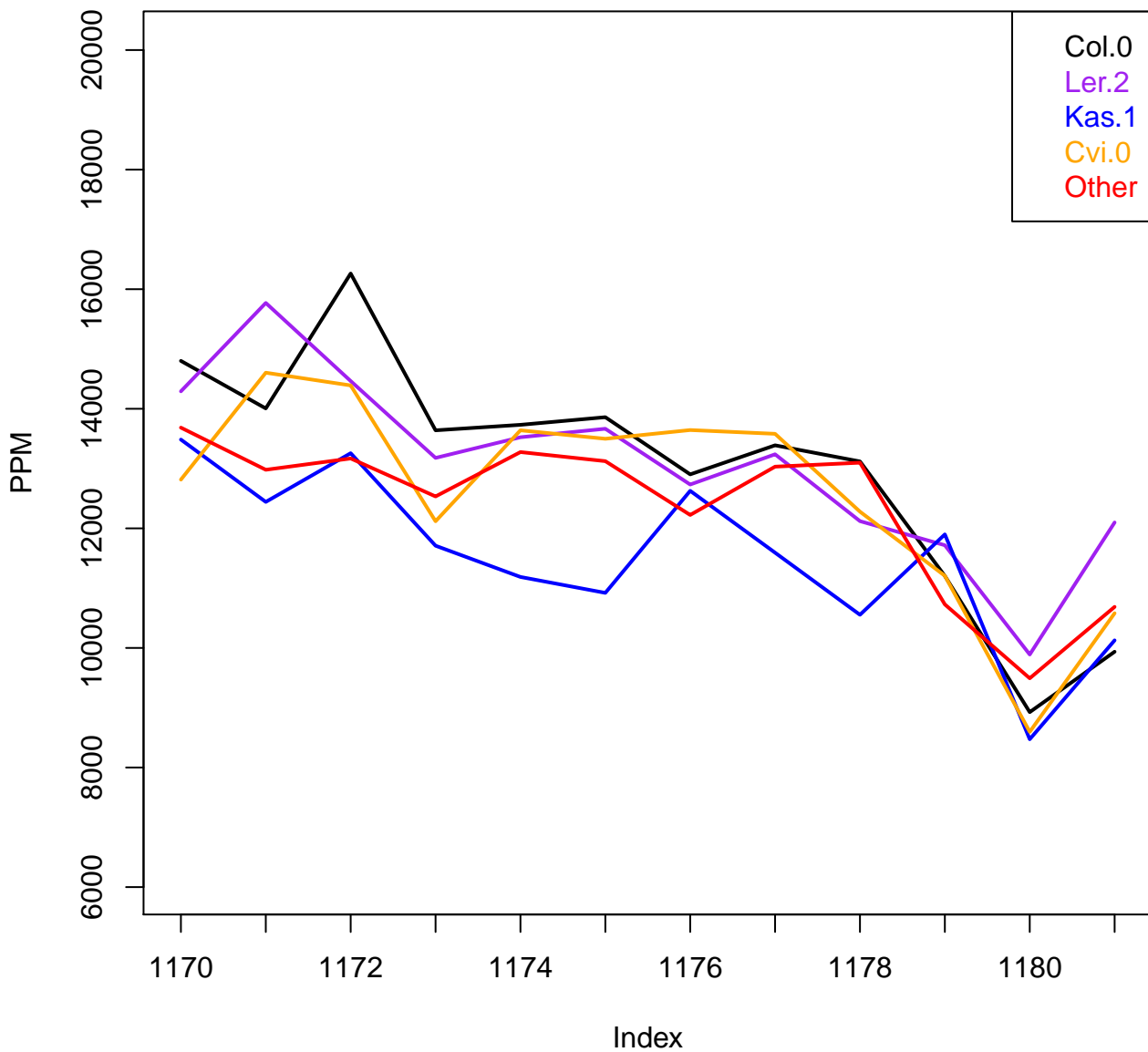

# K39

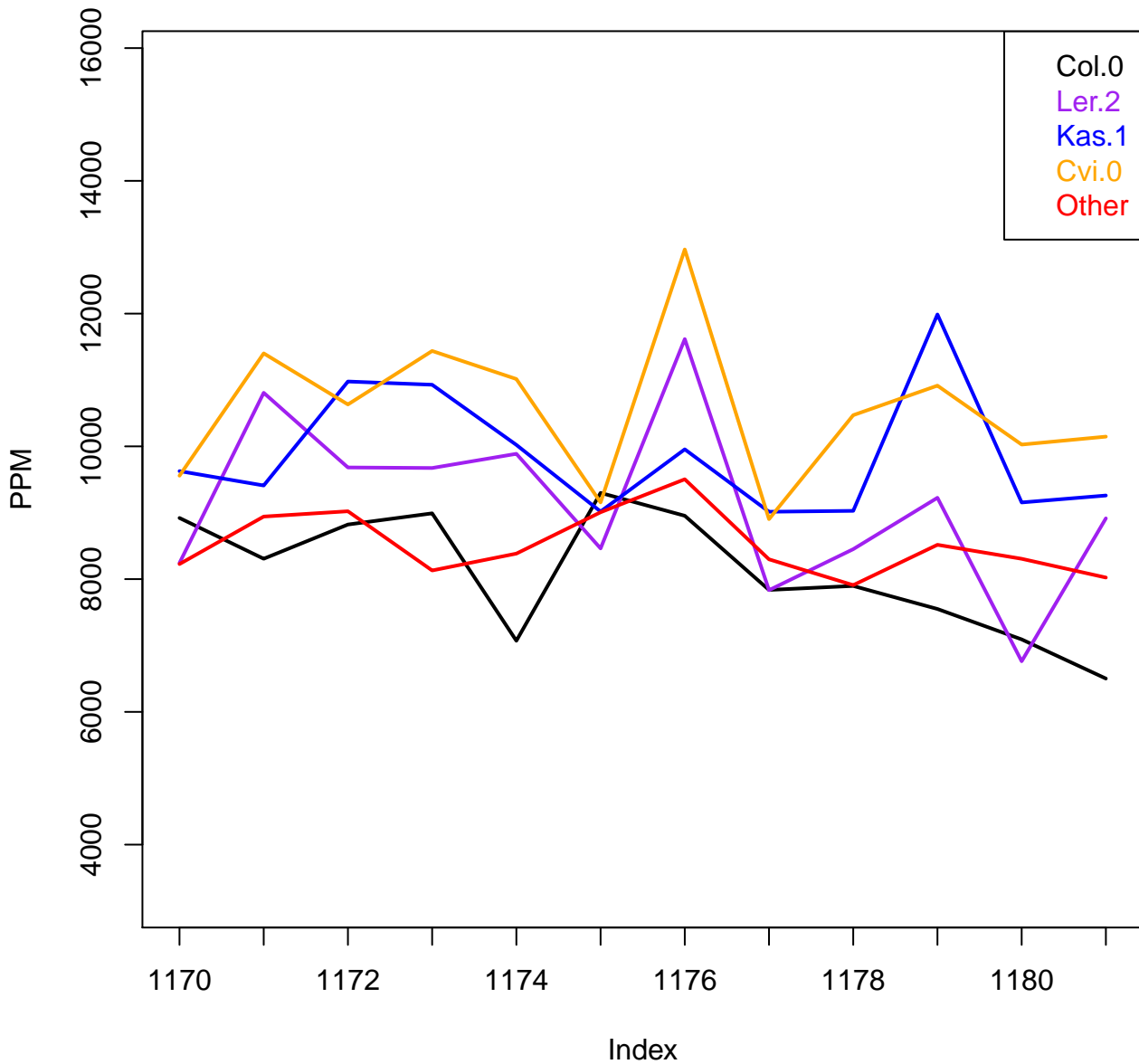

# Ca43

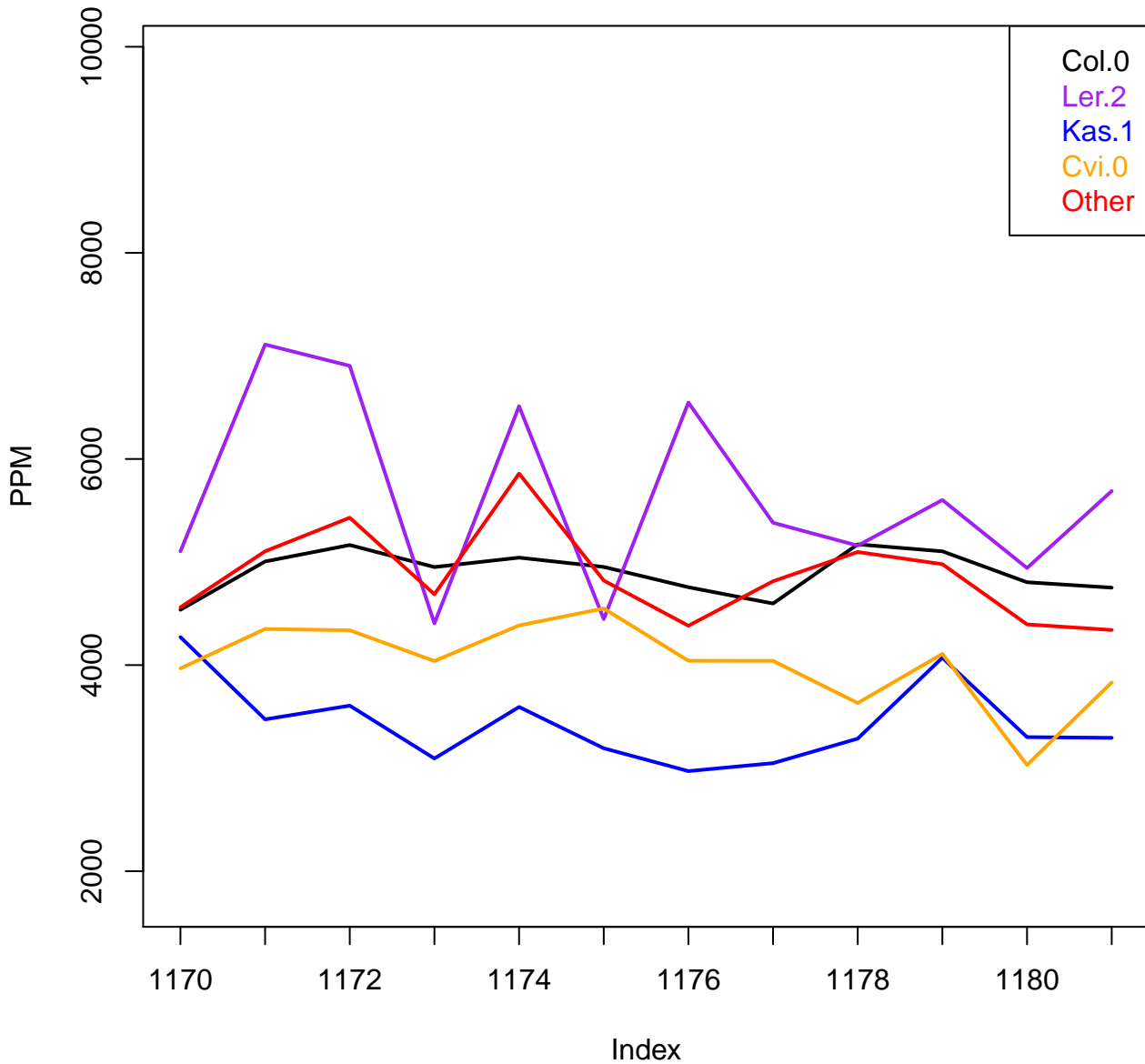

# Mn55

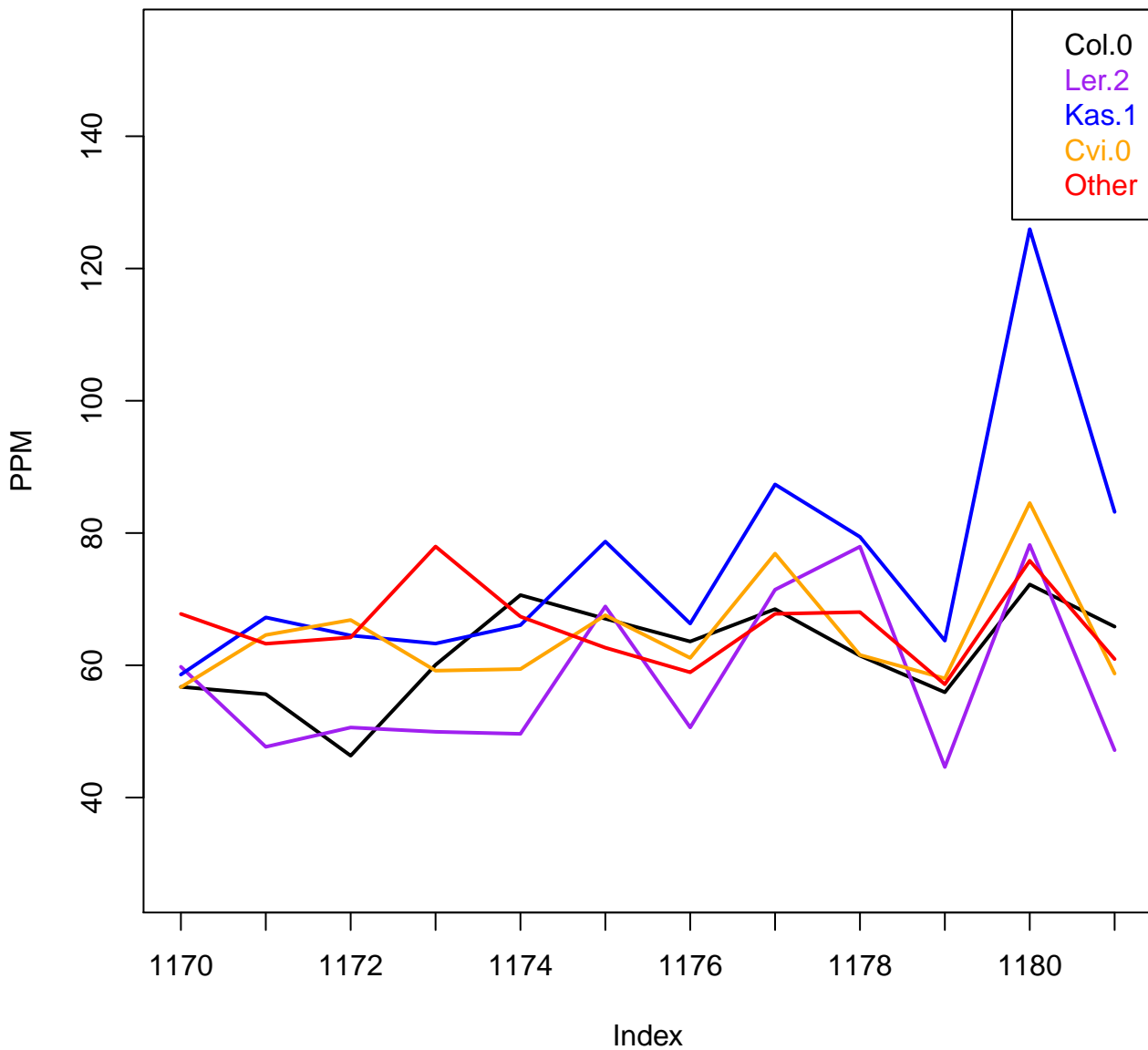

# Fe57

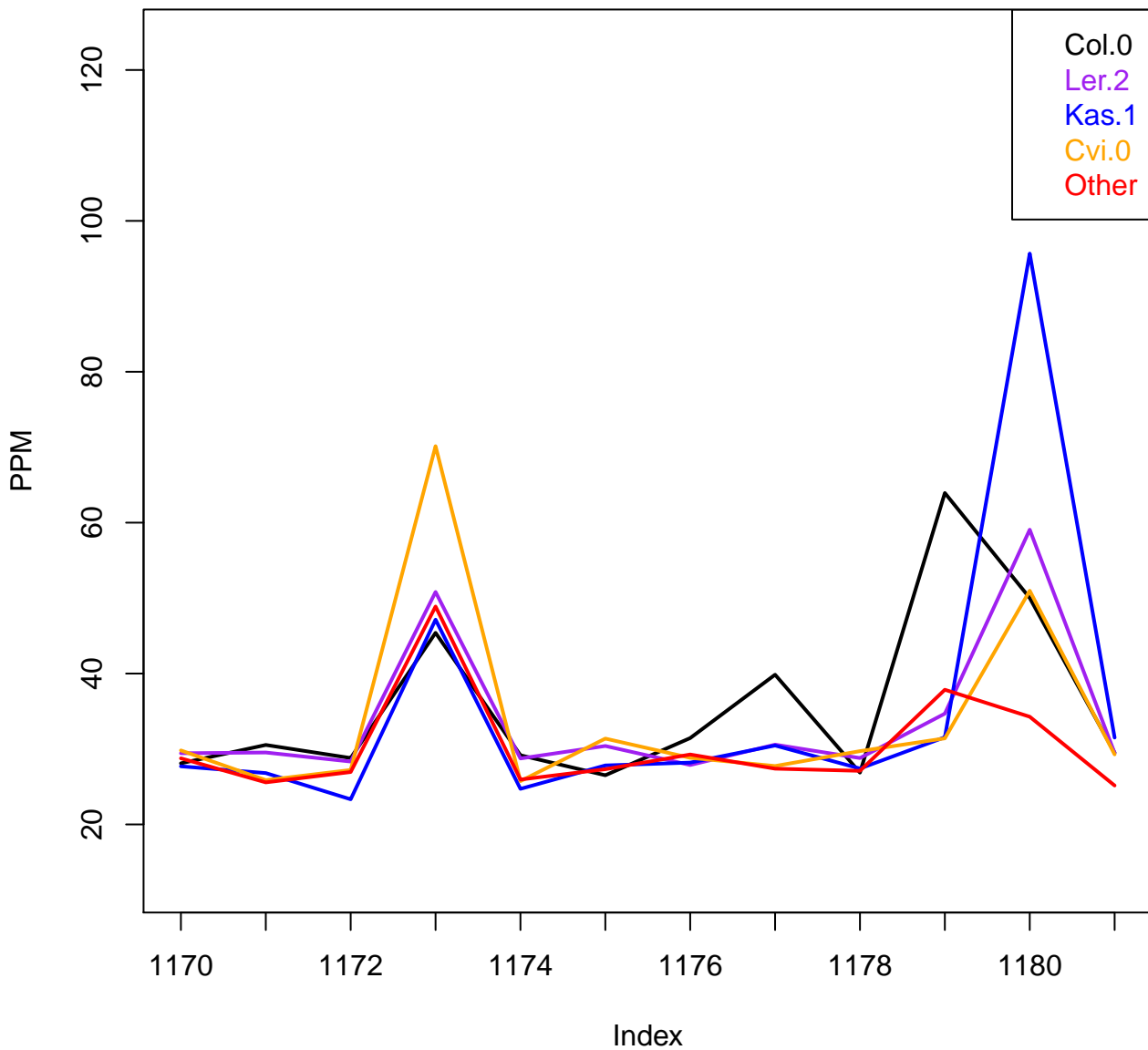

# Co59

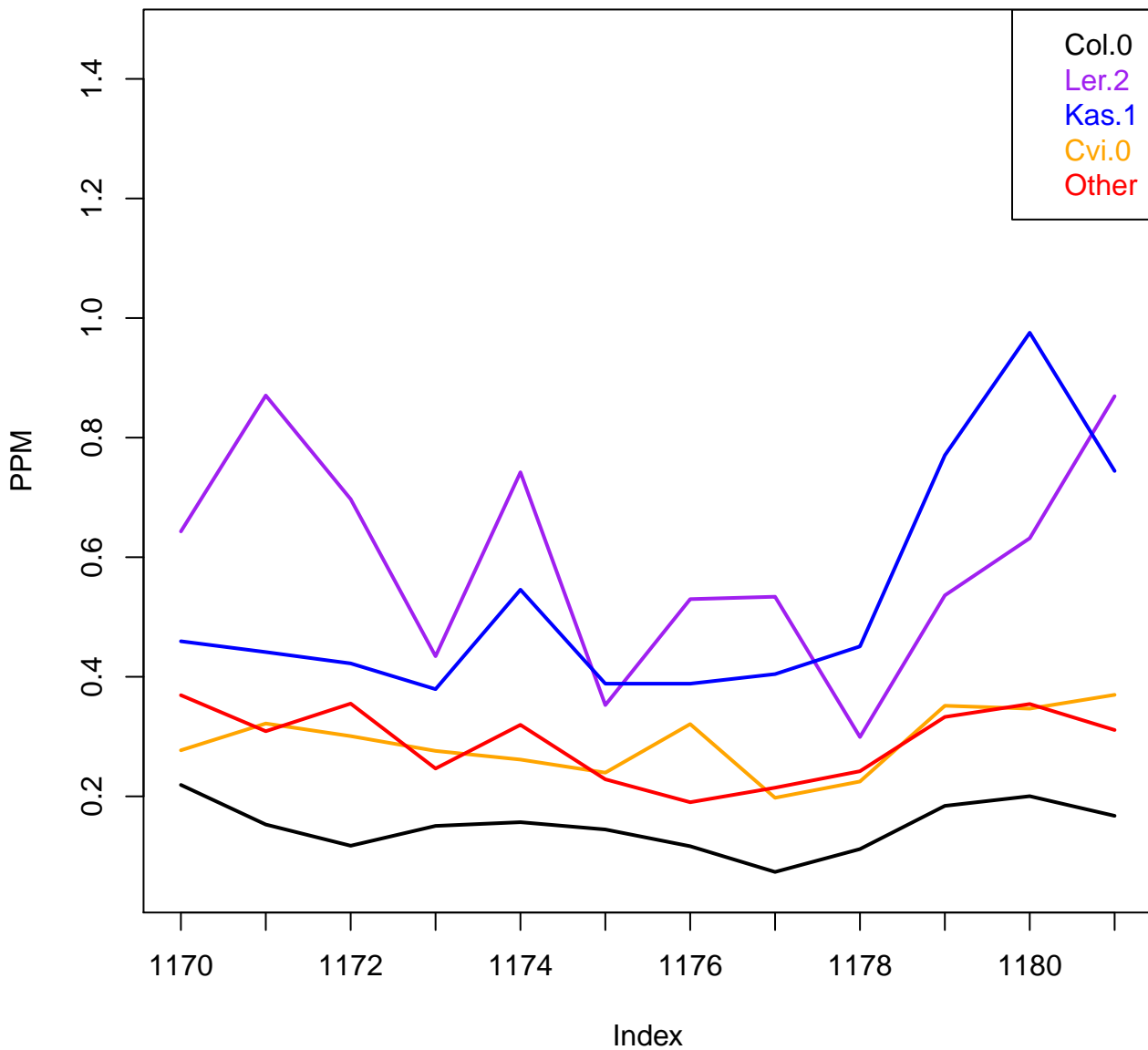

# Ni60

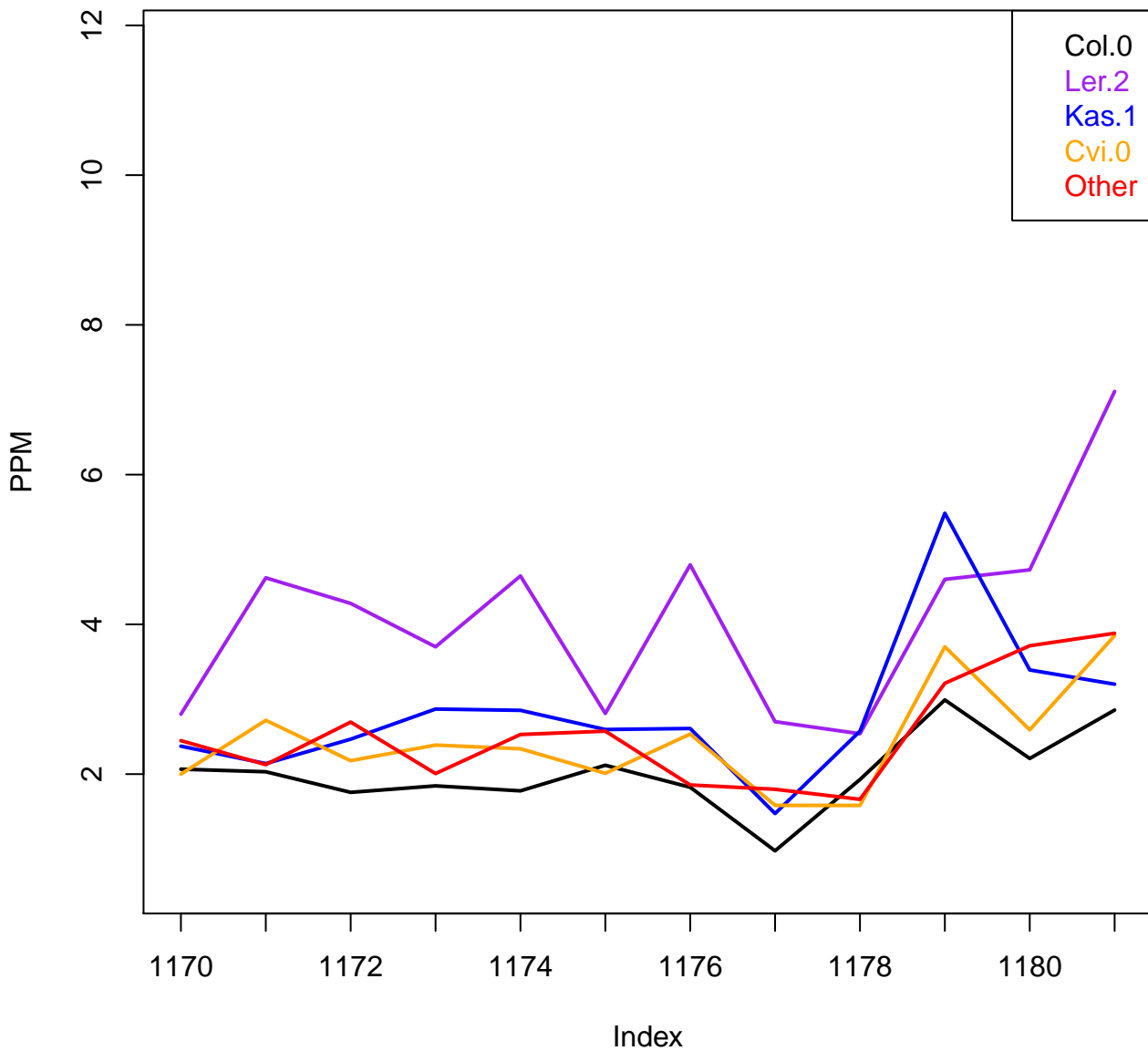

# Cu65

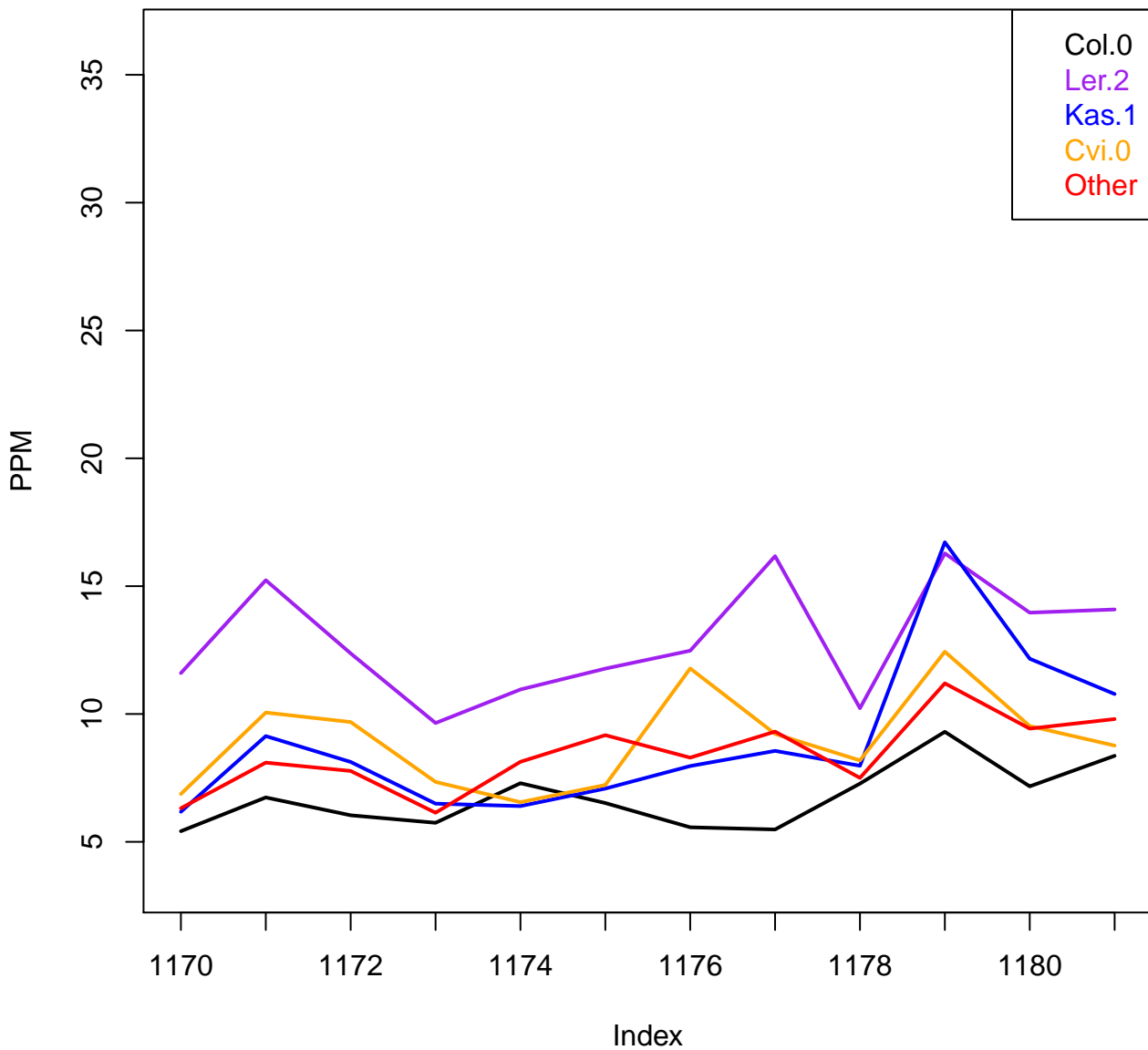

# Zn66

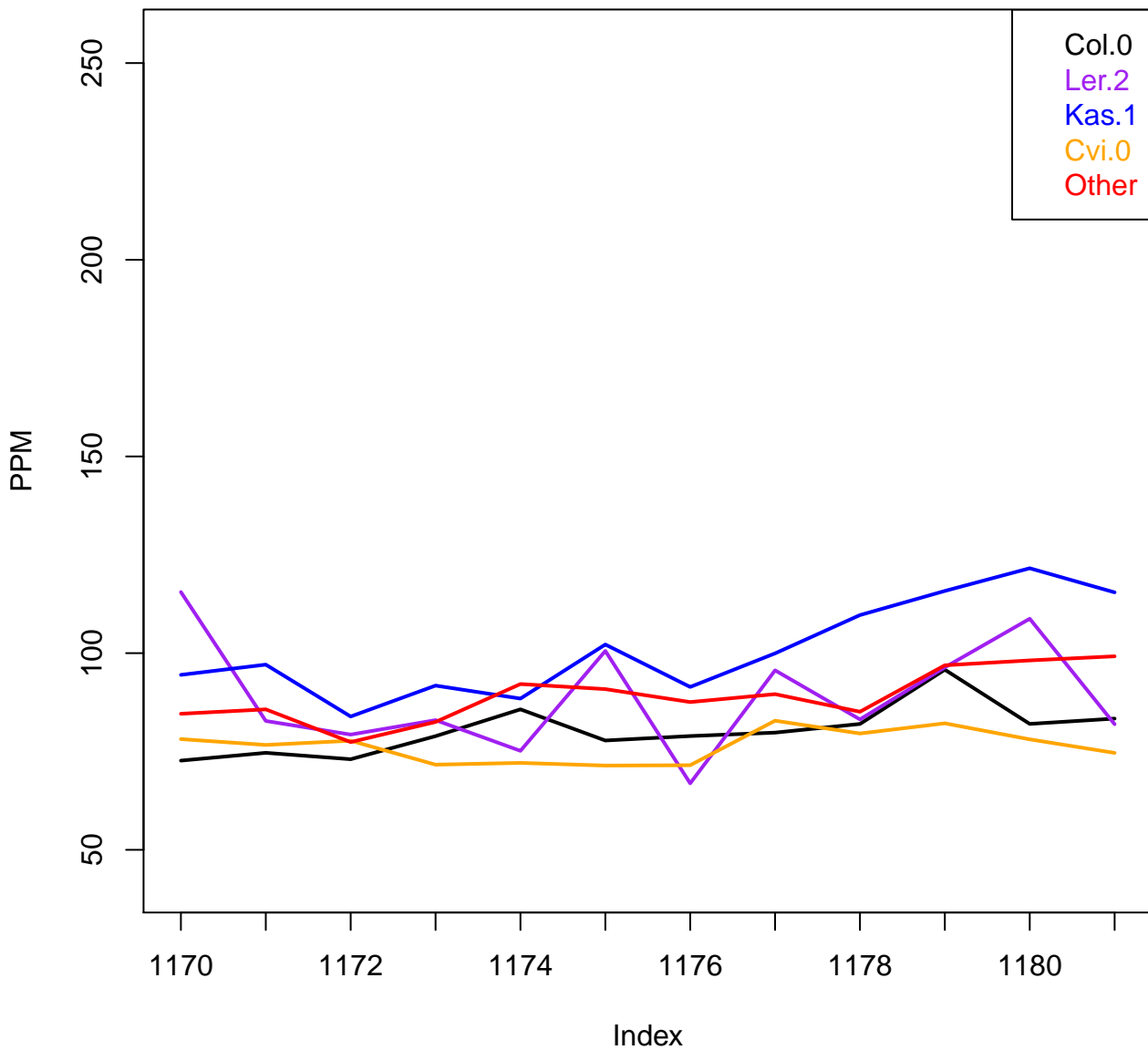

# As75

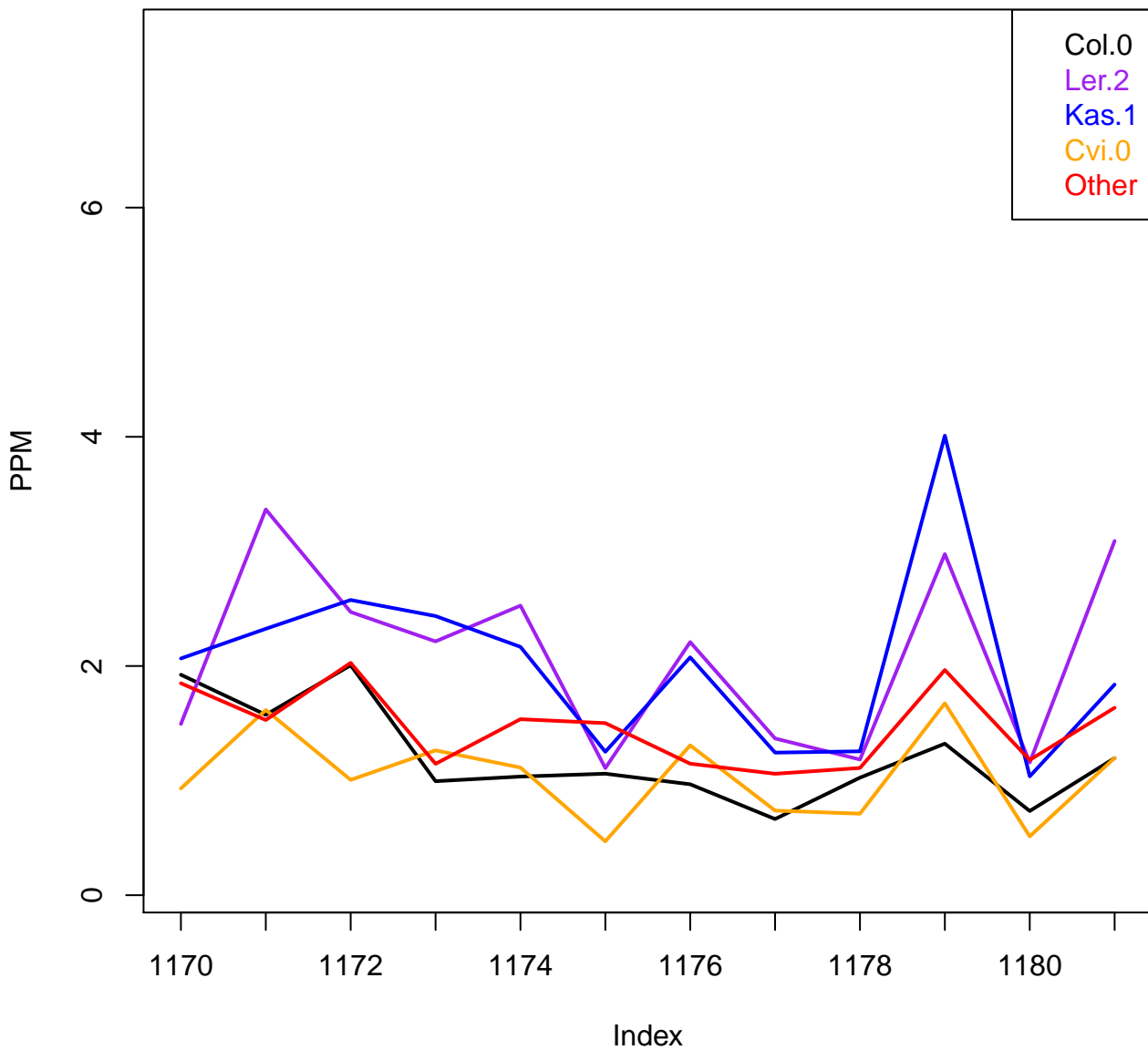

# Se82

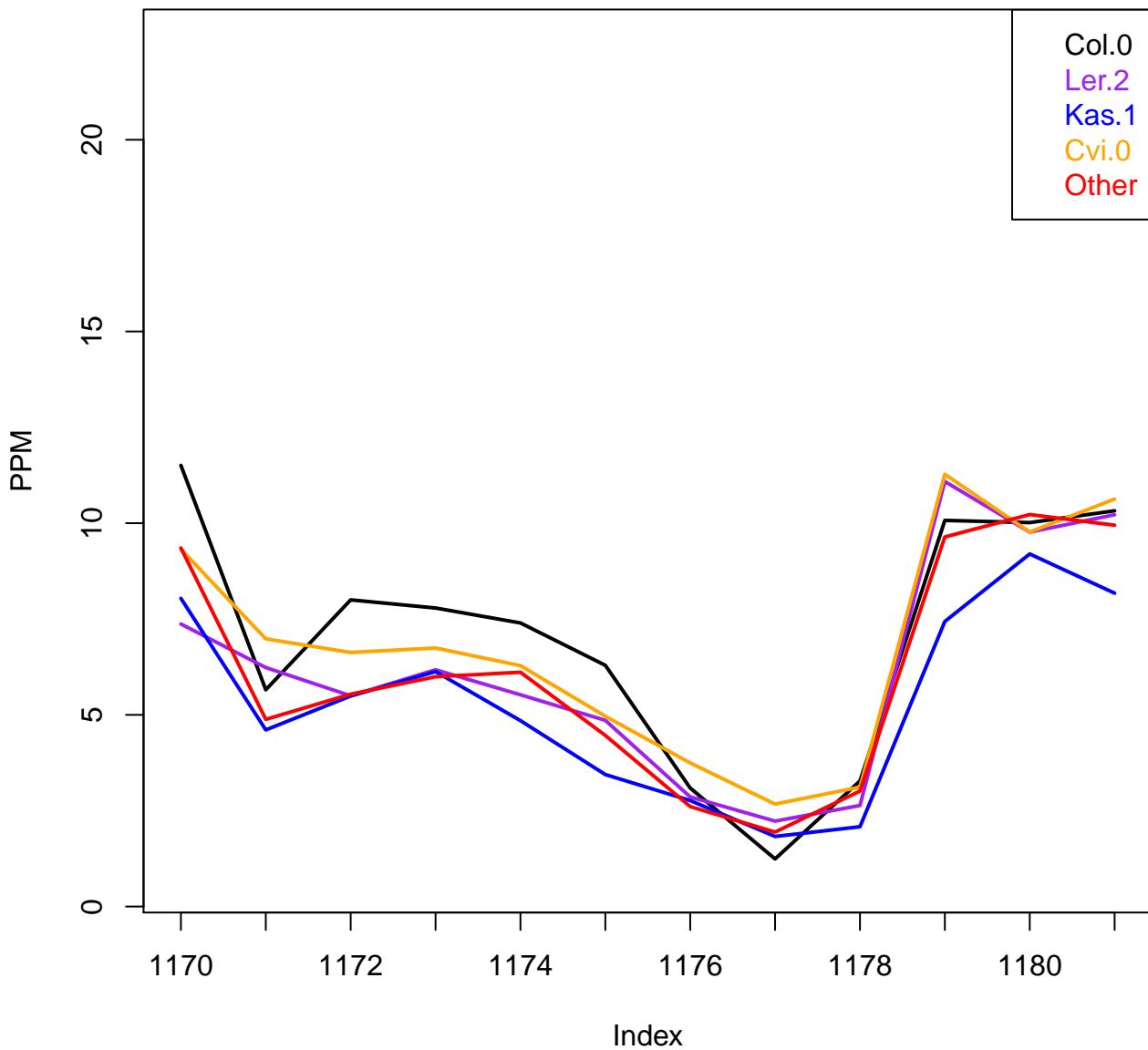

# Rb85

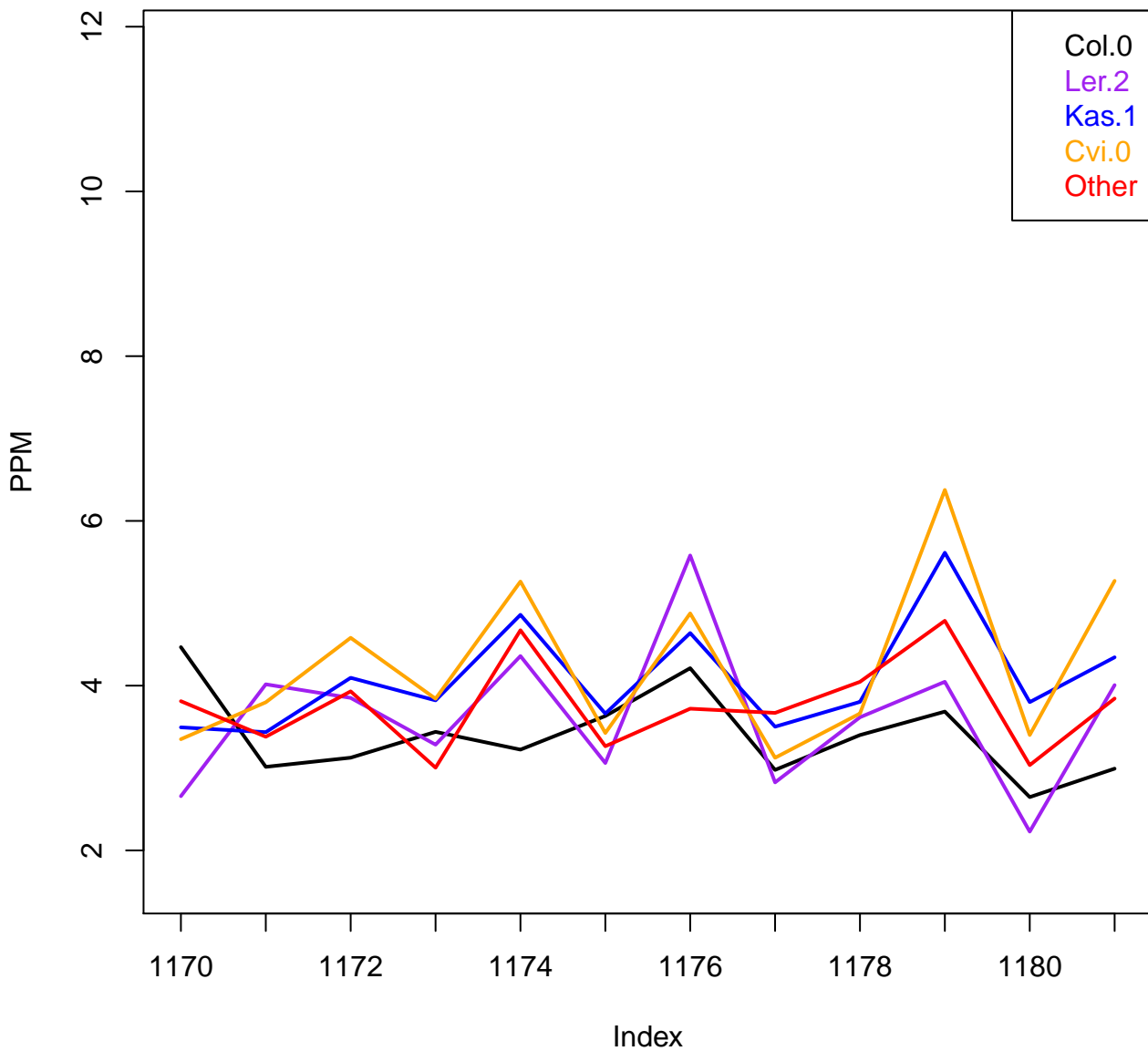

# Mo98

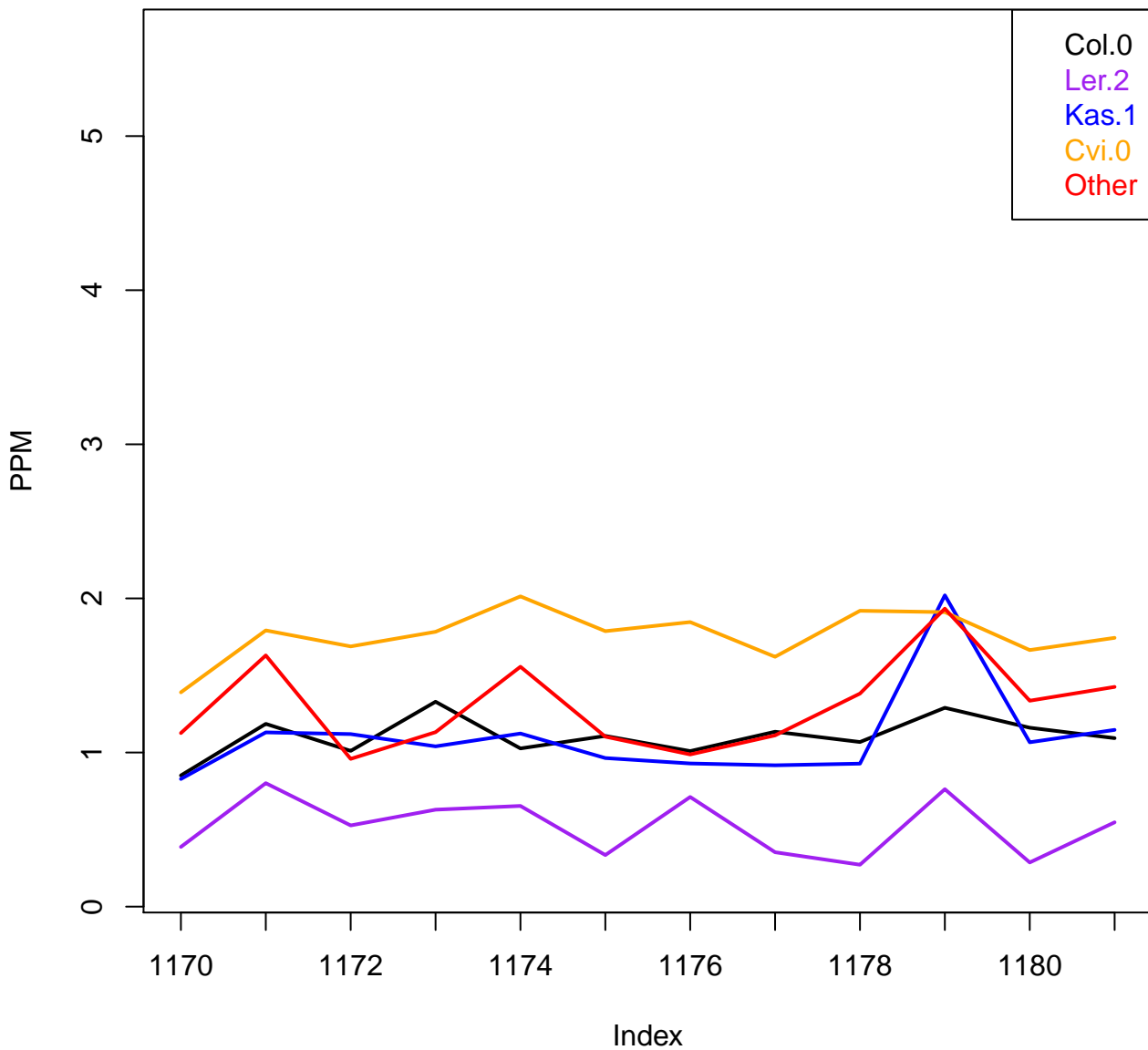

# Cd114

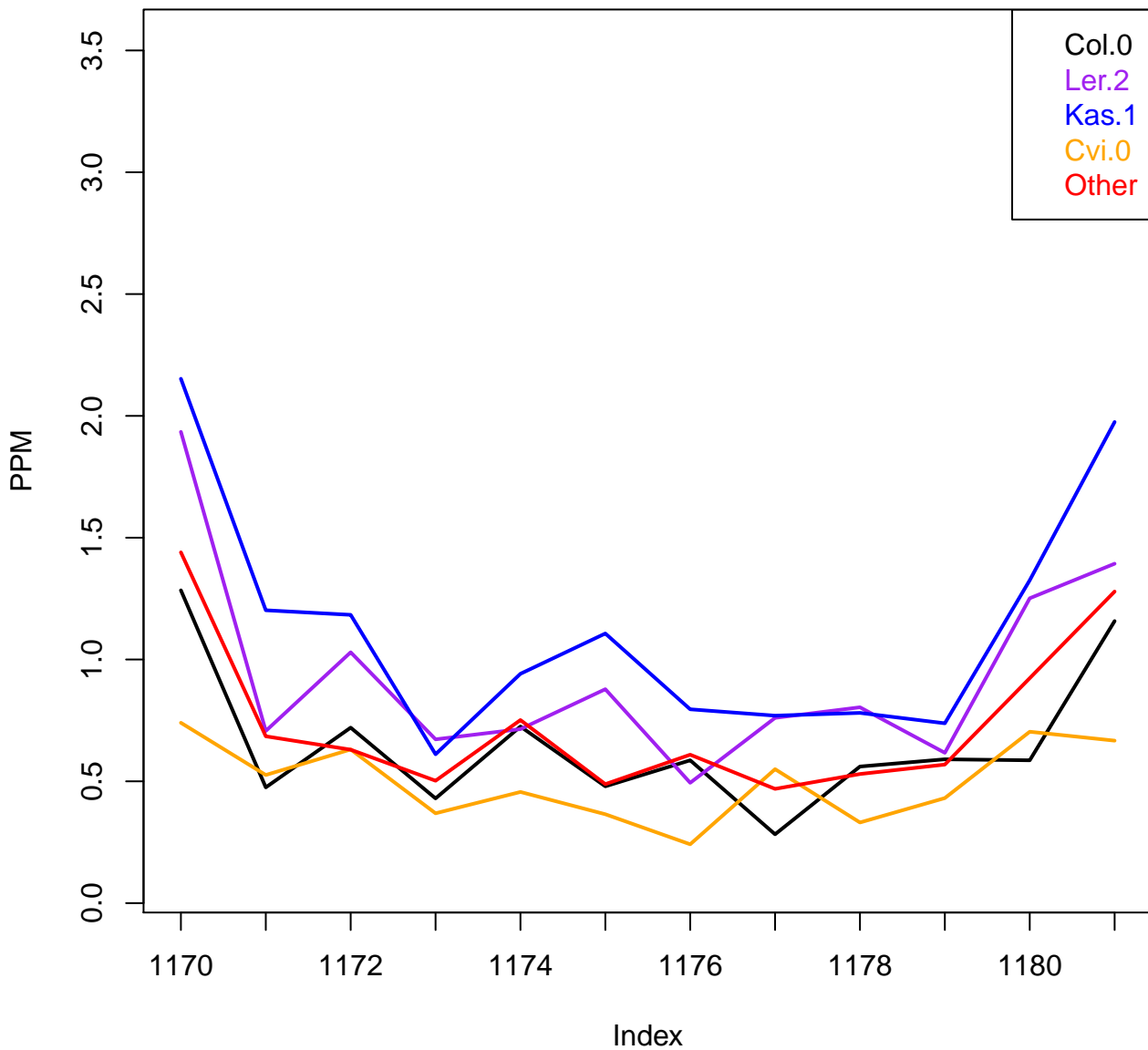

Supplement: Figure S6 — Plot of control line averages for each tray before normalization for Seed experiment. All non-control lines are averaged into the “Other” line. (PDF) [file pone.0035121.s008.pdf]

# Li7

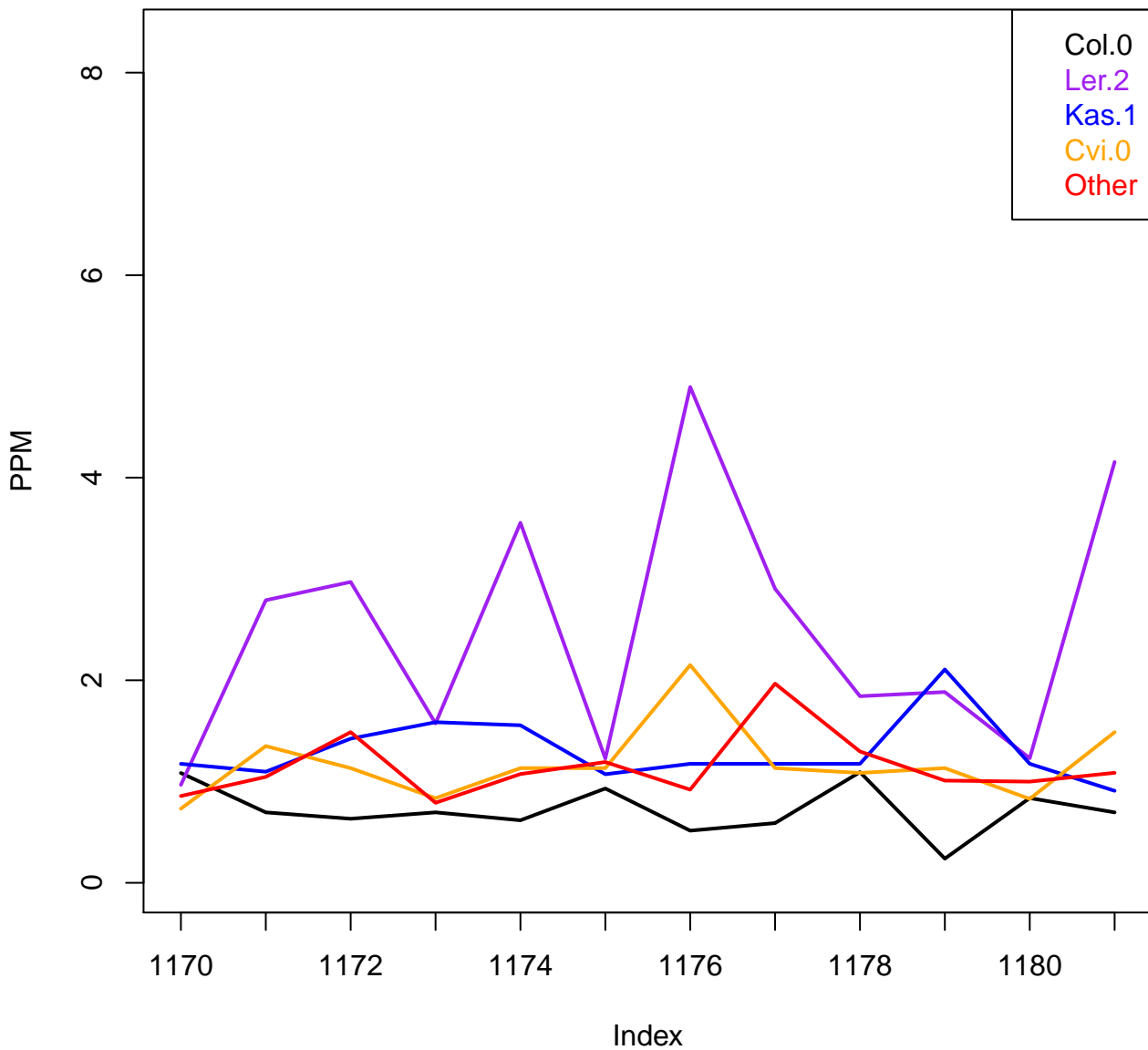

# B11

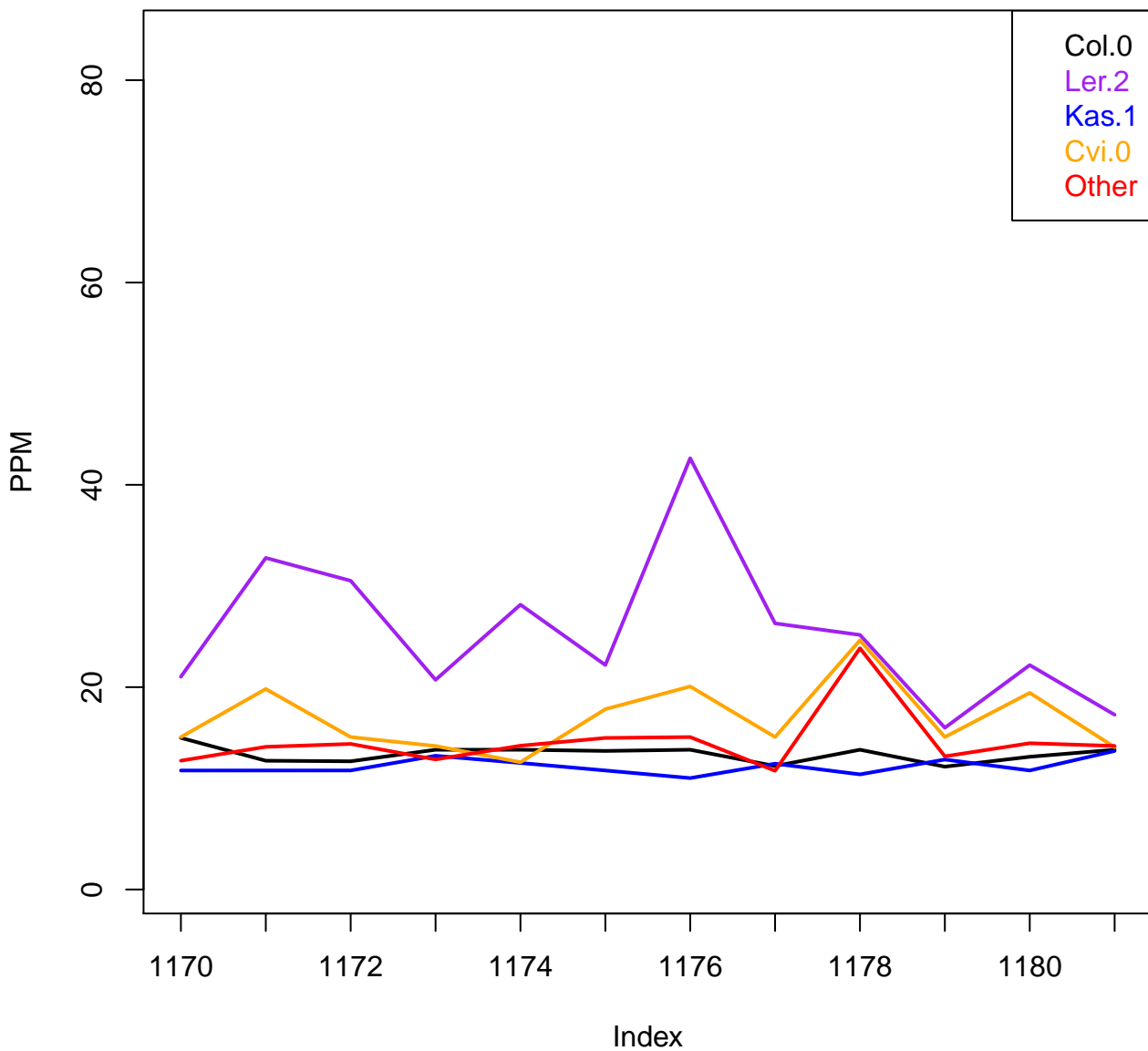

# Na23

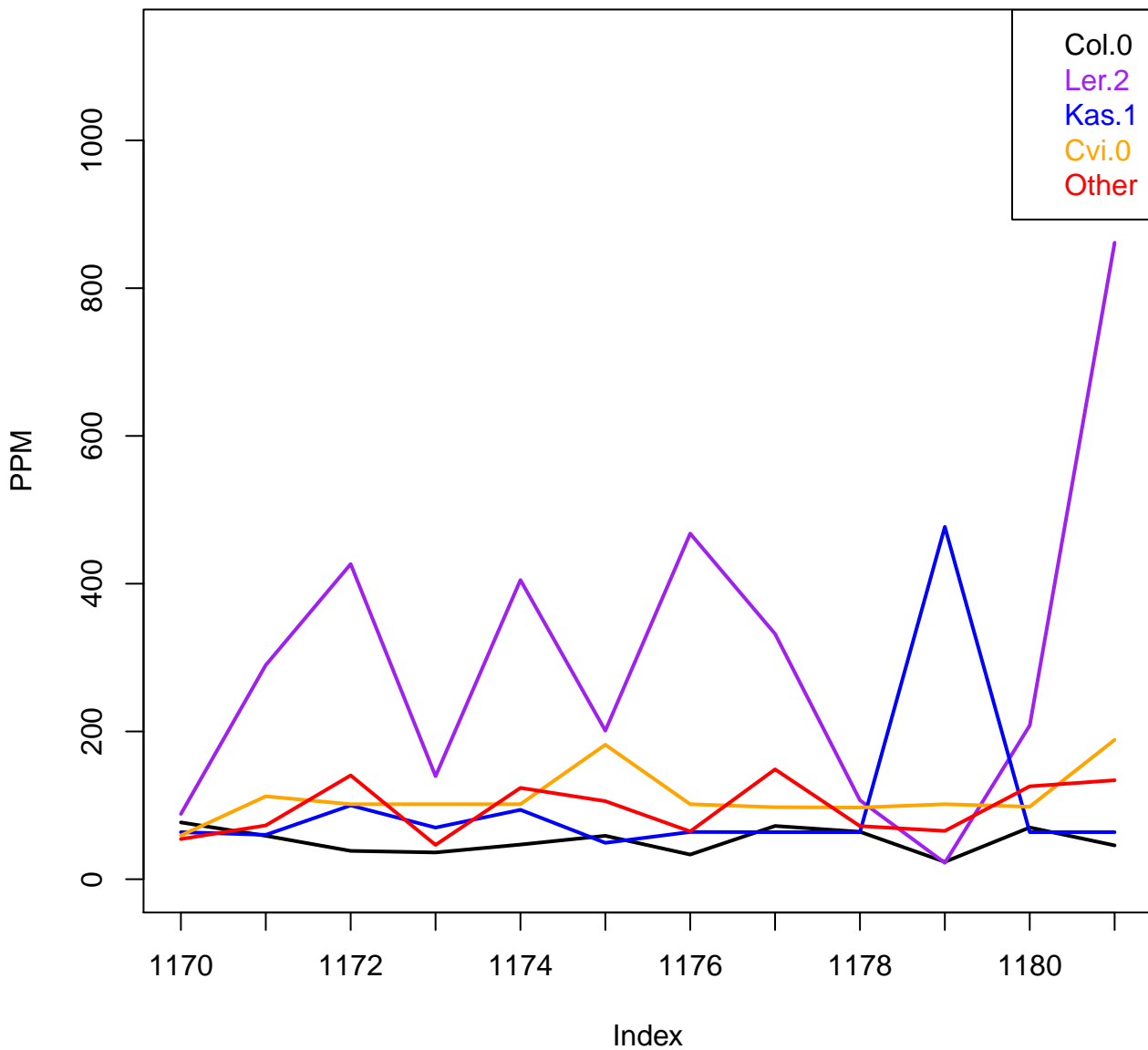

# Mg25

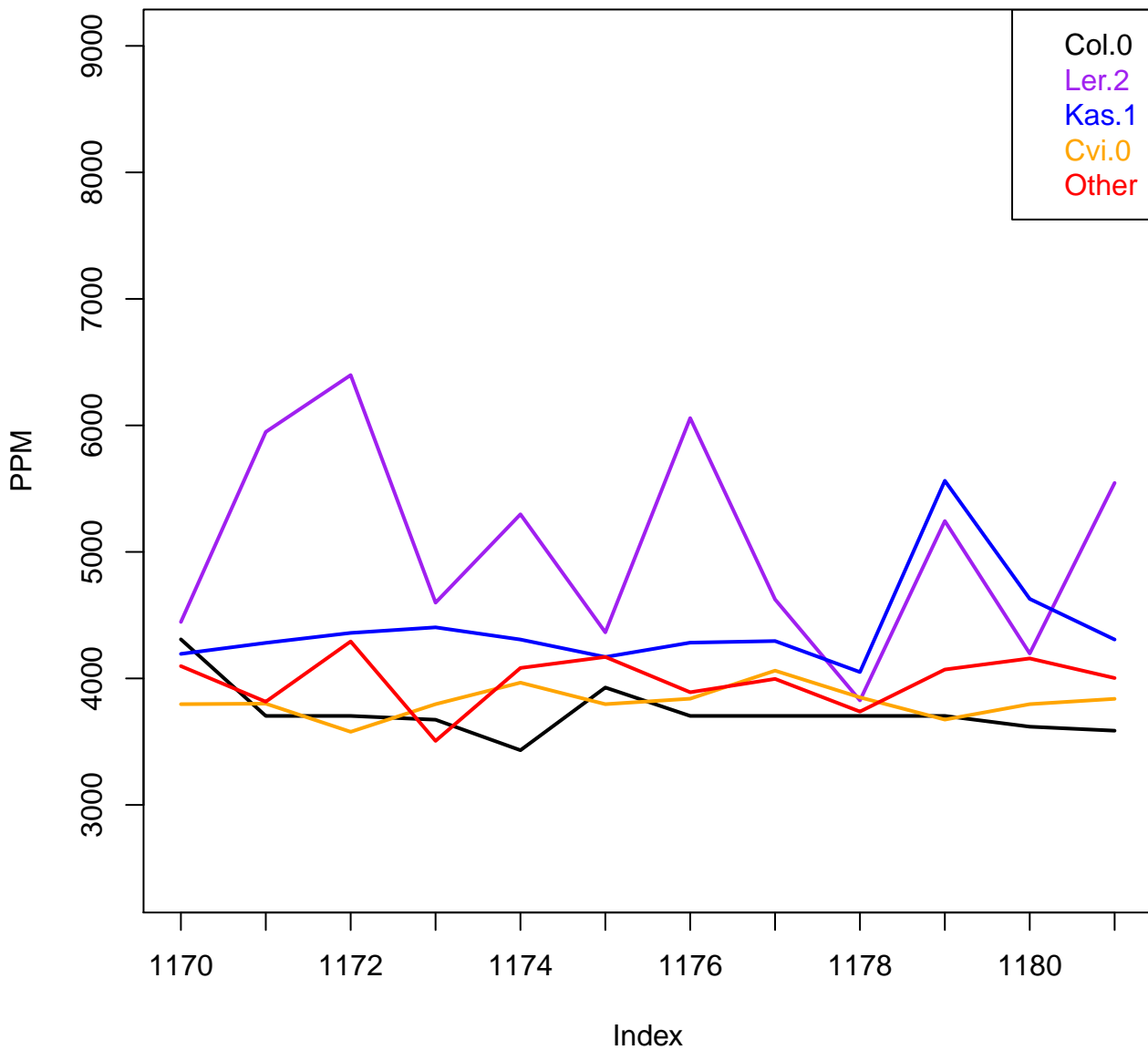

# P31

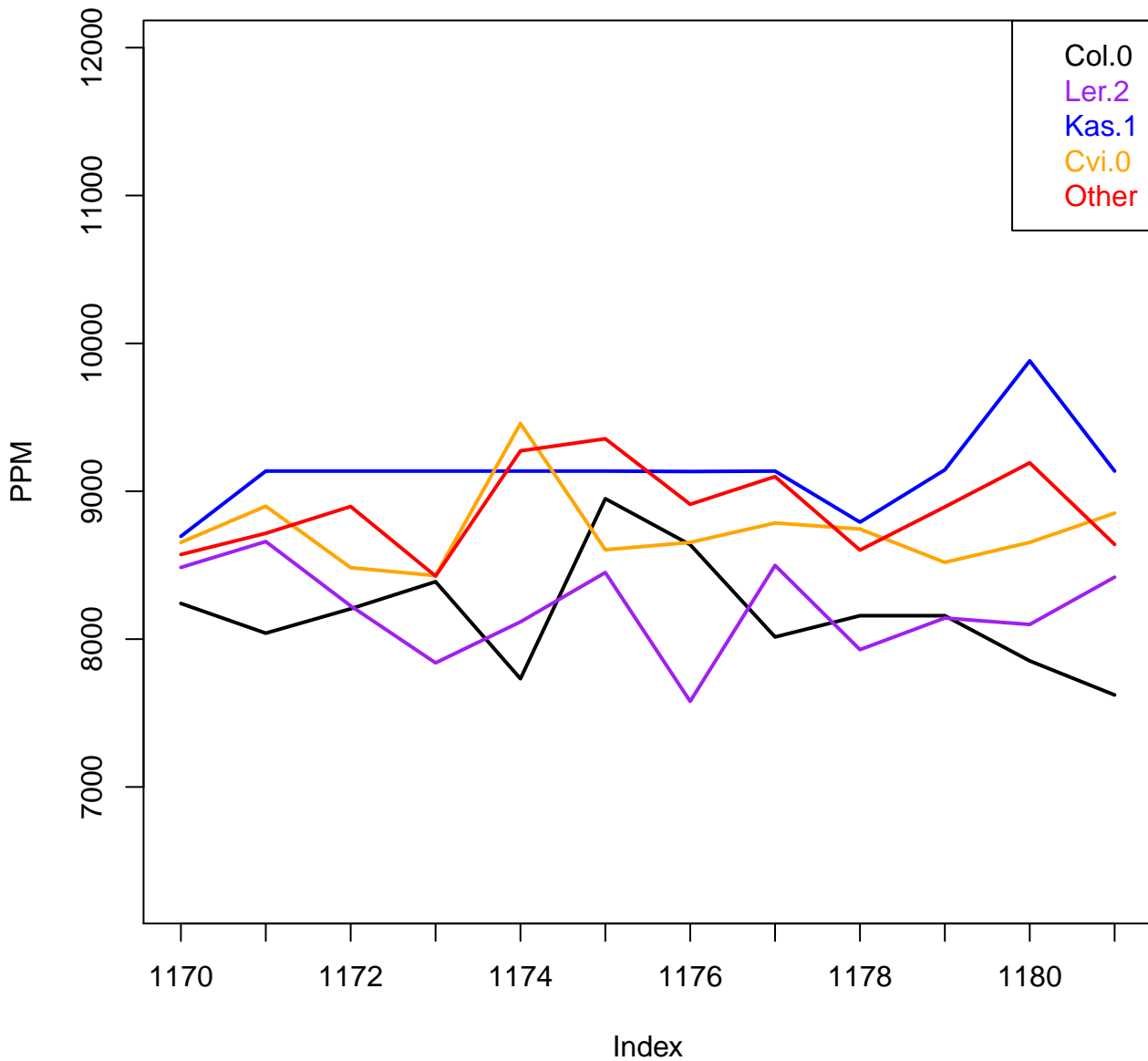

# S34

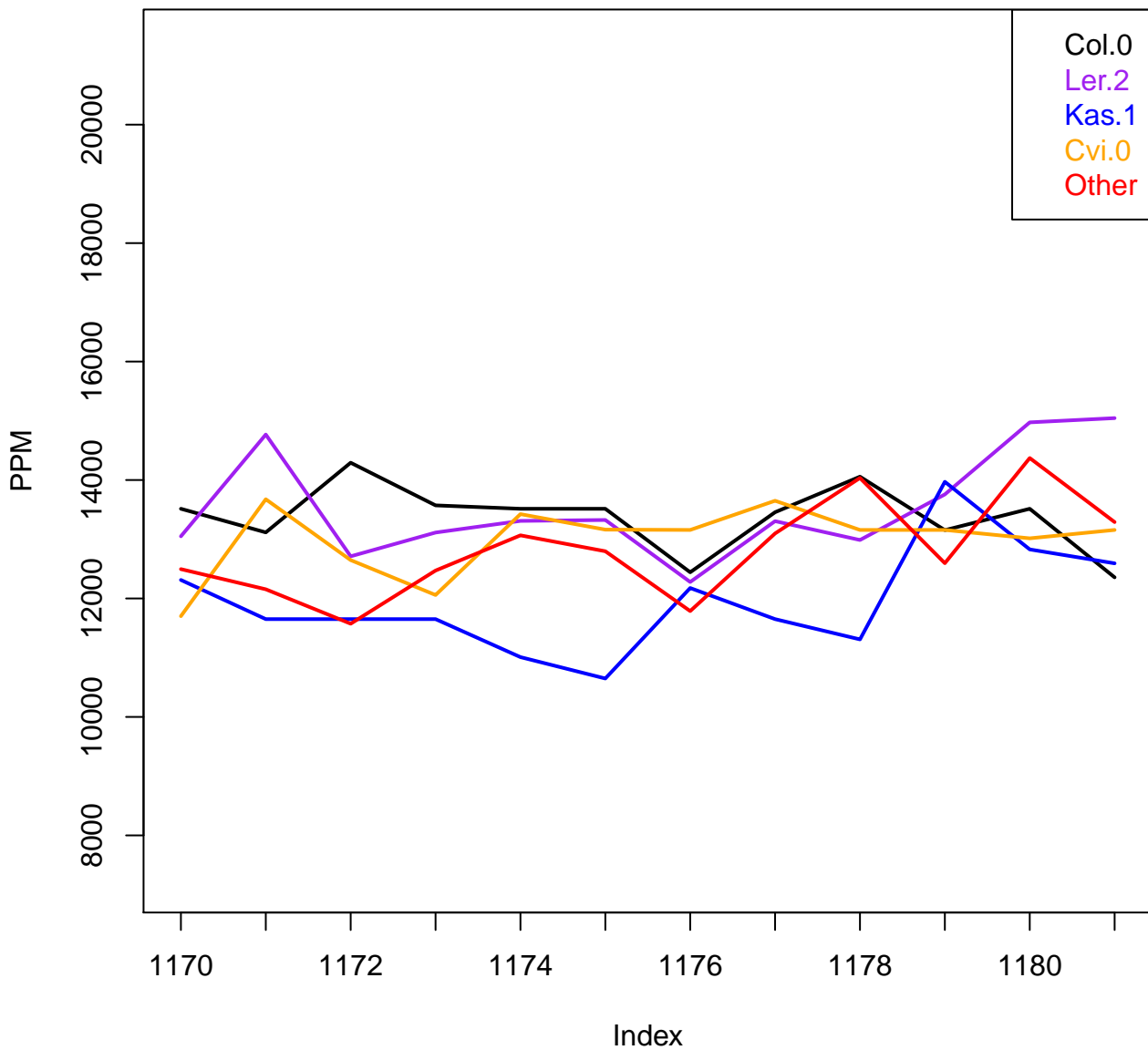

# K39

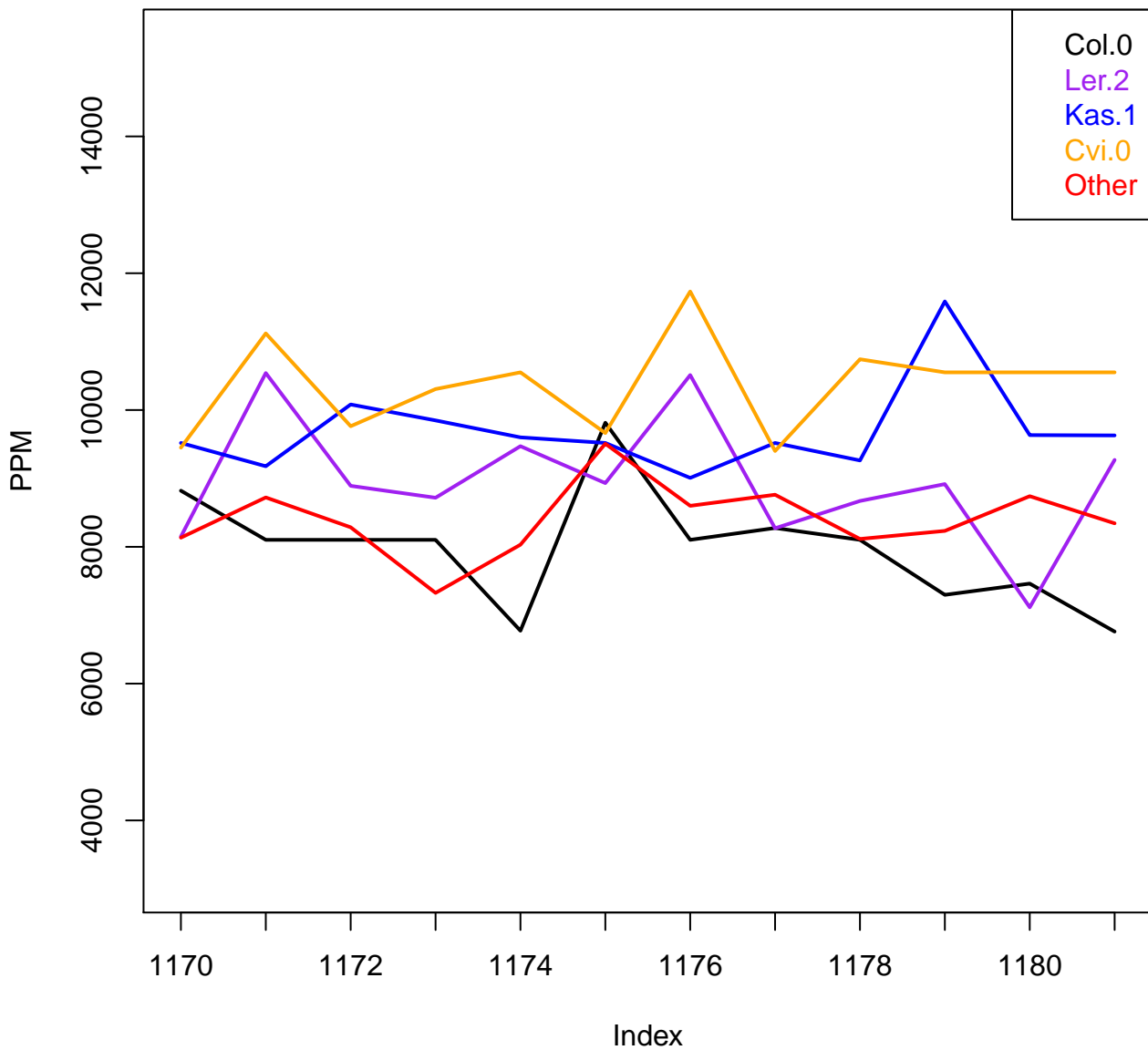

# Ca43

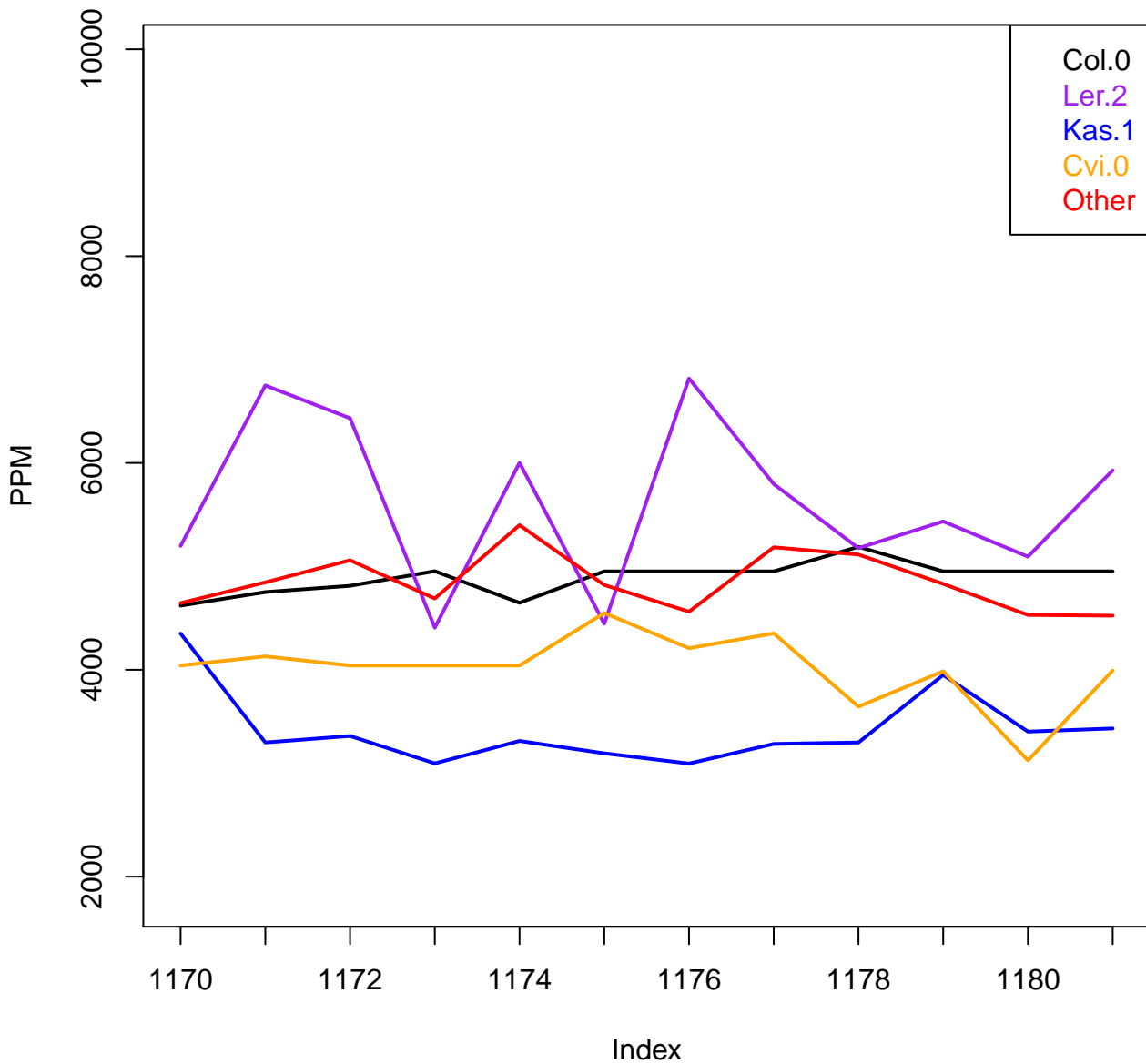

# Mn55

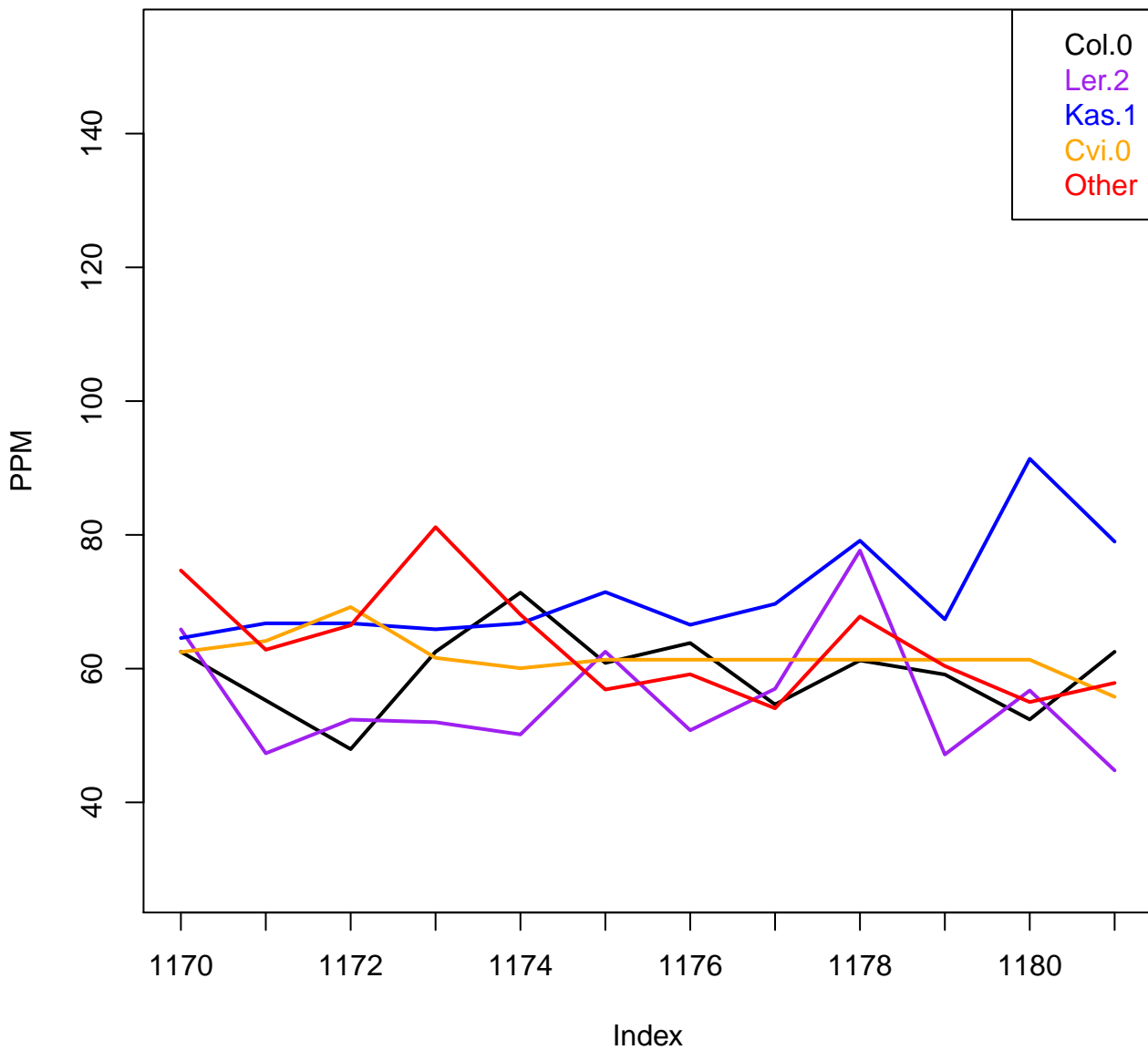

# Fe57

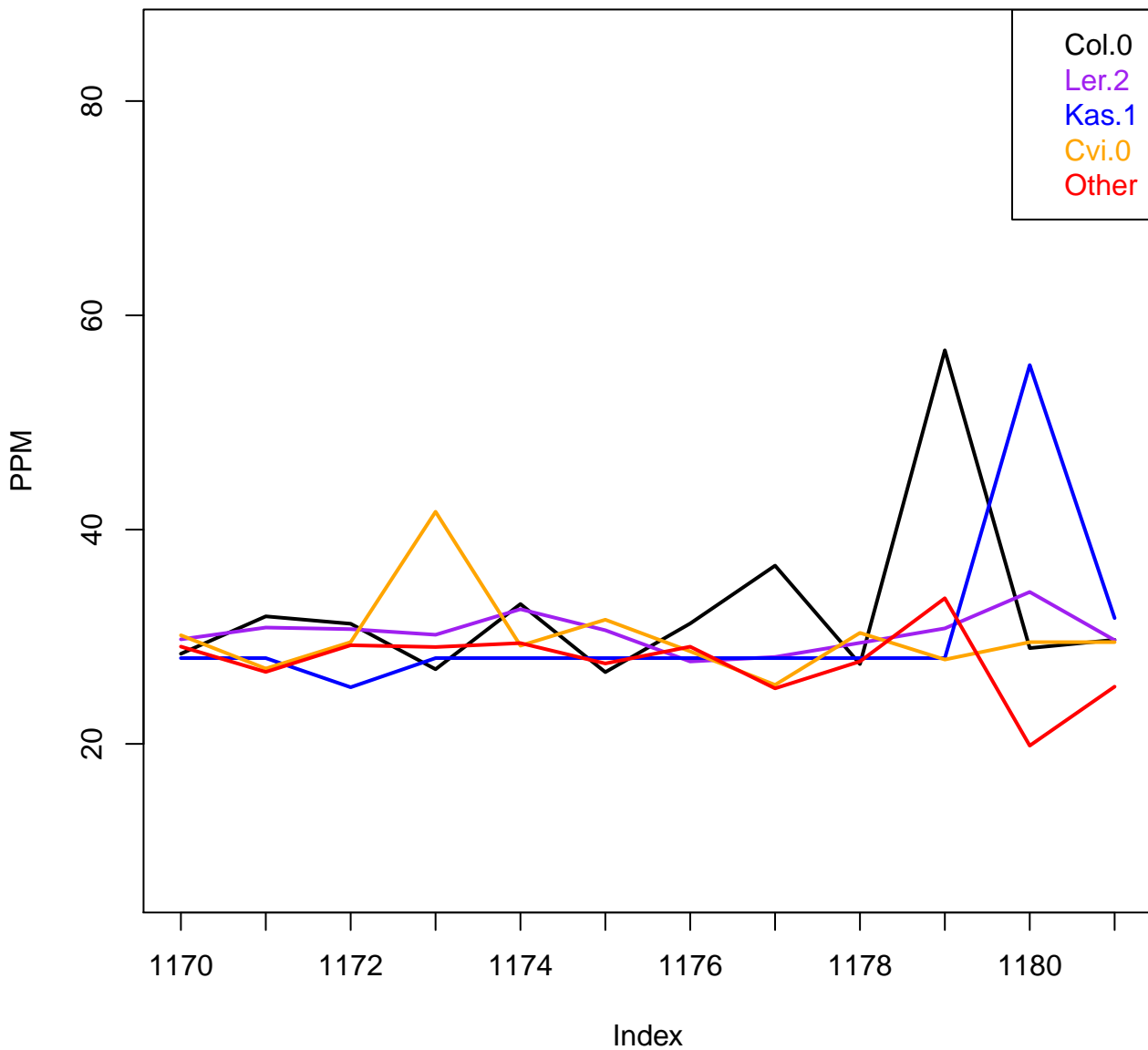

# Co59

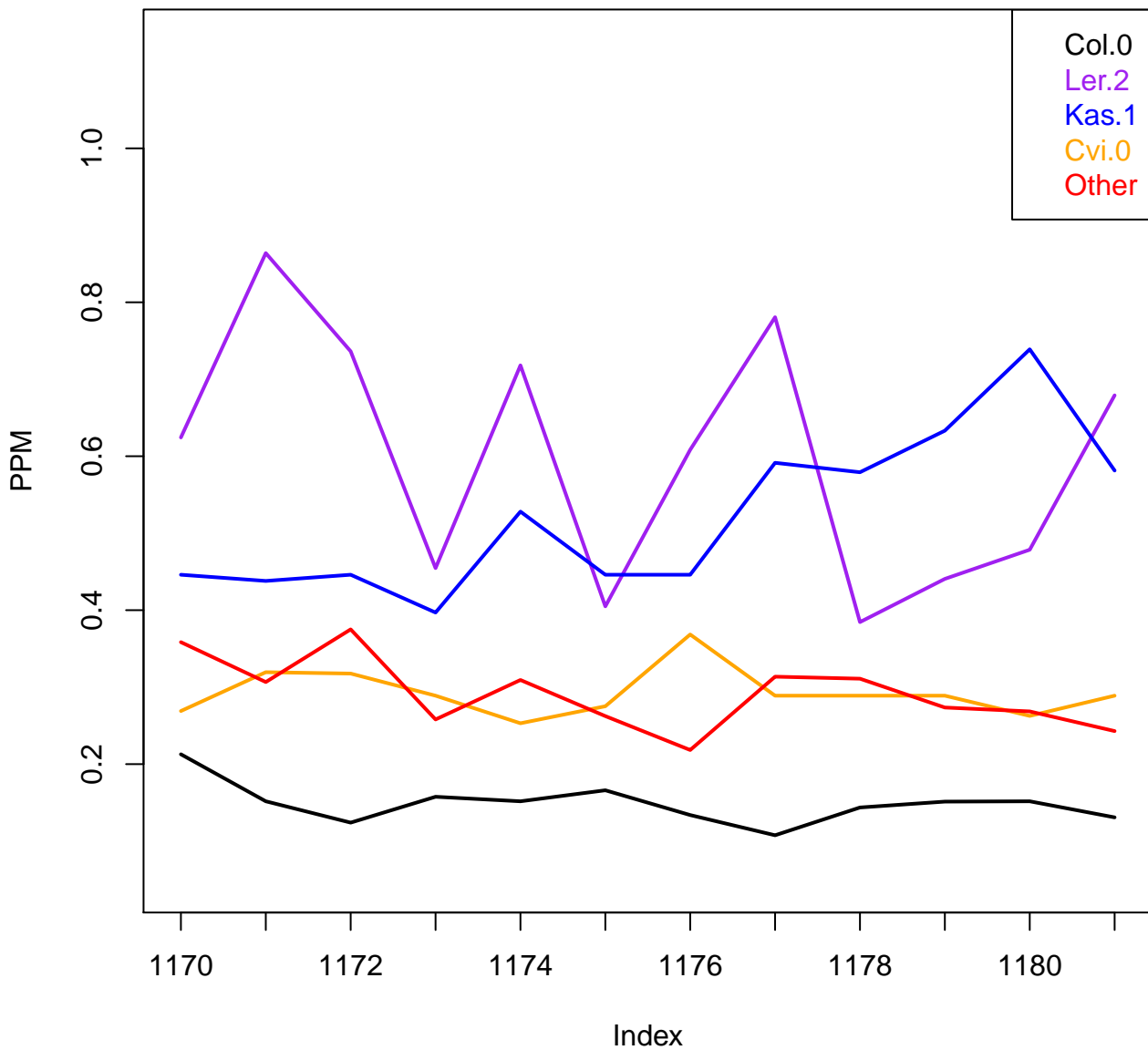

# Ni60

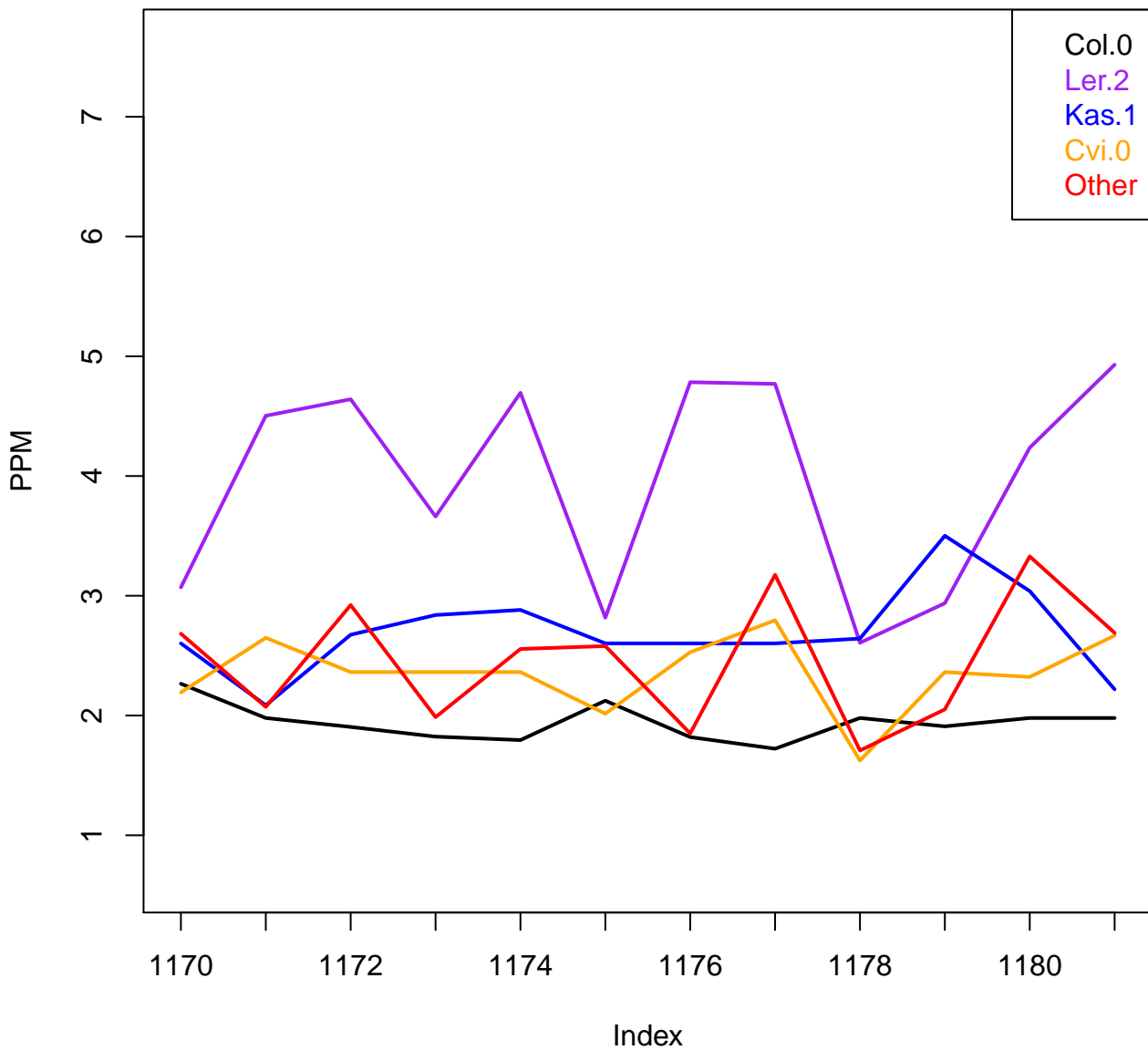

# Cu65

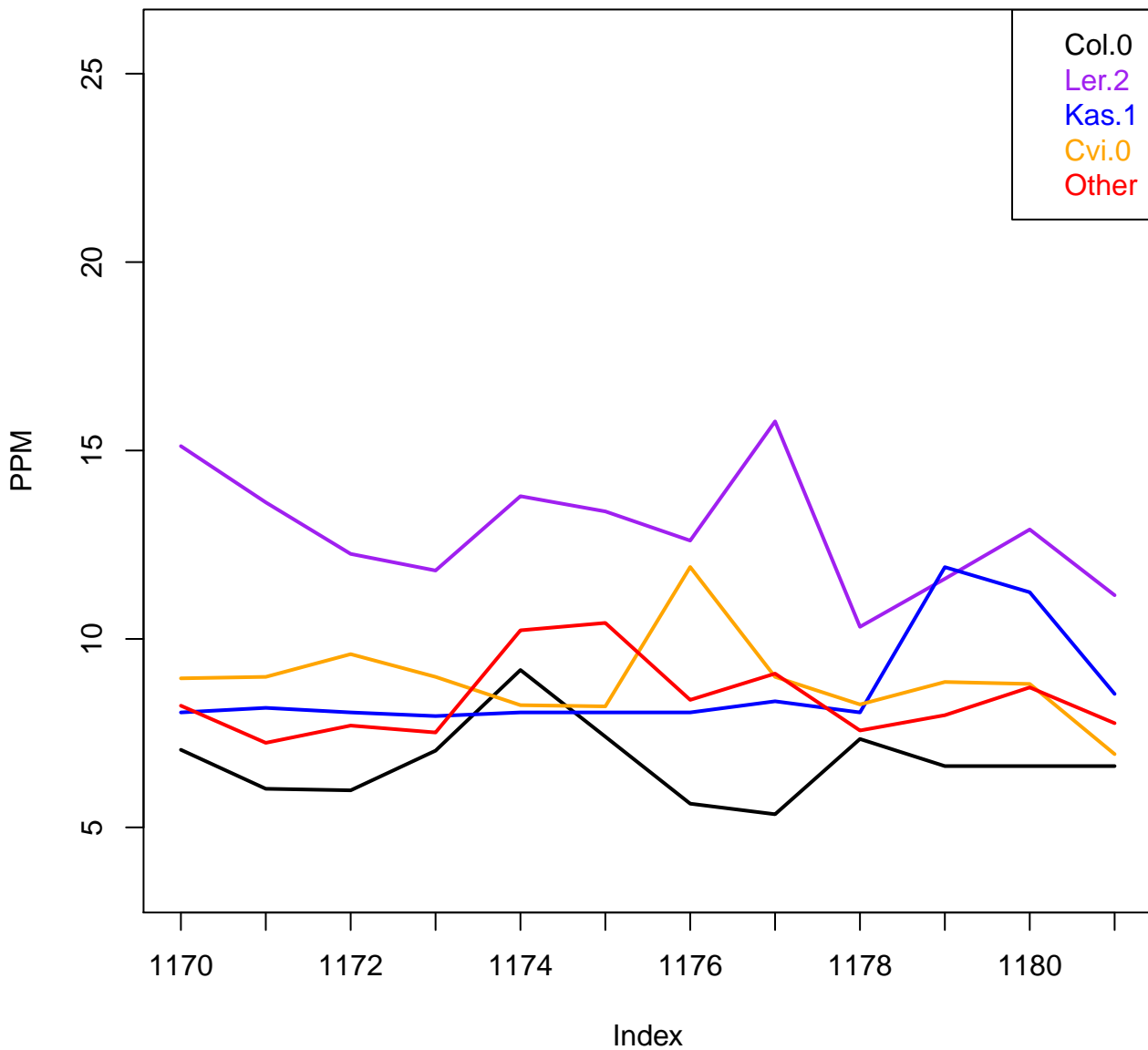

# Zn66

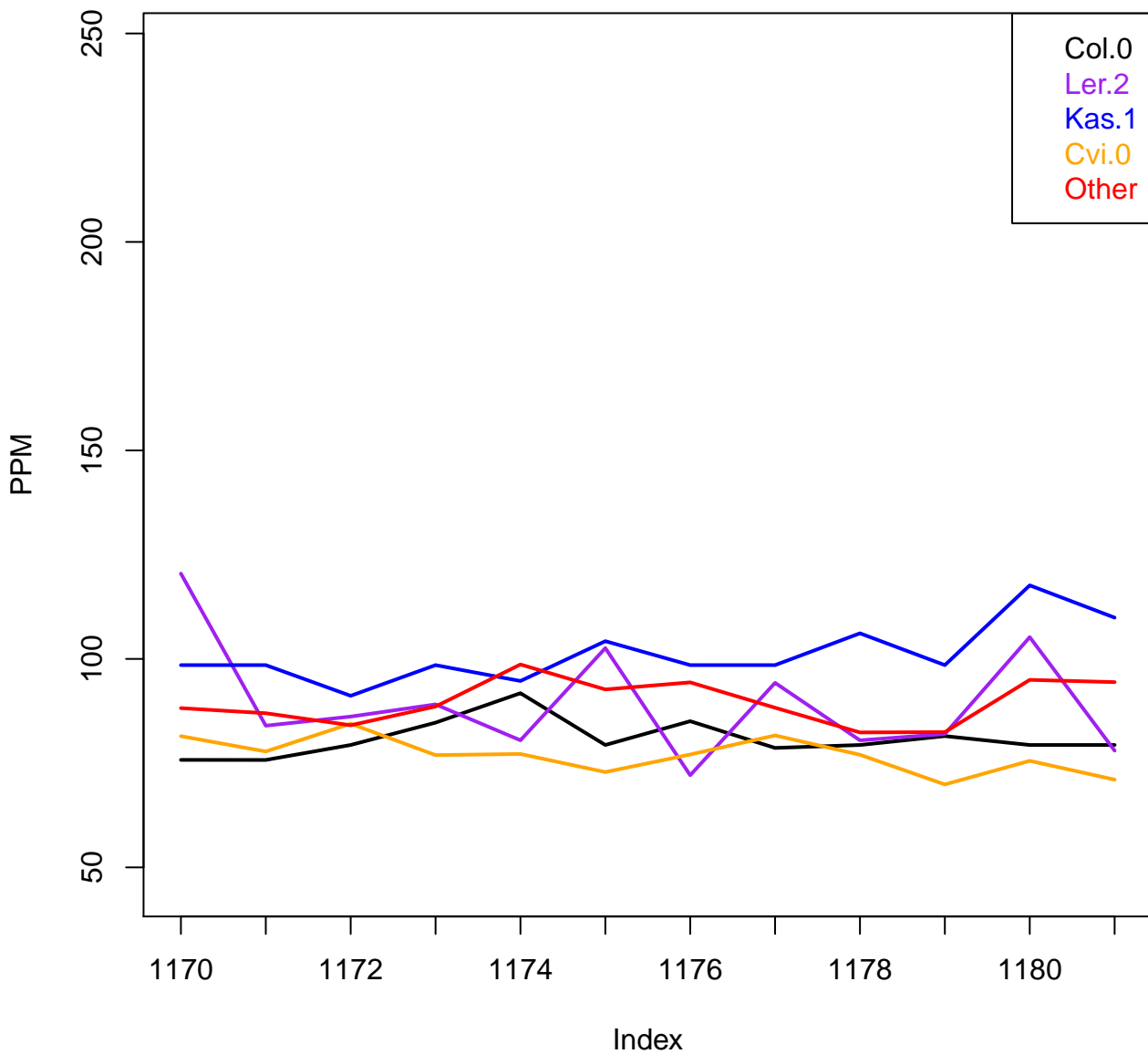

# As75

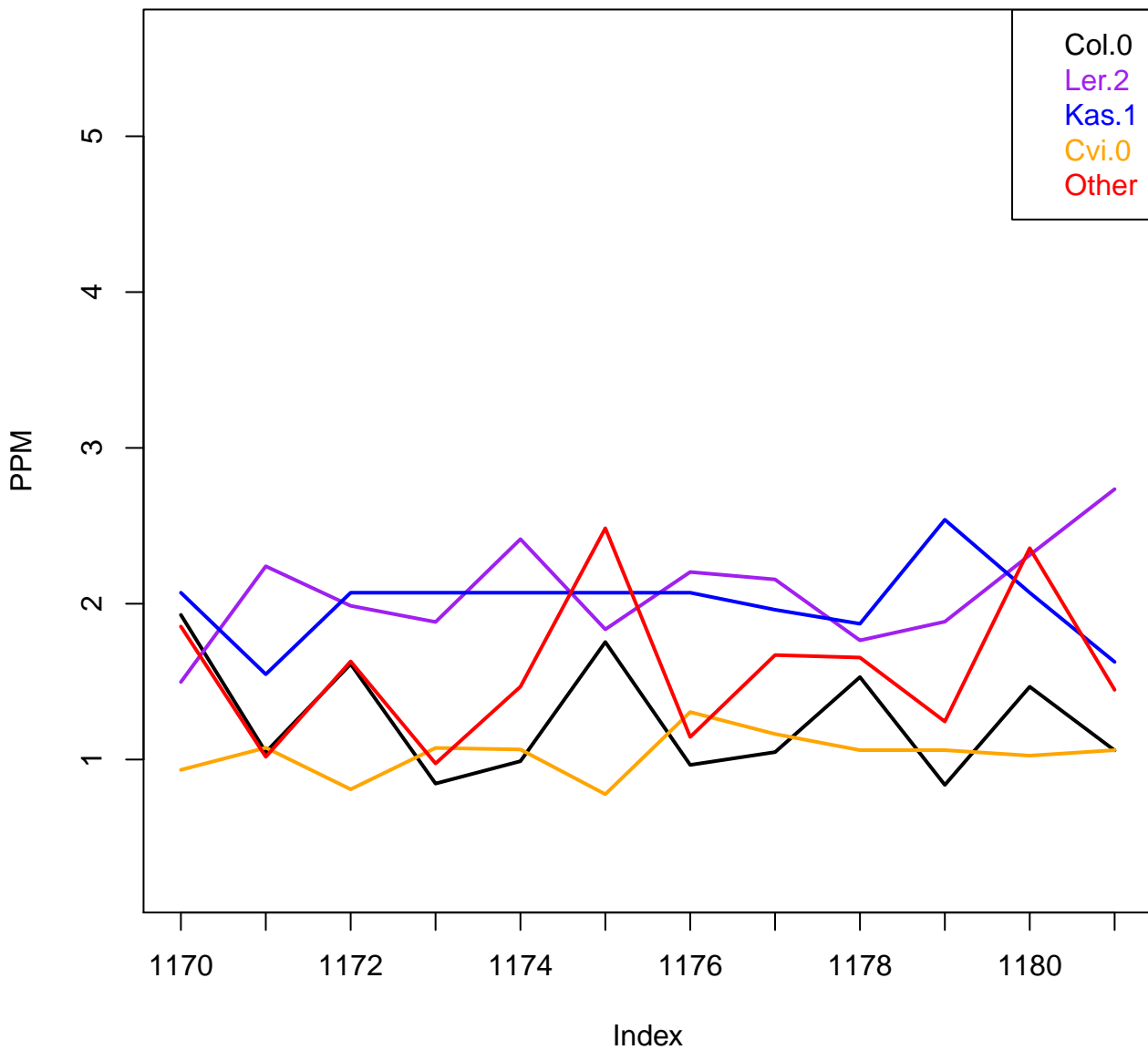

# Se82

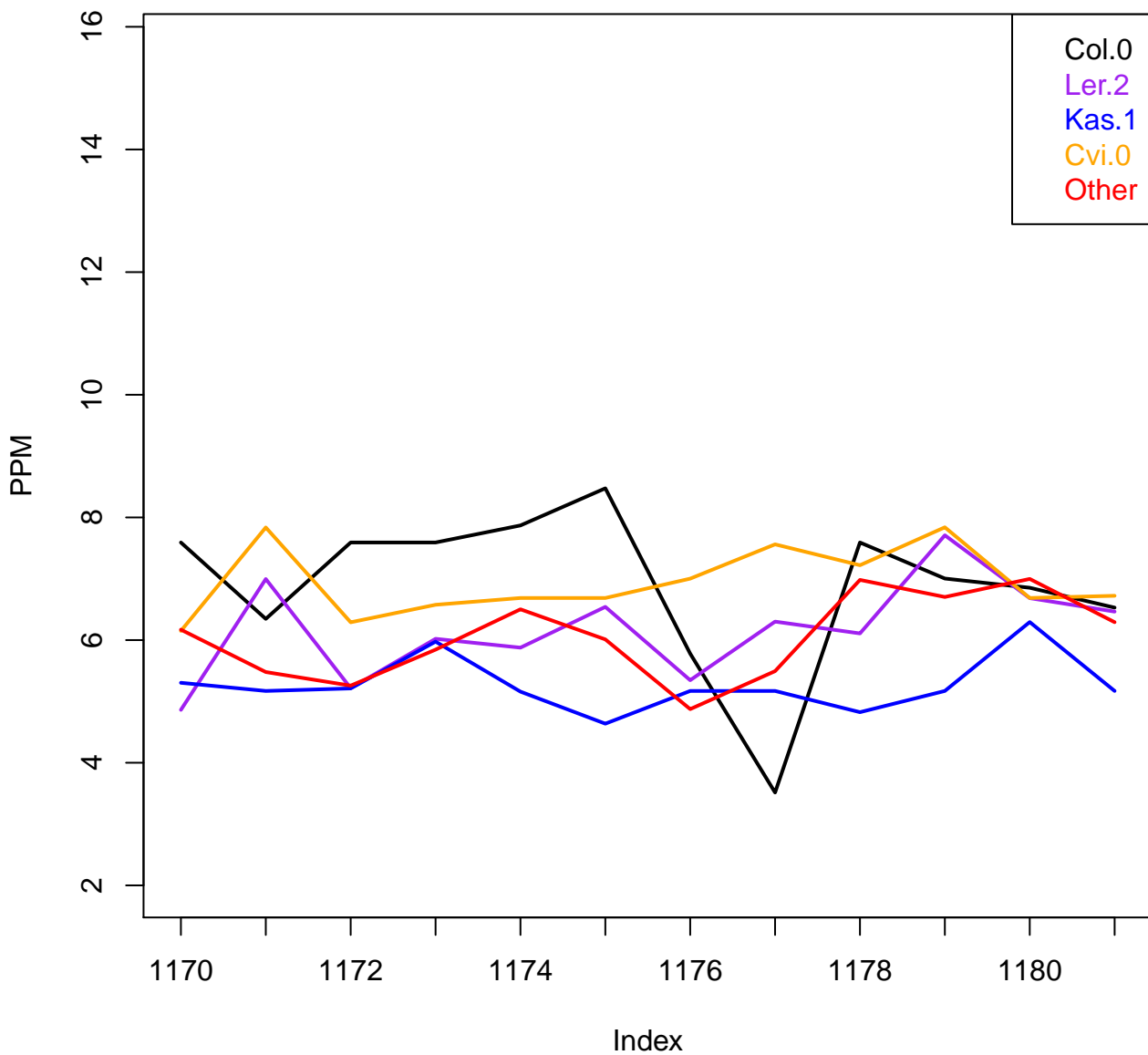

# Rb85

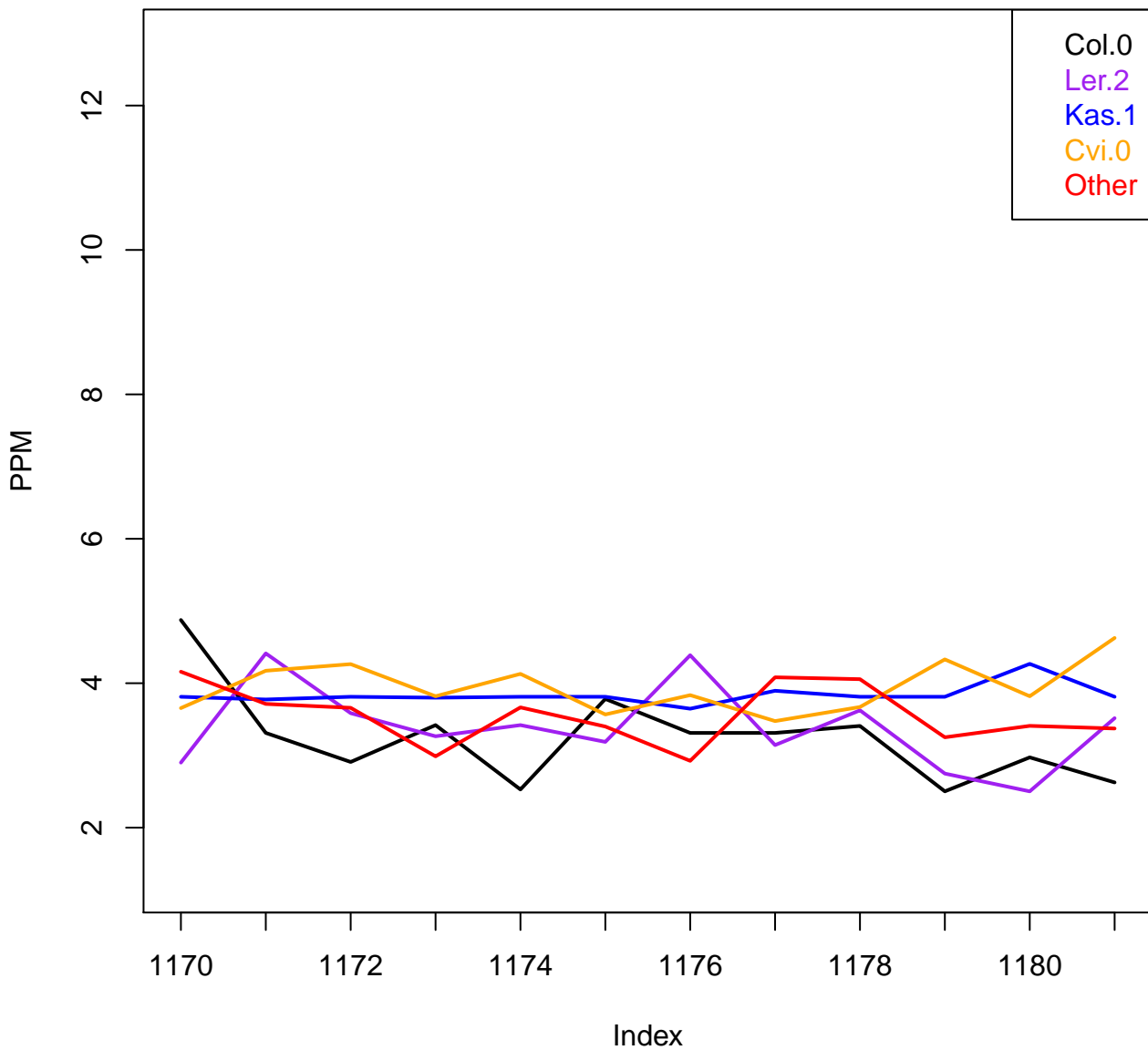

# Mo98

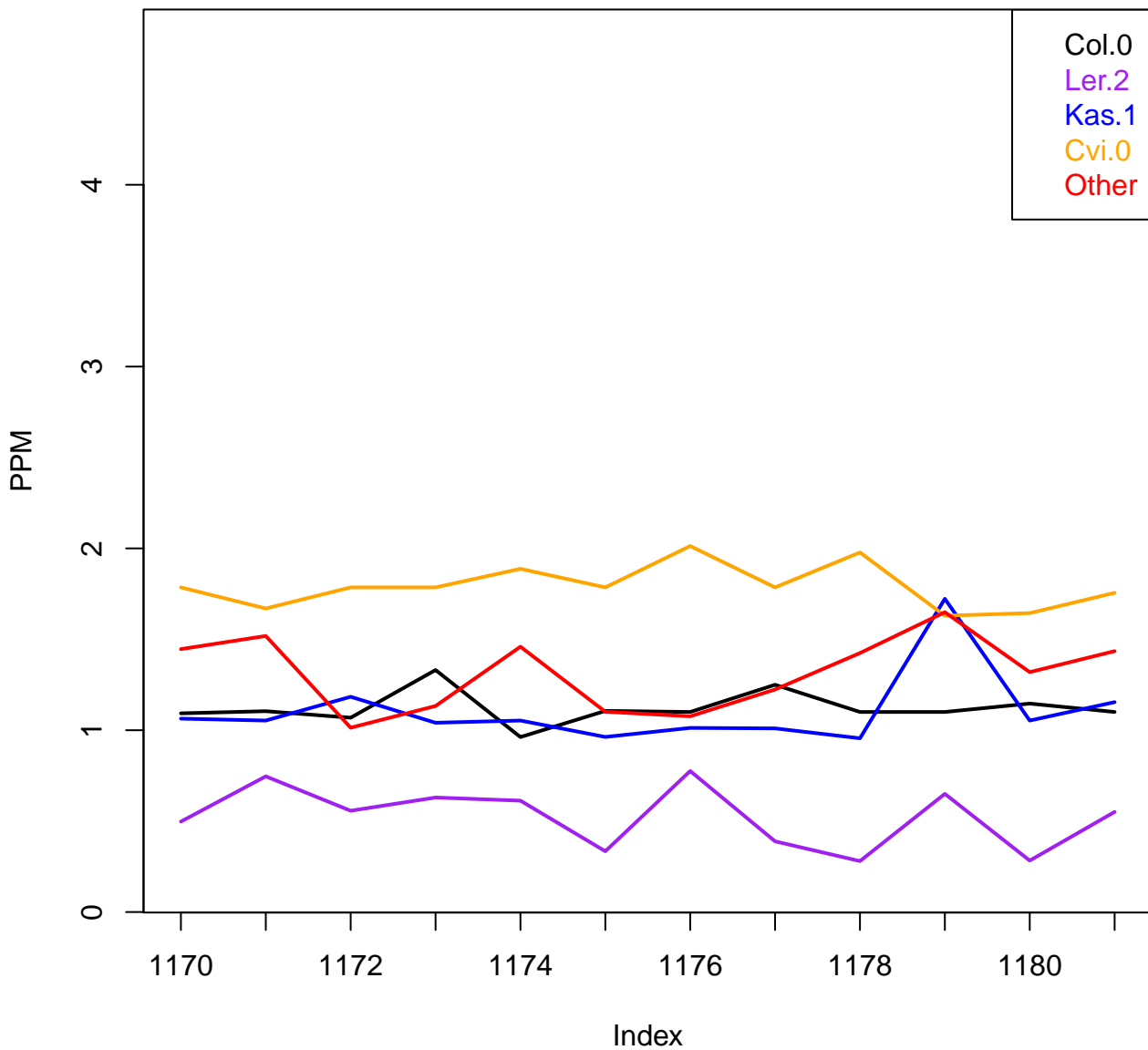

# Cd114

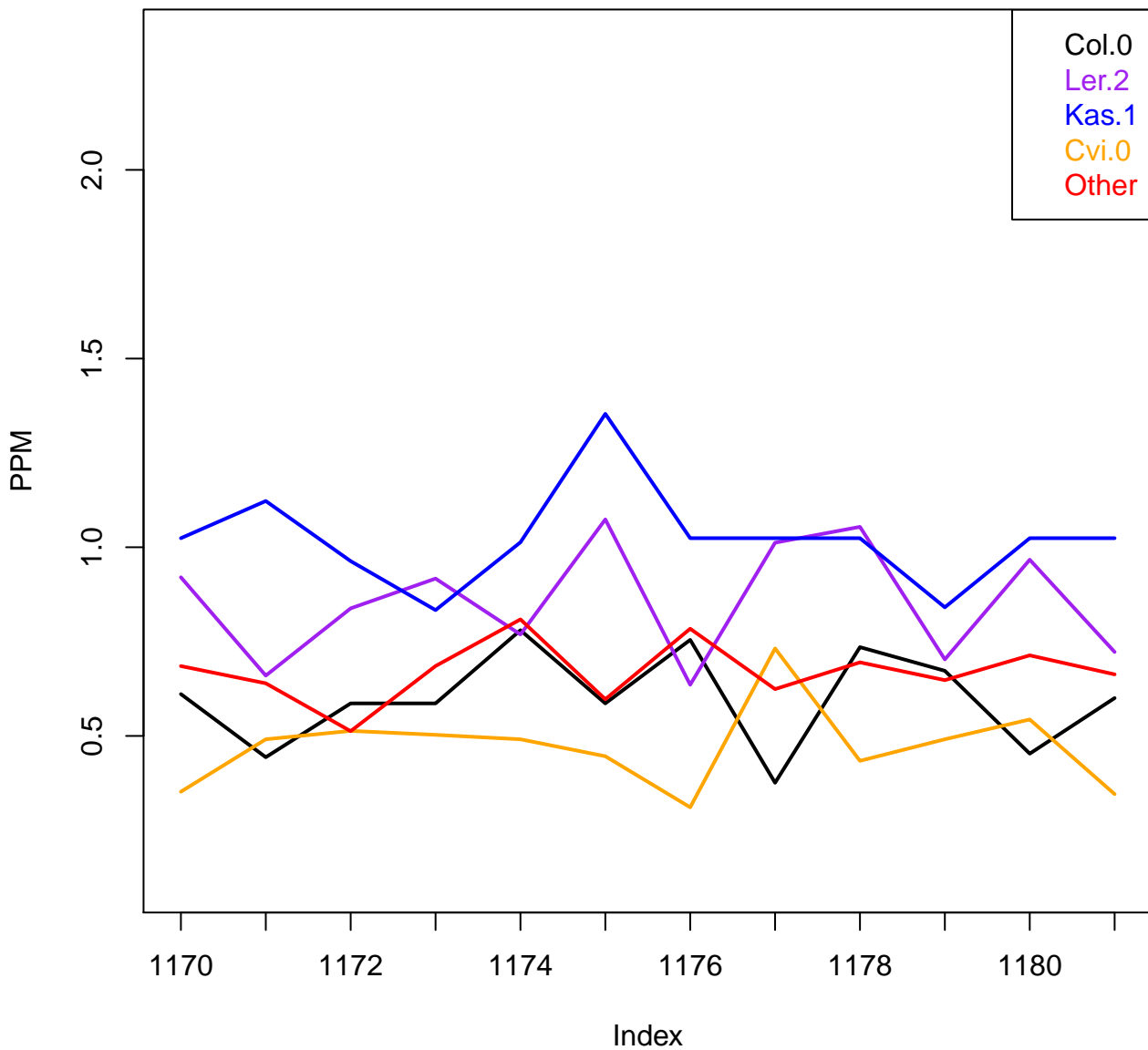

Supplement: Figure S7 — Plot of control line averages for each tray after normalization for Seed experiment. All non-control lines are averaged into the “Other” line. (PDF) [file pone.0035121.s009.pdf]
