# Supplementary material for: Biodiversity of Mineral Nutrient and Trace Element Accumulation in Arabidopsis thaliana
Source: PLoS One. 2012 Apr 27;7(4):e35121. doi: 10.1371/journal.pone.0035121 (PMC3338729; doi:10.1371/journal.pone.0035121)
Supplement: Figure S1 — Histograms of line averages of weight normalized PPM data from each experiment for each element. (PDF) [file pone.0035121.s003.pdf]

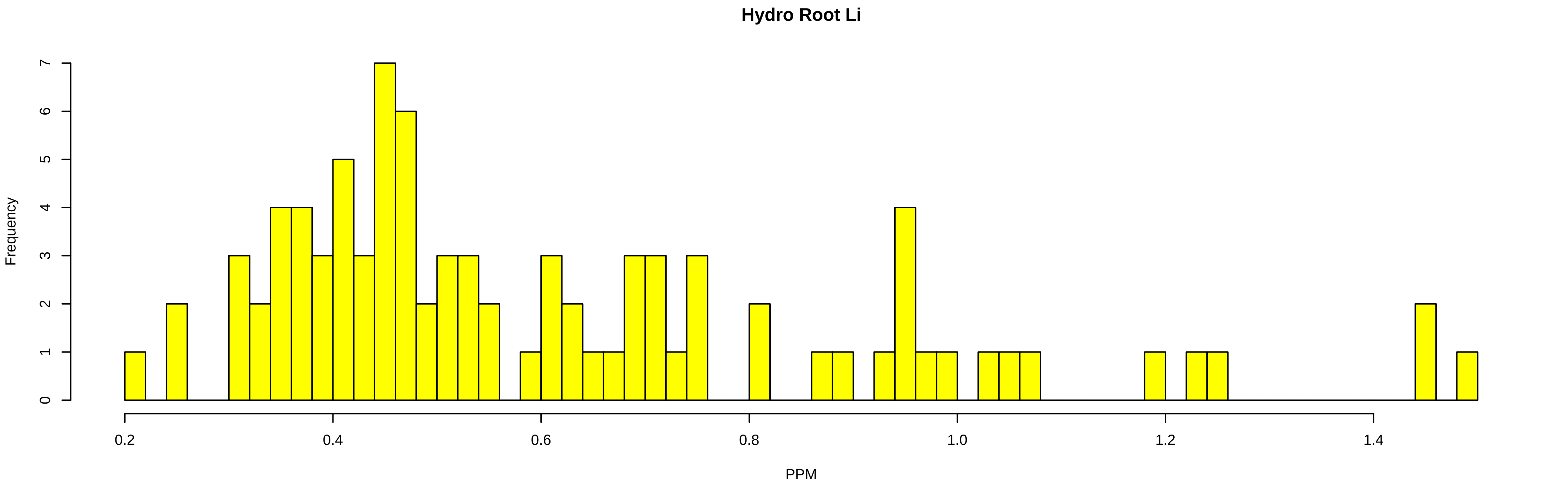
Hydro Root Li
Frequency
PPM
0.2
0.4
0.6
0.8
1.0
1.2
1.4

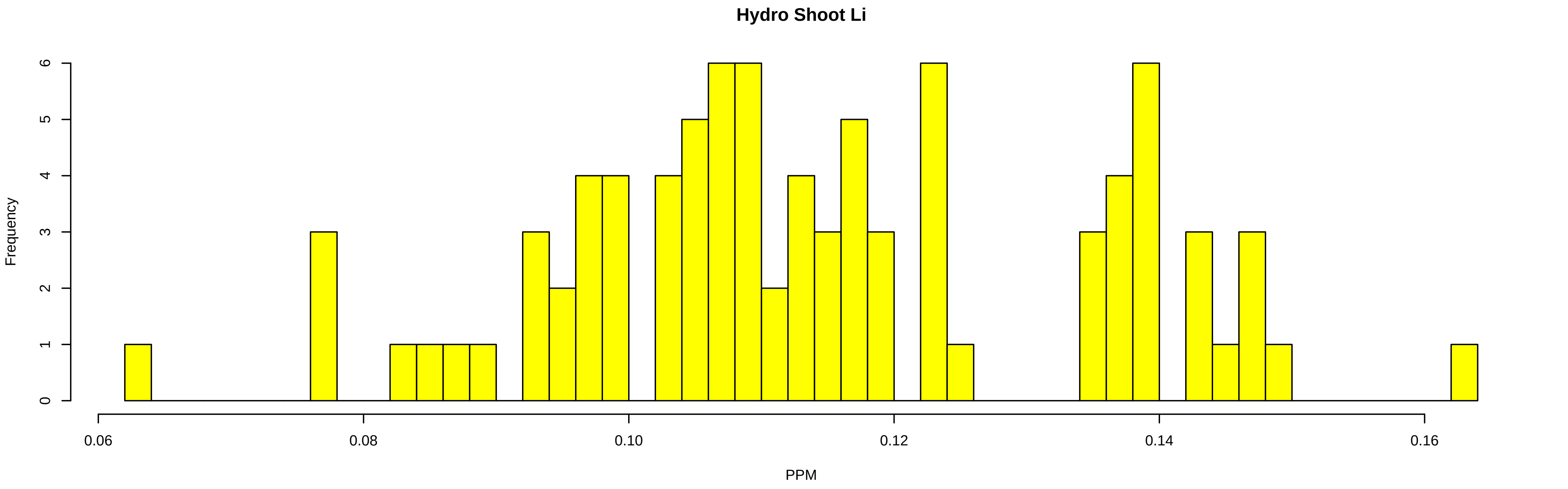
Hydro Shoot Li
Frequency
PPM
0.06
0.08
0.10
0.12
0.14
0.16

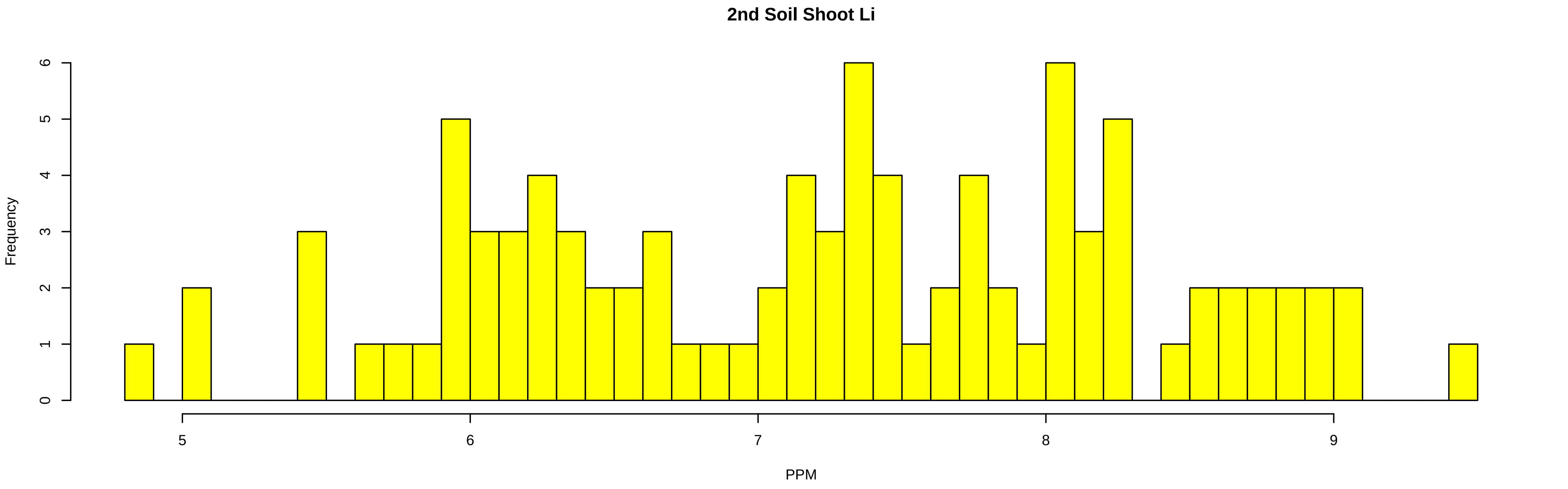
2nd Soil Shoot Li
Frequency
PPM
5
6
7
8
9

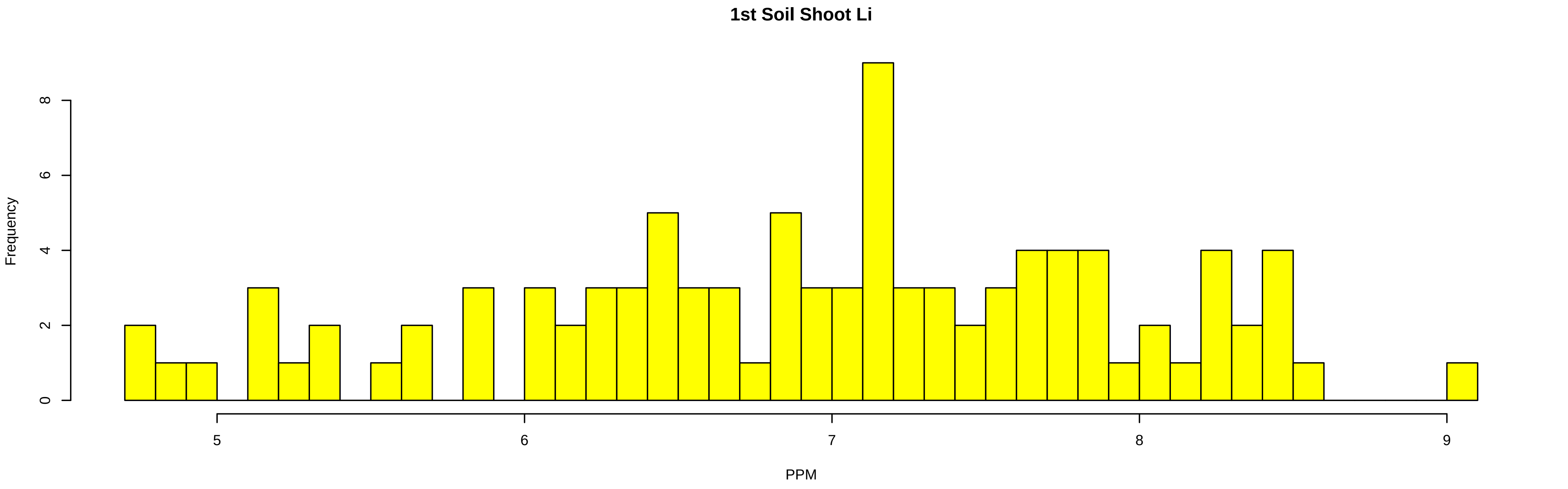
1st Soil Shoot Li
Frequency
PPM
5
6
7
8
9

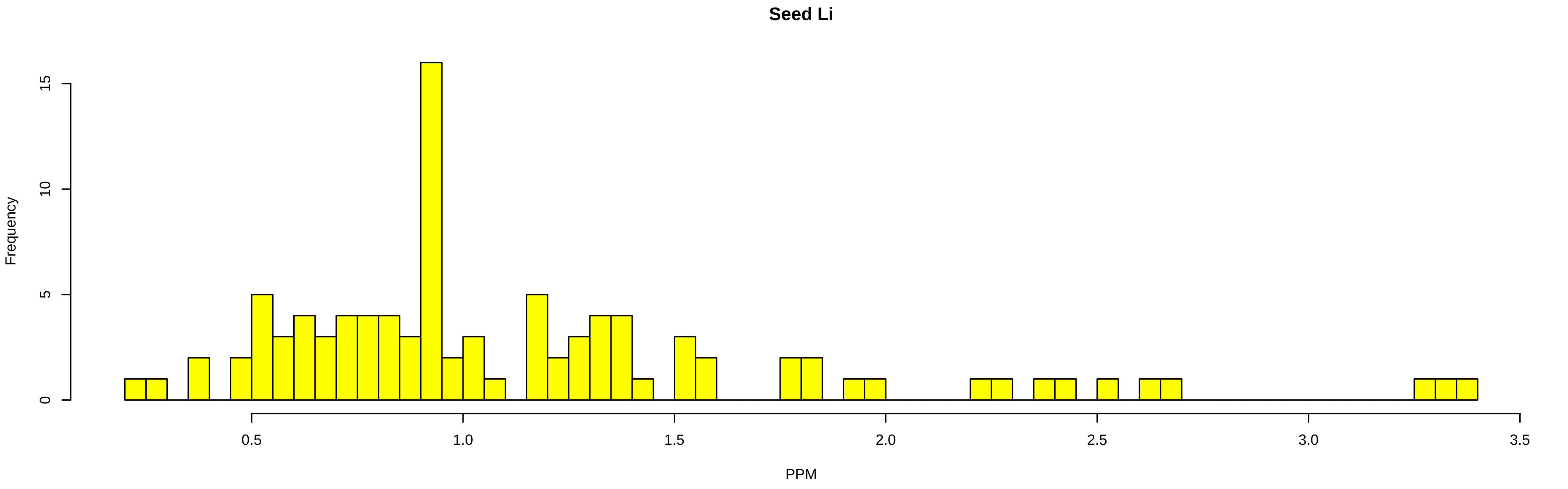
Seed Li
Frequency
PPM
0.5
1.0
1.5
2.0
2.5
3.0
3.5

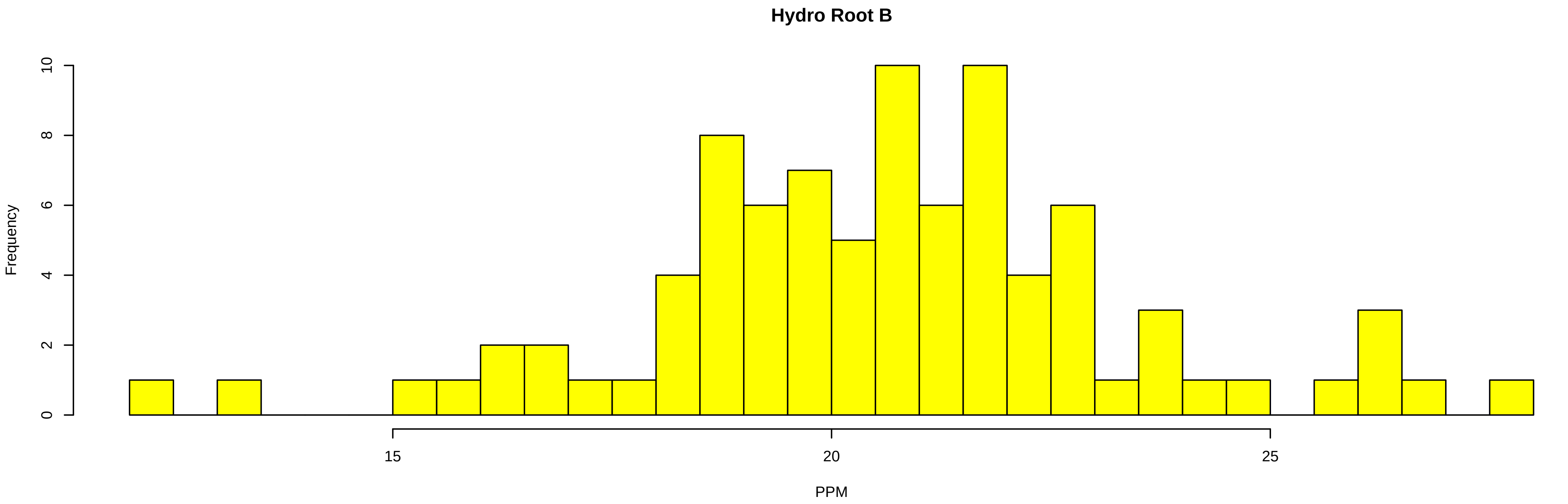
Hydro Root B
Frequency
0
2
4
6
8
10
15
20
25
PPM

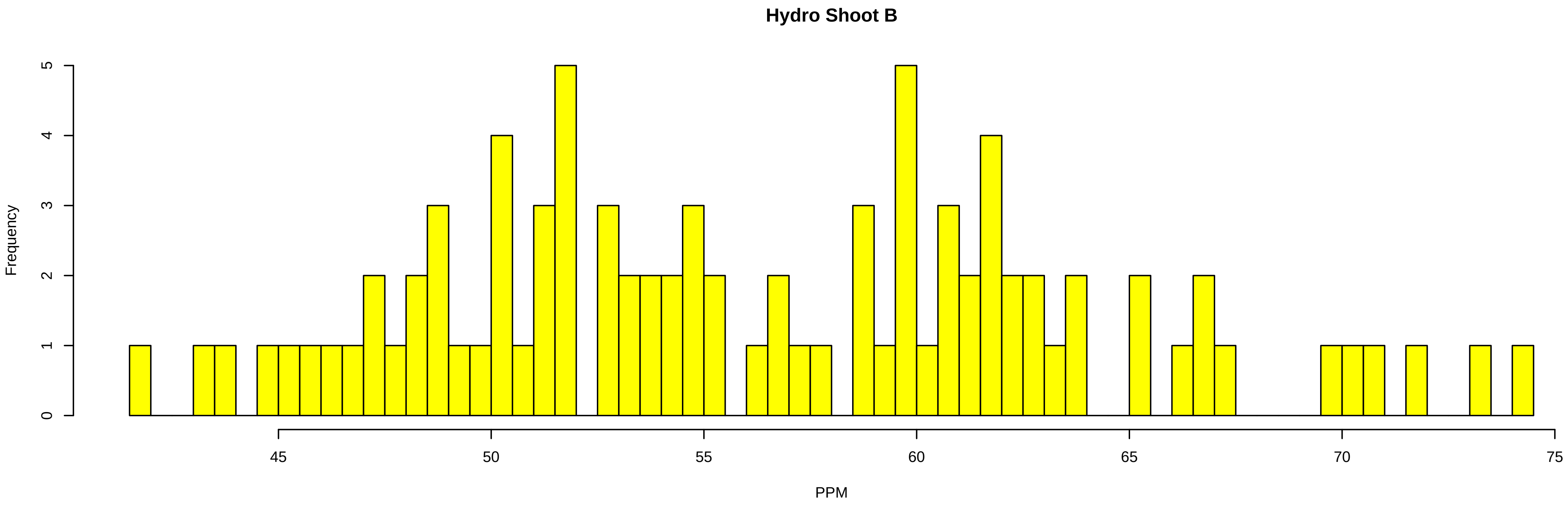
Hydro Shoot B
Frequency
0
1
2
3
4
5
45
50
55
60
65
70
75
PPM

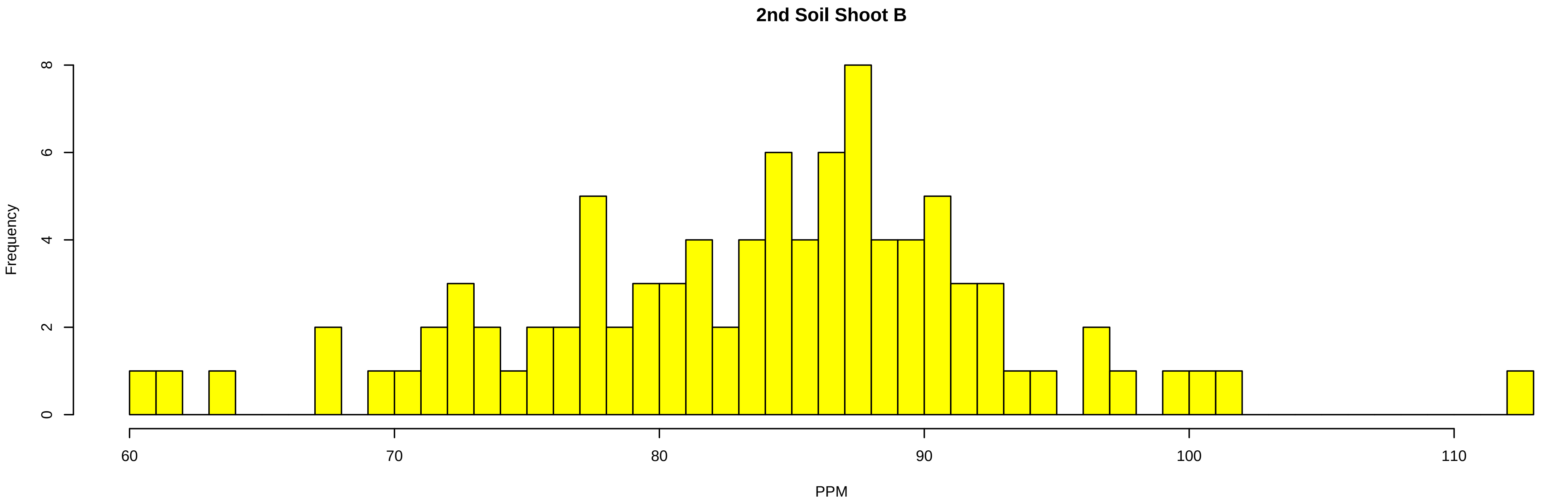
2nd Soil Shoot B
Frequency
0
2
4
6
8
60
70
80
90
100
110
PPM

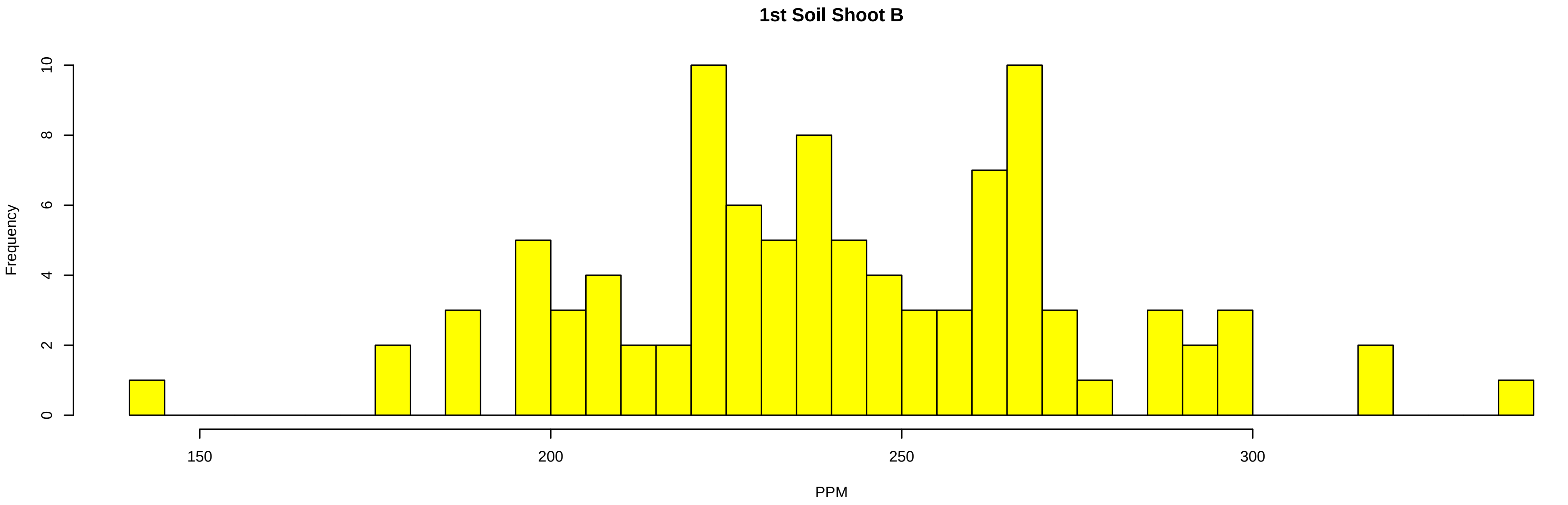
1st Soil Shoot B
Frequency
0
2
4
6
8
10
150
200
250
300
PPM

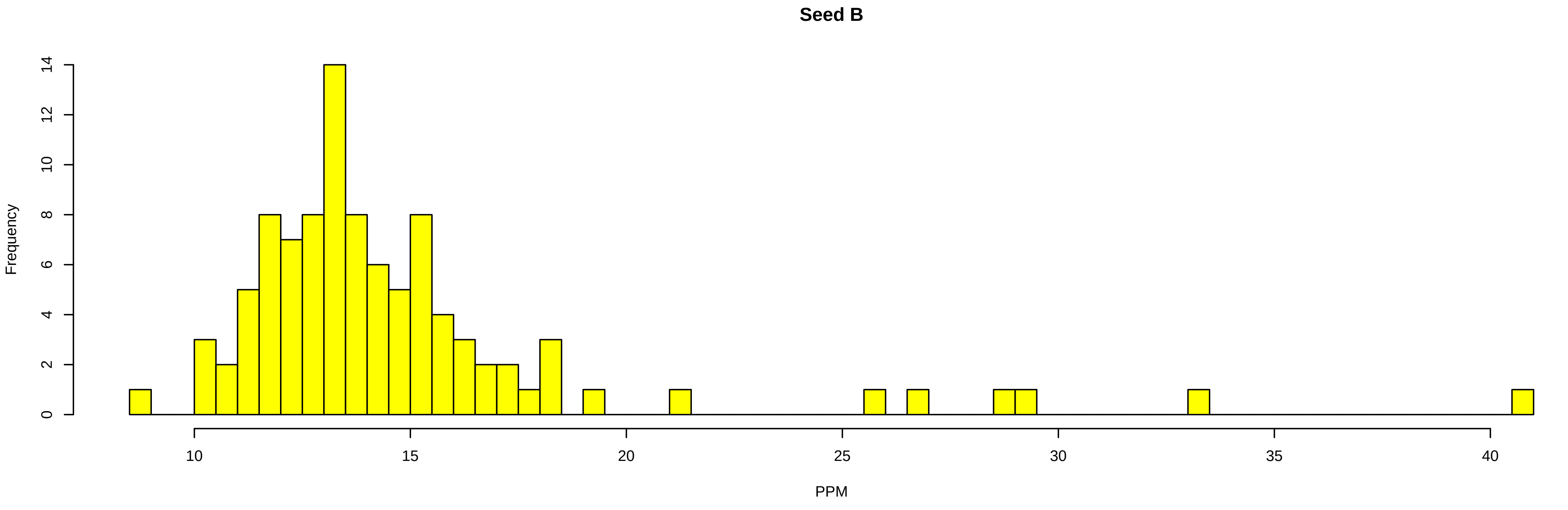
Seed B
Frequency
0
2
4
6
8
10
12
14
10
15
20
25
30
35
40
PPM

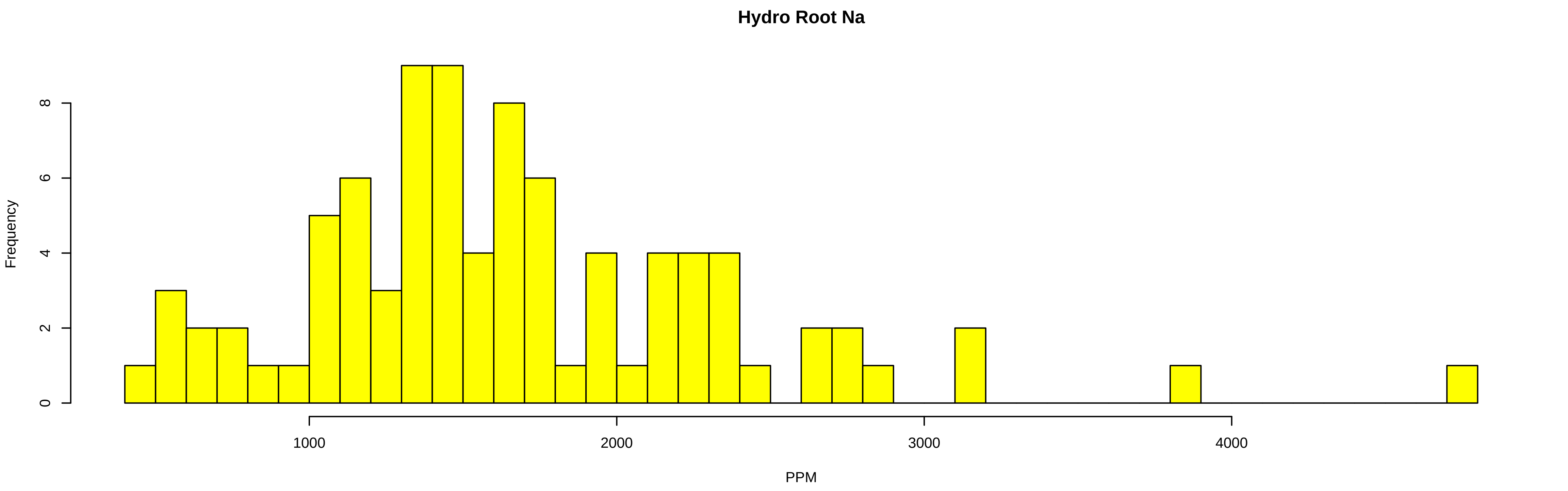
Hydro Root Na
Frequency
0
2
4
6
8
1000
2000
3000
4000
PPM

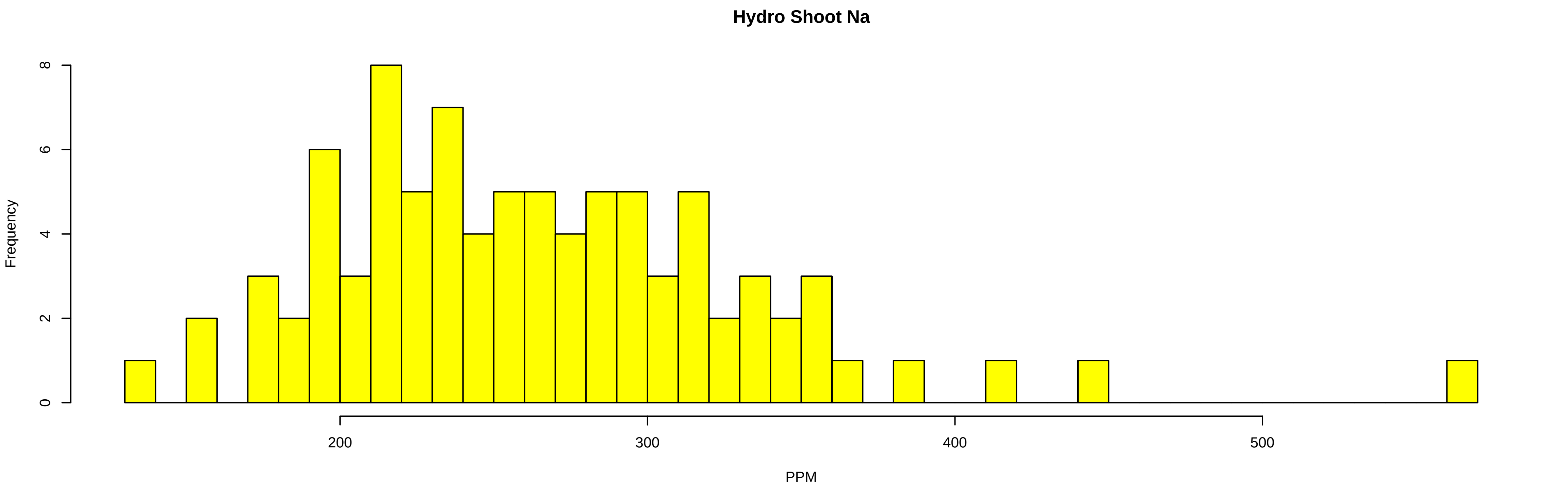
Hydro Shoot Na
Frequency
0
2
4
6
8
200
300
400
500
PPM

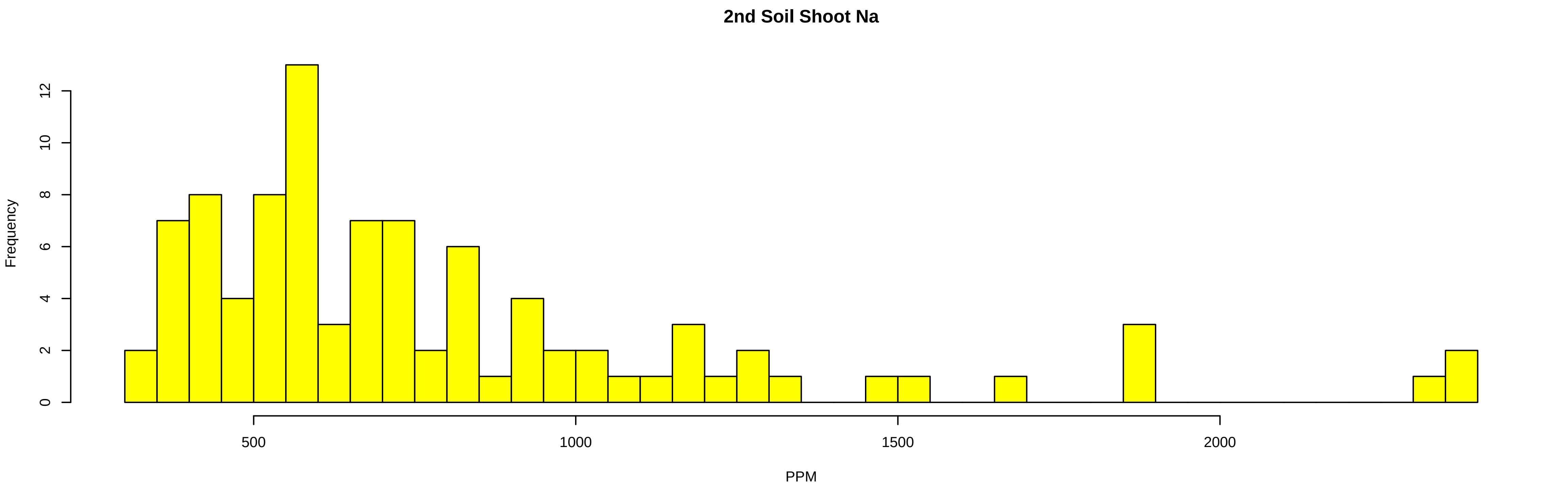
2nd Soil Shoot Na
Frequency
0
2
4
6
8
10
12
500
1000
1500
2000
PPM

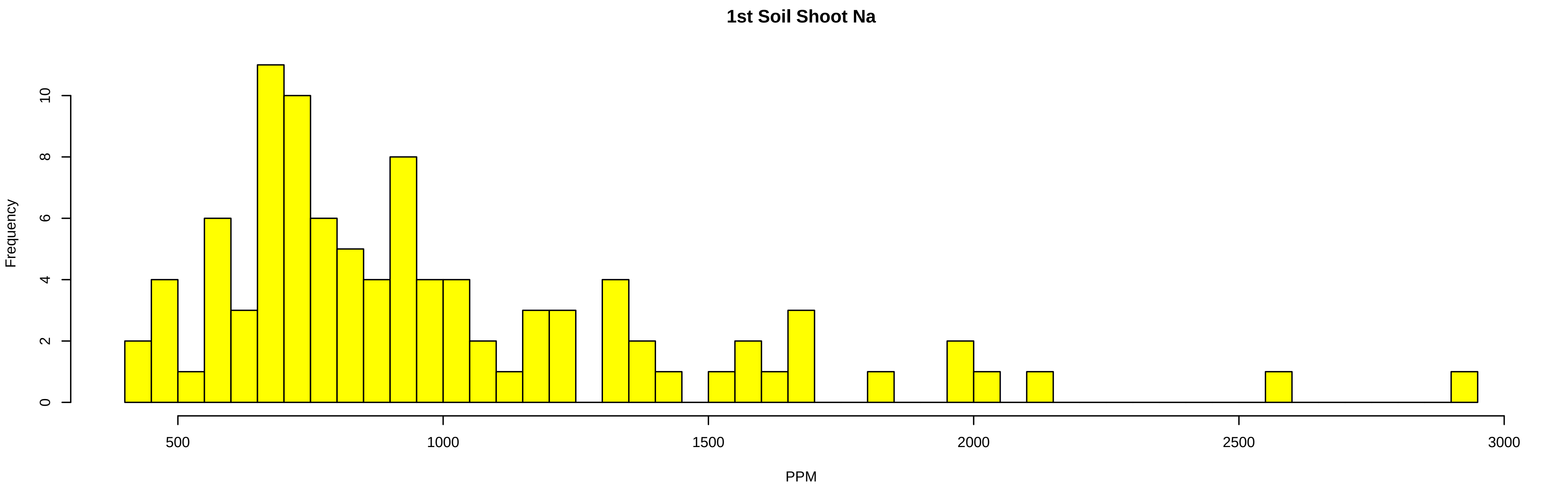
1st Soil Shoot Na
Frequency
0
2
4
6
8
10
500
1000
1500
2000
2500
3000
PPM

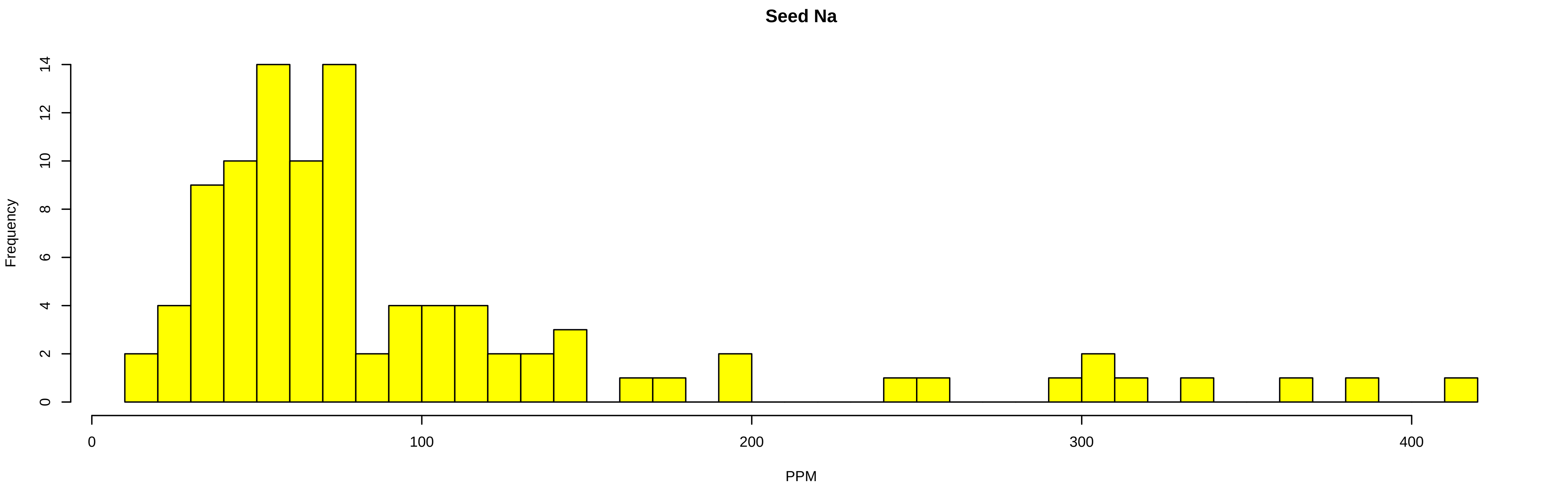
Seed Na
Frequency
0
2
4
6
8
10
12
14
0
100
200
300
400
PPM

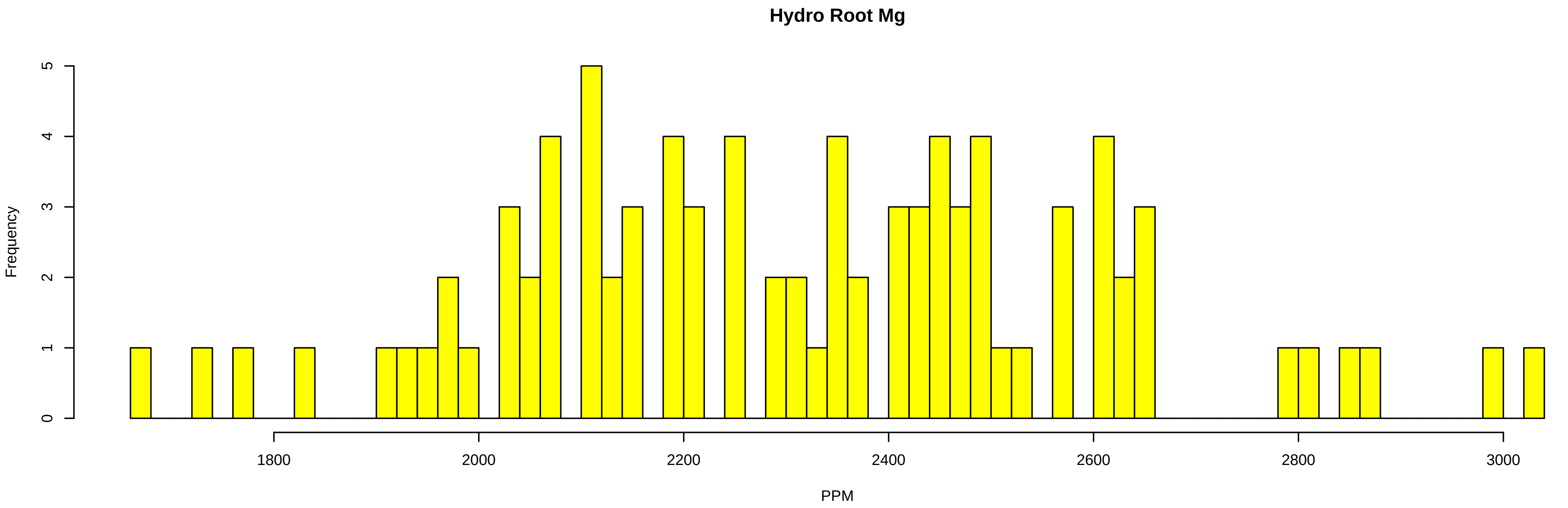
Hydro Root Mg
Frequency
PPM
1800
2000
2200
2400
2600
2800
3000
0
1
2
3
4
5

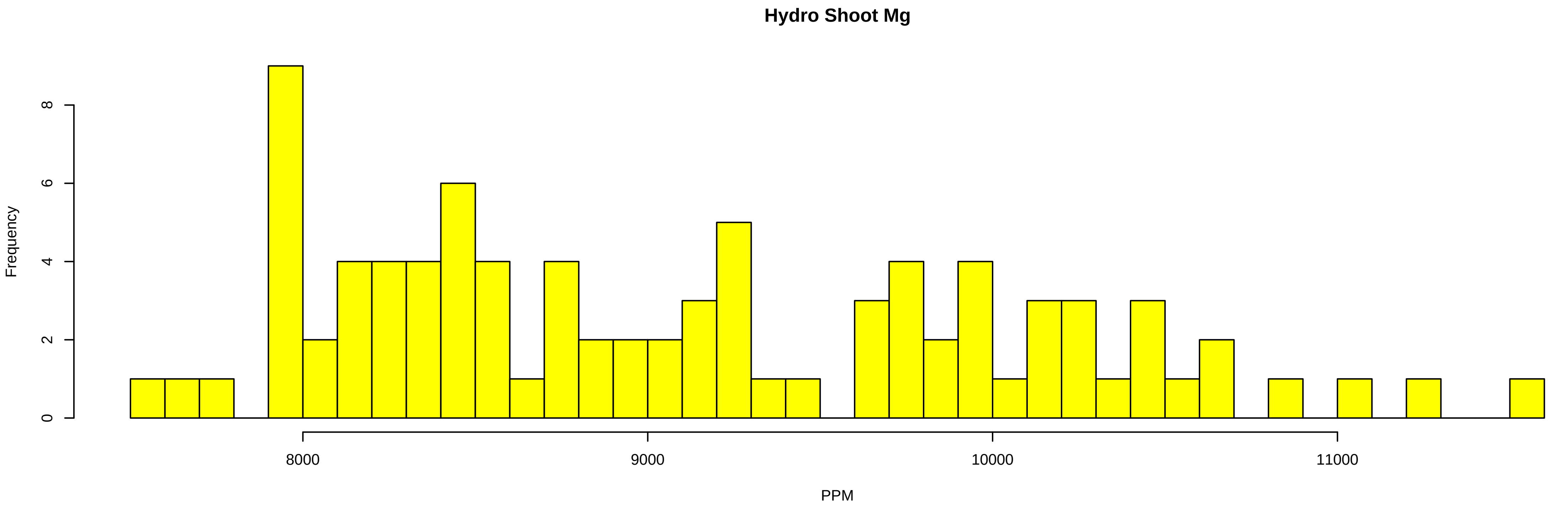
Hydro Shoot Mg
Frequency
PPM
8000
9000
10000
11000
0
2
4
6
8

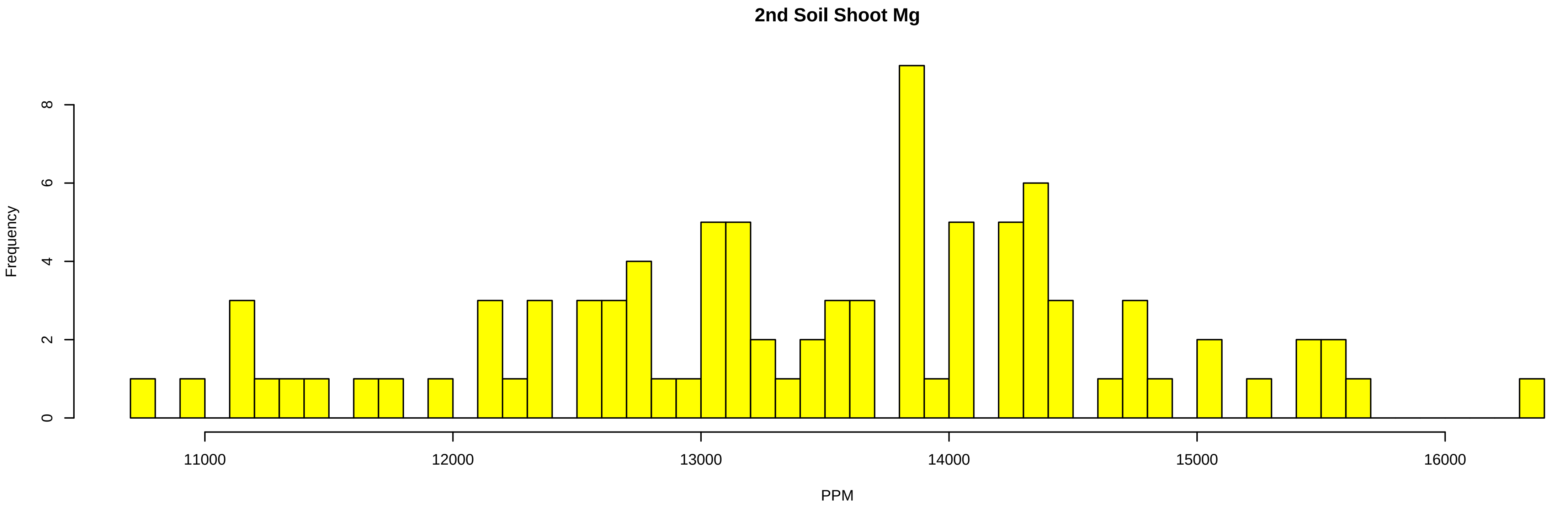
2nd Soil Shoot Mg
Frequency
PPM
11000
12000
13000
14000
15000
16000
0
2
4
6
8

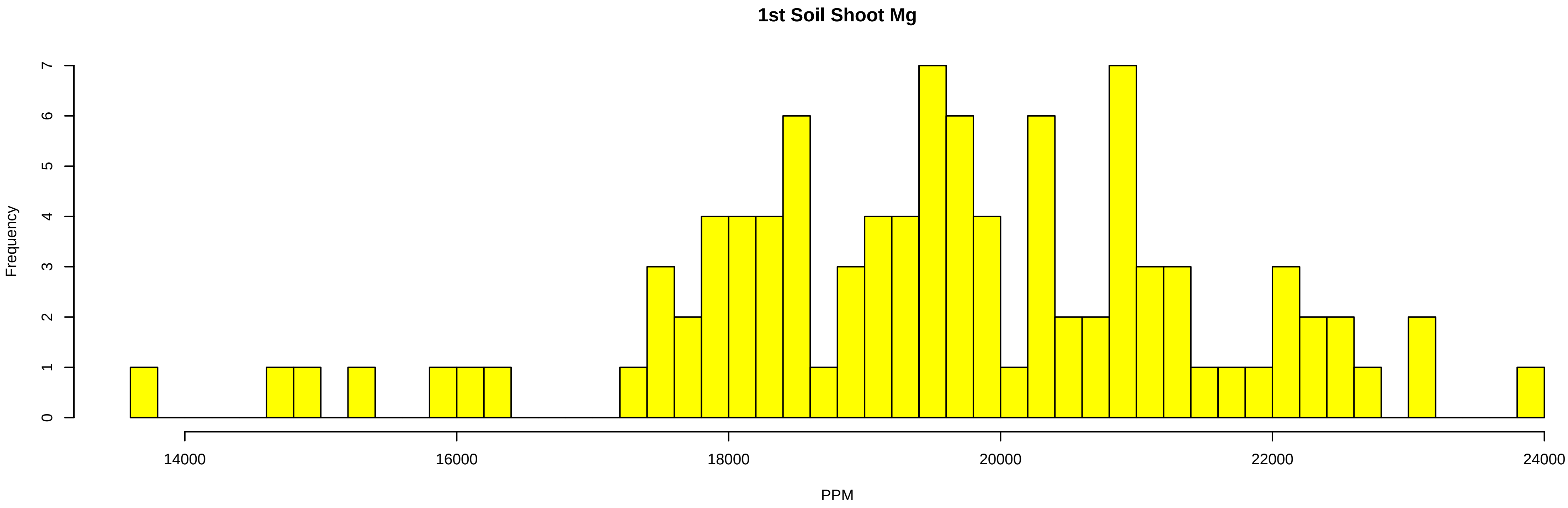
1st Soil Shoot Mg
Frequency
PPM
14000
16000
18000
20000
22000
24000
0
1
2
3
4
5
6
7

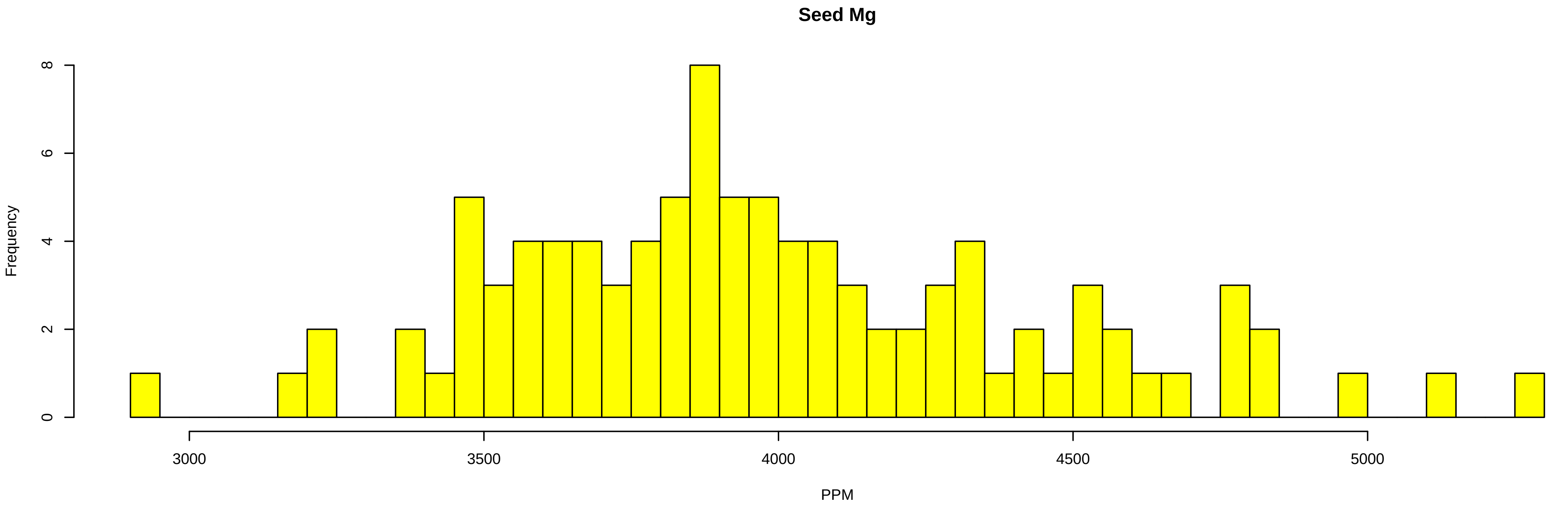
Seed Mg
Frequency
PPM
3000
3500
4000
4500
5000
0
2
4
6
8

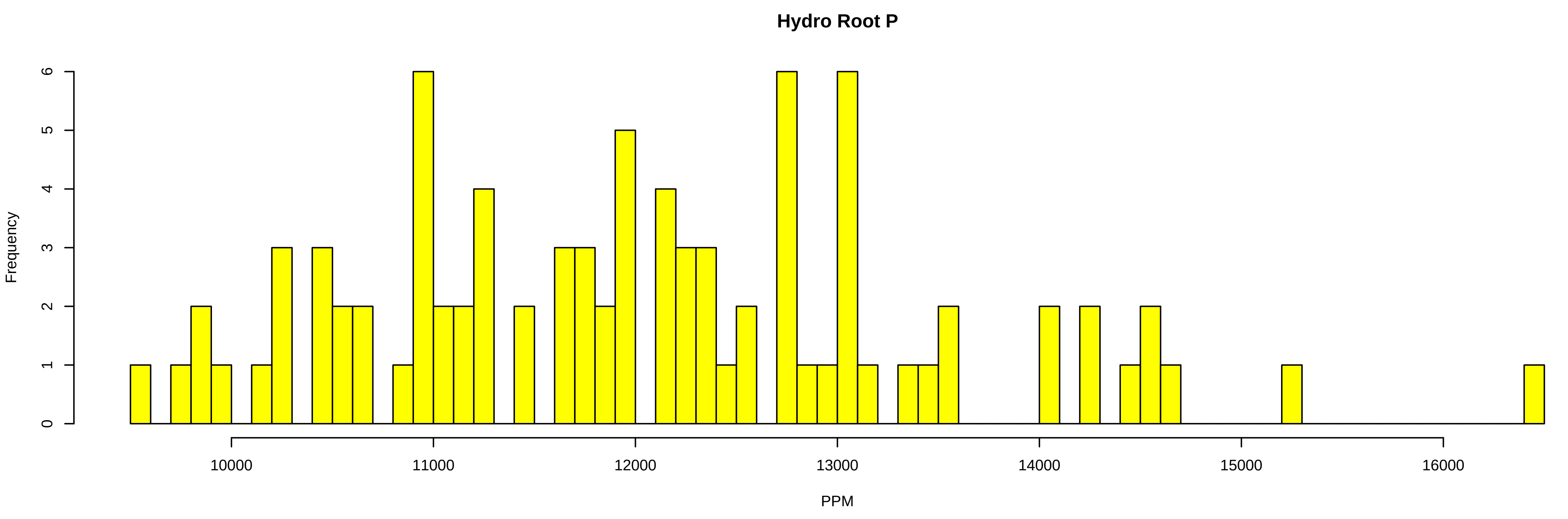
Hydro Root P
Frequency
0 1 2 3 4 5 6
10000 11000 12000 13000 14000 15000 16000
PPM

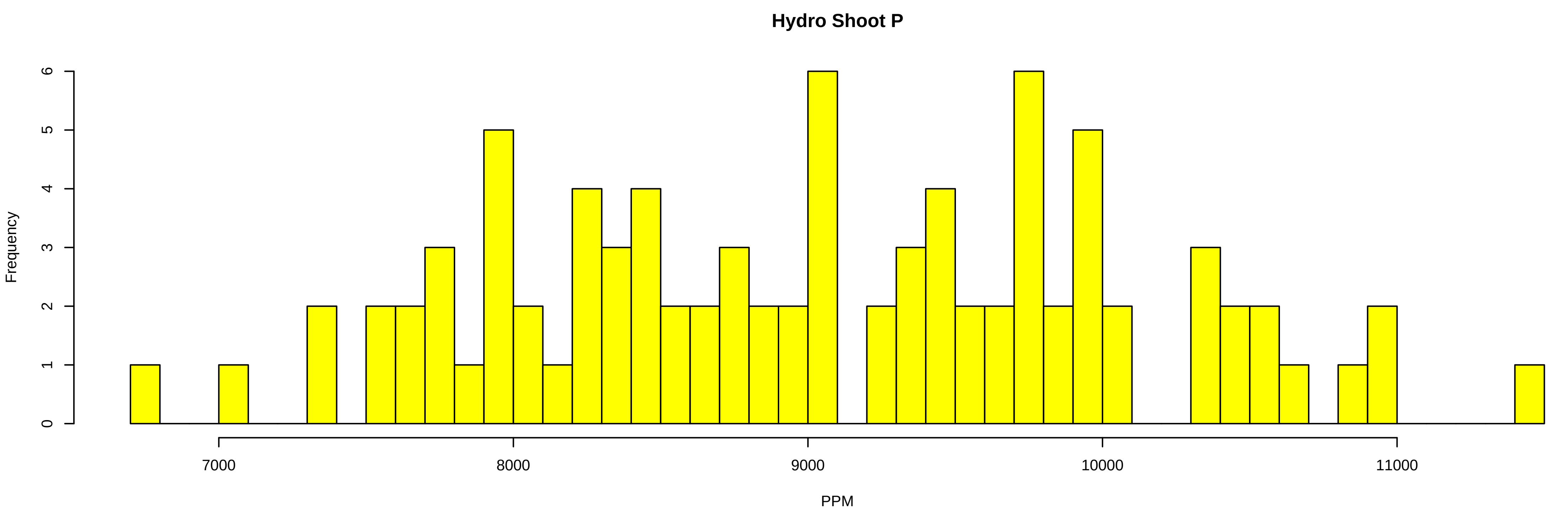
Hydro Shoot P
Frequency
0 1 2 3 4 5 6
7000 8000 9000 10000 11000
PPM

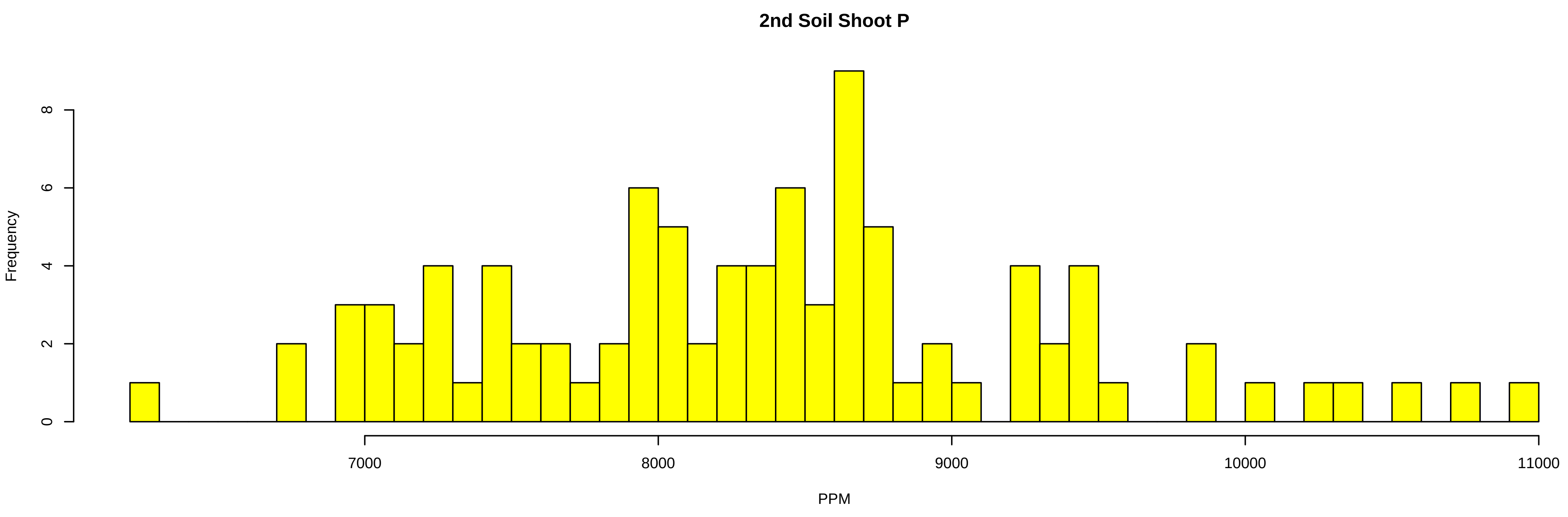
2nd Soil Shoot P
Frequency
0 2 4 6 8
7000 8000 9000 10000 11000
PPM

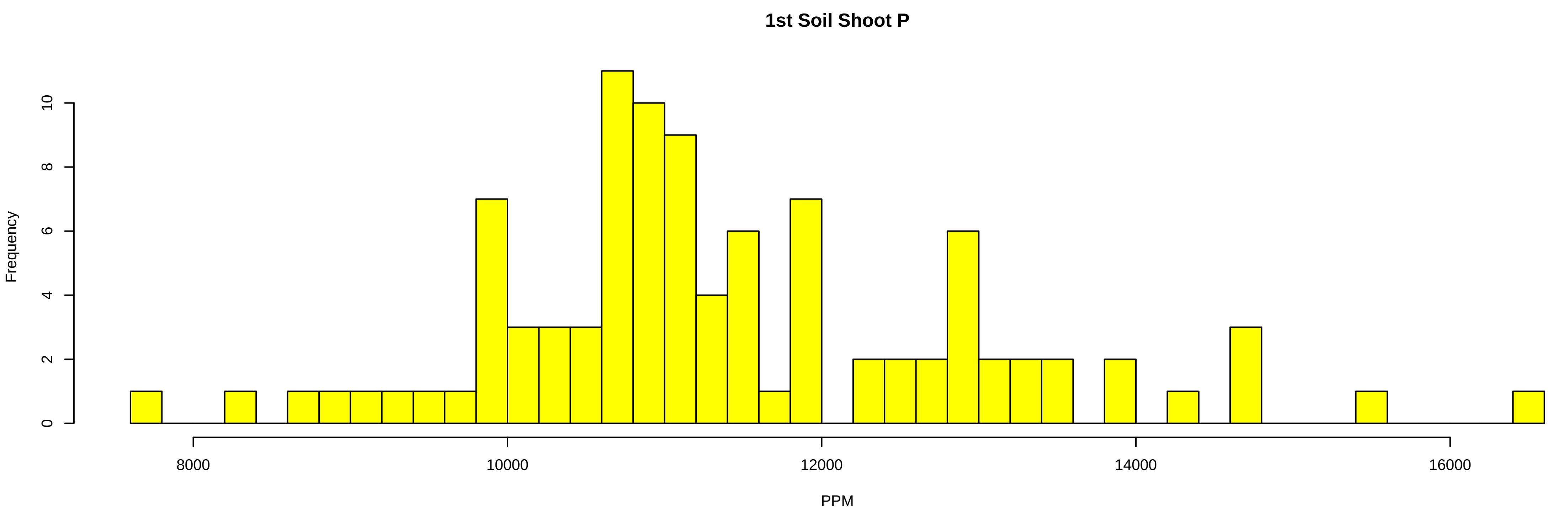
1st Soil Shoot P
Frequency
0 2 4 6 8 10
8000 10000 12000 14000 16000
PPM

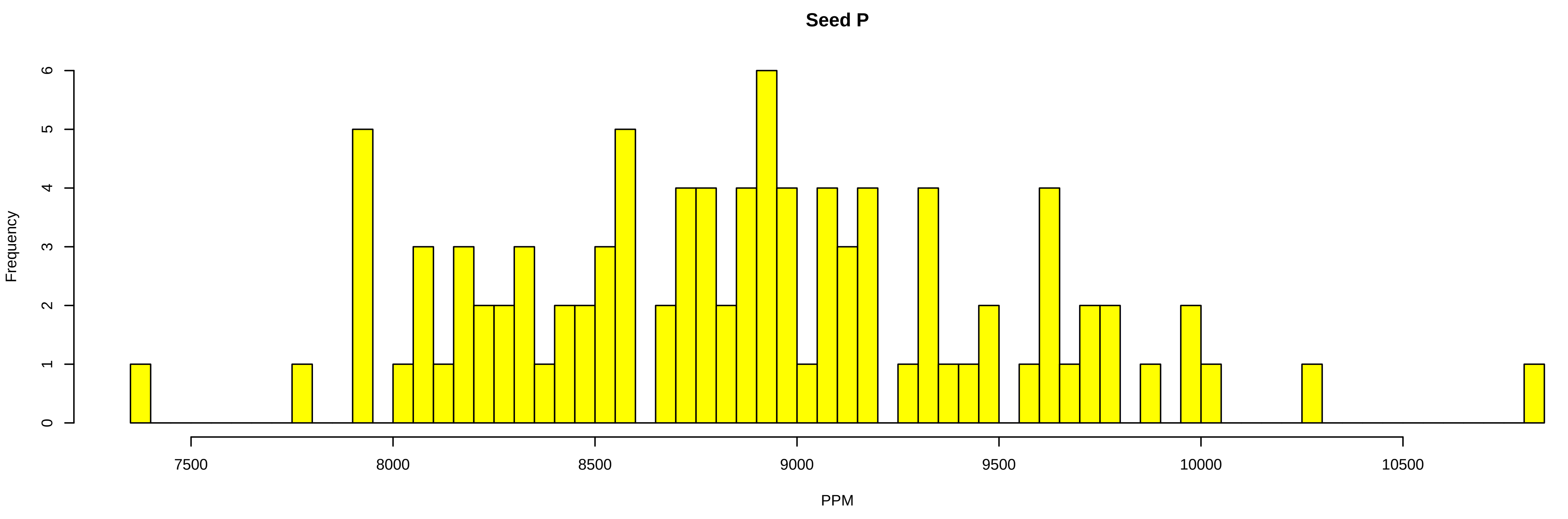
Seed P
Frequency
0 1 2 3 4 5 6
7500 8000 8500 9000 9500 10000 10500
PPM

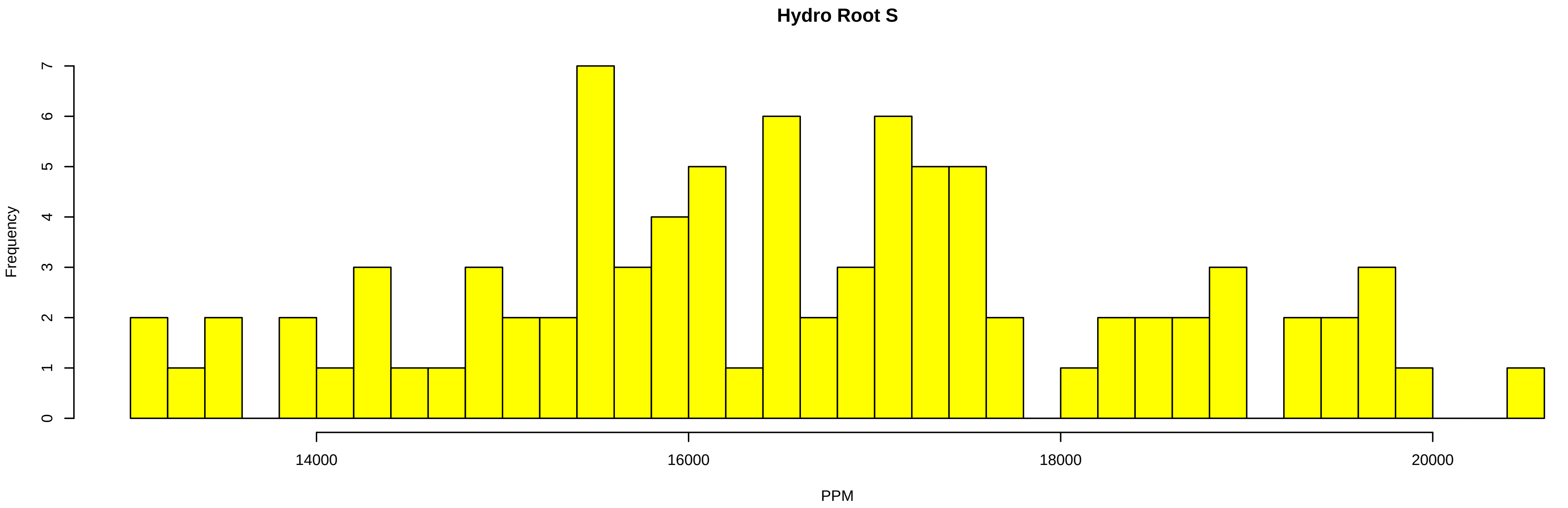
Hydro Root S
Frequency
0
1
2
3
4
5
6
7
14000
16000
18000
20000
PPM

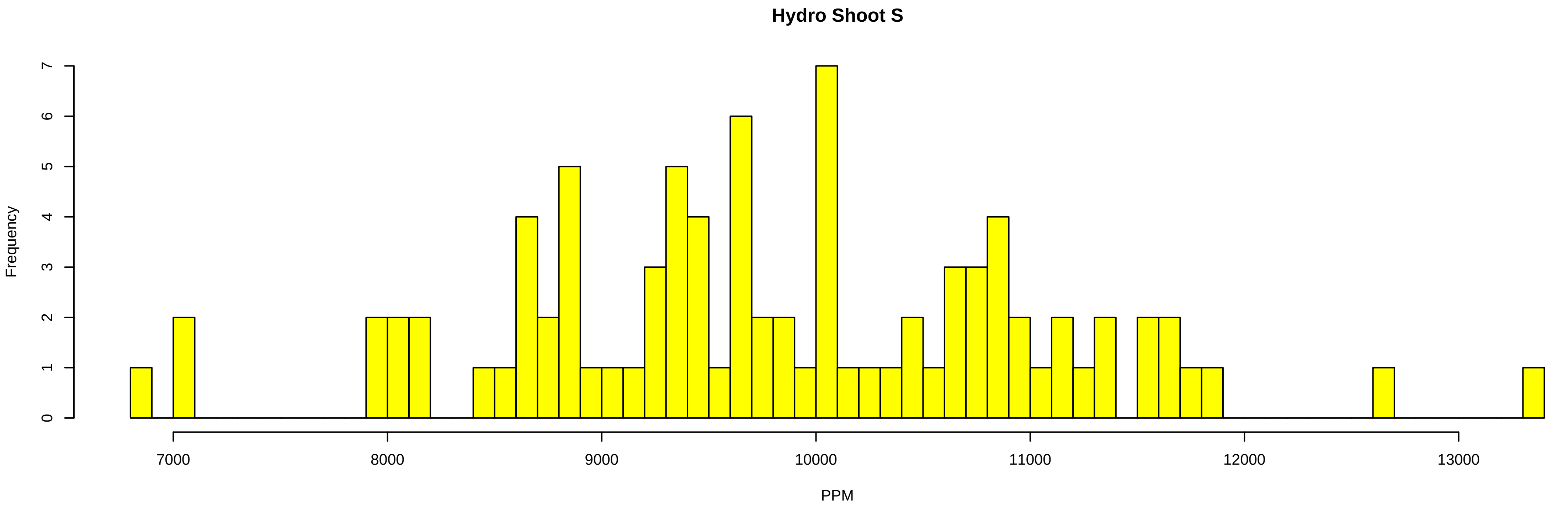
Hydro Shoot S
Frequency
0
1
2
3
4
5
6
7
7000
8000
9000
10000
11000
12000
13000
PPM

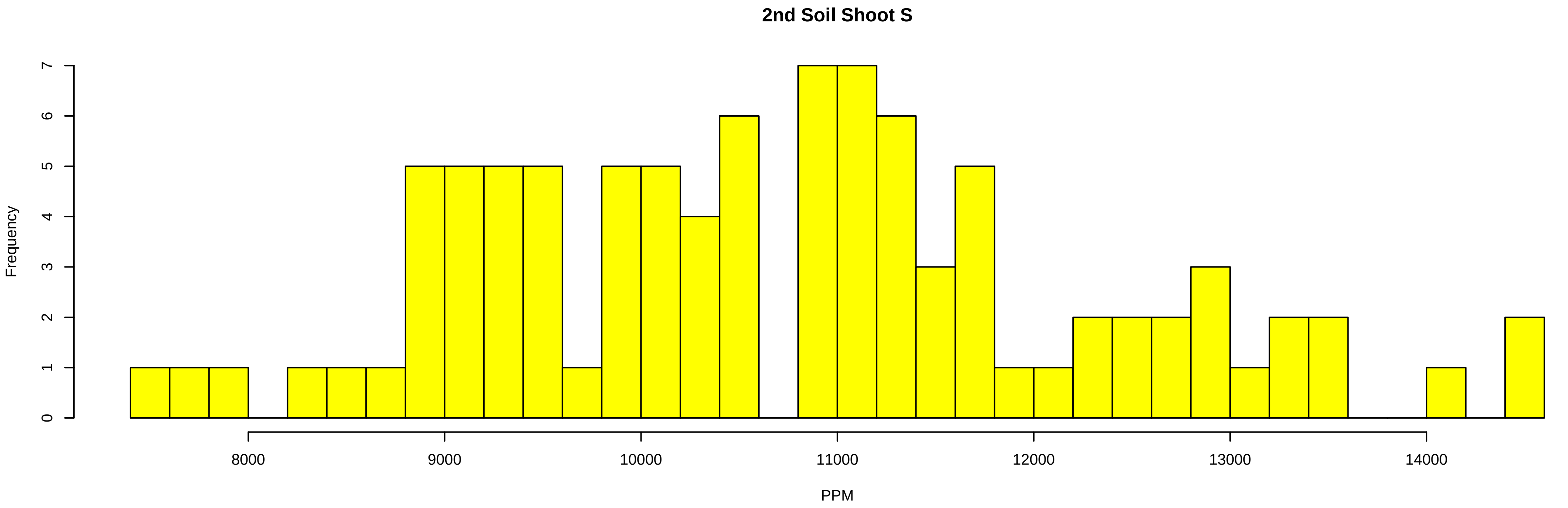
2nd Soil Shoot S
Frequency
0
1
2
3
4
5
6
7
8000
9000
10000
11000
12000
13000
14000
PPM

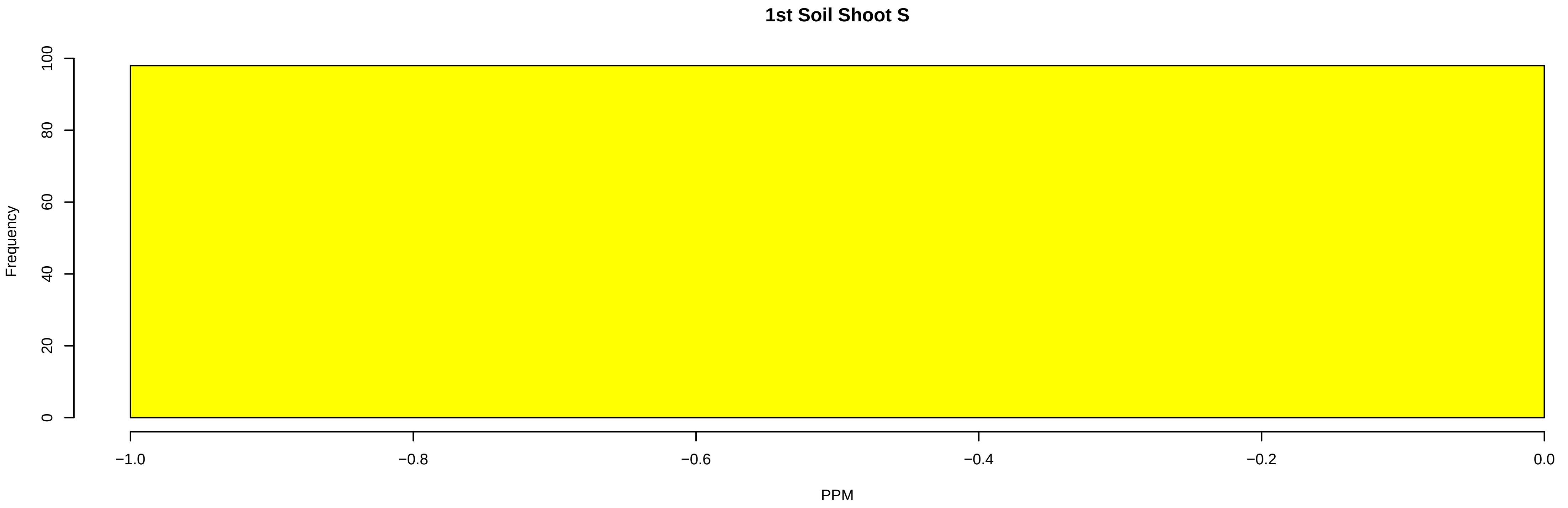
1st Soil Shoot S
Frequency
0
20
40
60
80
100
−1.0
−0.8
−0.6
−0.4
−0.2
0.0
PPM

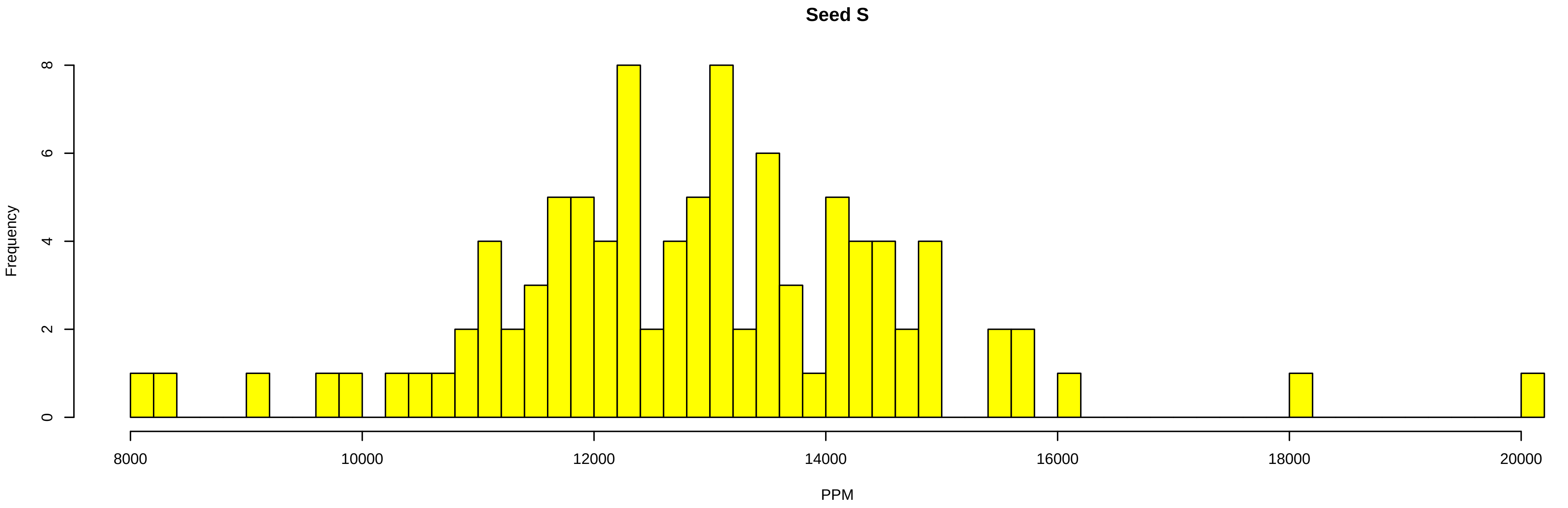
Seed S
Frequency
0
2
4
6
8
8000
10000
12000
14000
16000
18000
20000
PPM

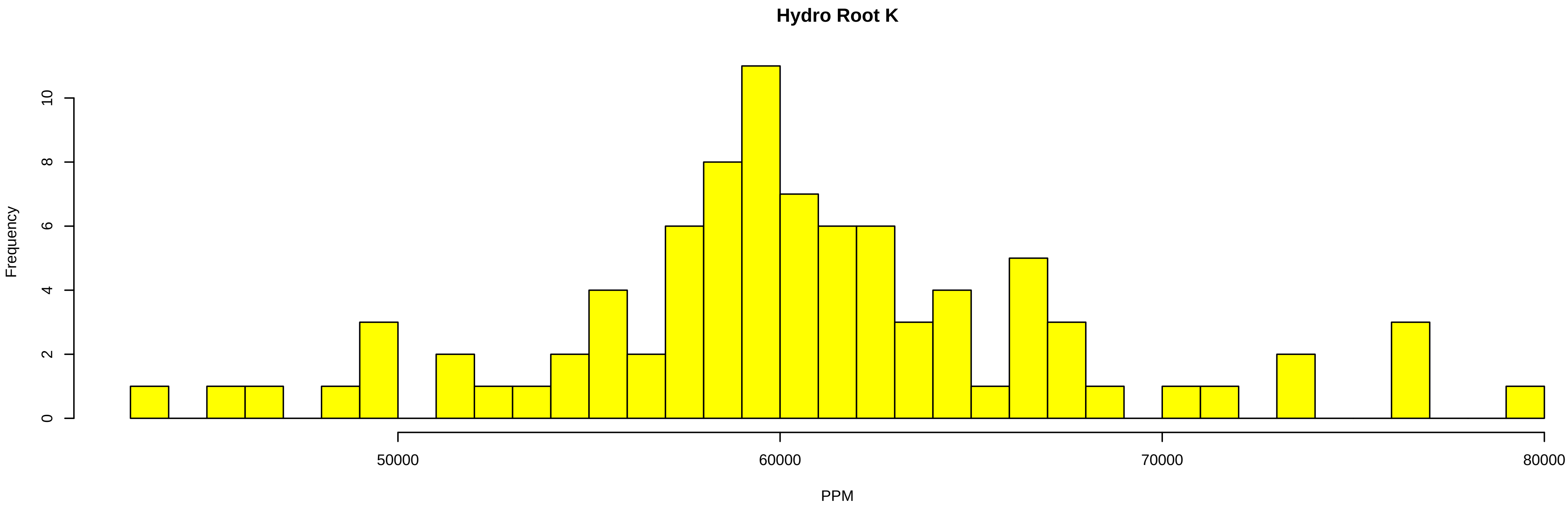

Hydro Root K
Frequency
0
2
4
6
8
10
50000
60000
70000
80000
PPM

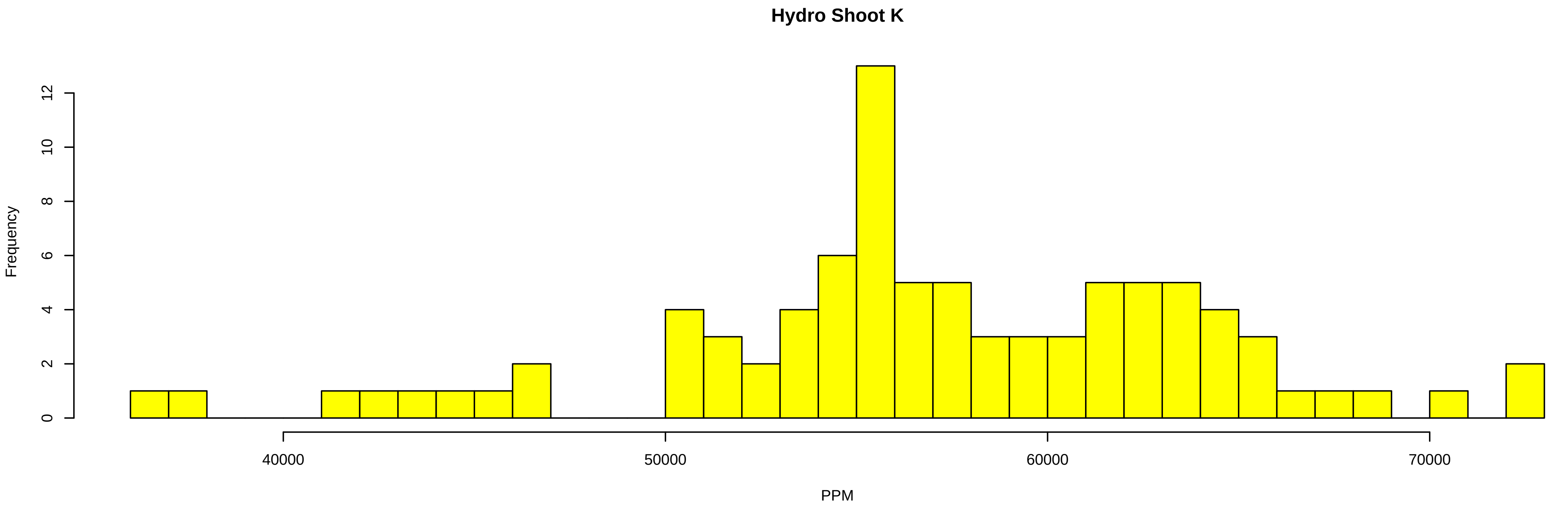

Hydro Shoot K
Frequency
0
2
4
6
8
10
12
40000
50000
60000
70000
PPM

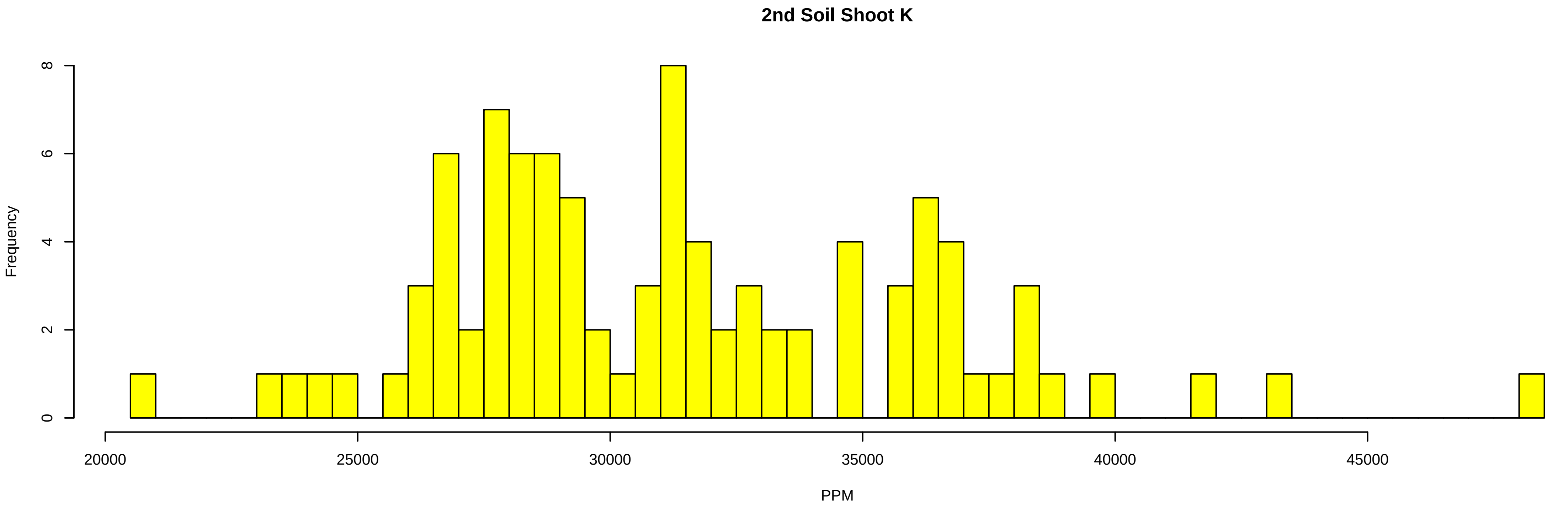

2nd Soil Shoot K
Frequency
0
2
4
6
8
20000
25000
30000
35000
40000
45000
PPM

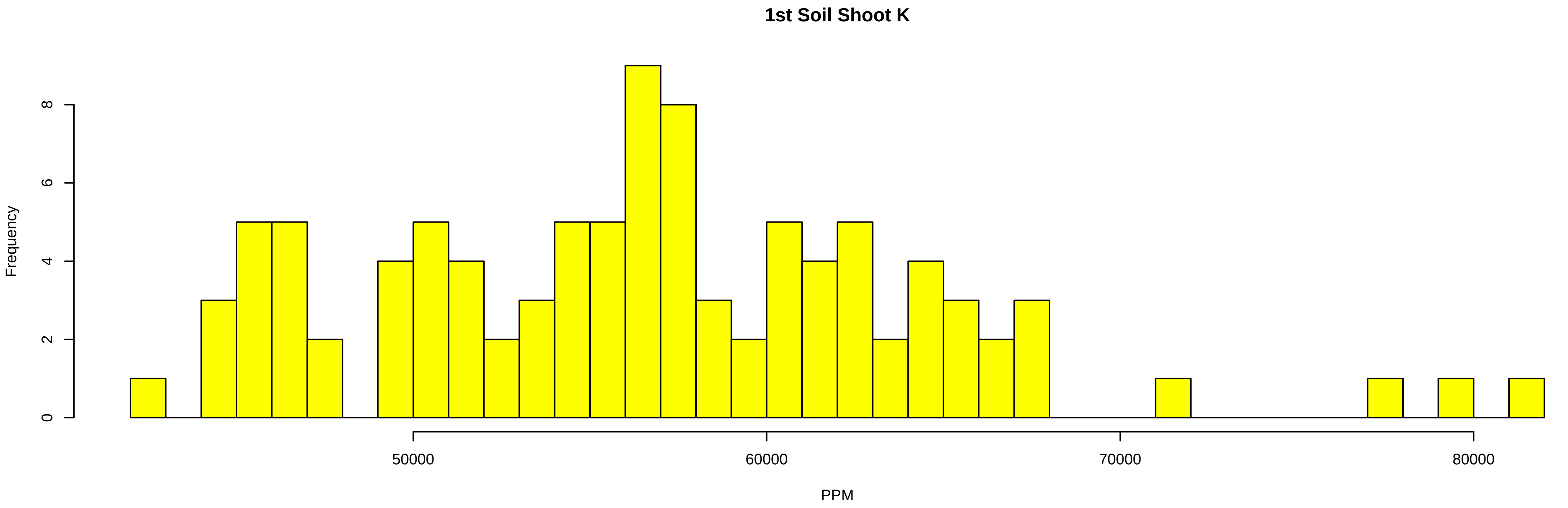

1st Soil Shoot K
Frequency
0
2
4
6
8
50000
60000
70000
80000
PPM

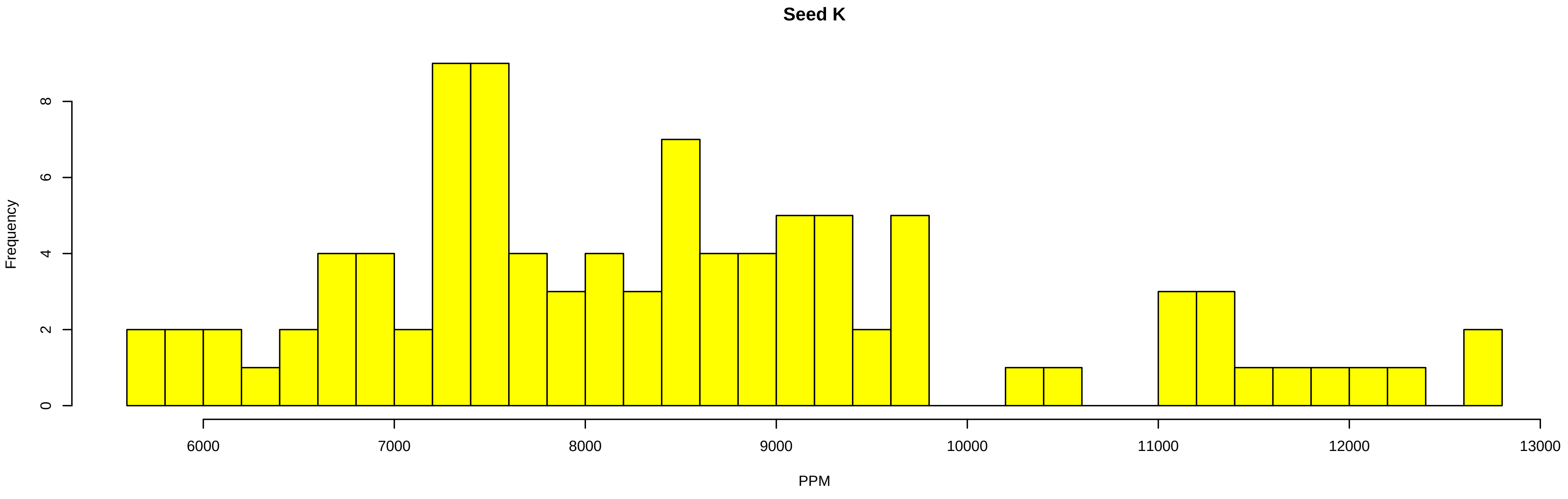

Seed K
Frequency
0
2
4
6
8
6000
7000
8000
9000
10000
11000
12000
13000
PPM

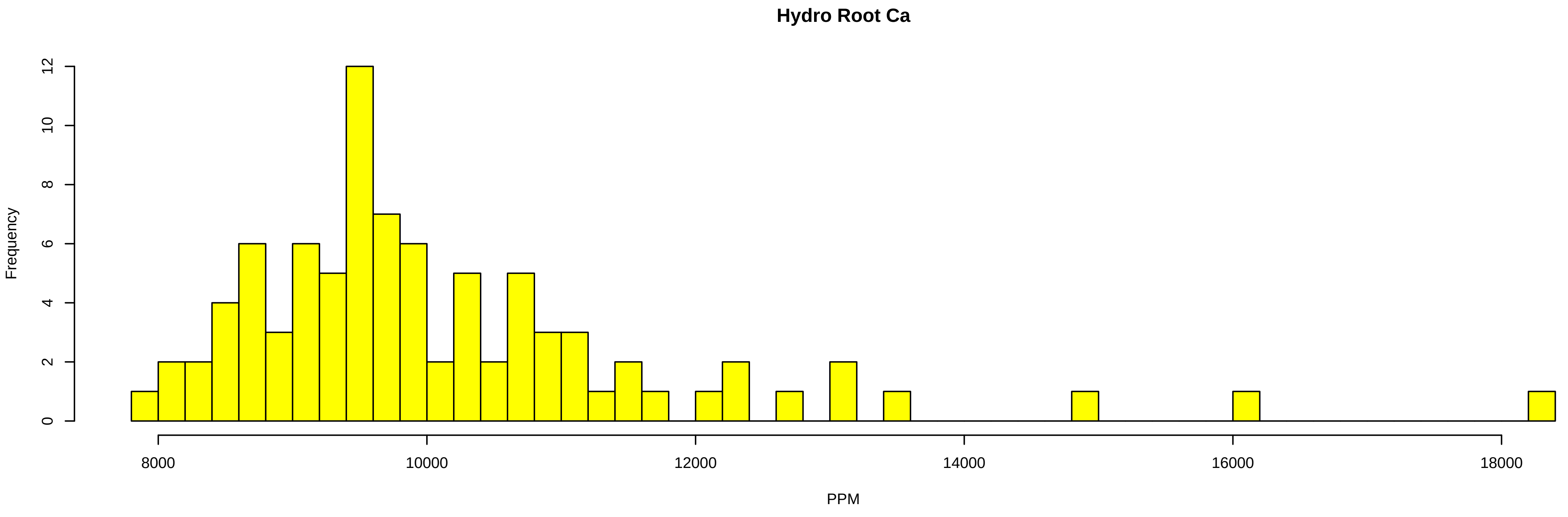
Hydro Root Ca
Frequency
0 2 4 6 8 10 12
8000 10000 12000 14000 16000 18000
PPM

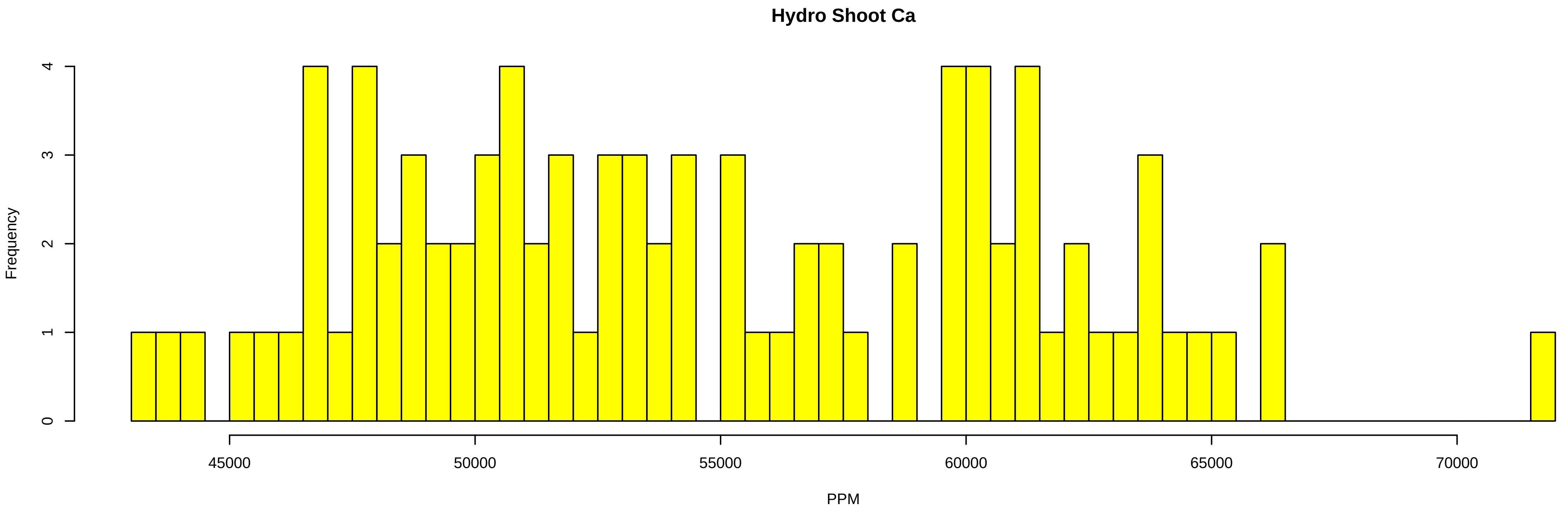
Hydro Shoot Ca
Frequency
0 1 2 3 4
45000 50000 55000 60000 65000 70000
PPM

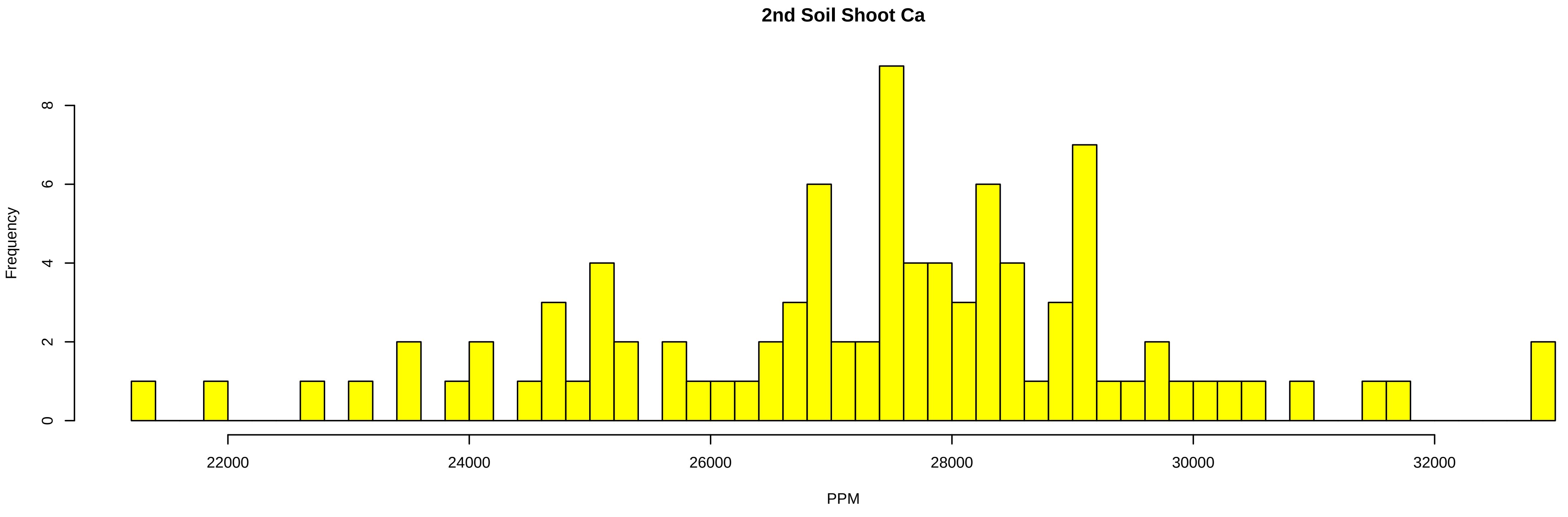
2nd Soil Shoot Ca
Frequency
0 2 4 6 8
22000 24000 26000 28000 30000 32000
PPM

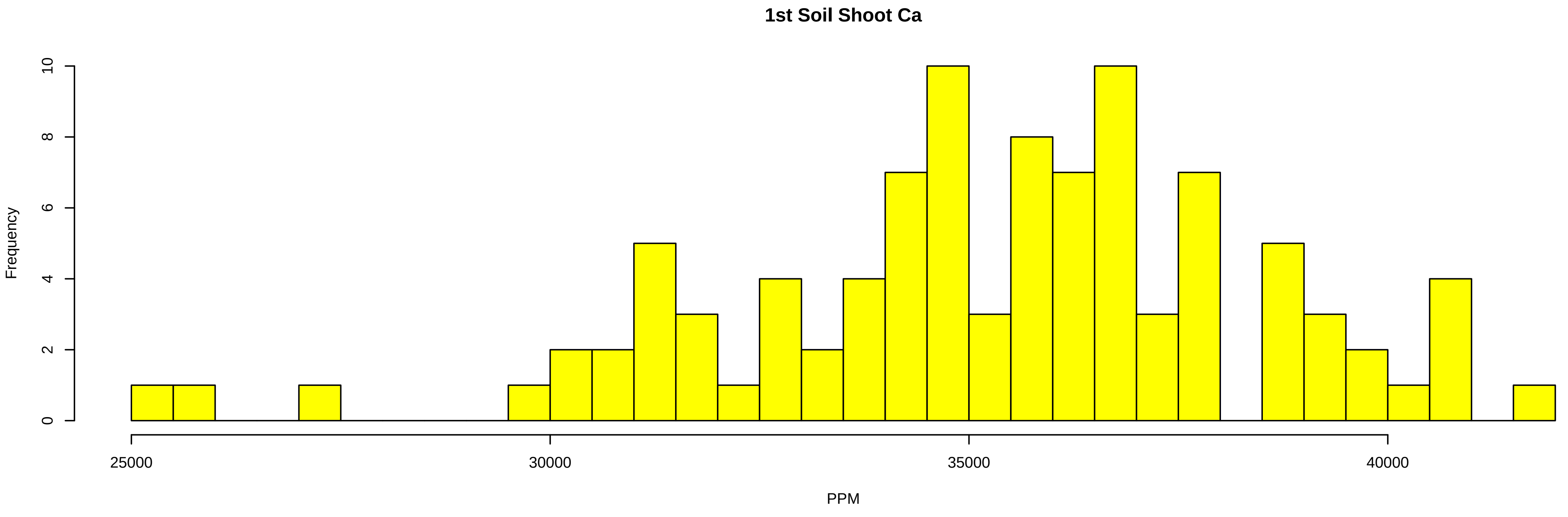
1st Soil Shoot Ca
Frequency
0 2 4 6 8 10
25000 30000 35000 40000
PPM

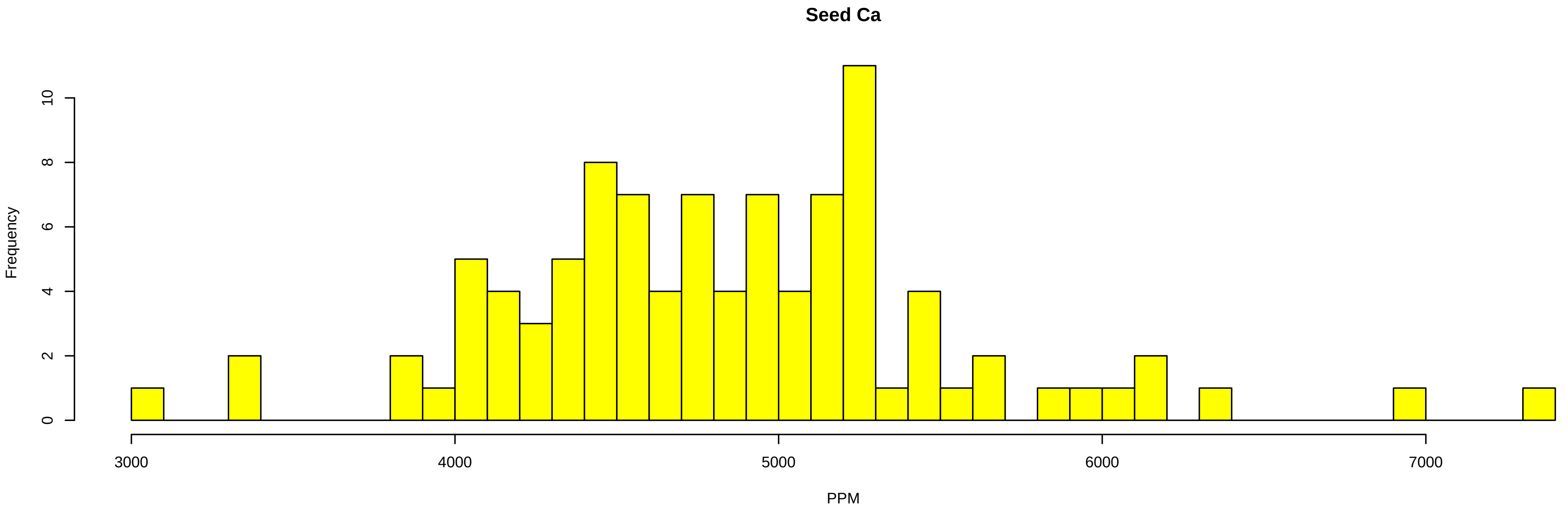
Seed Ca
Frequency
0 2 4 6 8 10
3000 4000 5000 6000 7000
PPM

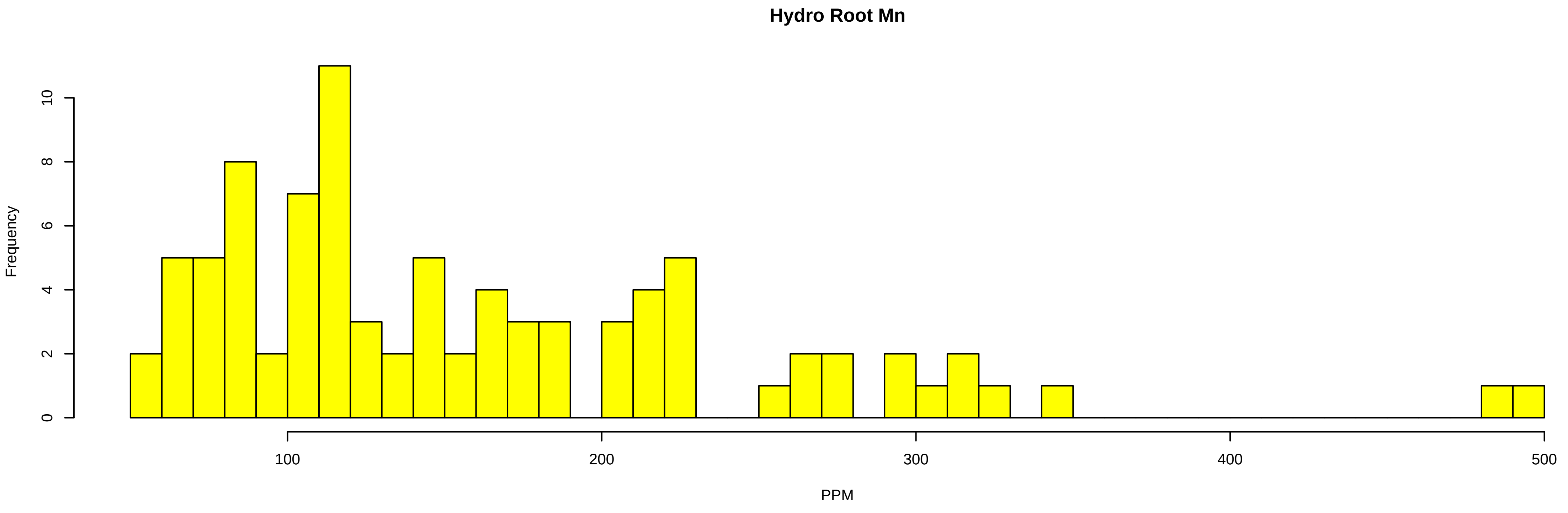
Hydro Root Mn
Frequency
0
2
4
6
8
10
100
200
300
400
500
PPM

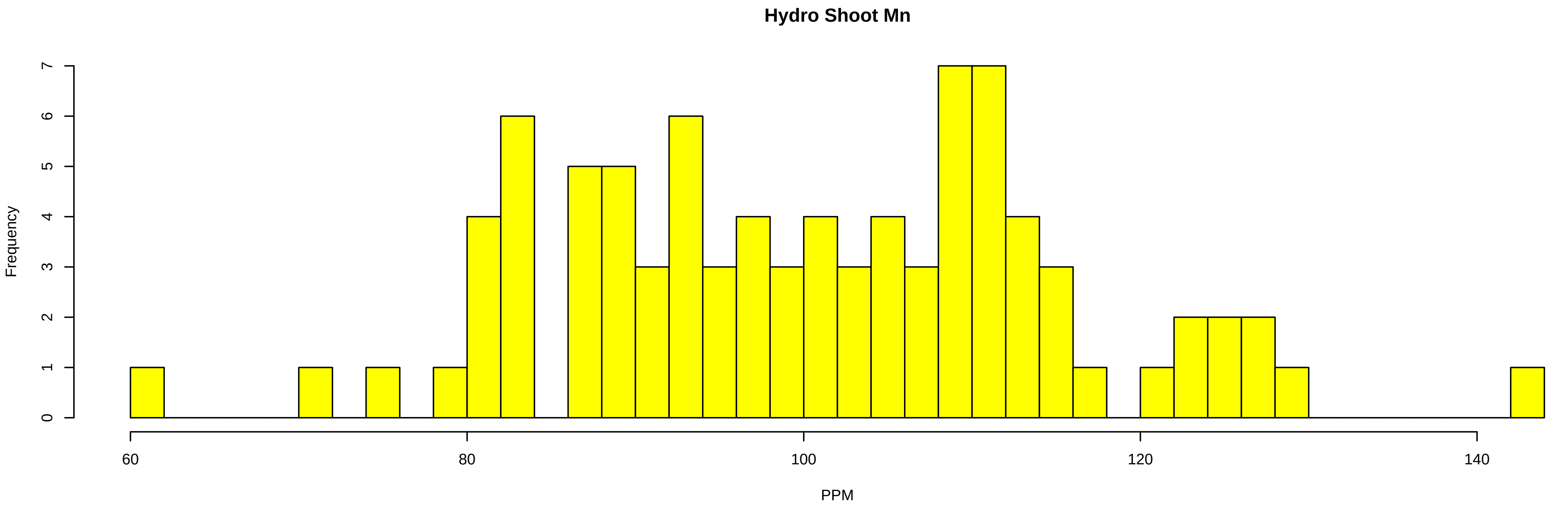
Hydro Shoot Mn
Frequency
0
1
2
3
4
5
6
7
60
80
100
120
140
PPM

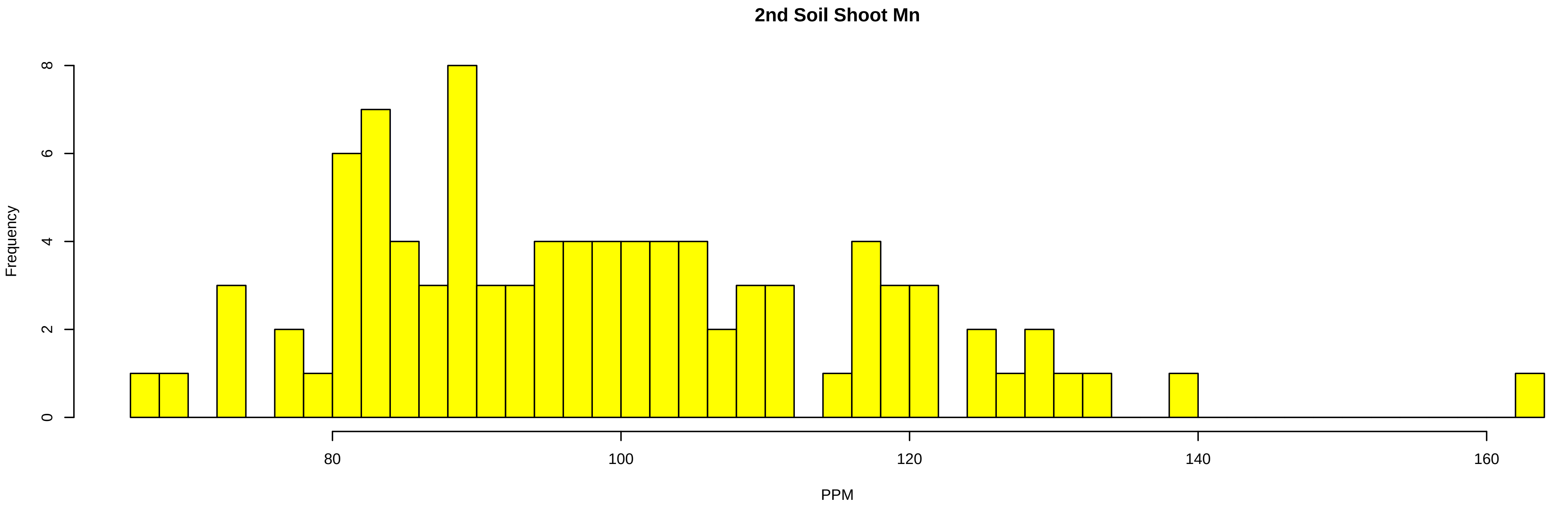
2nd Soil Shoot Mn
Frequency
0
2
4
6
8
80
100
120
140
160
PPM

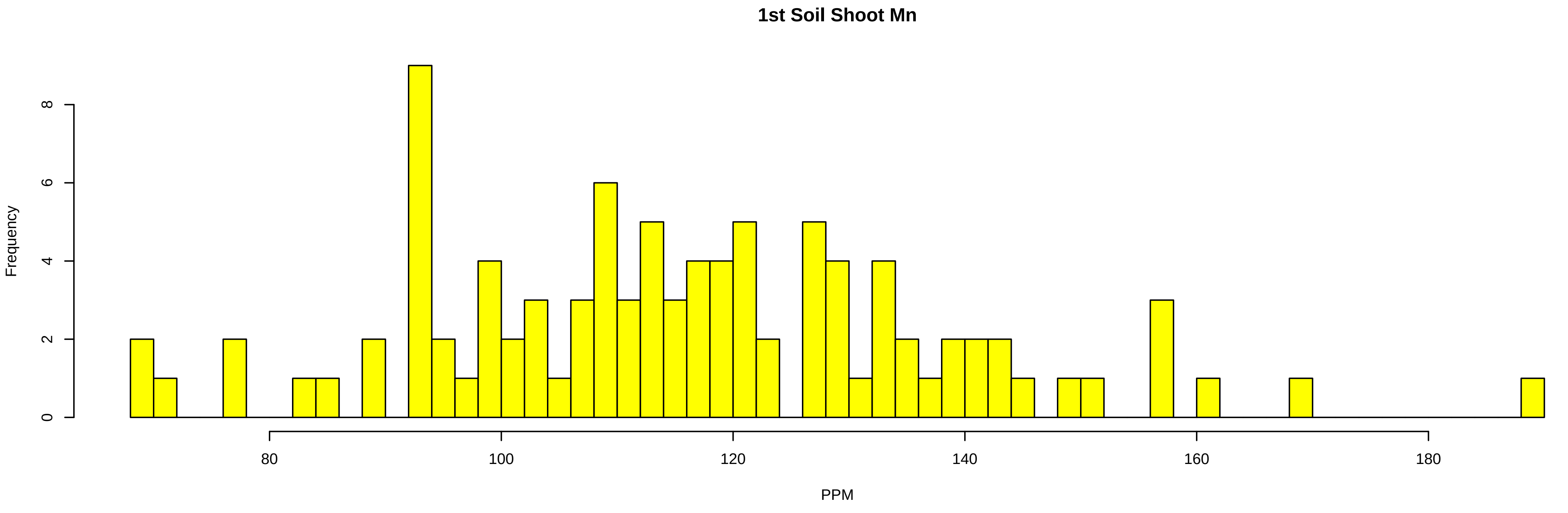
1st Soil Shoot Mn
Frequency
0
2
4
6
8
80
100
120
140
160
180
PPM

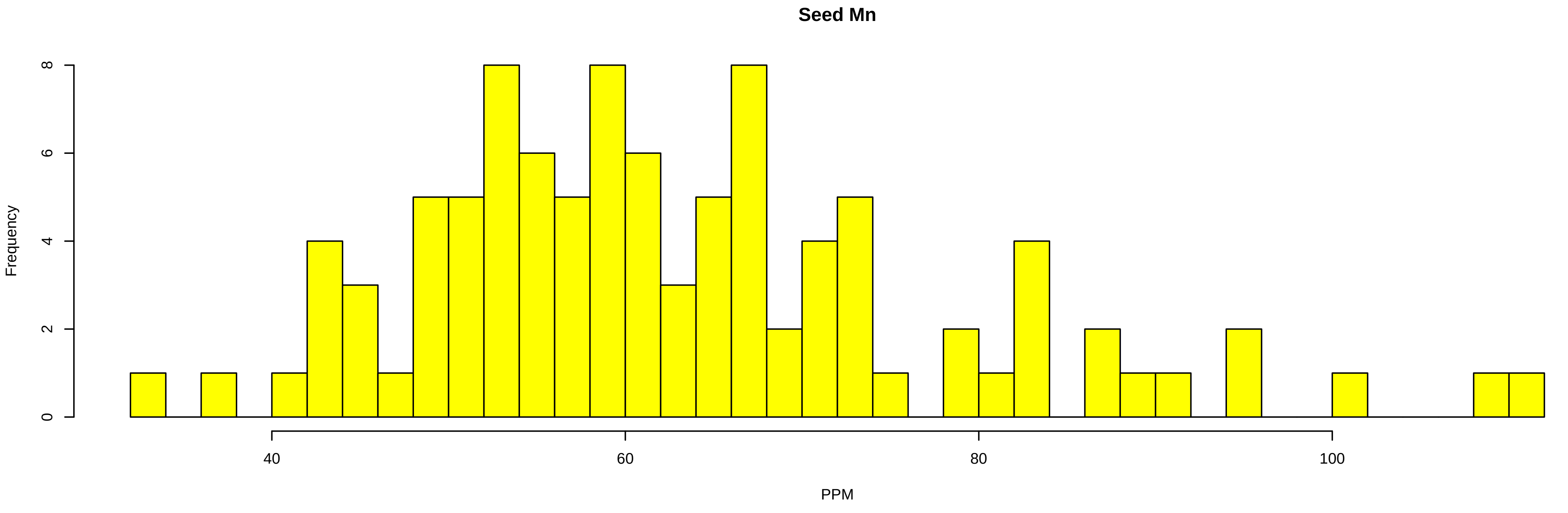
Seed Mn
Frequency
0
2
4
6
8
40
60
80
100
PPM

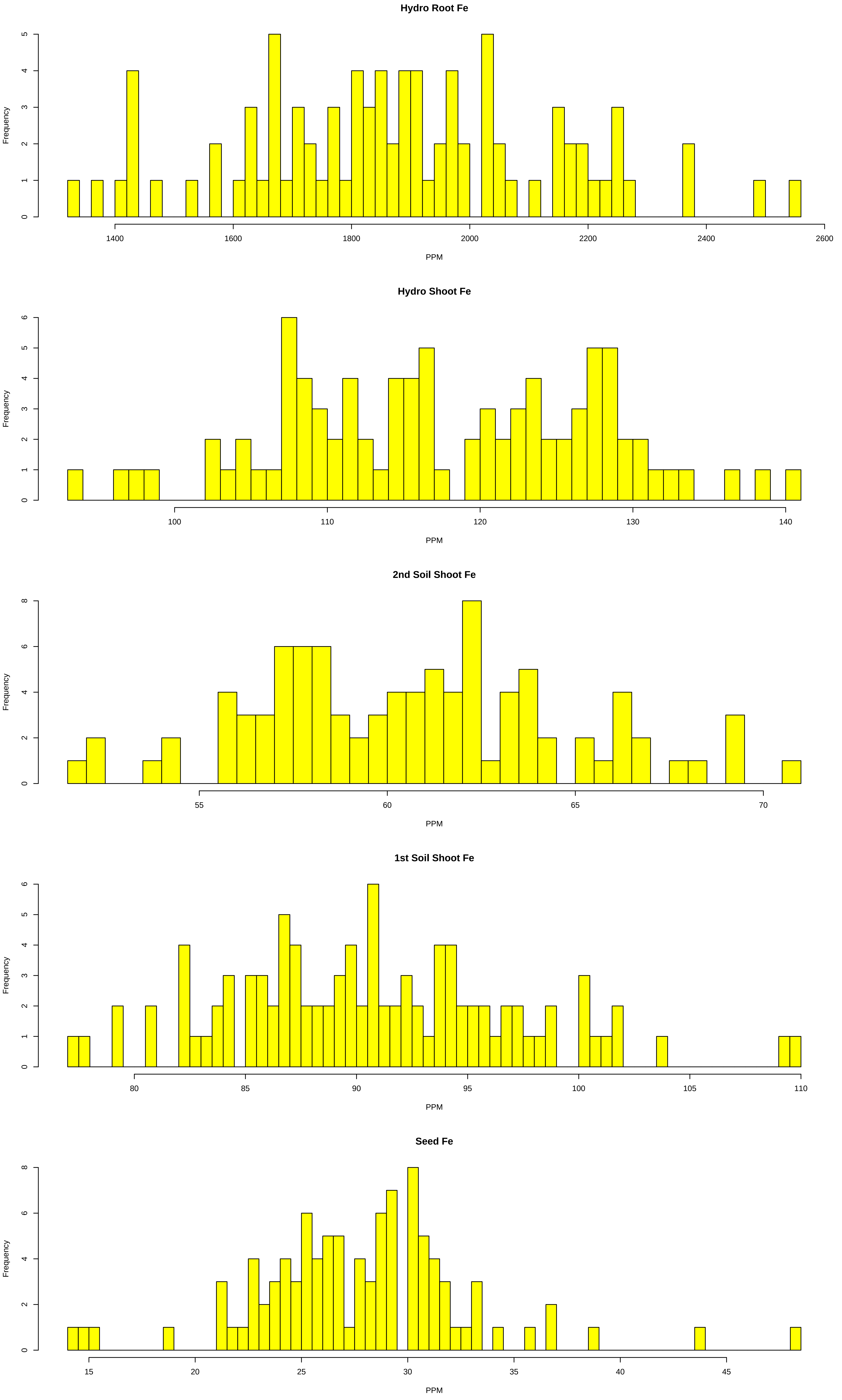
Hydro Root Fe
Frequency
PPM
Hydro Shoot Fe
Frequency
PPM
2nd Soil Shoot Fe
Frequency
PPM
1st Soil Shoot Fe
Frequency
PPM
Seed Fe
Frequency
PPM

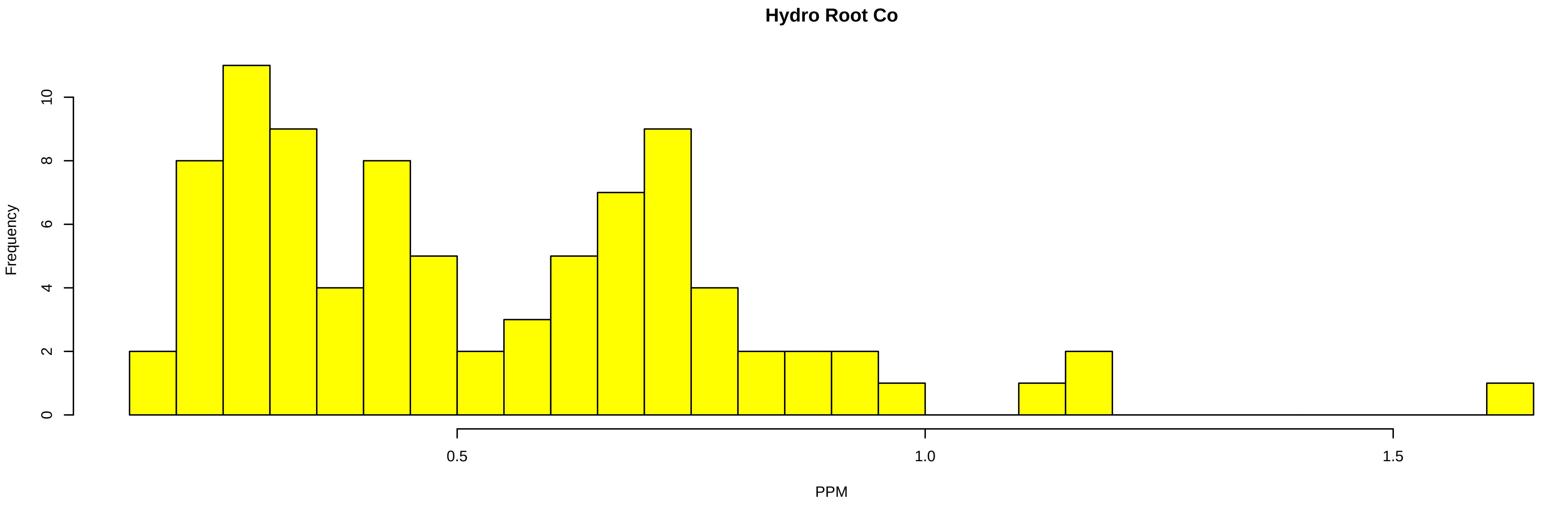
Hydro Root Co
Frequency
0
2
4
6
8
10
0.5
1.0
1.5
PPM

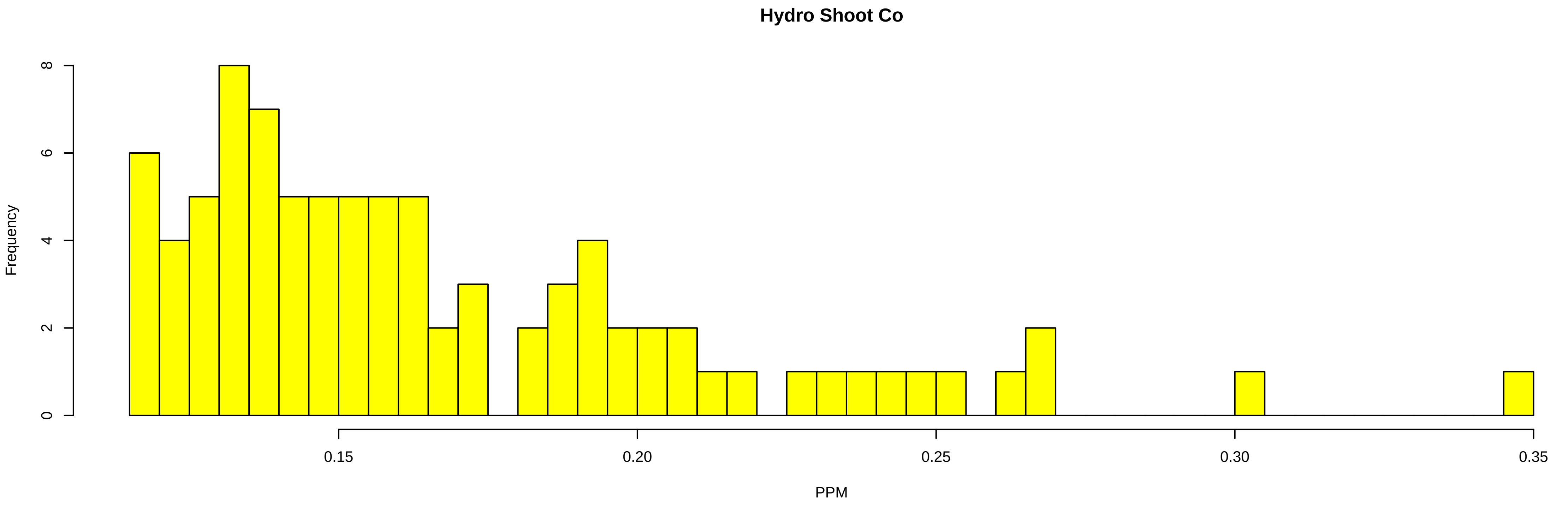
Hydro Shoot Co
Frequency
0
2
4
6
8
0.15
0.20
0.25
0.30
0.35
PPM

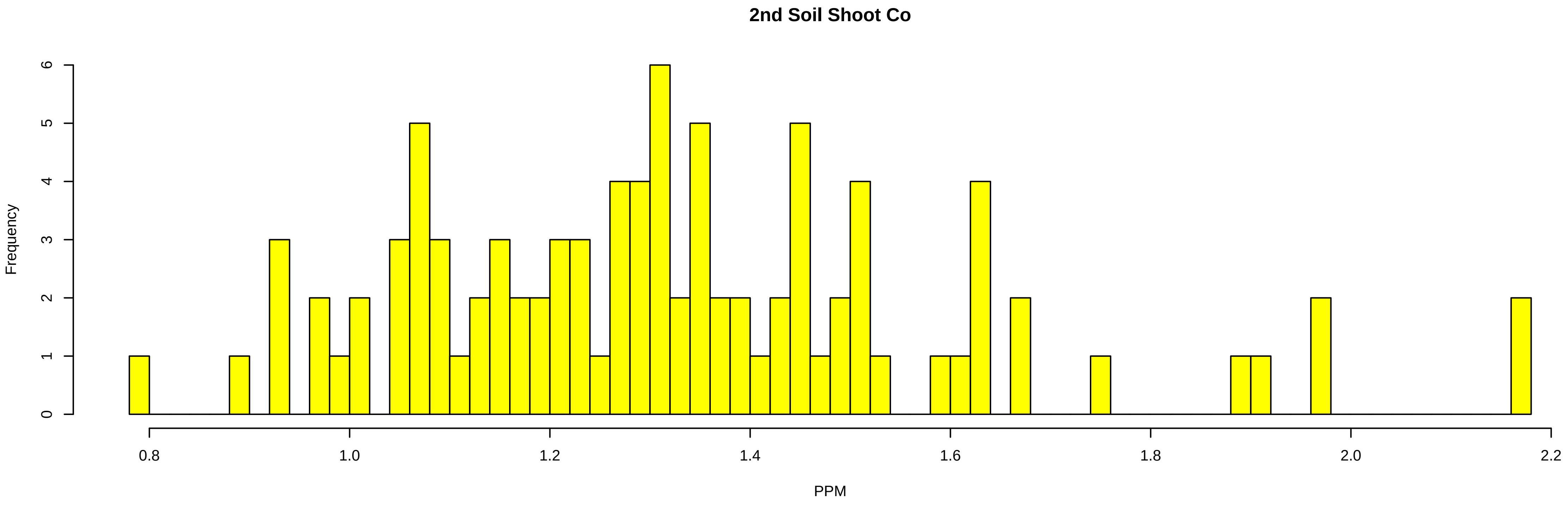
2nd Soil Shoot Co
Frequency
0
1
2
3
4
5
6
0.8
1.0
1.2
1.4
1.6
1.8
2.0
2.2
PPM

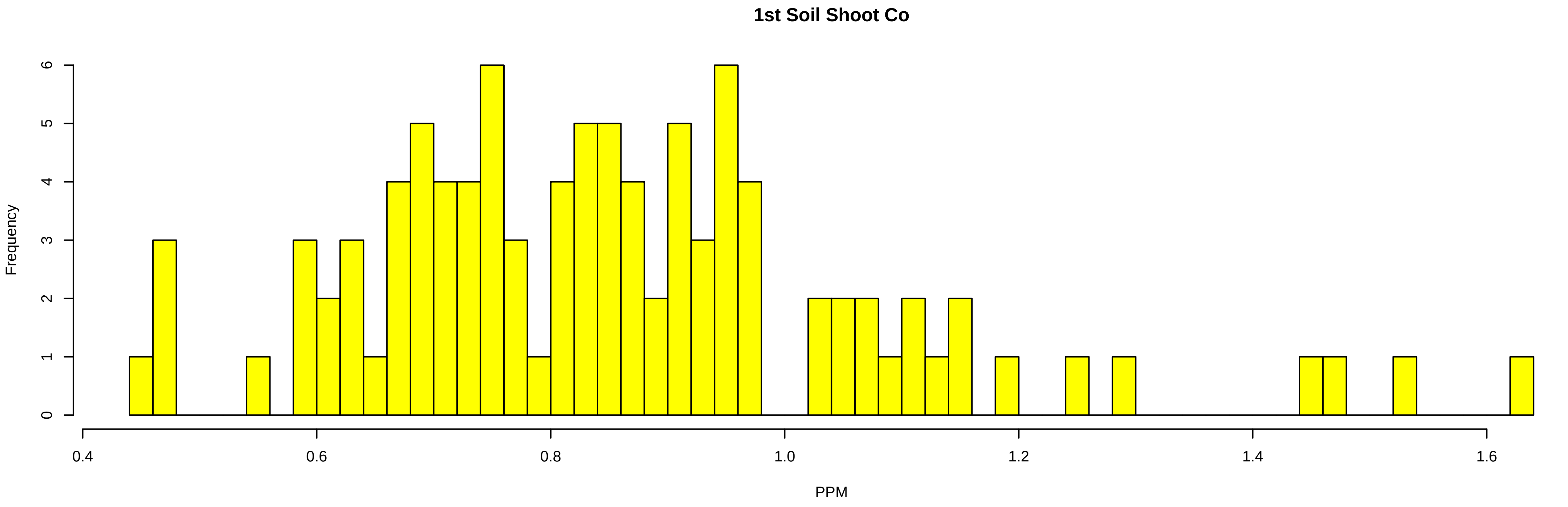
1st Soil Shoot Co
Frequency
0
1
2
3
4
5
6
0.4
0.6
0.8
1.0
1.2
1.4
1.6
PPM

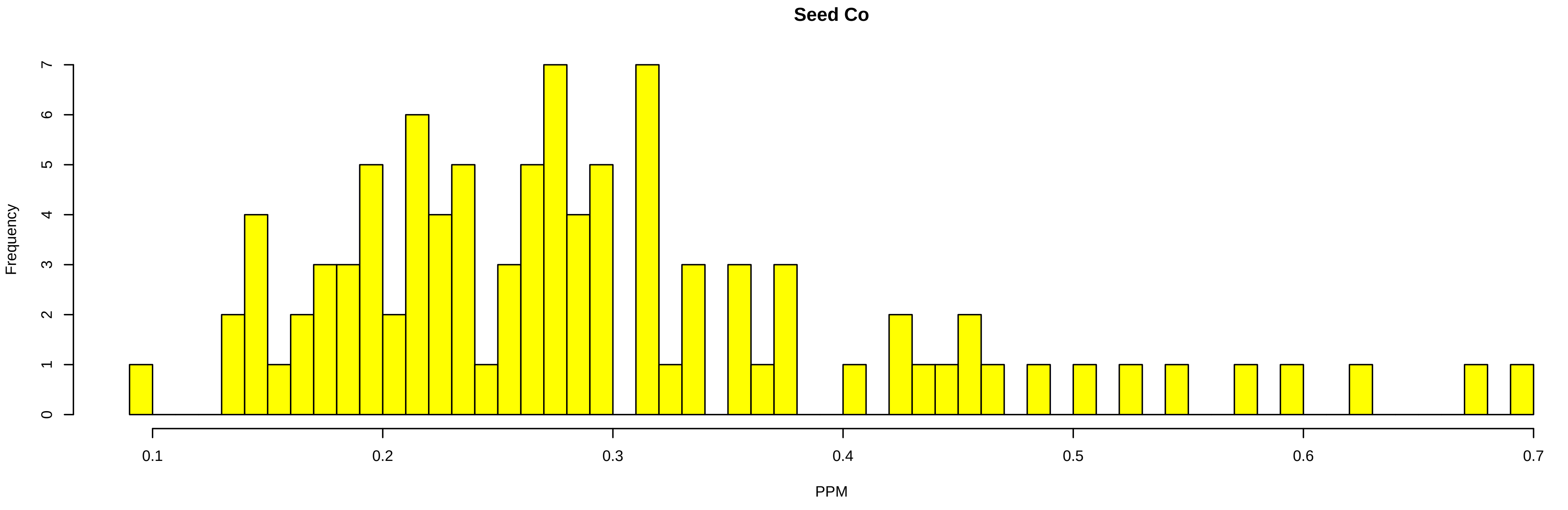
Seed Co
Frequency
0
1
2
3
4
5
6
7
0.1
0.2
0.3
0.4
0.5
0.6
0.7
PPM

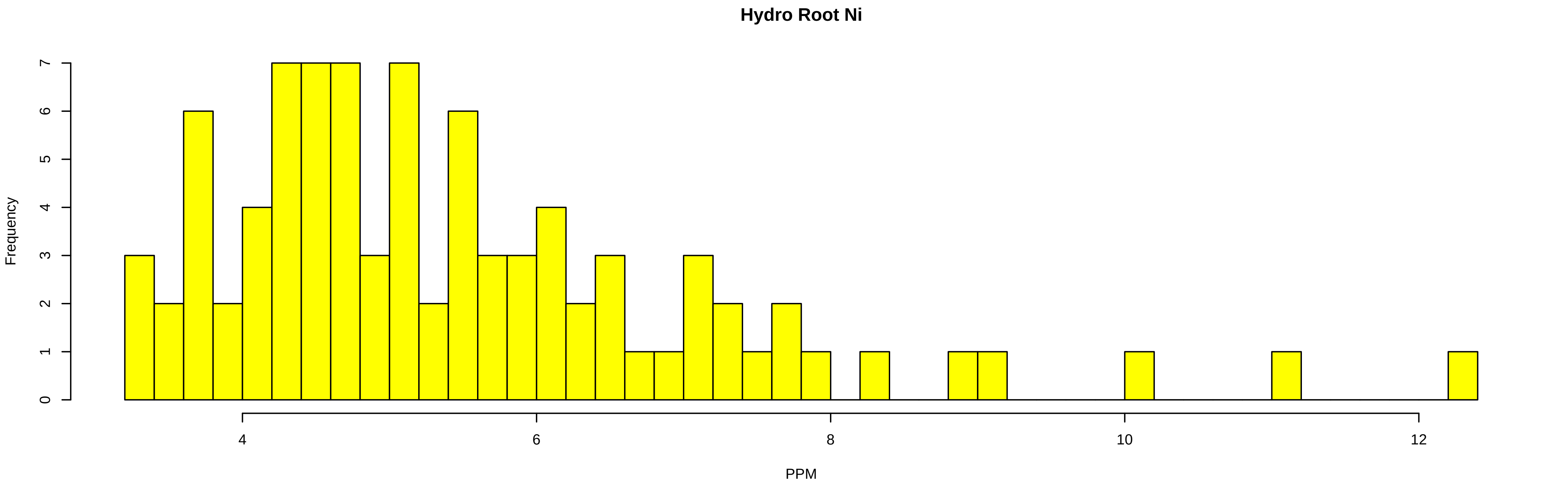
Hydro Root Ni
Frequency
0
1
2
3
4
5
6
7
4
6
8
10
12
PPM

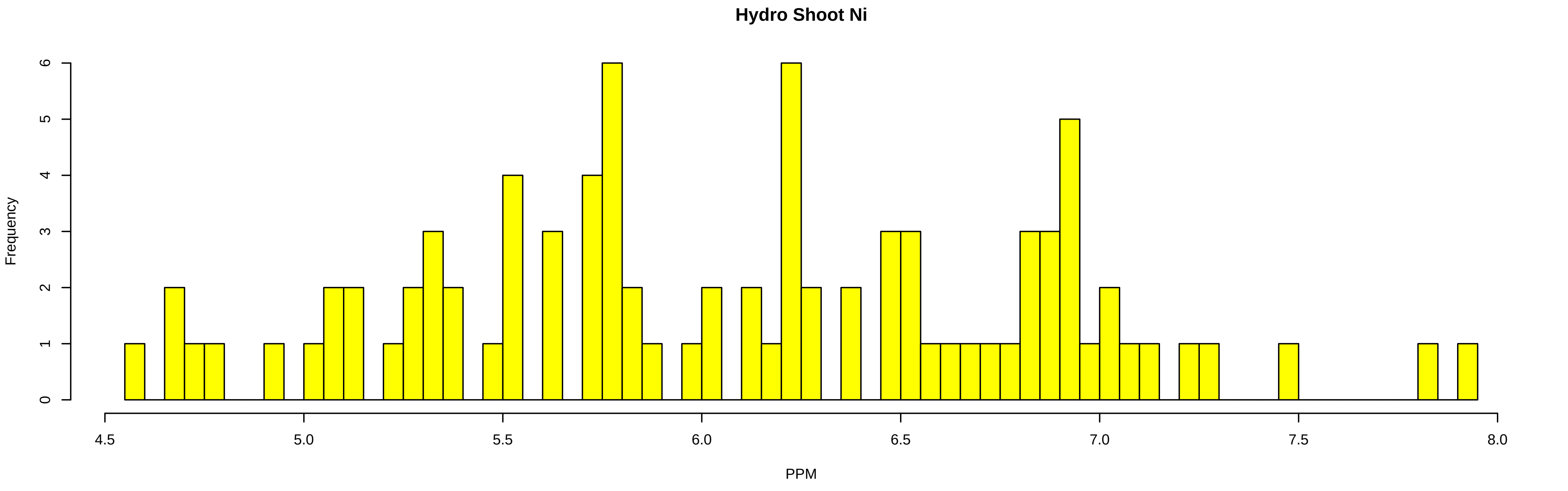
Hydro Shoot Ni
Frequency
0
1
2
3
4
5
6
4.5
5.0
5.5
6.0
6.5
7.0
7.5
8.0
PPM

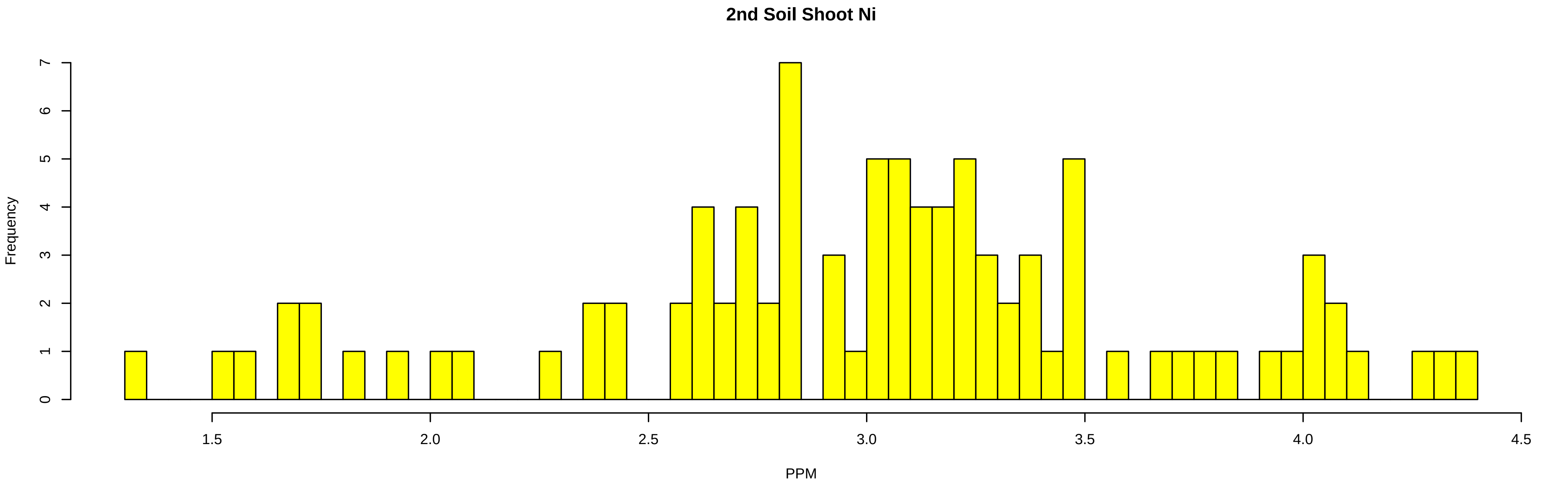
2nd Soil Shoot Ni
Frequency
0
1
2
3
4
5
6
7
1.5
2.0
2.5
3.0
3.5
4.0
4.5
PPM

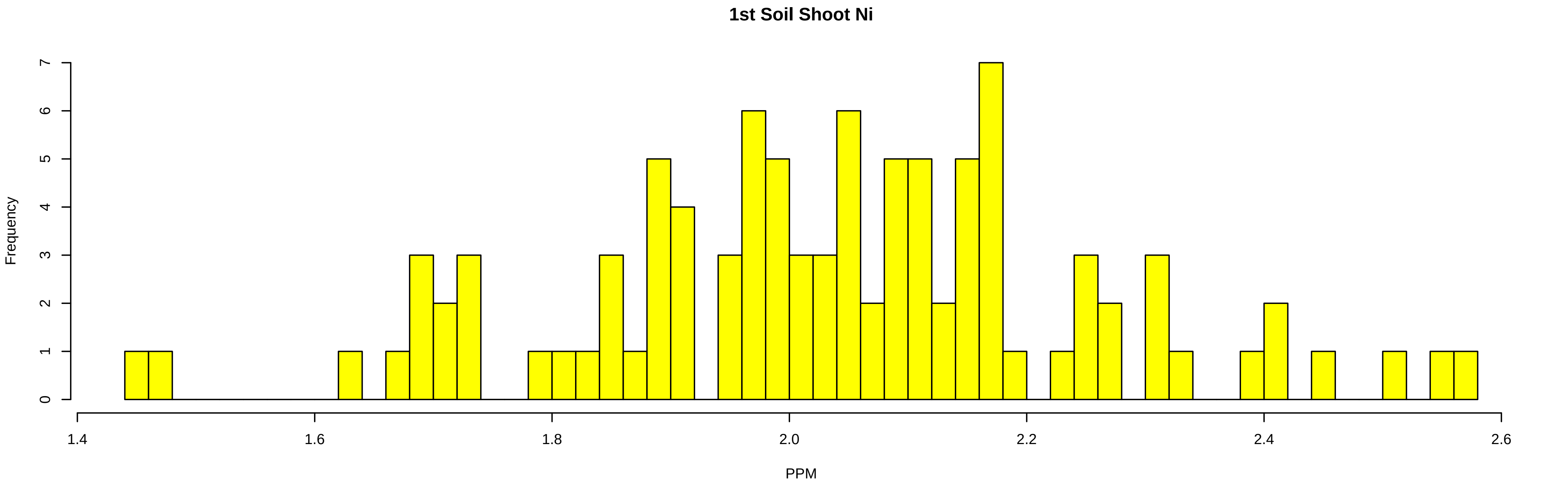
1st Soil Shoot Ni
Frequency
0
1
2
3
4
5
6
7
1.4
1.6
1.8
2.0
2.2
2.4
2.6
PPM

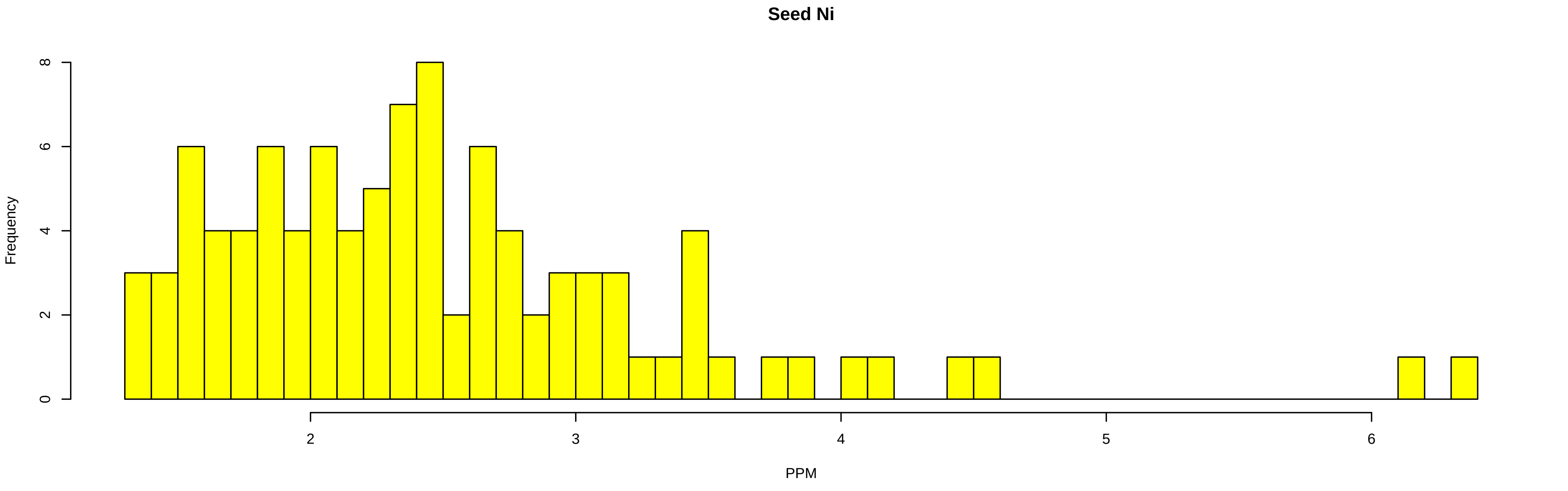
Seed Ni
Frequency
0
2
4
6
8
2
3
4
5
6
PPM

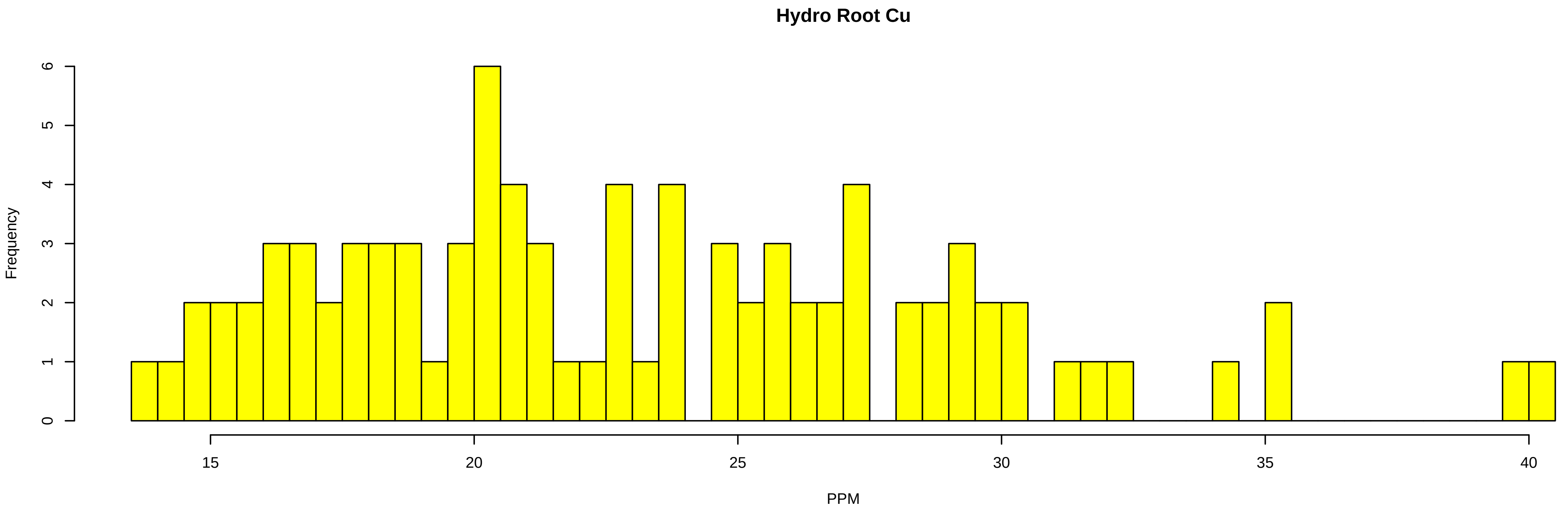
Hydro Root Cu
Frequency
PPM
0
1
2
3
4
5
6
15
20
25
30
35
40

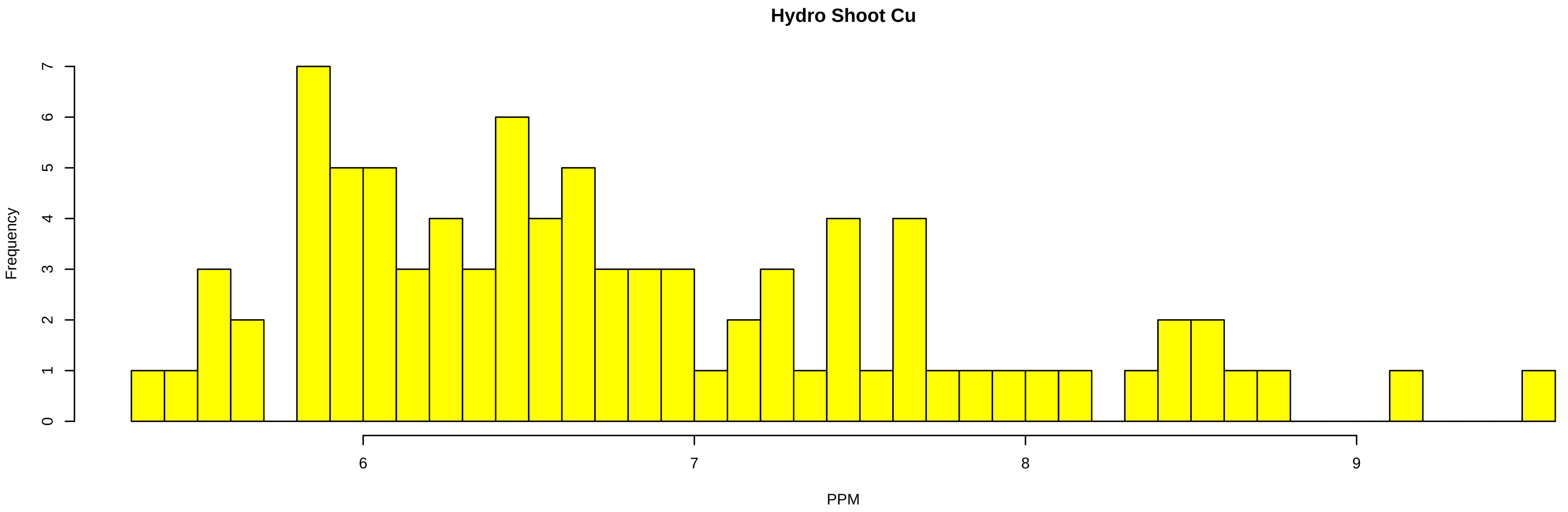
Hydro Shoot Cu
Frequency
PPM
0
1
2
3
4
5
6
7
6
7
8
9

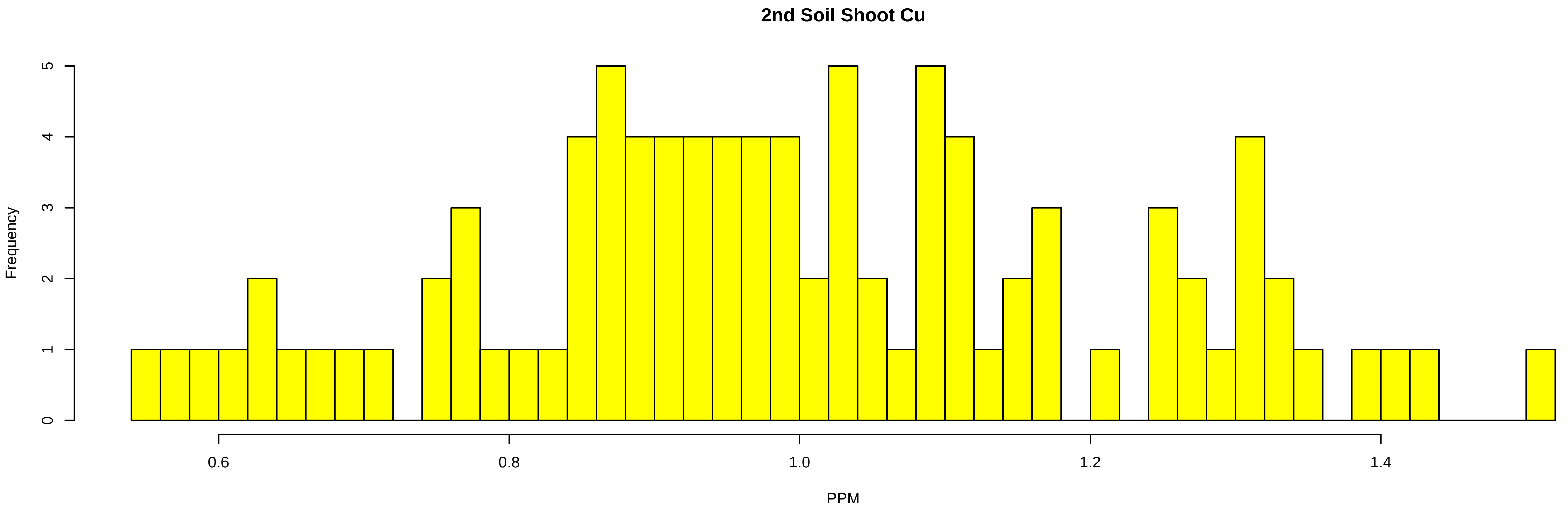
2nd Soil Shoot Cu
Frequency
PPM
0
1
2
3
4
5
0.6
0.8
1.0
1.2
1.4

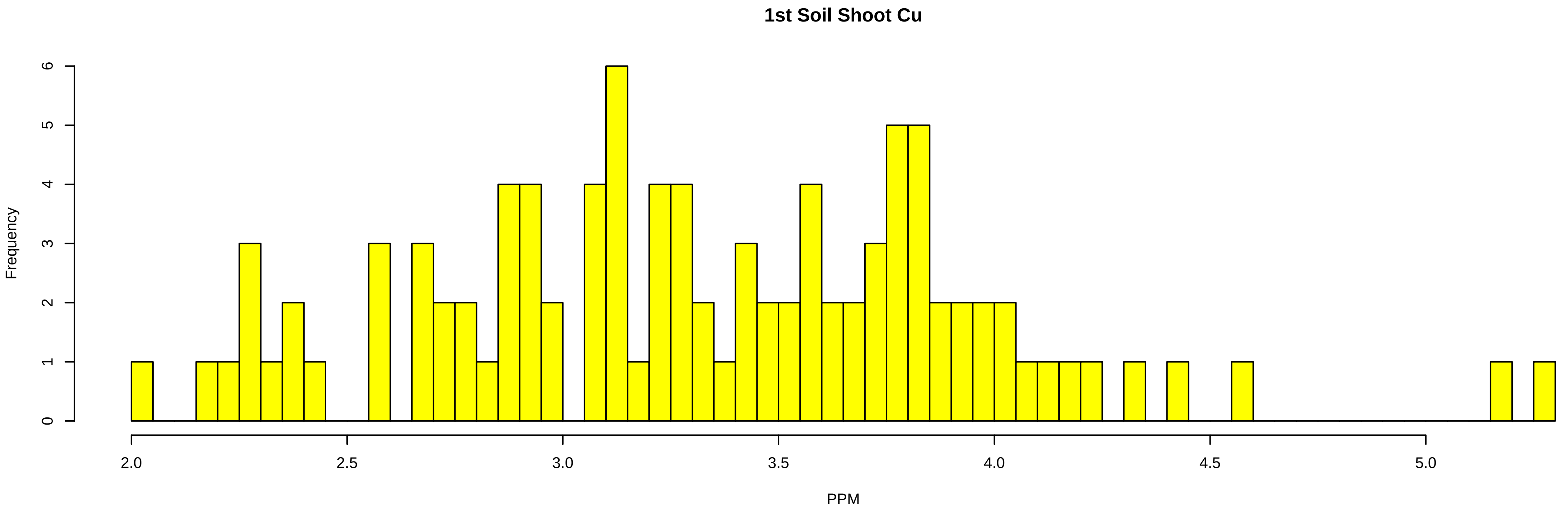
1st Soil Shoot Cu
Frequency
PPM
0
1
2
3
4
5
6
2.0
2.5
3.0
3.5
4.0
4.5
5.0

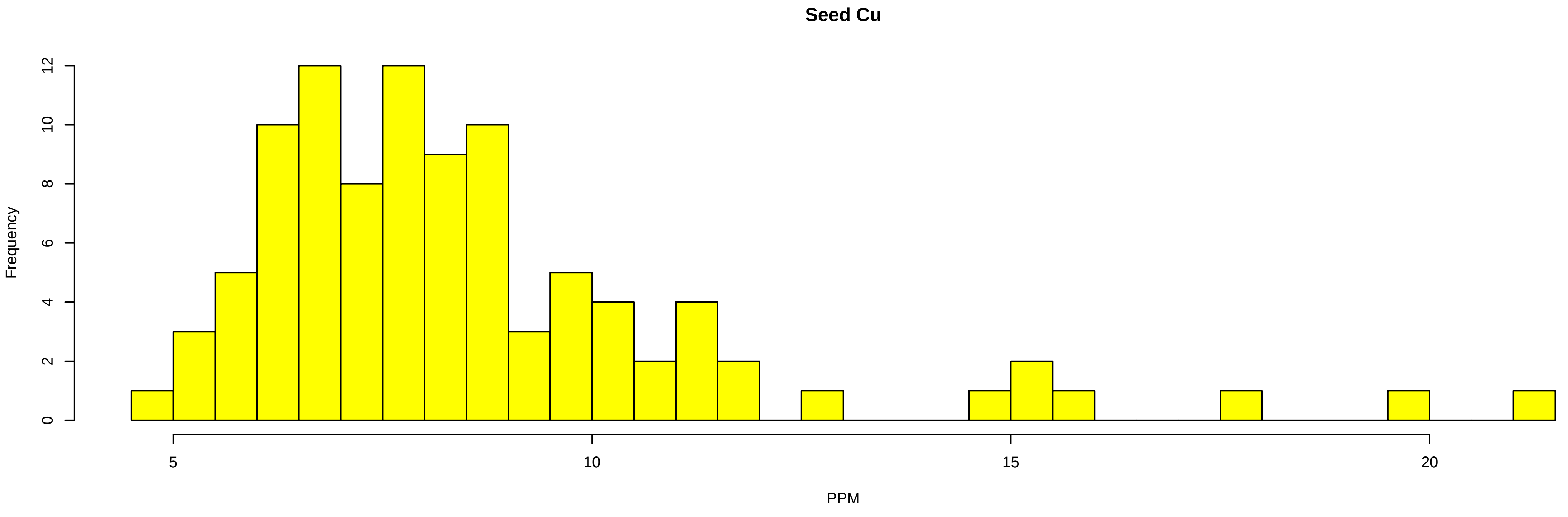
Seed Cu
Frequency
PPM
0
2
4
6
8
10
12
5
10
15
20

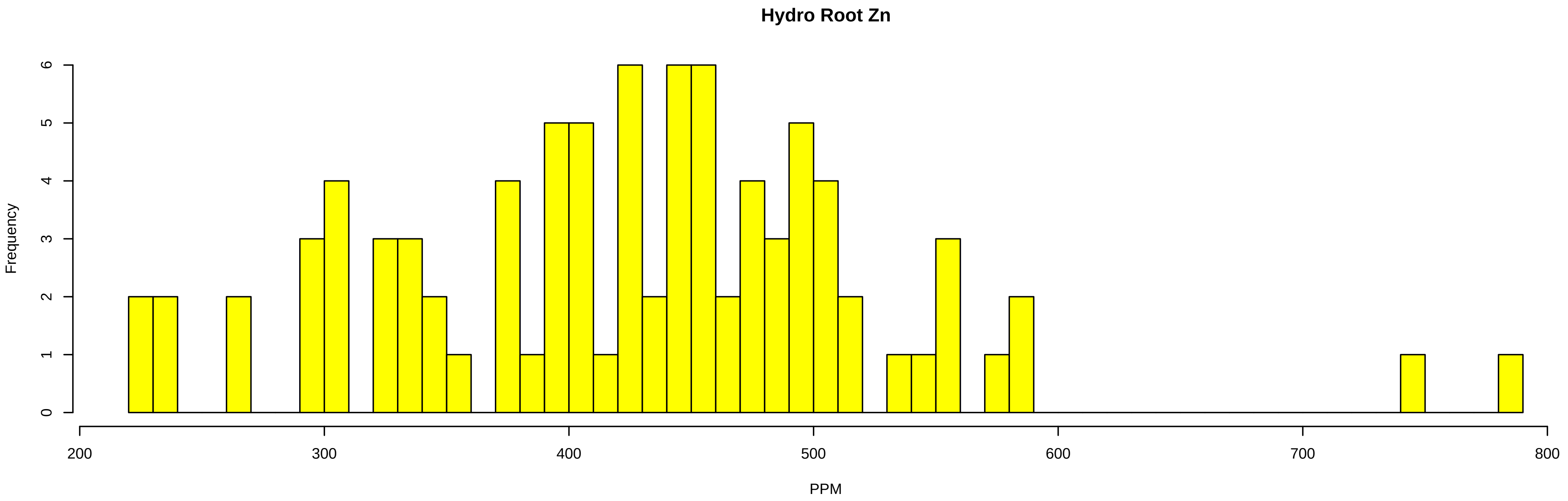
Hydro Root Zn
Frequency
PPM
0
1
2
3
4
5
6
200
300
400
500
600
700
800

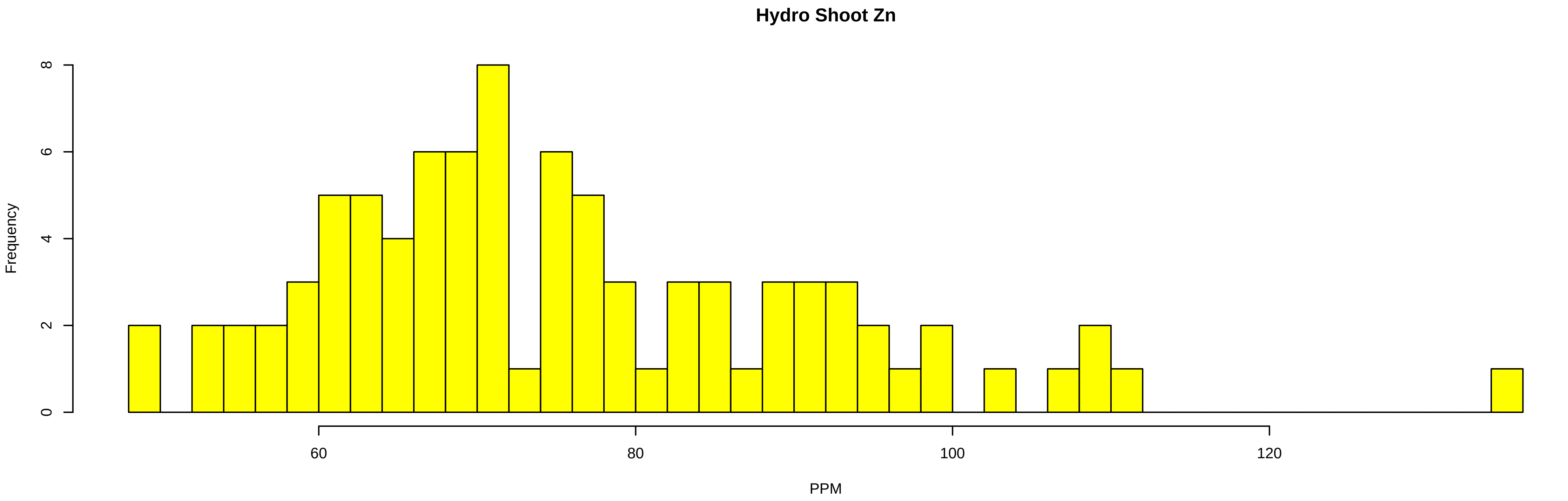
Hydro Shoot Zn
Frequency
PPM
0
2
4
6
8
60
80
100
120

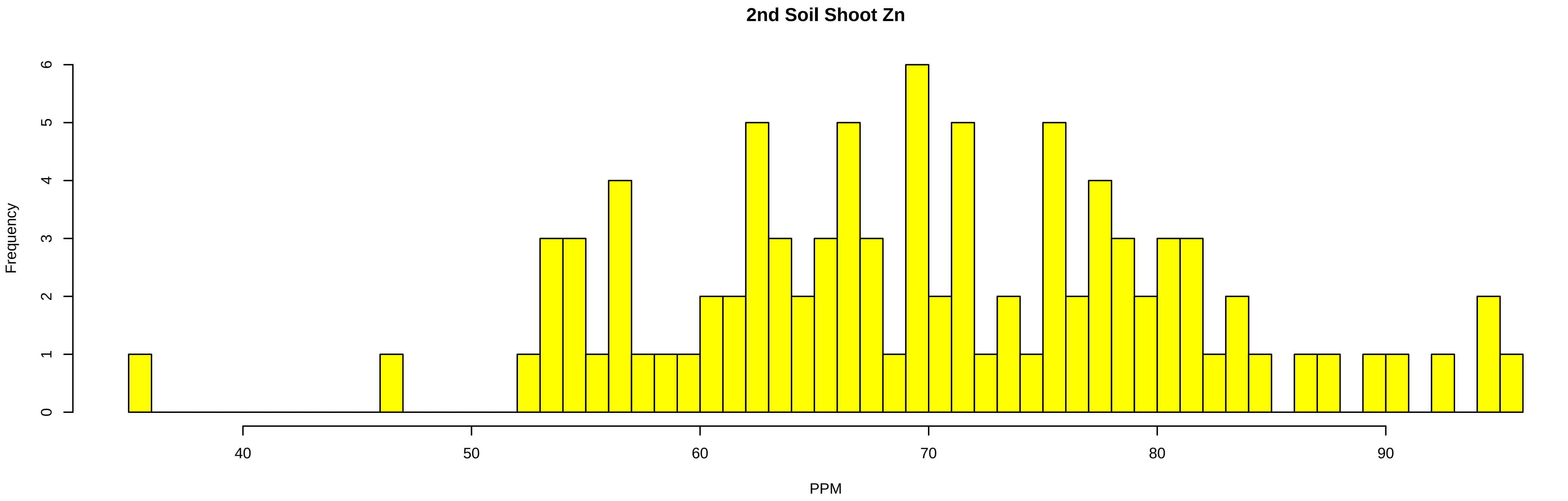
2nd Soil Shoot Zn
Frequency
PPM
0
1
2
3
4
5
6
40
50
60
70
80
90

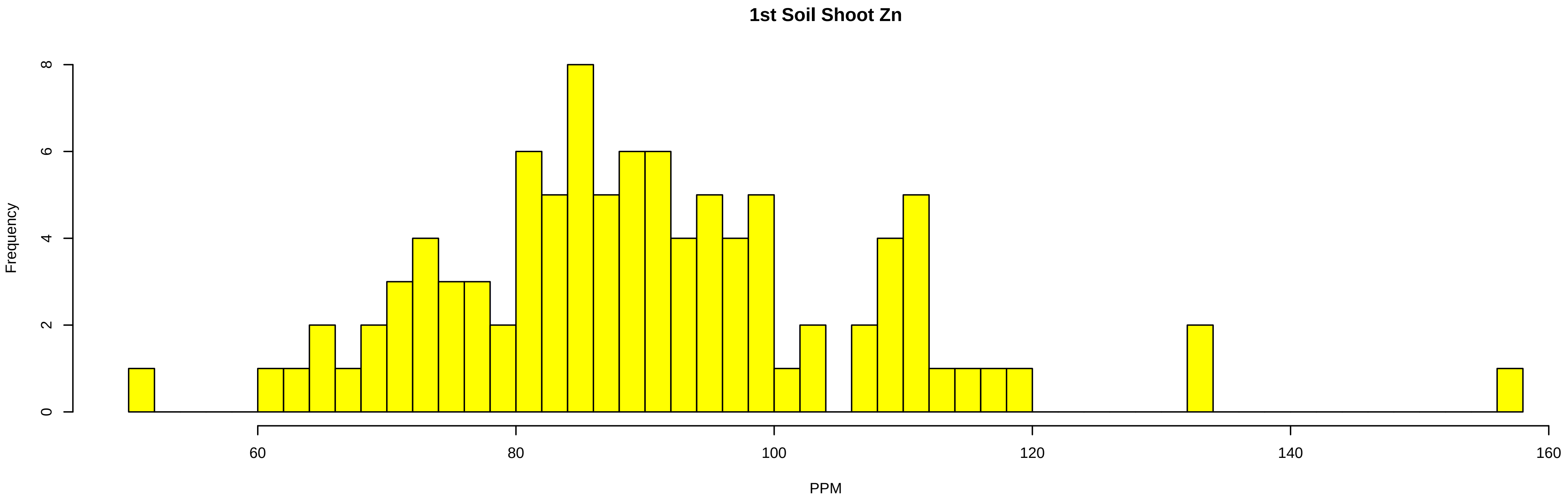
1st Soil Shoot Zn
Frequency
PPM
0
2
4
6
8
60
80
100
120
140
160

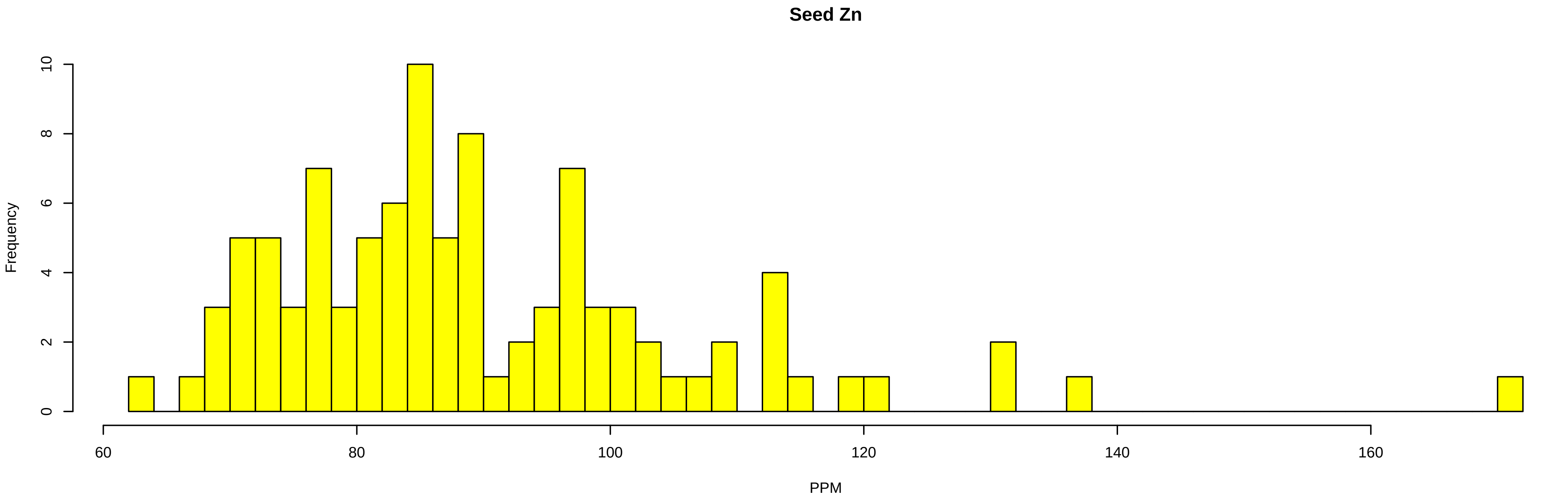
Seed Zn
Frequency
PPM
0
2
4
6
8
10
60
80
100
120
140
160

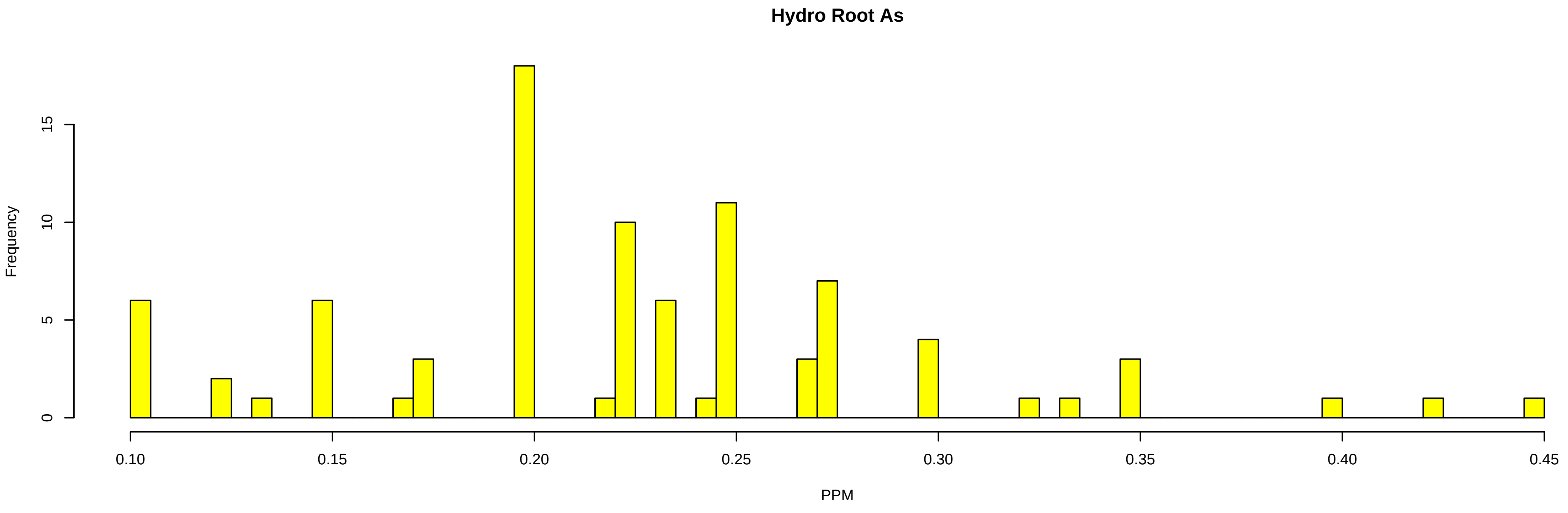
Hydro Root As
Frequency
0 5 10 15
0.10 0.15 0.20 0.25 0.30 0.35 0.40 0.45
PPM

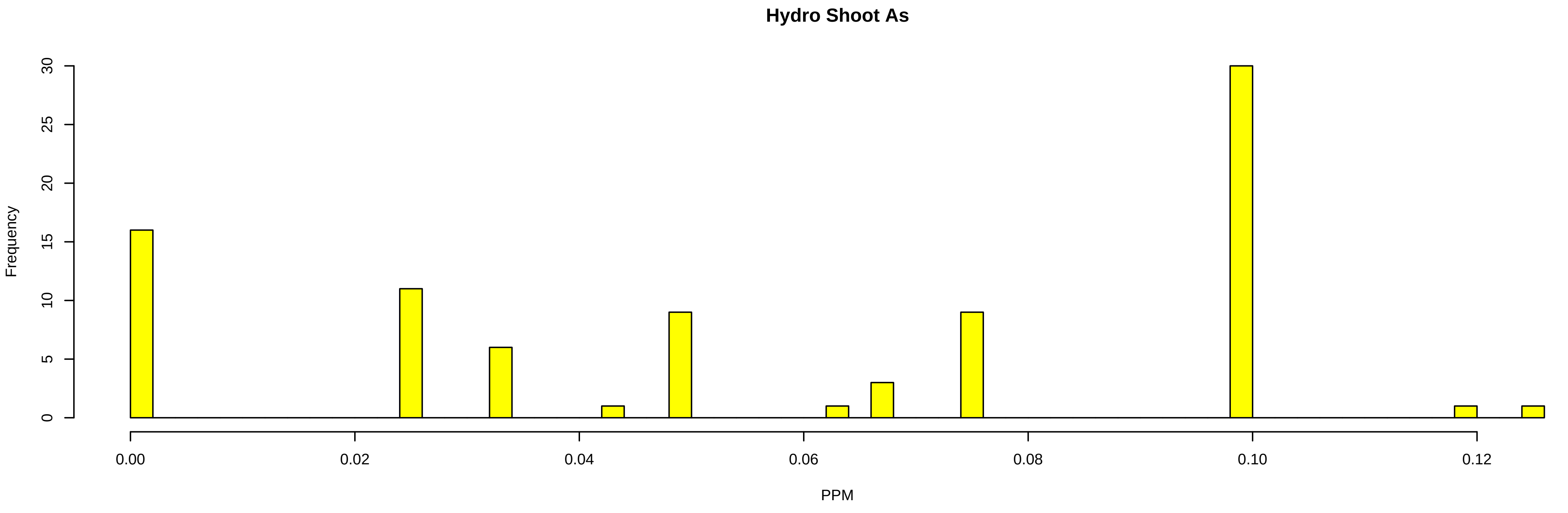
Hydro Shoot As
Frequency
0 5 10 15 20 25 30
0.00 0.02 0.04 0.06 0.08 0.10 0.12
PPM

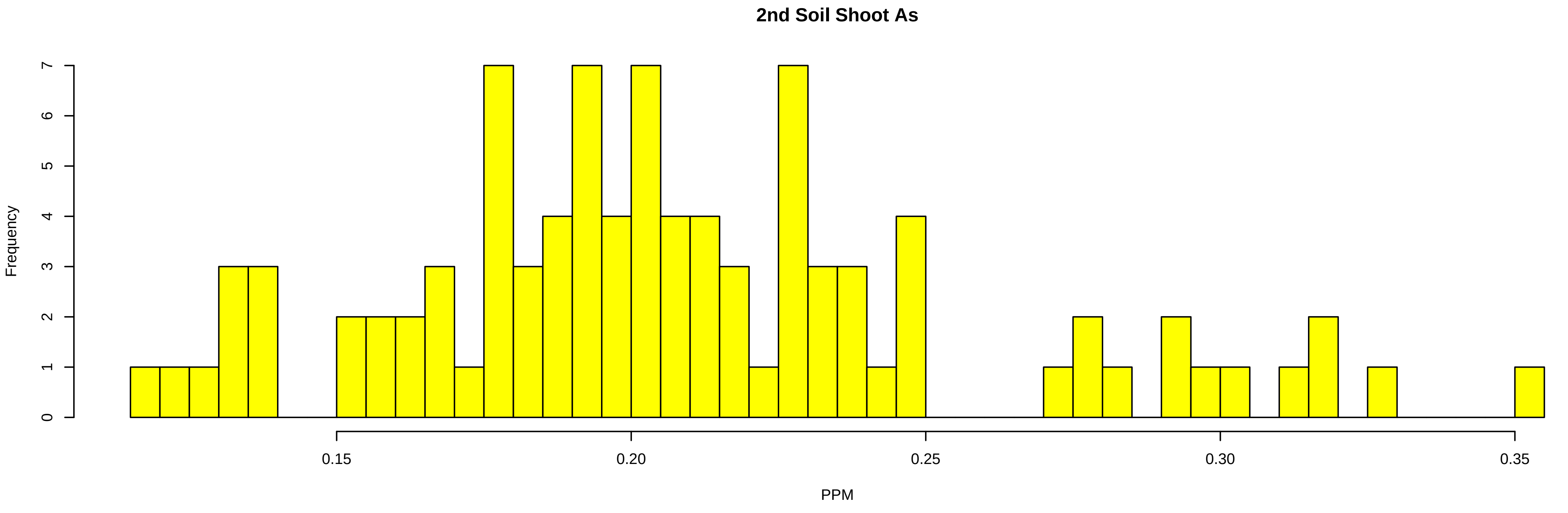
2nd Soil Shoot As
Frequency
0 1 2 3 4 5 6 7
0.15 0.20 0.25 0.30 0.35
PPM

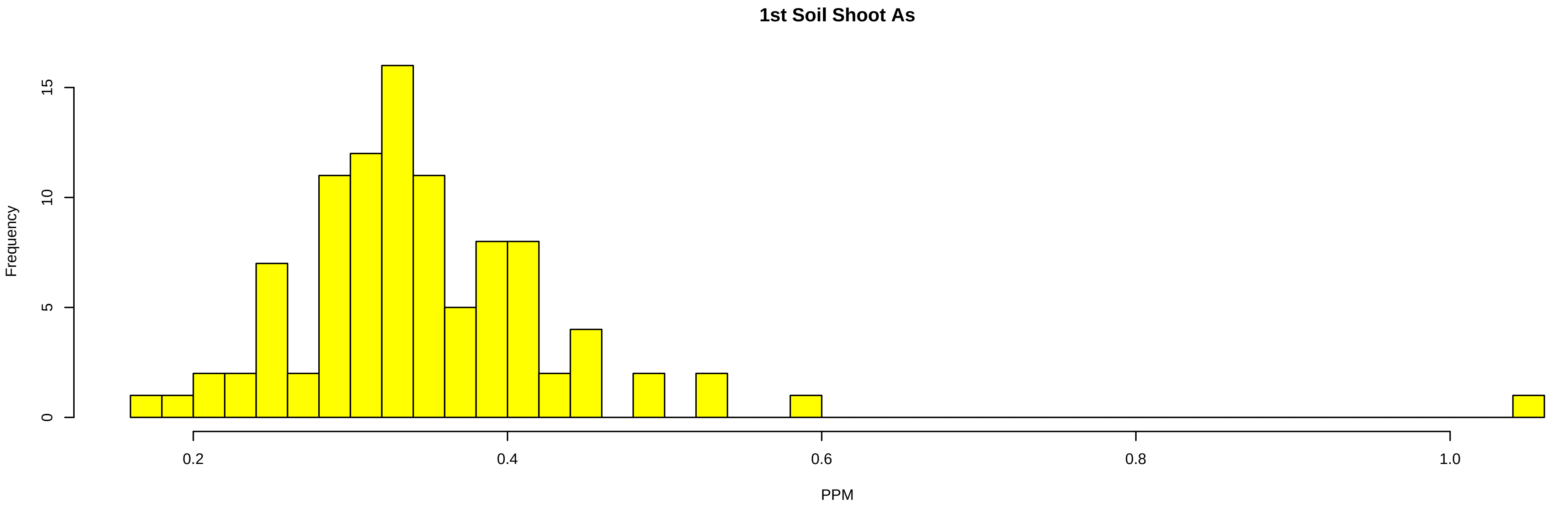
1st Soil Shoot As
Frequency
0 5 10 15
0.2 0.4 0.6 0.8 1.0
PPM

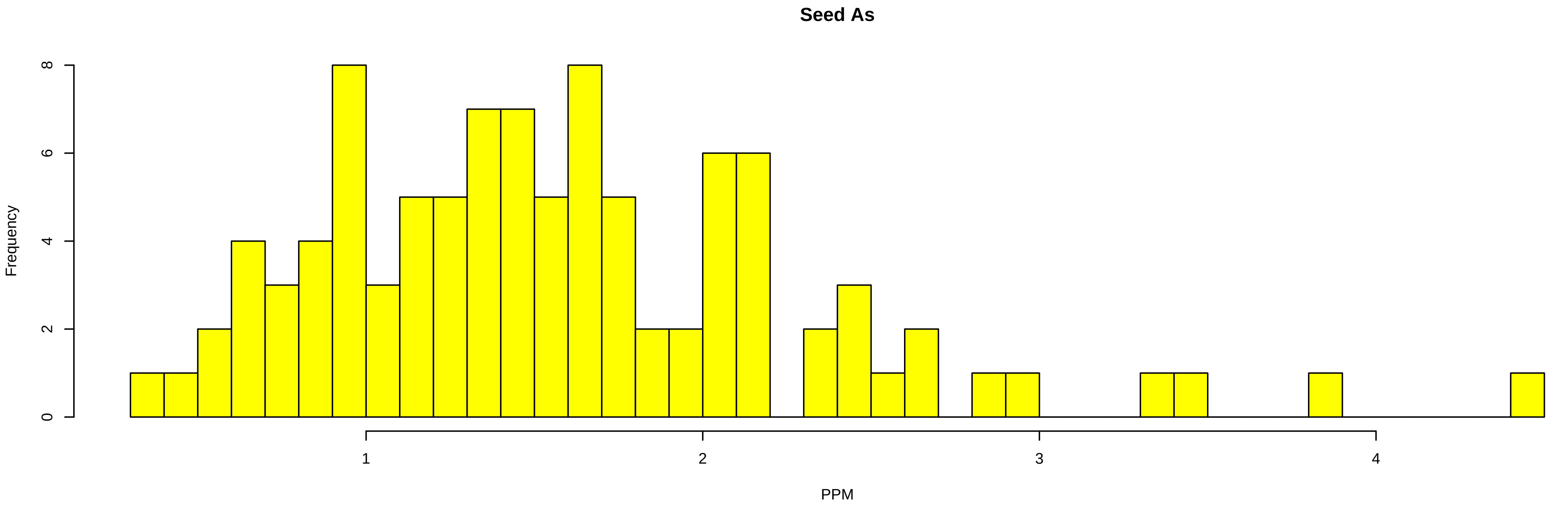
Seed As
Frequency
0 2 4 6 8
1 2 3 4
PPM

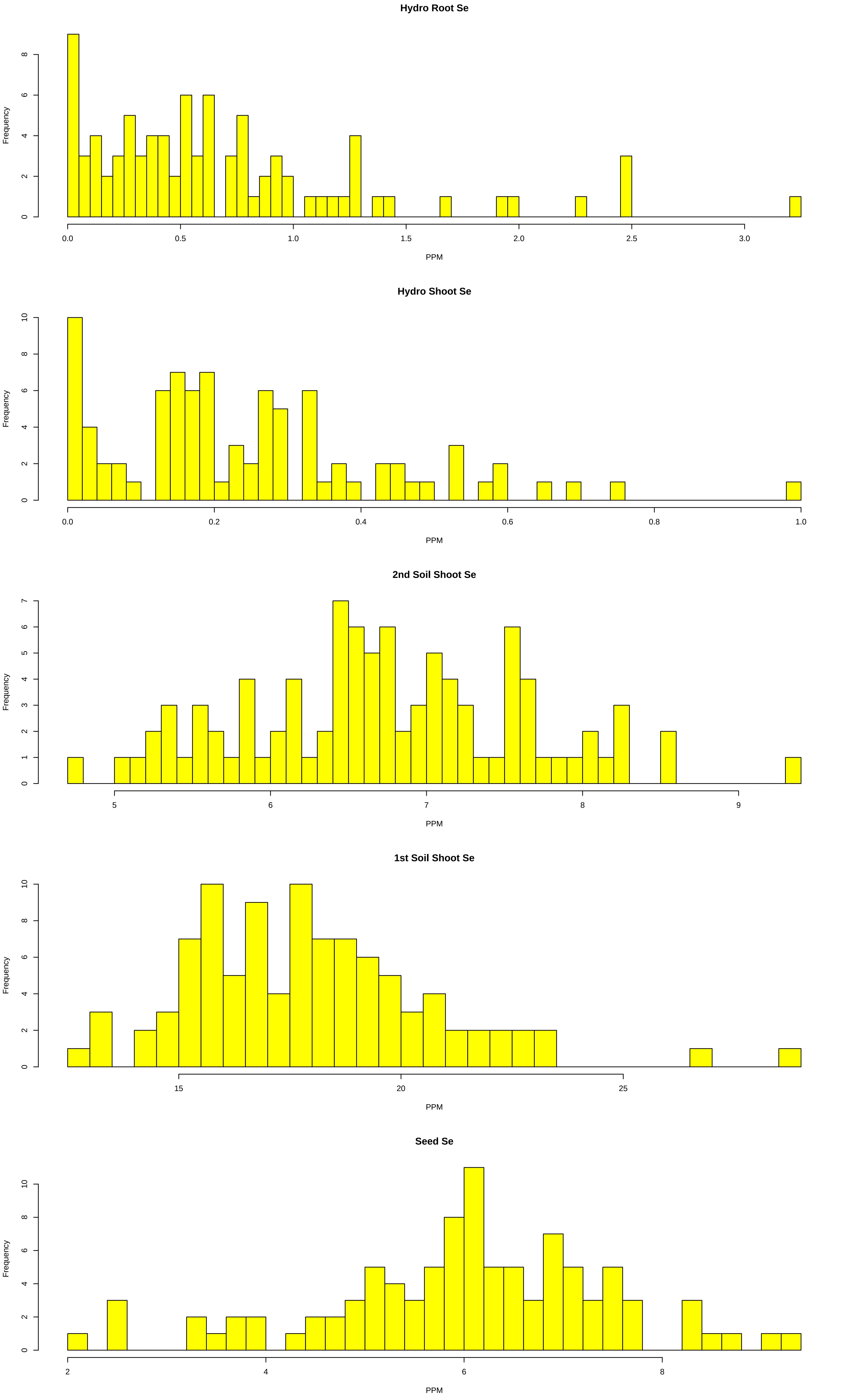
Hydro Root Se
Frequency
0
2
4
6
8
0.0
0.5
1.0
1.5
2.0
2.5
3.0
PPM
Hydro Shoot Se
Frequency
0
2
4
6
8
10
0.0
0.2
0.4
0.6
0.8
1.0
PPM
2nd Soil Shoot Se
Frequency
0
1
2
3
4
5
6
7
5
6
7
8
9
PPM
1st Soil Shoot Se
Frequency
0
2
4
6
8
10
15
20
25
PPM
Seed Se
Frequency
0
2
4
6
8
10
2
4
6
8
PPM

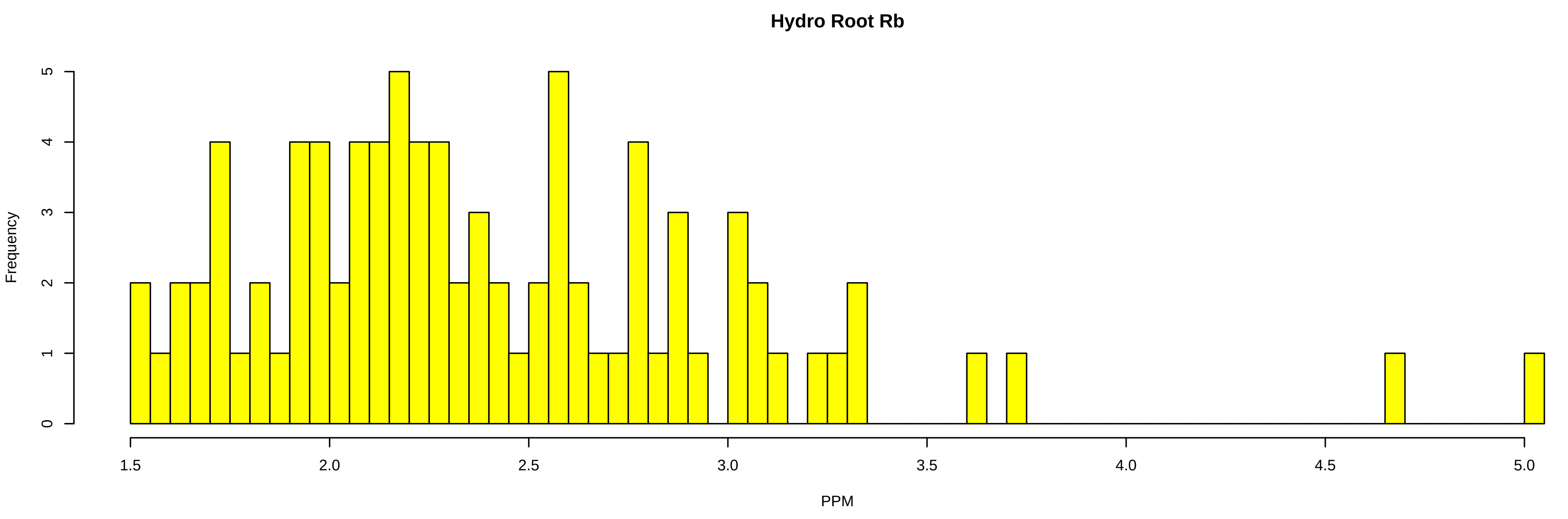
Hydro Root Rb
Frequency
PPM

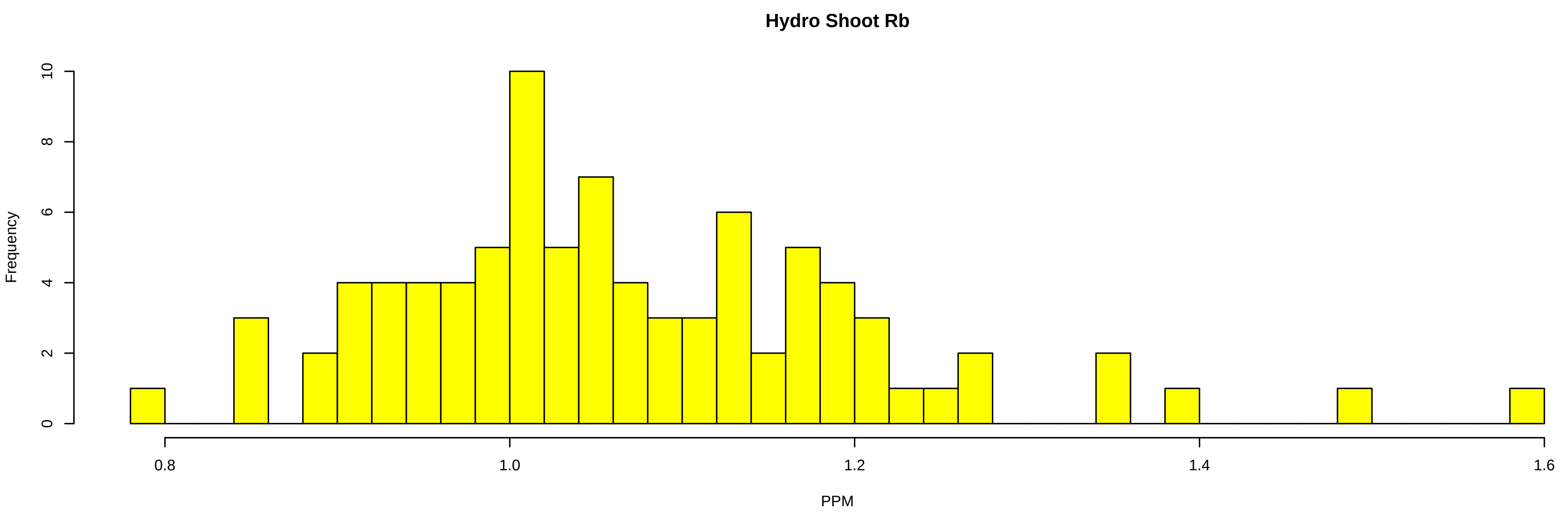
Hydro Shoot Rb
Frequency
PPM

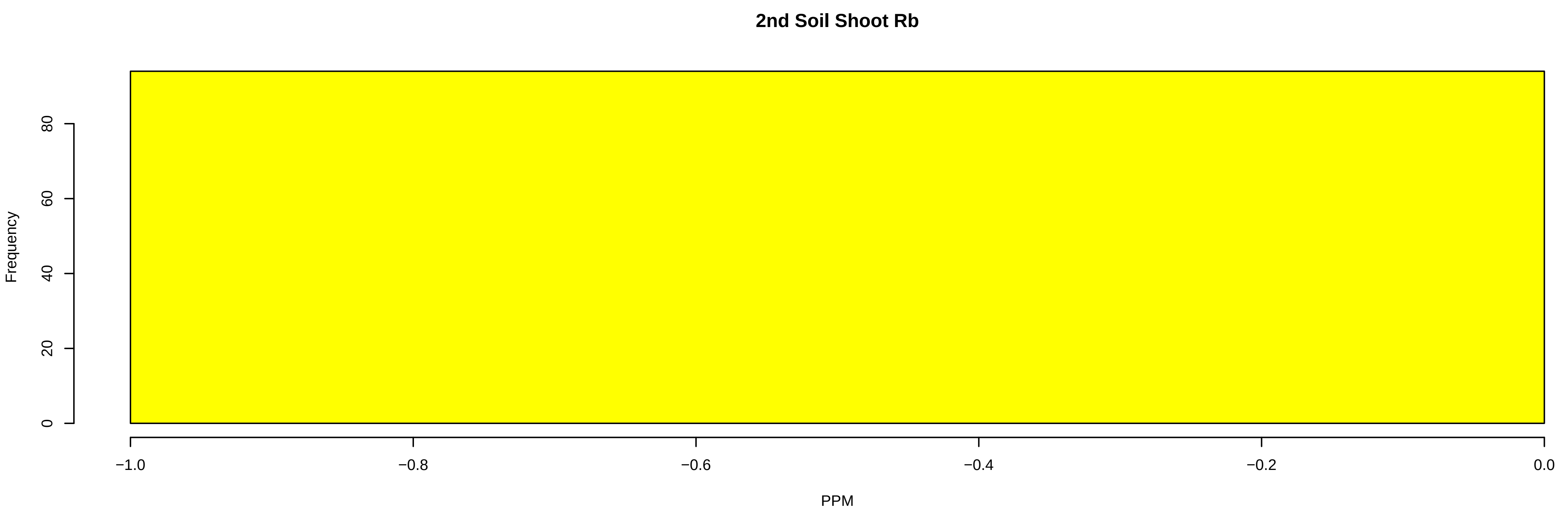
2nd Soil Shoot Rb
Frequency
PPM

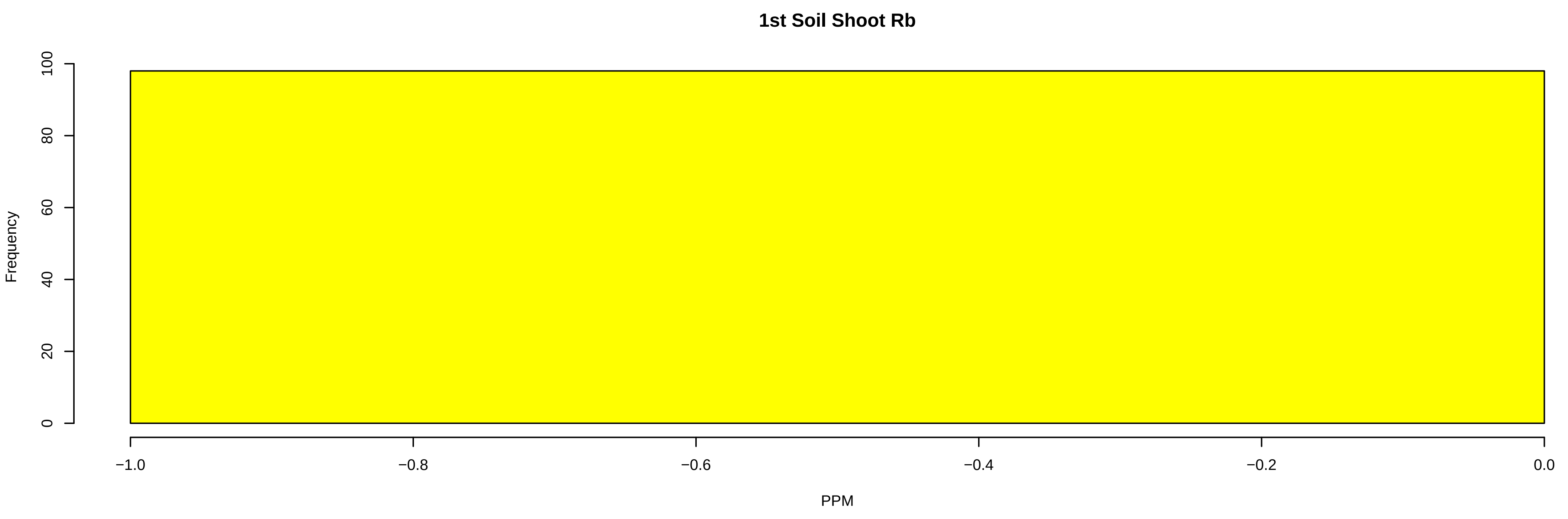
1st Soil Shoot Rb
Frequency
PPM

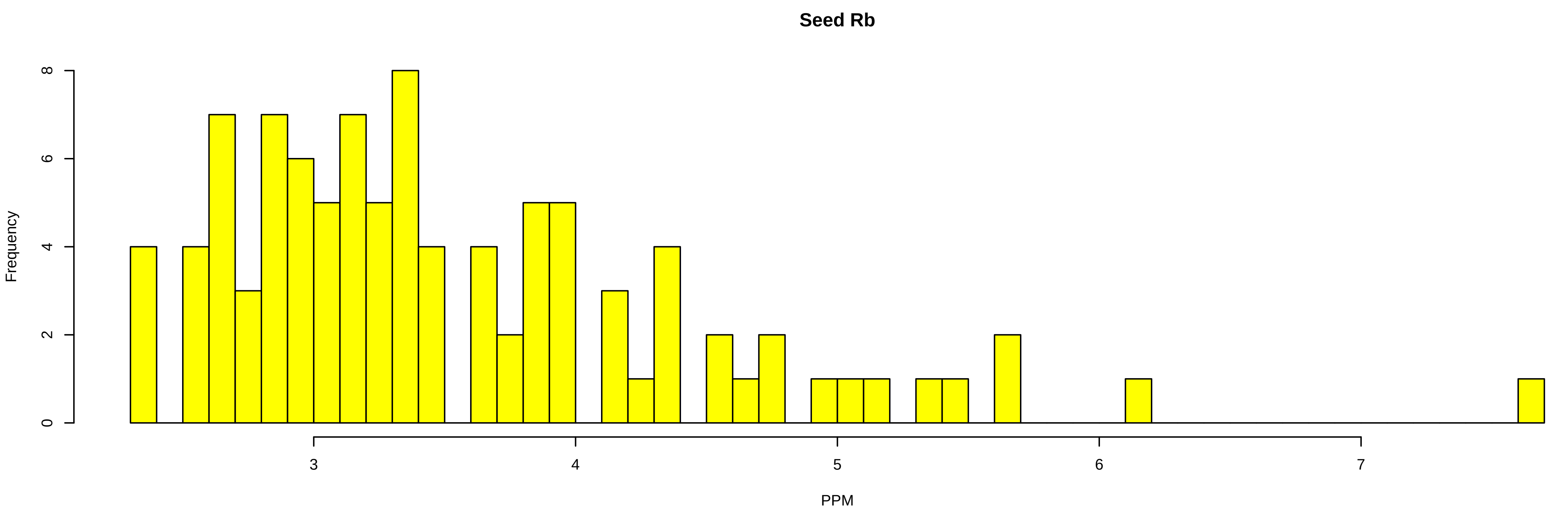
Seed Rb
Frequency
PPM

Hydro Root Mo

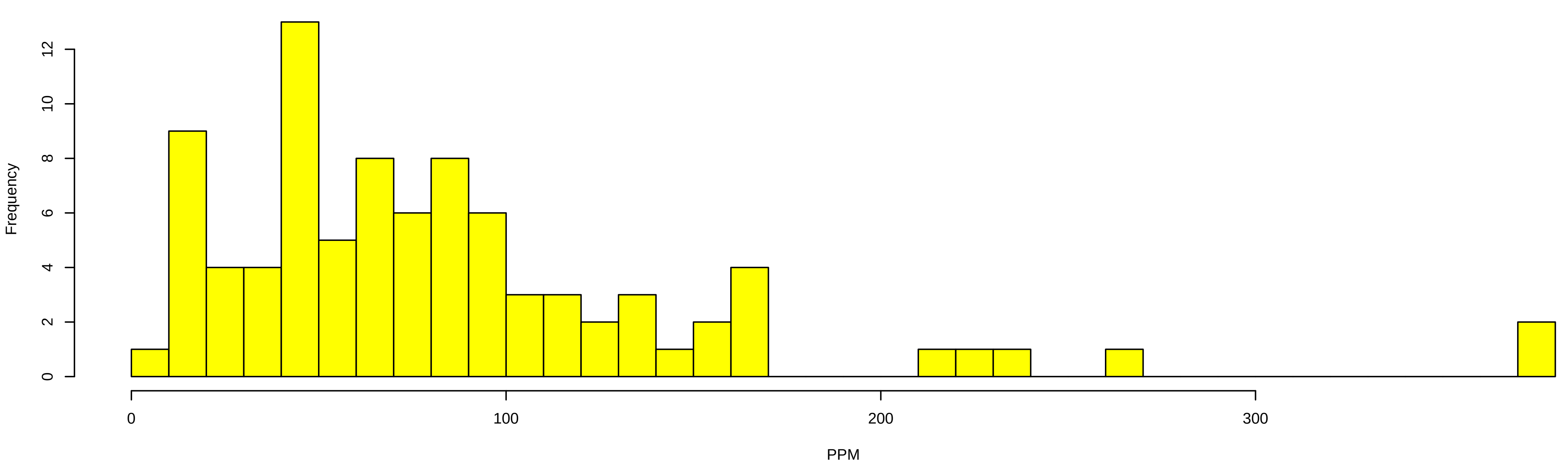

Hydro Shoot Mo

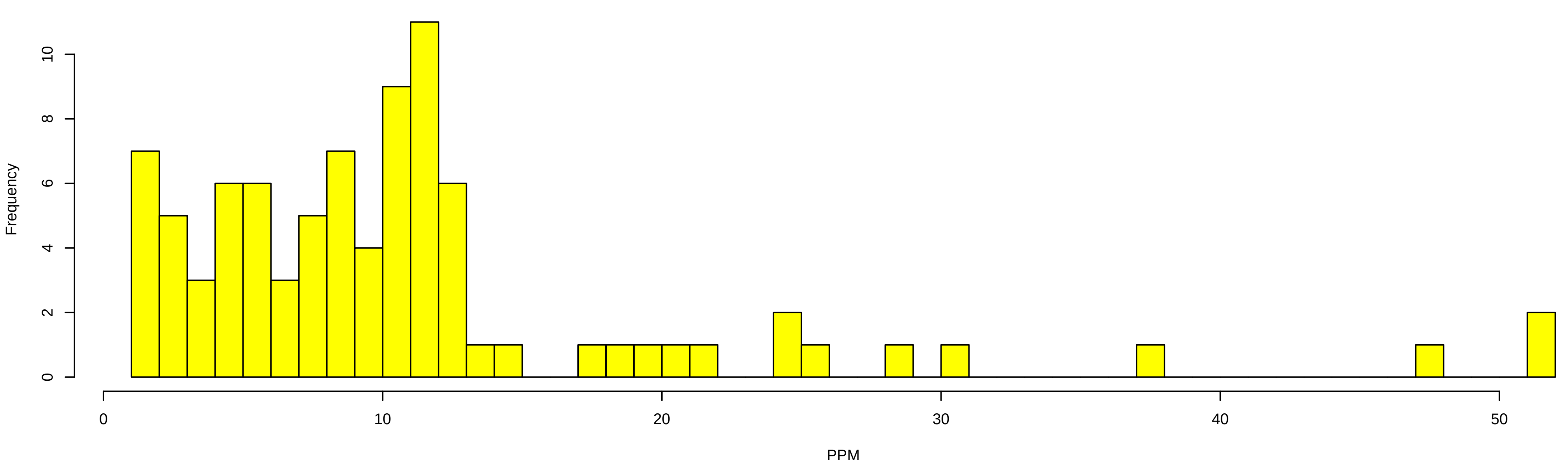

2nd Soil Shoot Mo

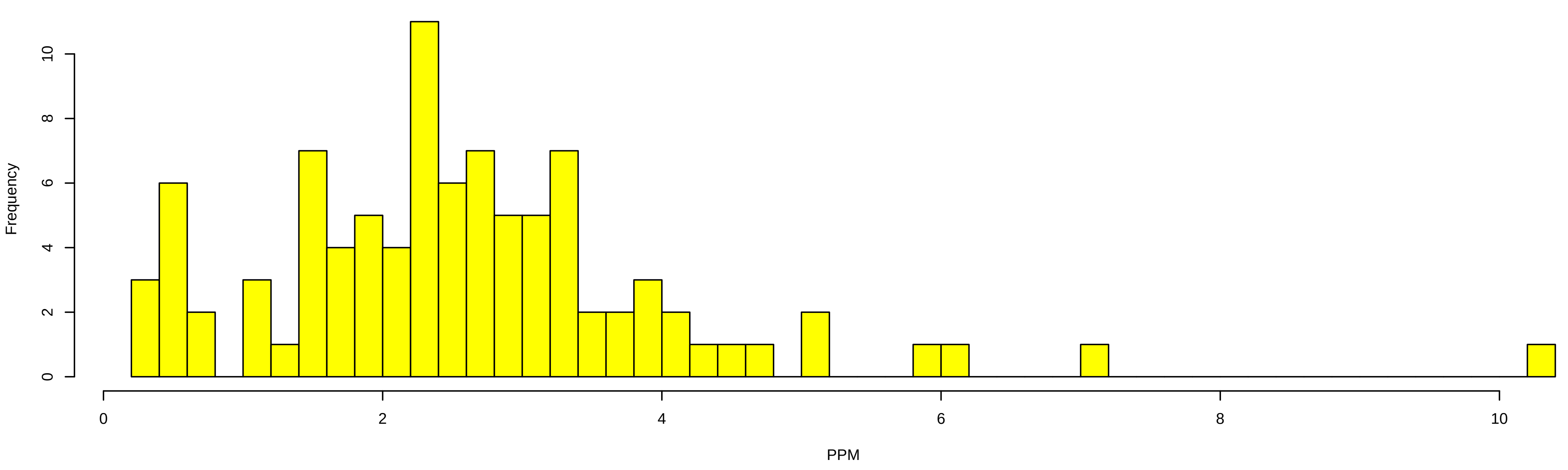

1st Soil Shoot Mo

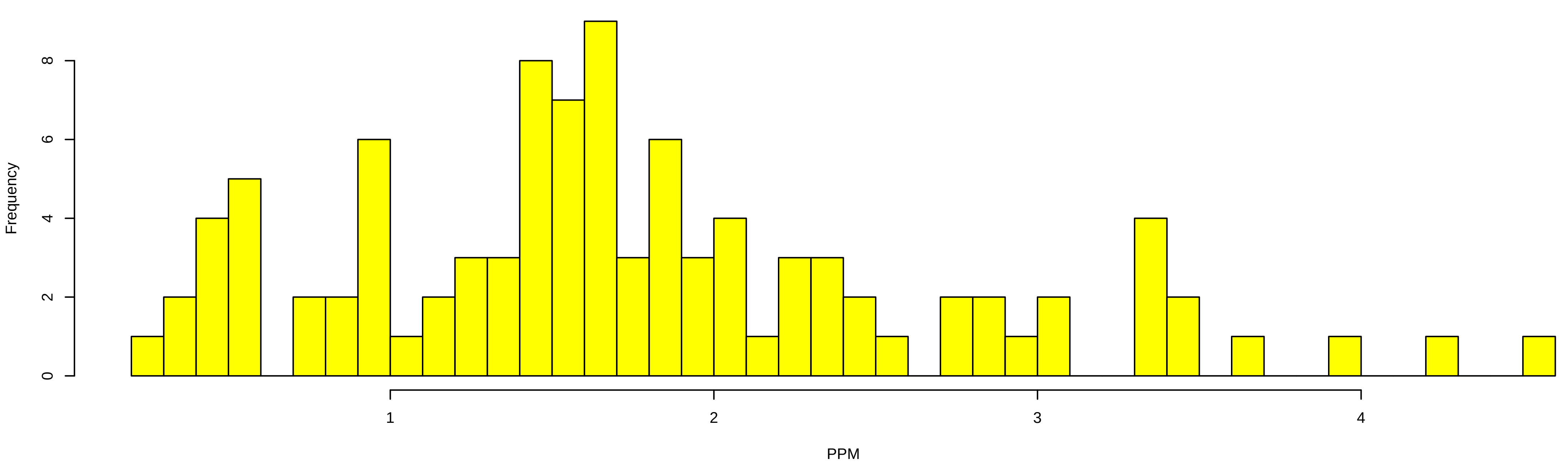

Seed Mo

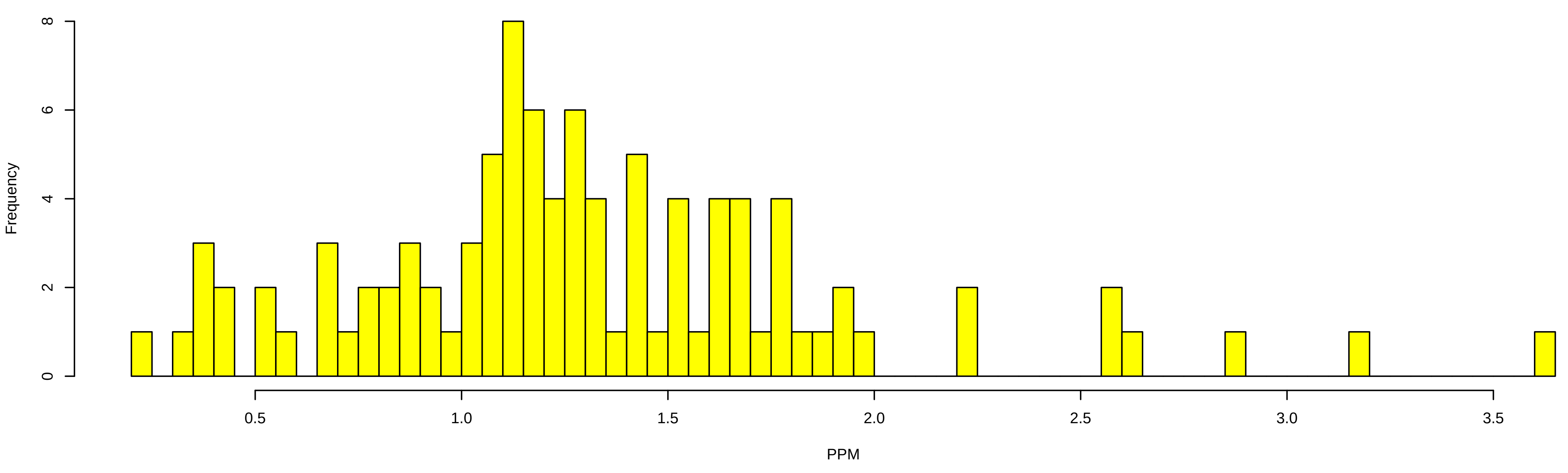

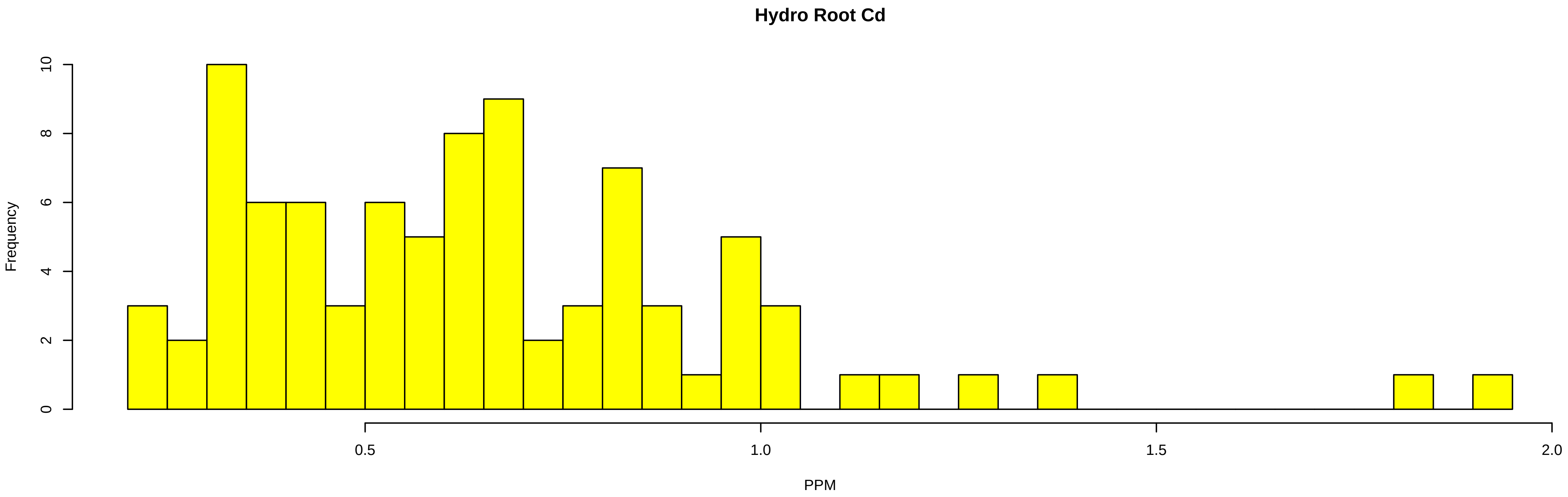
Hydro Root Cd
Frequency
0 2 4 6 8 10
0.5 1.0 1.5 2.0
PPM

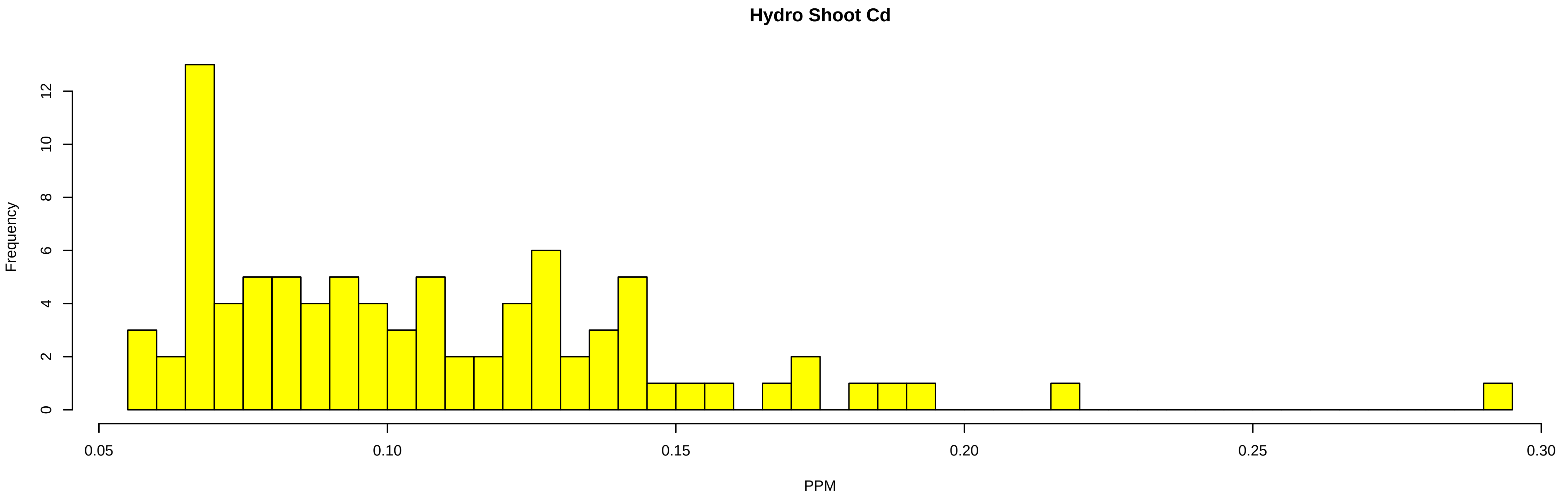
Hydro Shoot Cd
Frequency
0 2 4 6 8 10 12
0.05 0.10 0.15 0.20 0.25 0.30
PPM

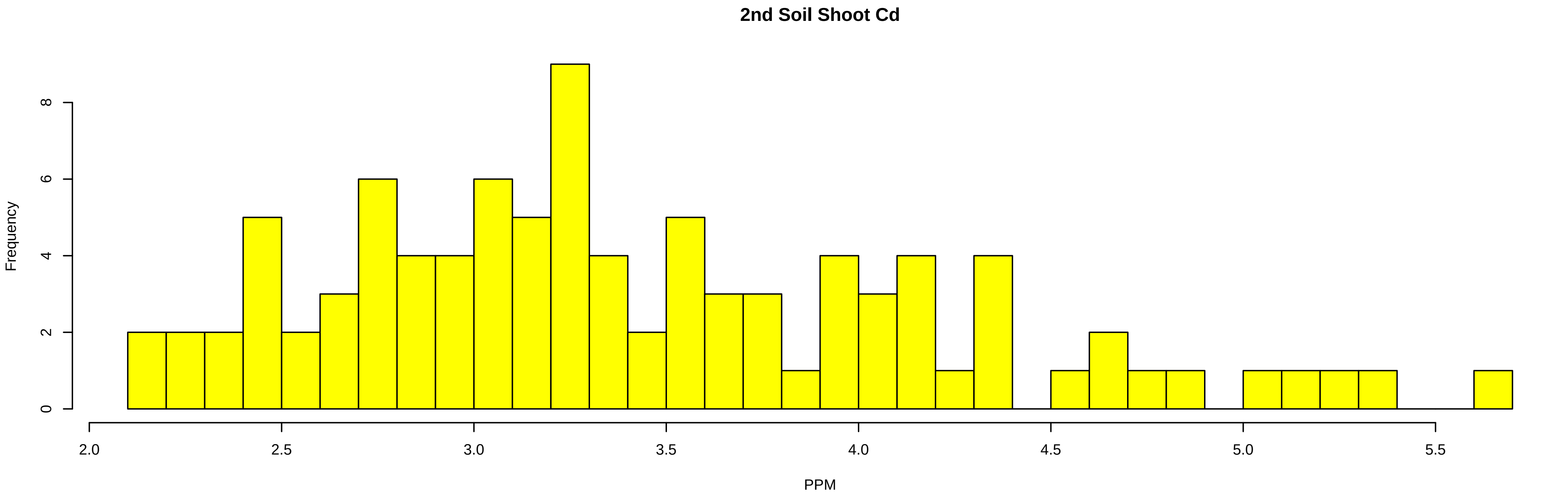
2nd Soil Shoot Cd
Frequency
0 2 4 6 8
2.0 2.5 3.0 3.5 4.0 4.5 5.0 5.5
PPM

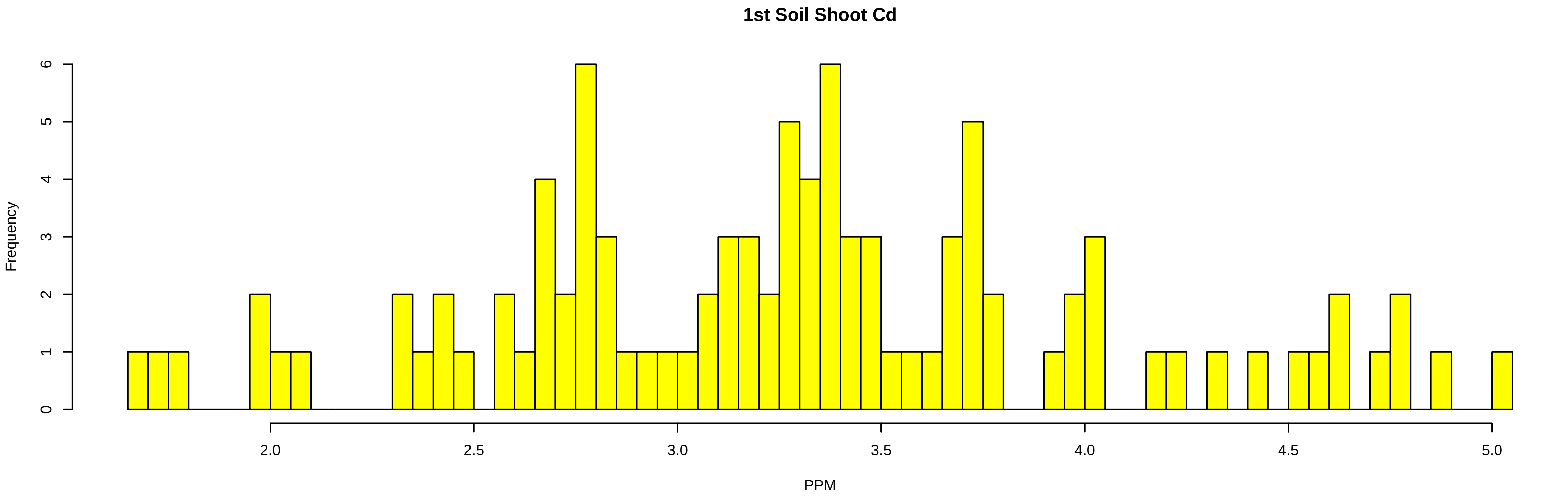
1st Soil Shoot Cd
Frequency
0 1 2 3 4 5 6
2.0 2.5 3.0 3.5 4.0 4.5 5.0
PPM

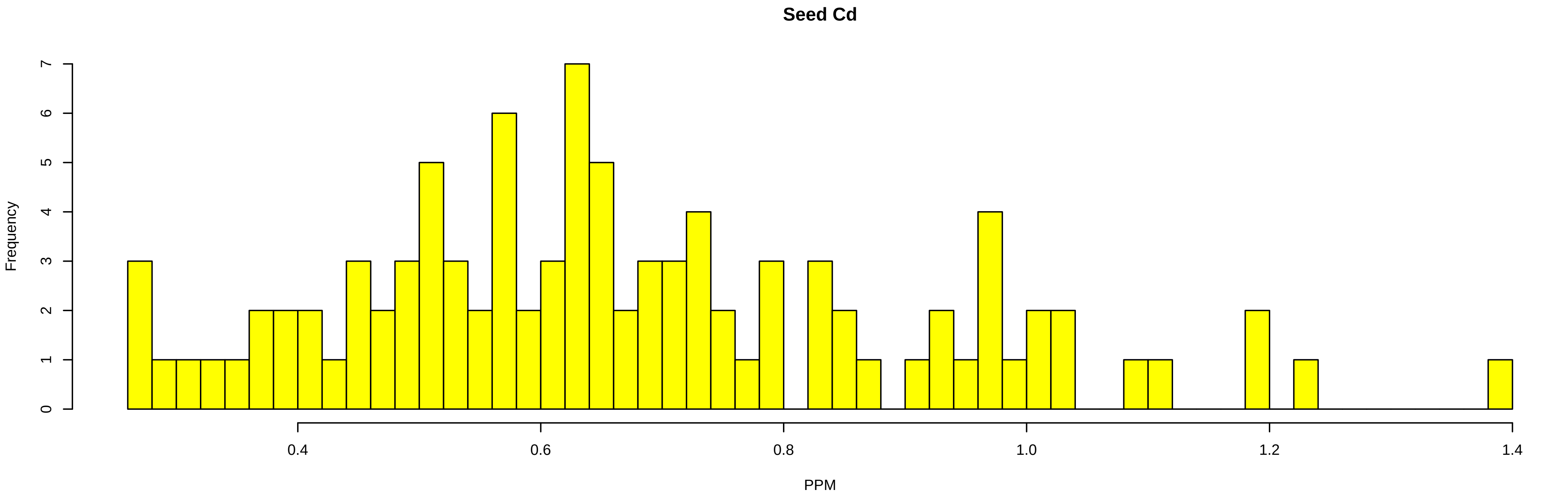
Seed Cd
Frequency
0 1 2 3 4 5 6 7
0.4 0.6 0.8 1.0 1.2 1.4
PPM
